# Evolution of the eukaryotic dynactin complex, the activator of cytoplasmic dynein

**DOI:** 10.1186/1471-2148-12-95

**Published:** 2012-06-22

**Authors:** Björn Hammesfahr, Martin Kollmar

**Affiliations:** 1 Abteilung NMR basierte Strukturbiologie, Max-Planck-Institut für Biophysikalische Chemie, Am Fassberg 11, Göttingen D-37077, Germany

## Abstract

**Background:**

Dynactin is a large multisubunit protein complex that enhances the processivity of cytoplasmic dynein and acts as an adapter between dynein and the cargo. It is composed of eleven different polypeptides of which eight are unique to this complex, namely dynactin1 (p150^Glued^), dynactin2 (p50 or dynamitin), dynactin3 (p24), dynactin4 (p62), dynactin5 (p25), dynactin6 (p27), and the actin-related proteins Arp1 and Arp10 (Arp11).

**Results:**

To reveal the evolution of dynactin across the eukaryotic tree the presence or absence of all dynactin subunits was determined in most of the available eukaryotic genome assemblies. Altogether, 3061 dynactin sequences from 478 organisms have been annotated. Phylogenetic trees of the various subunit sequences were used to reveal sub-family relationships and to reconstruct gene duplication events. Especially in the metazoan lineage, several of the dynactin subunits were duplicated independently in different branches. The largest subunit repertoire is found in vertebrates. Dynactin diversity in vertebrates is further increased by alternative splicing of several subunits. The most prominent example is the dynactin1 gene, which may code for up to 36 different isoforms due to three different transcription start sites and four exons that are spliced as differentially included exons.

**Conclusions:**

The dynactin complex is a very ancient complex that most likely included all subunits in the last common ancestor of extant eukaryotes. The absence of dynactin in certain species coincides with that of the cytoplasmic dynein heavy chain: Organisms that do not encode cytoplasmic dynein like plants and diplomonads also do not encode the unique dynactin subunits. The conserved core of dynactin consists of dynactin1, dynactin2, dynactin4, dynactin5, Arp1, and the heterodimeric actin capping protein. The evolution of the remaining subunits dynactin3, dynactin6, and Arp10 is characterized by many branch- and species-specific gene loss events.

## Background

Dynactin is a multisubunit protein complex in eukaryotic cells required as an activator of cytoplasmic dynein, the major minus end-directed microtubule motor [1,2]. Dynactin acts as an adapter between dynein and the cargo [2-4] and enhances the movement of dynein by increasing its processivity [5-8]. The dynein-dynactin complex plays an important role during mitosis [9,10] and is necessary for synapse stabilization [11]. It is involved in nuclear migration, and during cell division in mitotic spindle positioning [12-14] and organization of spindle microtubule arrays [15]. Although most of dynactins functions are in conjunction with cytoplasmic dynein it also binds to and modulates kinesin-2 [16,17] and kinesin-5 [18].

Dynactin is composed of eleven different subunits ranging in size from 22 to 150 kDa [19]. Several components are present as dimers or oligomers in the complex resulting in an overall molecular weight of 1.2 MDa. The novel dynactin subunits have initially been named according to the molecular weights of the vertebrate subunits in SDS gels [1]. However, as the molecular weights differ between species the original naming is not adequate to describe the protein family relation of the subunits in all eukaryotes. Therefore and because these subunits are unique to the dynactin complex we adopt and use the nomenclature dynactin1 to dynactin6 (symbols DCTN1 to DCTN6), which has recently been established by the HUGO Gene Nomenclature Committee (HGNC; [20]), throughout this analysis.

The structure of the complex can be divided into two distinct domains: the Arp1 rod and the projecting arm [21,22]. The projecting arm (consisting of the so-called sidearm and shoulder complex) links dynactin to cytoplasmic dynein, kinesin motors, and microtubules. It is composed of two dynactin1 (p150^Glued^), four dynactin2 (p50 or dynamitin), and two dynactin3 subunits (p24 and p22 have been used for the mouse and the human ortholog, respectively). The Arp1 rod is built of eight Arp1 molecules forming a short actin-like filament, probably one β-actin molecule, and the conventional actin capping proteins Capα and Capβ, which are located at the barbed-end of the mini-filament. The other end of the filament is terminated by Arp10 (the name Arp11 is synonymously used for the vertebrate orthologs [20]) and dynactin4 (p62), to which the dynactin5 (p25) and dynactin6 (p27) subunits are associated. The heterotetrameric complex of dynactin4, dynactin5, dynactin6 and Arp10 is also called pointed-end complex.

Dynactin1 is the largest subunit of the dynactin complex [23] and belongs to the microtubule plus end-binding protein family [24]. The microtubule-binding CAP-Gly (cytoskeleton-associated protein-glycine-rich) domain is located at the N-terminus [23,25]. The CAP-Gly domain is connected to the other subunits of the complex via two long coiled-coil regions. The first coiled-coil region following the CAP-Gly domain binds to the intermediate chain of cytoplasmic dynein [26,27]. Dynactin2 is the connection between the projecting arm and the Arp1 rod [28,29] and its over-expression *in vivo* causes disruption of the dynactin complex [21,28,30]. Dynactin3 is required for attachment of dynactin1 to dynactin2 [31]. Arp1 is the actin-related protein most similar to actin and forms an actin-like mini-filament [22] that represents the backbone of dynactin, to which the other dynactin subunits bind. It is supposed that membranous cargoes bind to dynactin via the Arp1 rod [4,32,33].

The first studies on dynactin have been performed with chicken brain samples [1,2]. Subsequently, dynactin subunits have been identified and analyzed in the model organisms *Neurospora crassa*[34-39], *Saccharomyces cerevisiae*[40-43], *Drosophila melanogaster*[44,45] and *Caenorhabditis elegans*[45-47]. Although the composition of the dynactin complex in vertebrates gradually became apparent, a thorough analysis of the complex and its subunits in terms of gene duplicates, alternatively spliced isoforms, and phylogenetic evolution is still missing. That a surprising diversity might be found has been shown by a recent study of the motor protein repertoire of 21 insect genomes uncovering a branch specific duplication of the well-known dynactin1 (p150^Glued^) gene in *Drosophila* species [48].

Building such a multi-protein complex with a filament of fixed size seems rather complicated. Because most of the analyses of the complex have been done with vertebrate samples, it would be interesting to see whether the various unicellular protists that often have smaller gene repertoires, may have evolved compacted versions of the dynactin complex. Vice-versa, there could have been a minimal dynactin complex at the origin of the eukaryotes that multicellular eukaryotes expanded to accomplish more and different tasks. Here, we examined every known protein of the complex and determined its absence and presence in all eukaryotic genomes as available in September 2011. Furthermore, we inspected all genes to identify alternatively spliced exons and their appearance during evolution. For our analysis, we manually assembled and annotated more than 4,700 dynactin and actin-related protein sequences from about 550 species. All sequences were inspected and validated at the genomic DNA level to remove wrongly predicted sequence regions, to manually fill gaps in gene predictions, and to reveal the correct exon/intron boundaries. The sequences and related data like gene structure reconstructions and biochemical properties are available through CyMoBase ( http://www.cymobase.org).

## Results

### Identification of dynactin genes

Dynactin protein sequences are not as strongly conserved as for example tubulins, and three of the dynactin subunits are relatively short complicating their identification if they were spread on several exons. In addition, dynactin contains two actin-related proteins of which Arp1 is closely related to actin while Arp10 is a very divergent member thus hindering their immediate identification. The dynactin subunits might have been duplicated in single species or certain branches, like the *Drosophila* dynactin1 gene [48]. These events can only be revealed through the phylogenetic analysis of the corresponding protein sequences. Thus, it is of major importance to obtain the best sequence data possible and to create the most accurate multiple sequence alignments. Automatic gene predictions are error-prone (for example, automatic gene prediction programs do not recognize GC---AG intron splice sites), and even those gene predictions are available for only a small subset of all sequenced eukaryotic genomes [49]. Therefore, we manually assembled and annotated all dynactin and actin-related sequences used in this study. Manual identification and assembly means that we started from a set of sequences verified by cDNA and used those for searches with standard tools like TBLASTN in the genome assemblies. Unfortunately, only a few full-length mRNA/cDNA sequences for dynactin subunits are available, which served as representatives for correct sequences. Every search hit has further been analysed by manual inspection of the corresponding genomic DNA sequence either to reveal the correct intron/exon boundaries or to extend hits that only covered short parts of the search sequence. Those sequences were excluded, for which some local similarity was identified (e.g. similarity to the dynactin1 CAP-Gly domain) but for which the remaining parts of the respective subunits could not be found although the genomic sequences of the respective contigs were long enough. Genomes, for which the respective dynactin subunits could not unambiguously be assembled in the first instance, were reanalysed as soon as further data was added to the multiple sequence alignments. In this way the completeness of the search for dynactin subunits and the accuracy of the gene assembly and annotation has continuously been re-evaluated and improved. In addition to manually assembling all sequences, the multiple sequence alignments of the dynactin sequences have been created and were maintained and improved manually (Additional file 1).

Sequences of which small parts were missing due to gaps in the genome assemblies (up to 5%) were termed “Partials”. “Partials” are not expected to considerably influence the phylogenetic tree computations. Sequences of which more than 5% were missing due to genome assembly gaps or incomplete EST data but that are otherwise unambiguous orthologs or paralogs were termed “Fragments”. "Fragments" are important to denote the presence of the subunits in the respective species in the qualitative analysis. Dynactin genes were termed pseudogenes if they contain more features like frame shifts and in-frame stop codons and miss more conserved sequence regions than can be attributed to sequencing or assembly errors.

In total, the dynactin dataset contains 3061 sequences from 478 organisms (Table 1, Additional file 2), of which 2872 have been derived from 353 WGS sequencing projects. 2668 sequences are complete, and an additional 191 sequences are partially complete. In addition, 1766 actin and actin-related proteins from 323 species have been assembled to finally reveal the subfamily relationship of potential Arp1 and Arp10 orthologs in questionable cases. For plotting the presence or absence of dynactin subunits across the tree of the eukaryotes we only included those species whose genomes have been sequenced with high coverage and which provided reliable data in many other cases [48,50-52]. Nevertheless, low-coverage genomes have also been analysed because every single piece of sequence could be very important to resolve ambiguous regions in related species or to clarify phylogenetic question. For example, we also analysed the incomplete genome of the agnath *Petromyzon marinus* to reveal at which stage alternative splice forms had been evolved in vertebrate evolution. To infer the phylogenetic relationship of duplicated dynactin subunits we calculated phylogenetic trees using the Maximum Likelihood and Bayesian methods. Gene structures were reconstructed for all sequences using WebScipio [53] and can be inspected via CyMoBase ( http://www.cymobase.org) for further investigation.

**Table 1:** Data statistics

	**Total**	**Dynactin1 p150 ropy-3 Nip100**	**Dynactin2 p50 Jnm1**	**Dynactin3 p24 ropy-10 Ldb18**	**Dynactin4 p62 ropy-2**	**Dynactin5 p25 ropy-12**	**Dynactin6 p27**	**Cap1 (Capα)**	**Cap2 (Capβ)**	**Arp1 ropy–4**	**Arp10 Arp11 ropy–7**
**Sequence**											
Total	3061	321	312	278	322	326	258	368	299	306	271
From WGS	2872	300	280	248	289	296	229	359	298	304	269
Pseudogenes	60	1	6	3	19	12	5	14	0	0	0
**Completeness**											
Complete	2668	246	259	250	250	273	220	345	286	293	246
Partials	191	27	14	23	28	31	27	10	4	10	17
Fragments	181	48	37	5	44	12	9	6	9	3	8
**Species**											
Total	2863	288	306	274	301	313	246	289	292	287	267
WGS-projects	2567	257	261	238	256	271	215	270	278	269	252
EST-projects	960	86	106	96	103	113	93	102	102	87	72
WGS- and EST-projects	1314	124	135	116	134	148	115	146	149	133	114
**Sequences in Taxa**											
Metazoa	1339	155	132	141	159	158	132	167	94	101	100
Fungi	1339	144	140	118	124	118	96	138	141	165	155
Apusozoa	9	0	1	1	1	1	1	1	1	1	1
Amoeba	56	5	5	4	5	5	5	10	8	4	5
SAR	227	15	28	10	27	32	17	30	29	30	9
Excavata	21	0	1	0	1	5	2	4	7	1	0
Viridiplantae	53	0	0	0	0	0	0	27	26	0	0
Rhodophyta	0	0	0	0	0	0	0	0	0	0	0
Glaucophyta	5	0	1	1	1	2	0	1	1	0	0
Cryptophyta	3	0	1	0	0	1	1	0	0	0	0
Haptophyta	0	0	0	0	0	0	0	0	0	0	0

### Dynactin1

**Figure 1:**
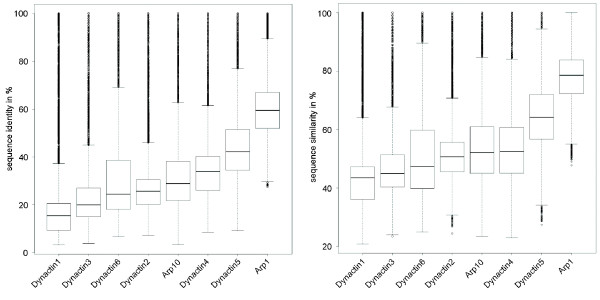
**Sequence conservation in dynactin subunits.** Box plots of the sequence identities and similarities of the dynactin subunits.

Dynactin1 plays a major role for the function of the dynactin complex as it connects the Arp1 rod, and thus the cargo binding sites, to cytoplasmic dynein, the transporter protein complex, and to microtubules, the track. It can hardly be imagined to build a functional dynactin complex without a dynactin1 subunit. However, dynactin1 is also the least conserved of the dynactin subunits (Figure 1). This is most likely due to its domain structure that consists of a short N-terminal globular CAP-Gly domain followed by two coiled-coil regions, which account for two thirds of its primary sequence. Both the region separating the two coiled-coil regions and the C-terminal region are not even conserved between metazoan and fungal dynactin1 subunits, which belong to the opisthokont branch. Given the functional importance of dynactin1 we were surprised not to be able to identify homologs in any Apicomplexa, in the Heterolobosea *Naegleria gruberi*, and the Apusozoa *Thecamonas trahens* (Table 1). When searching for dynactin1 homologs in these organisms we analysed all TBLASTN and PSI-BLAST hits showing sequence similarity to CAP-Gly domains but we only found other CAP-Gly domain containing proteins like CLIP-170/restin [54], and the tubulin-specific chaperones B and E [55,56].

Duplicates of dynactin1 have been found in independent branches of the eukaryotic tree (Additional file 3). In the Brachycera branch (including the *Drosophila* clade) the dynactin1 gene has been duplicated once [48]. Another duplication of dynactin1 was found in the Actinopterygii branch, supported by *Brachydanio rerio*, *Takifugu rubripes*, and *Gasterosteus aculeatus*. Some of the nematods like *Brugia malayi* also encode two versions of dynactin1. Two duplications of dynactin1 were found in the genome of the fungus *Rhizopus arrhizus*, and one additional dynactin1 in *Mucor circinelloides*. The variant A and B subunits each grouped together, suggesting a gene duplication predating the separation of the two species. Variant C of *Rhizopus arrhizus* grouped to variant B indicating another *Rhizopus*-specific duplication.

**Figure 2:**
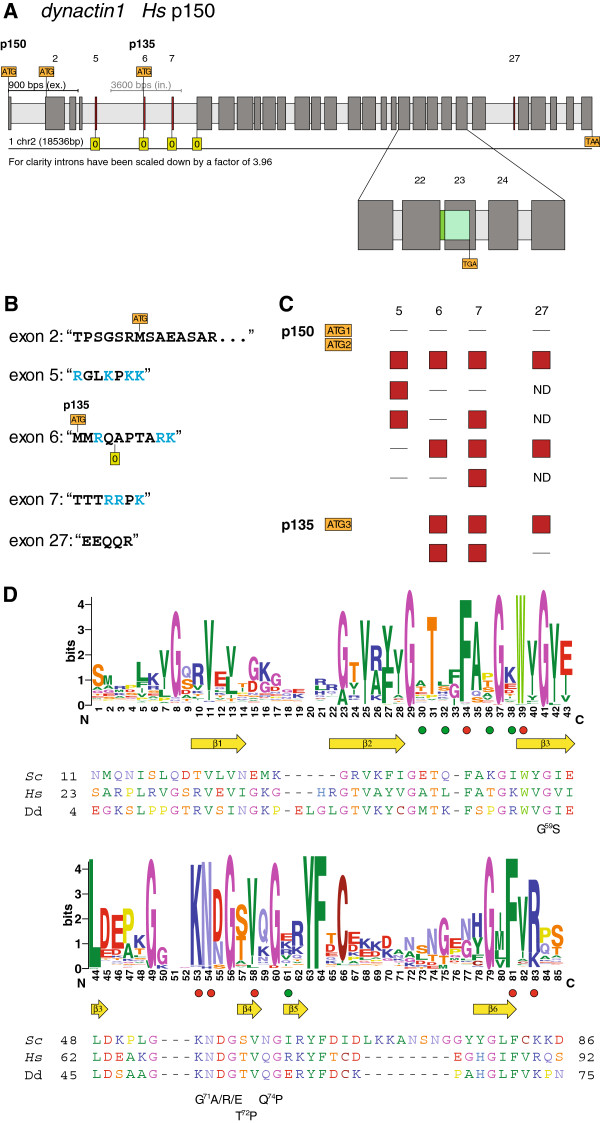
**Gene structure and isoforms generated by alternative splicing of dynactin1.** The gene structure was reconstructed with WebScipio and represents the dynactin1 (p150) homolog of *Homo sapiens* encoded by 32 exons including four alternatively spliced exons **(A)**. Dark gray bars represent exons, light gray bars indicate introns, and coloured bars symbolize the alternatively spliced exons. For better visualisation, exons and introns are scaled differently. ATG in orange rectangles represent translation start positions. Translation start codons exist in exons 1, 2, and 6, respectively. The zero in green rectangles represents the first reading frame. A zoomed view on the exons 21–25 shows intron retention of intron 22 (dark-green bar) that results in the translation of exon 23 in a different reading frame leading to a premature stop codon (light-green bar). The protein sequences for the alternative exons are given **(B)** as well as a short summary of the combinations of the alternatively spliced exons that have been found in full-length cDNA data **(C)**. Due to missing full-length cDNA sequences the inclusion or exclusion of alternative exon 27 could not be determined for all combinations of exons 5 to 7 (ND = not determined). The sequence logos **(D)** illustrate the sequence conservation within the multiple sequence alignment of the CAP-Gly domain. For better orientation, the sequences of three representative CAP-Gly domains are shown: the human CAP-Gly domain as the main target of disease associated mutations, the *Saccharomyces cerevisiae* and the *Dictyostelium* CAP-Gly domains as representatives of widely used model organisms. β-strands as determined from the crystal structure are drawn as yellow arrows. Green dots point to amino acids of the human CAP-Gly domain that have been proposed to constitute the second EB1-binding site [59] and red dots highlight residues that are part of the conserved EEY/F motif binding site [25,59,60]. Some mutations as found in human diseases are given below the reference sequences with numbering referring to human dynactin1.

The dynactin1 gene of *Homo sapiens* is encoded in 32 exons on chromosome 2 (Figure 2A, [57]). All exons are constitutively expressed and present in all dynactin1 transcripts, except for exon 5 (“RGLKPKK”), the second part of exon 6 (“APTARK”), exon 7 (“TTTRRPK”), and exon 27 (“EEQQR”) that are alternatively spliced (Figure 2B). Some alternative transcripts have already been described based on the analysis of a fetal human cDNA library (dynactin1-Δ5; dynactin1-Δ5,6: dynactin1-Δ5,6,7; [58]) suggesting that exons 5–7 are each differentially included. In order to reveal a more general view of possible transcripts we extensively searched for corresponding sequences of vertebrate species in the available EST and cDNA databases and found the following combinations for exons 5–7 (Figure 2C):

none of the alternative exons is included in the transcript (Δ5,6,7)

exon 5 included, resulting in four additional positively charged residues (lysines or arginines, Δ6,7)

exon 7 included, three additional positively charged residues (Δ5,6)

exon 5 and 7 included, seven additional positively charged residues (Δ6)

exon 6 and 7 included, five additional positively charged residues (Δ5)

exon 5, 6 und 7 included, nine additional positively charged residues

We did not find EST or cDNA-data for transcripts including only exon 6 (Δ5,7), or EST-data including exons 5 and 6 without exon 7 (Δ7). Exon 27 is also a differentially included exon. Maybe because of lack of more full-length cDNA data or maybe because of tight regulation, exon 27 is found to be absent in dynactin1-Δ5,6,7, and to be present in dynactin1-Δ5 and dynactin1 (Figure 2C). In addition, transcripts are generated from three alternative start positions. The first is at the beginning of exon 1, the second is at the beginning of exon 2, and the third possible transcript starts with exon 6 (“MMRQAPTARK…”), which corresponds to the “p135” construct. While transcript start sites 1 and 2 are found in all described combinations of exons 5–7 and exon 27, transcript start site 3 (exon 6) is only found in combination with exon 7 included and either exon 27 included or spliced out.

Interestingly, the alternative exons encode different numbers of basic residues, arginines and lysines. Although only six of the eight possible combinations of the alternative exons have been found in EST and cDNA data so far, vertebrates seem to be able to stepwise increase the number of basic residues in this region from zero to nine. The basic residues influence the sliding behaviour of dynactin along the microtubules with fewer charges allowing a faster diffusion [58]. The function of the region including the fourth differentially included exon, exon 27, which is located subsequent to the second coiled-coil region and thus behind a proposed Arp1 binding site [23], has not been analysed so far. While the third transcription start site produces a dynactin1 without a CAP-Gly domain (“p135”) the functional difference between transcripts of the two other transcription start sites is not known yet. The longer N-terminus (about 20 residues) is not visible in any of the available crystal structures of dynactin1 CAP-Gly domains [25,59-61]. In addition, a solution state structure (PDBid 2COY) revealed that the N-terminus is an unstructured and unordered coil.

There is another alternative transcript generated by retention of intron 22 (Figure 2A). This intron retention results in a premature stop codon and has only been found in combination with transcription start site 2. The resulting transcript includes the CAP-Gly microtubule binding domain and the dynein intermediate chain binding site but stops before the second proposed coiled-coil region. The C-terminal part of dynactin1 starting with the second coiled-coil region has been proposed to bind to Arp1 and truncation mutants of *Drosophila* dynactin1 have been shown not to be incorporated into dynactin [23,62]. This most likely also accounts for the alternative transcripts including intron 22 of vertebrate dynactin1.

The alternatively spliced exons and transcription start sites are conserved in all vertebrates and were also found in the agnath *Petromyzon marinus* the sistergroup of all gnathostomes representing the deepest separation in extant vertebrates. Especially the lysines and arginines and their positions are invariant. However, in the fish type A dynactin1 subunits the exons 5 have been lost, as well as the third potential translation start in exon 6. Instead, exon 6 encodes only the part that is alternatively spliced in type B dynactin1. Thus a “p135”-like isoform cannot be build from fish type A dynactin1 subunits. Alternatively spliced isoforms have not been identified in any other of the analysed species.

The sequence conservation plot across all dynactin1 CAP-Gly domains shows that the core structure consisting of six beta-strands and several key residues for binding microtubule plus end-tracking proteins is highly conserved (Figure 2D). The key residues for binding the C-terminal EEY/F tail motifs of CLIP170, EB1 proteins, and α-tubulines are F52, W57, K68, N69, and R90 (human dynactin1 numbering, [25,59]). These are almost invariant from stramenopiles to alveolates to humans (Figure 2D). In contrast, the residues of the proposed second EB1-binding site A49, L51, T54, K56, and R76 (human dynactin1 numbering, [59]) are not conserved (Figure 2D). EB1 proteins are present in all eukaryotes (plants, *Giardia*, stramenopiles, Alveolata, *Trichomonas*, Opisthokonts, data not shown). Thus, this proposed second EB1-binding site could be specific to mammals or, most likely, be an artefact from crystal packing effects. The latter is supported by another crystal structure of the complex of the dynactin CAP-Gly domain and the C-terminus of EB1, in which only the C-terminal EEY motif binds to dynactin1 [60]. Several mutations in the CAP-Gly domain of human dynactin1 are associated with diseases. The G59S mutation has been identified in patients with distal spinal bulbar muscular atrophy (dSBMA, [63]) and the G71R/E/A, T72P, and Q74P mutations have been found in patients with Perry’s syndrome [64]. All mutations lead to destabilization of the CAP-Gly domain [65]. The two glycines G59 and G71 are invariant in all dynactin1 CAP-Gly domains. While the threonine and glutamine are variable across the eukaryotes prolines are never found at these positions (Figure 2D).

### Dynactin2

Dynactin2 was found in almost all branches of the eukaryotic tree that contain a dynactin complex (Table 1). The only two species containing a likely functional dynactin complex without dynactin2 are the closely related yeasts *Ogataea parapolymorpha* and *Ogataea angusta*. Because two different species of *Ogataea* have been sequenced it is unlikely that dynactin2 could be missed because of gaps in the assemblies. None of the genomes analysed encodes more than one functional dynactin2 gene. Some mammals and *Caenorhabditis brenneri* contain dynactin2 pseudogenes.

**Figure 3:**
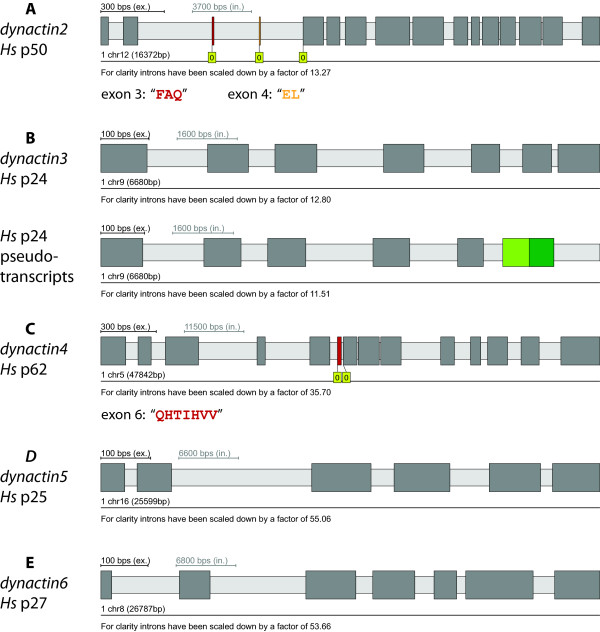
**Gene structures and alternatively spliced exons of dynactin subunits.**The gene structures including alternatively spliced exons of the dynactin subunits of *Homo sapiens* were reconstructed with WebScipio. The colour coding is the same as in Figure 2**A)** The scheme shows the gene structure of dynactin2 (p50) consisting of 16 exons including the differentially included exons 3 and 4. **B)** Gene structure of dynactin3 (7 exons). For dynactin3, pseudo-transcripts were identified (for detailed information see Additional File 4). **C)** The dynactin4 (p62) gene is comprised of 14 exons of which exon 6 is alternatively spliced. **E)** and **F)** Gene structures of dynactin5 (6 exons) and dynactin6 (7 exons), respectively.

Dynactin2 from *Homo sapiens* is encoded in 16 exons on chromosome 12 (Figure 3A). Two of the exons, the very short exons exon 3 (“FAQ”, residues 36–38) and exon 4 (“EL”, residues 39 and 40), are alternatively spliced. Both exons are independently differentially included and many EST and cDNA clones from many vertebrates exist excluding exons 3 and 4 (dynactin2-Δ3, 4) as well as including each exon separately (dynactin2-Δ3 and dynactin2-Δ4) and both exons together. The two alternatively spliced exons were also found in the agnath *Petromyzon marinus*, but not in any invertebrate and thus seem to be an invention of the most ancient vertebrate. While the up- and downstream coding sequence around exons 3 and 4 is slightly variable in vertebrates, the sequence of the two short exons is invariant. In contrast to dynactin1 we could not identify any further transcription start sites. The analysis of the available EST/cDNA data do not support alternatively spliced isoforms in any other species than vertebrates.

The first dynactin2 cDNA sequences were isolated from rat and human, and consisted of the long form including both alternative exons (isoform-1, [28]). Although immunobiochemical studies of the dynactin2 expression in various adult rat tissues have been interpreted to result from the same transcript [28] the slightly different sizes of the dynactin2 bands in the SDS-gels could in retrospect originate from the tissue-specific expression of the alternative splice forms. Later, isoform-1 and the dynactin2 isoform excluding the two alternative exons (isoform-2, dynactin2-Δ3,4) have been shown to be tissue specific transcribed [66], and very recently isoform-2 from human has been compared to chicken dynactin2-Δ3 with respect to determinants for self-oligomerization and interactions with other dynactin subunits [29].

The residues encoded by the alternative exons (residues 36 to 40) are located in the N-terminal region of dynactin2 but have not been the specific focus of any biochemical study yet. Both the N-terminal and the C-terminal part of dynactin2 are needed for proper self-assembly and binding to dynactin3. The N-terminal 100 residues seem to be required and sufficient for binding to Arp1 [29,67]. Binding essays showed that determinants for the optimal recruitment of dynactin1 are located in the N-terminal half of dynactin2 but that the N-terminal 100 residues alone are not sufficient [29]. It could thus be possible that a certain combination of alternatively spliced exons in dynactin2 correlates with the differentially inclusion of exon 27 of dynactin1. More specific experiments will be necessary to reveal how such small modifications of two to five residues could modify dynactin2’s binding to Arp1, dynactin1, and dynactin3.

### Dynactin3

We were not able to identify dynactin3 homologs in Ustilaginomycetes, Chytridiomycota, *Naegleria gruberi*, *Bigelowiella natans*, Ciliophora, plants, and Stramenopiles (Table 1). Dynactin3 homologs could also not be identified in the Schizosaccharomyces branch and most of the analysed yeast species. It has been proposed that dynactin3 is the least conserved of the dynactin subunits [45]. This analysis has been based on the comparison of the sequence identities of the dynactin subunits of chicken, *Drosophila*, *C. elegans*, and *Neurospora crassa* to the mouse subunits. In order to determine the least conserved dynactin subunit based on all eukaryotes we calculated sequence identity and similarity matrices for all subunits (Figure 1). Because the data includes sequences from all branches of the eukaryotes each subunit shows a broad distribution. The comparison of the medians of the populations shows that dynactin1 is the least conserved dynactin subunit followed by dynactin3 and dynactin6. Because we were able to identify dynactin3 in almost all opisthokonts the dynactin3 subunits have most likely been lost independently in most Saccharomyctes, the Basidiomycote *Ustilago maydis*, and in the fungi of the Chytridiomycota. Similarly we should have been able to find the dynactin3 homologs in ciliates based on the dynactin3 subunits from the Apicomplexa. The other branches, for which we could not find dynactin3 homologs, have either lost the gene or the dynactin3 proteins must be very different from the known dynactin3 subunits. *Naegleria*, *Bigelowiella*, and stramenopiles species normally do not contain intron-rich genes. Thus, it is unlikely that we missed dynactin3 subunits because they were not present in gene prediction datasets (that are available for some species and that we searched with PSI-BLAST) or because the scores of short exon hits were too low to be detected with TBLASTN.

Dynactin3 has been duplicated in *Rattus norvegicus*. The translations of both genes are identical except for three amino acids that are conserved substitutions. However, the gene of homolog B does not contain any introns and is not supported by EST data. Therefore, it is most likely the result of a recent retro-transcription of a processed pseudogene. Human dynactin3 is encoded on chromosome 9 in 7 exons, which are constitutively spliced (Figure 3B). A few EST clones suggest the alternative transcription of exon 6 that, however, leads to pseudo-transcripts (Figure 3B, Additional file 4). Alternatively spliced isoforms have also not been identified in any other species.

### Dynactin4

Dynactin4 was found in all branches of the eukaryotic tree that contain dynactin. However, homologs could not be identified in many yeast and most of the Schizosaccharomyces species. Dynactin4 proteins are much longer than dynactin3 proteins and we would expect to identify homologs in the yeast and Schizosaccharomyces species based on the supposed homology to the identified dynactin4 proteins. Missing dynactin4 genes are therefore rather the result of gene loss than the result of identification problems.

The published dynactin4 sequence from *Neurospora crassa* (ropy-2 or RO2 gene) contains a sequencing error that led to a predicted N-terminal extension of 173 residues [37]. The genomic sequence encodes another methionine 62 residues upstream of the translation start site. Homologous sequence to these 62 residues including the methionine could only be found in *Neurospora* species and the closely related *Sordaria macrospora* but not in other Sordariales (e.g. *Chaetomium*, *Thielavia*) or any other fungi. The sequence starting from the second methionine is highly conserved in all fungi and thus this methionine is most probably also the translation start site in *Neurospora* and *Sordaria* (Additional file 5).

Dynactin4 from *Homo sapiens* is encoded in 14 exons on chromosome 5 (Figure 3C). Exon 6 (“QHTIHVV”) is a differentially included alternatively spliced exon. Different isoforms have already been reported for rat [68] but not further evaluated. The alternatively spliced exon is conserved in sequence, length, and reading frame in all vertebrates and was also found in the agnath *Petromyzon marinus*, but not in cephalochordates (*Branchiostoma floridae*), tunicates (e.g. *Ciona intestinalis*), and other invertebrates. The exon invention event therefore predates the separation of the Gnathostomata and the Hyperoartia. There is not enough EST/cDNA data available to proof the alternative character of the exon in all vertebrates. For example, there is only one EST clone from *Petromyzon* that covers the respective sequence region and includes exon 6 but none without exon 6. But because there are EST/cDNA clones for several fish, mammals, and *Xenopus* with and without exon 6 it is highly probable that exon 6 is alternatively spliced in all vertebrates. Alternatively spliced isoforms have not been identified in any other species.

Dynactin4 subunits have been predicted to contain N-terminal LIM [68] or RING domains [69], which are short domains consisting of two zinc fingers of the treble clef fold group arranged in tandem [70,71]. The treble clef fold is characterised by a β-hairpin at the N-terminus and an α-helix at the C-terminus that both contribute two ligands for zinc binding [72]. In LIM domains these ligands are almost exclusively cysteins while cysteins could be replaced by histidines in RING domains. In addition, the tandem treble clef fingers are separated by a two-residue spacer in LIM domains, which is invariant in length and seems to be essential for LIM-domain function [73]. Dynactin4 subunits from almost all species contain eight CxxC motifs, of which the seventh and eights motif are separated by about 150 residues. The cysteins are never substituted by histidines and a two-residue spacer exists only between the fifth and sixth motif. A multiple sequence alignment based secondary structure prediction using Jpred [74] did not reveal any α-helical propensity close to the CxxC motifs (data not shown). Thus, dynactin4 can bind up to four zinc ions but it is unlikely that these zinc fingers adopt the treble clef fold and form LIM or RING domains. Rather, the CxxC motifs will form so-called zinc ribbons, which are composed of two β-hairpins forming two structurally similar zinc-binding sub-sites. These sites are often separated by even protein domains [72]. Thus, as long as structural data is not available it is not possible to predict to which of the other motifs the eighth CxxC motif of dynactin4 might fold to build a zinc finger. The highly probable contribution of the eighth motif to the structure of the zinc-finger domain might also explain why expression of only the N-terminal 130 residues of dynactin4 resulted in aggregates [68].

### Dynactin5

Dynactin5 was found in all eukaryotic branches that contain dynactin, except for species of the Schizosaccharomycetes clade and some yeast species of the Saccharomyces clade. In the yeast species *Vanderwaltozyma polyspora*, none of the dynactin subunits were identified, except for dynactin5 and the capping proteins. *Vanderwaltozyma* also does not encode a cytoplasmic dynein homolog. Thus, the *Vp*Dynactin5 might either be an artefact (unlikely) or it gained a species-specific function outside the dynactin complex (needs experimental verification). The absence of dynein and dynactin in *Vanderwaltozyma* is most likely related to the specific phenotypic feature that spores are formed by extra mitotic replications after meiosis independent of bud formation [75]. Sole dynactin5 subunits have also been found in Euglenozoa, and dynactin6 additionally in *Trypanosoma cruzi*. Euglenozoa also contain cytoplasmic dynein heavy and intermediate chains. The presence of only dyanctin5 (and also dynactin6) is in accordance with the report of a freely soluble pool of these subuntis in cells [19]. In addition, a dynactin5 homolog was found for the plant *Vitis vinifera* in the cDNA database. This sequence could not be identified in the genome assembly and grouped to a cluster containing parasitic Nematodes in the phylogenetic tree (data not shown) indicating that it is most likely a contamination of the *Vitis vinifera* cDNA library. Some mammals contain one or more pseudogenes resulting from retro-transcripts.

Dynactin5 from *Homo sapiens* is encoded in six exons on chromosome 16 (Figure 3D). The available EST and cDNA data do not provide evidence for any alternatively spliced exons in human dynactin5 as well as dynactin5’s from any species.

**Figure 4:**
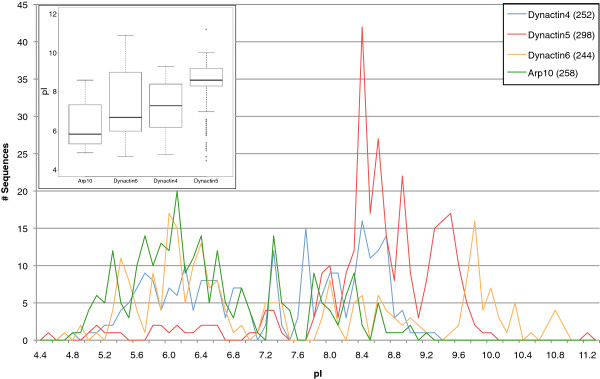
**Distribution of the pI values of the dynactin4, dynactin5, dynactin6, and Arp10 subunits.** The isoelectric points of the dynactin subunits were rounded to the first decimal place and the number of sequences having a pI of a certain range (increment of 0.1) plotted. The numbers next to the subunits in the legend denote the total numbers of sequences used in the graph. The inlet contains box plots of the data for each dynactin subunit.

It has been reported that the subunits dynactin4, dynactin5, and Arp11 from mouse, *Drosophila*, and *C.elegans* have conserved alkaline pIs [21]. It has been suggested that one or all may interact electrostatically with negatively charged membrane lipids or other acidic cargoes such as lipid droplets or viral nucleocapsids [21]. Recently, dynactin5 from *Neurospora crassa* was shown to be required for early endosome interaction [76]. However, *Drosophila* Arp11 and dynactin4 and Arp11 from *C. elegans* actually have acidic pIs (5.16, 6.61, and 6.4, respectively). These contradictions were perplexing and we decided to determine whether potential electrostatic interactions between dynactin subunits and membranes are conserved across the eukaryotes. Therefore, we have analysed the distribution of pI values of the pointed-end complex subunits of all species (Figure 4). Dynactin4, dynactin6, and Arp10/Arp11 show broad distributions from acidic to alkaline pIs. In contrast, almost all dynactin5 subunits have alkaline pIs suggesting a dynactin5 specific interaction within the complex or to other cellular components. The other pointed-end subunits might have conserved functions that are, however, most likely independent from electrostatic interactions.

### Dynactin6

Dynactin6 is encoded in all eukaryotic branches, except for Aconoidasida (including *Plasmodium species*), plants, Rhizaria, Bacillariophyta, Saccharomycotina and Schizosaccharomycetes. In the *Euteleostei* branch (containing part of the fish), the dynactin6 gene has been duplicated. The dynactin6 gene from *Homo sapiens* is located on chromosome 8 (Figure 3E). It consists of 7 exons all of which are constitutively spliced. Alternatively spliced isoforms have not been identified in any species.

### Arp1

The only species encoding dynactin subunits except Arp1 were the yeast *Vanderwaltozyma polyspora DSM 70294*, the stramenopiles *Aureococcus anophageferens*, and the cryptophyte *Guillardia theta*. Duplicates have been identified in mammals and anole lizard (Additional file 6) grouping to two types, variant A (also known as α-centractin, [77,78]) and variant B (also known as β-centractin, [79]). Because the fish Arp1s are most closely related to variant B while the bird and frog Arp1s are most closely related to variant A, the Arp1 duplication event must have been at the origin of the vertebrates, most probably as part of the two whole genome duplications (WGDs) that happened at the emergence of the vertebrates [80]. Unfortunately, EST or genomic DNA data is not available for any Arp1 in *Petromyzon marinus*. Therefore, we cannot conclude yet whether the Arp1 gene duplication happened at the basis of the vertebrates or the Gnathostomata. Subsequent to the duplication, the ancestors of the fish, birds, and frogs each lost one additional Arp1 paralog, while the mammals and the anole lizard retained both of them. Arp1A and Arp1B have both been shown to be part of dynactin and were found in a constant ratio of about 15:1 in the cytosolic fraction [79]. There was no evidence for a free pool of either isoform and it could not be resolved whether Arp1A and Arp1B appear in distinct or mixed complexes. A recent proteomics study of microtubule associated genes in brain tissue also showed that both paralogs are part of the dynactin pool [81]. Formally it could be possible that all combinations of the two Arp1 paralogs are present in dynactin complexes in mammalian cells. However, because the two paralogs are 90% identical (96% similar) and the few differences are distributed over the length of the Arp1 molecule and because most vertebrates retained only one Arp1 homolog it is likely that even mixed dynactin complexes are functionally identical.

### Arp10 (Arp11)

Ten actin-related proteins have been found in the completed genome sequence of *Saccharomyces cerevisiae*, namely Arp1 to Arp10 [82]. Subsequently, next to Arp1 a second actin-related protein has been identified in the vertebrate dynactin complex. It has been named Arp11 although its closest grouping homologs in a phylogenetic tree of actin and actin-related proteins were the yeast Arp10 and ropy-7 from *Neurospora crassa*[21]. Most probably, the support for a potential subfamily grouping was not as significant as for other groups of actin-related proteins. Along the same lines a comparative analysis of 20 completely sequenced eukaryotic genomes did not reveal compelling evidence for grouping Arp10 and Arp11 into one subfamily but recognized that, until then, the appearance of Arp10 and Arp11 was mutually exclusive [83]. It has been suggested that both should be grouped together if yeast Arp10 was found in the dynactin complex or to separate them if both Arp10 and Arp11 were found in a single organism [83]. Recently, yeast Arp10 has been shown to be an integral part of the dynactin complex [41].

Arp10 and Arp11 proteins are very divergent, not only in comparison to the other actin-related proteins but also in between the subfamily. In order to determine their presence or absence in species not encoding unambiguous orthologs we assembled all actin related genes of these species for comparison with complete Arp repertoires of representative organisms. Altogether more than 2,300 Arp proteins have been assembled and analysed including all previously designated Arp classes [83]. Thus, Arp11 orthologs have been identified in the Metazoa, the Fungi (except yeasts), the Amoebozoa, and Oomycetes branch. Arp10 orthologs have been identified in almost all species of the Saccharomycotina branch. Exceptions are *Zygosaccharomyces rouxii*, *Vanderwaltozyma polyspora*, *Candida glabrata*, and *Lodderomyces elongisporus*. Both Arp10 and Arp11 have been found in a mutually exclusive manner and group together in the phylogenetic tree of all Arp proteins (Additional file 1). Therefore, and because representatives of both have been shown to be present in dynactin, both are orthologs. According to HUGO this group should be named Arp10 (symbol ACTR10) and Arp11 can be used as synonym [20]. As with the other dynactin genes we will follow the HUGO recommendation and use the name Arp10 for orthologs of this group of actin-related proteins throughout the rest of the analysis. Arp10 has been duplicated in *Gallus gallus*, and two Arp10 homologs were identified in the pseudotetraploid *Xenopus laevis*. In the other branches of the eukaryotes, none of the assembled actin-related proteins clearly belongs to the Arp10 subfamily.

### Capping proteins Capα (Cap1) and Capβ (Cap2)

The ubiquitous actin capping proteins Capα (Cap1) and Capβ (Cap2) are part of the dynactin complex but except for capping the Arp1 minifilament they do not seem to have dynactin-specific functions and will therefore be discussed in Additional file 7.

## Discussion

**Figure 5:**
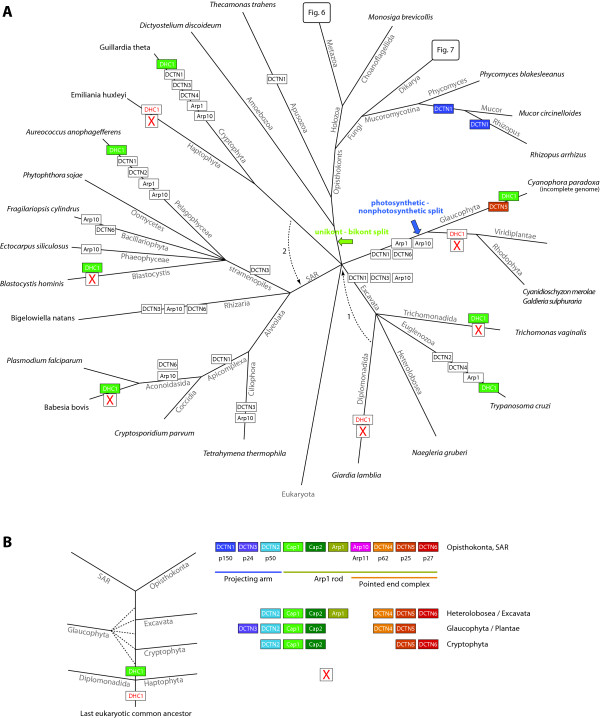
**Evolution of the dynactin complex with respect to the species evolution. A)**. The tree represents the most widely accepted phylogenetic tree of the eukaryotes. However, especially the grouping of taxa that emerged close to the origin of the eukaryotes remains highly debated. Therefore, alternative branchings are also indicated in the tree. The phylogeny of the supposed supergroup Excavata is the least understood because only a few species of this branch have been completely sequenced so far. While the grouping of the Heterolobosea, Trichomonada, and Euglenozoa into the Excavata is found in most analyses, the grouping of the Diplomonadida as separate phylum or as part of the Excavata is still debated (arrow 1 [84]). According to most of the recent phylogenetic analyses, the Alveolata, Rhizaria, and Stramenopiles form the superfamily SAR [85,86]. The placement of the Haptophyceae and Cryptophyta to the SAR is still highly debated. Although several analyses are in favour of this grouping (arrow 2; [87-89]) most analyses are in contrast [85,86,90-92]. At each leaf of the tree one representative species of the branch is printed. Branch lengths are arbitrary. The tree illustrates the presence and absence of each subunit of the dynactin complex in the corresponding species under the hypothesis of five eukaryotic supergroups and the position of the LECA as indicated. Alternative eukaryotic roots are indicated by coloured arrows. Coloured boxes show gene duplications and white boxes show gene loss events of dynactin subunits. The presence (green box) or absence (white box) of the cytoplasmic dynein heavy chain gene DHC1 is indicated for those species that do not have dynactin or miss most of the subunits. **B)** A possible tree of some major branches of the eukaryotes is shown together with the subunits encoded by the respective taxa. The tree is based on the most parsimonious way the branches could have diverged based on the assumption that during this evolution subunits have only been gained and not lost.

Here, we have performed an exhaustive analysis of all known dynactin subunits in all eukaryotic genomes available until September 2011. The presence of dynactin subunits is always coupled to the presence of a cytoplasmic dynein heavy chain (DHC1). Some branches do not contain a DHC1 and accordingly do not contain any dynactin subunit (Figure 5A): plants, diplomonads (e.g. *Giardia lamblia*), Haptophyceae (e.g. *Emiliania huxleyi*), Entamoebidae, some of the Microsporidia, and Rhodophyta (e.g. *Cyanidioschyzon merolae* and *Galdieria sulphuraria*). While the presence of dynactin is coupled to the presence of a DHC1 there are a few species that contain cytoplasmic dyneins but do not encode dynactin subunits: Piroplasmida (e.g. *Babesia* and *Theileria* species), some Microsporidia, and Parabasalia (e.g. *Trichomonas vaginalis*). The DHC1s of the known Piroplasmida and Microsporidia are, however, extremely divergent and shortened (about 3,200 instead of the usual 4,500 residues) and it is not known whether these are functional motors at all. Together, these results demonstrate the strong functional interconnection between dynactin and cytoplasmic dynein. In addition, both were most probably already present in the last common ancestor of the eukaryotes. Although dynein-independent functions have been reported for dynactin these are most likely sub-functionalisations in specific branches of the eukaryotic tree in which either dyneins partnership became obsolete for certain functions or in which dynactin acquired additional specific binding partners.

All of dynactins known eleven subunits were already present in the last common ancestor of the eukaryotes because all of them have been identified in at least two of the major lineages (Figure 5A). However, in many genomes single subunits are missing. Is this due to gene loss events or due to problems in their identification? The dynactin complex and the dynactin subunits have first been identified and characterised in vertebrates and insects, and these constitute the reference sequences. It could be possible, that some subunits have not been identified in several branches and species, which diverged very early in eukaryotic evolution, because of their low similarity to the subunits of the metazoan species that prevented their identification. However, unambiguous homologs have been identified and annotated in every major lineage of the eukaryotes demonstrating that the sequence similarity in general dates back to the last common ancestor. Even when we searched with these homologs instead of the reference sequences, missing homologs in closely related species could not be identified. For example, although a dynactin1 has been found in *Tetrahymena* we were not able to identify dynactin1 homologs in *Toxoplasma gondii*, *Plasmodium* and *Cryptosporidium* species. Therefore, we rather assume that subunits have been lost during evolution although we cannot exclude that we might have missed divergent homologs that can only be revealed in experiments, but not sequence based analyses. In addition, subunits might be missing because of gaps in the sequence assemblies.

### Evolution of the dynactin complex in eukaryotes

The evolution of the dynactin complex in eukaryotes is characterised by many branch- and species-specific gene loss and gene duplication events (Figure 5A). The monophyly of the SAR branch is well established now [93] as well as the monophyly of the Opisthokonts (and even unikonts, [94]) and Excavata [94]. The last common ancestors of both the SAR and the unikonts contained all eleven dynactin subunits (Figure 5B). If the unikont-bikont hypothesis, that combines all major kingdoms except the unikonts into a supergroup called bikonts and places the eukaryotic root between these two supergroups [93,95], were true the last common ancestor of all extant eukaryotes (LECA) must have contained the complete dynactin complex (Figure 5A). Another hypothesis places the origin of the eukaryotes between Plantae and the rest (photosynthetic-nonphotosynthetic split, [96,97]). Unfortunately, the genome sequence of the Glaucophyte *Cyanophora paradoxa* is not complete, but it seems that based on the latter hypothesis the LECA would have contained only dynactin2, dynactin3, dynactin4, dynactin5, and the CAP proteins. The dynactin data does not help in resolving the issue of unambiguously placing the eukaryotic root because its analysis involves eleven subunits and is biased by the very small number of sequenced species in the taxa Cryptophyta, Haptophyta, Glaucophyta and Excavata except Euglenozoa. Thus it could be possible that more complete dynactin inventories will be found in newly sequenced species of these taxa like in the SAR branch in which all dynactin subunits were found in total but not in a single species. Building a parsimonious tree from the presence and absence of the dynactin subunits alone in all species is not possible without breaking established monophyletic groups like the sistergroups Fungi and Holozoa, or the sistergroups Blastocystis and Oomycetes. However, if we try to reconstruct a tree of the eukaryotes based on the major taxa by only breaking the still debated phylogenetic groupings of the Haptophyta and the Diplomonadida but leaving established supergroups intact, the following scenario can be imagined (Figure 5B). Diplomonadida and Haptophyta both do not contain cytoplasmic dynein and dynactin and were therefore the first to diverge in eukaryotic evolution. The LECA would have not contained dynein and dynactin in this case. Next, the dynactin5 and dynactin6 subcomplex and dynactin2 were invented and the Cryptophyta separated. This would be consistent with the finding of a freely soluble pool of dynactin5 and dynactin6 in cells [19]. The placing of the Glaucophyta (as part of the Plantae) is not yet clear due to the incomplete genome of the single representative *Cyanophora paradoxa*. The Glaucophyta do have already dynactin3 and dynactin4 but miss dynactin6. Subsequently, Arp1 and dynactin4 evolved completing the Arp1 rod in Heterolobosea (Excavata). Finally, the projecting arm had been completed in SAR and Opisthokonta. However, this model is based on the assumption that dynactin subunits had only been gained and not lost during early eukaryotic evolution, and the model contradicts the unikont-bikont and the photosynthetic-nonphotosynthetic split hypotheses. Given the many dynactin gene loss events in later separating branches it is more likely that the LECA already contained all dynactin subunits. This assumption could be combined with both split hypotheses and is in agreement with analyses of other protein complexes in which the reconstructed complexes of the LECA contained most of the present-day subunits [98,99].

From the stage of the SAR and Opisthokonta the subsequent evolution of the branches is determined by many and specific gene loss events. Especially Arp10, dynactin6, dynactin1, and dynactin3 have been lost independently in many branches. The Arp1 filament capping function of Arp10 might have been taken over by one of the so far unclassified actin-related proteins or dynactin4. Dynactin6 forms a tight complex with dynactin5 in vertebrates [21] but because also yeasts have, if at all, only dynactin5 it might be possible that dynactin5 forms a homodimer in the species lacking dynactin6. Dynactin1 subunits have independently been lost by many species or their dynactin1 homologs have lost the CAP-Gly domain hindering their identification because of the low sequence similarity of the coiled-coil regions. Vertebrate and *Drosophila* dynactin1 transcripts without a CAP-Gly domain (corresponding to “p135”) are very well versed to bridge the Arp1 rod to dynein and microtubules showing similar intracellular trafficking of organelles [15,58,100]. Thus, so far unknown dynactin1s without CAP-Gly domains could still be present in Apicomplexa, Heterolobosea and Apusozoa. Dynactin3 is necessary for the incorporation of dynactin1 into the yeast dynactin complex [40]. However, it has been shown that dynactin1 in vertebrates and *Drosophila* contains an independent Arp1 binding site, and therefore dynactin3 might not be essential for the dynactin complex in all species. This might explain dynactin3’s absence in many branches that have dynactin1. Other reasons could be that we were not able to identify all dynactin3 subunits because of their low sequence conservation or that dynactin3 has diverged in independent branches so far that homology cannot be detected any more. In any case, strong changes happened to this subunit independently in many early branching eukaryotic lineages and also in closely related branches.

### Expansion of dynactin complexity in metazoa

**Figure 6:**
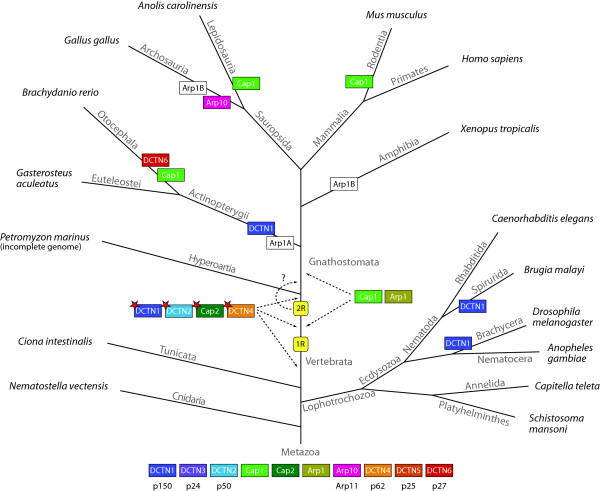
**Evolution of the dynactin complex with respect to the species evolution in Metazoa.** The tree represents the most widely accepted phylogenetic tree of the Metazoa. At each leaf one representative species of the branch is printed. Branch lengths are arbitrary. Coloured boxes show gene duplications of dynactin subunits. Coloured boxes with a red asterisk illustrate the introduction of alternatively spliced isoforms of the corresponding protein in vertebrates. White boxes denote gene loss events. The two whole genome duplication events at the origin of the vertebrates are shown (1R and 2R). The second duplication might also have happened in the Gnathostomata branch (dashed arrow). Due to missing data the duplication of the Cap1 and Arp1 genes cannot unambiguously be dated and could have happened either after the 1R event or after the divergence of the Hyperoartia.

Dynactin complex diversity in metazoa is greatly enhanced by branch specific gene duplications and the introduction of alternative splice forms (Figure 6). The dynactin1 gene has been duplicated independently in the nematods of the Spirurida branch, in the Brachycera including the *Drosophila* species [48], and in fish genomes (Figure 6). Thus, dynactin complexes with different properties could be generated in these species by assembling two different homodimers or a heterodimer of their different dynactin1 subunits. Dynactin6 and Arp10 have also been duplicated in the Otocephala branch (including *Brachydanio rerio*) and in birds, respectively. Arp1 has been duplicated early in vertebrate history and subsequently fish, birds, and amphibians lost different types of the duplicates. An alternative but less likely scenario would be that all dynactin subunit duplications were part of the two whole genome duplications at the origin of the vertebrates followed by numerous independent gene losses in the extant species.

Interestingly, alternatively spliced exons have been invented in vertebrate dynactin1, dynactin2, and dynactin4 genes either before, in between or after the two whole genome duplication events happened but before the divergence of the agnaths and the Gnathostomata (Figure 6). Thus, complexity and fine-tuning of dynactin1 can considerably be enhanced by differential inclusion of four exons that can be combined with three alternative start sites for translation (Figure 2). The alternative start sites affect the inclusion/exclusion of the CAP-Gly domain, and three of the alternative exons encode consecutive pieces of the basic region between the CAP-Gly domain and the first coiled-coil domain. These alternative splice forms therefore do not affect the binding of dynactin to cytoplasmic dynein but only the region attaching dynactin to microtubules [101]. Altogether, 36 different transcripts can theoretically be generated for each vertebrate dynactin1 gene. It is not known yet whether heterodimers of dynactin1 isoforms are possible, which would multiply the theoretically possible number of different dynactin complexes. However, it seems unlikely that a transcript with certain functionality, e.g. a transcript without the CAP-Gly domain, would be combined with a subunit that could in part reconstitute the missing function. This conclusion is consistent with findings in rat brain that showed distinct complexes of dynactin with either full-length dynactin1 (p150) or CAP-Gly diminished dynactin1 (p135, [100]). The three alternative splice forms analysed so far (p150-Δ5; p150-Δ5,6: p150-Δ5,6,7) also showed a tissue specific expression pattern [58] demonstrating that most likely only a limited number of different dynactin1 subunits and not all possible combinations are present in a single cell. However, all combinations will most likely be present in each organism.

Because most subunits are present in multiple copies in the complex, a single gene duplication of one subunit would already result in two, three, or more different complexes, if complexes were built not only from distinct but also mixed subunit compositions. Based on their gene content, the vertebrates can theoretically build thousands of different dynactin complexes considering all combinations of the genes and splice forms. However, most of the differences are introduced by tiny changes. For example, all identified alternatively spliced exons contain only between two and seven residues. In addition, the two Arp1 paralogs differ in only a few residues that are distributed over the length of the molecule. Thus, these small changes are not expected to considerably alter the overall structure of dynactin. However, it is well known that even single posttranslational modifications can dramatically change the functions of proteins from activating/deactivating enzymes or binding/non-binding other proteins or membranes (e.g. phosphorylation of dynactin1 strongly reduces its microtubule affinity, [102]). Concerning the two Arp1 paralogs it is hard to imagine how defined combinations could be generated in the cell given that eight to nine Arp1 subunits comprise the Arp1 minifilament. This would require a strong regulation of the protein level of both paralogs as well as a strong regulation of the position-specific incorporation into the minifilament. The 15:1 ratio of the paralogs could be regulated at the transcription level but it is very likely that both are just randomly incorporated into dynactin without influencing its structure, stability, and function. Therefore, dynactins functions will most likely only be modulated through the various alternative transcripts. The differences seem small but have not been studied at a molecular level yet.

### Reduction of dynactin complexity in yeasts

**Figure 7:**
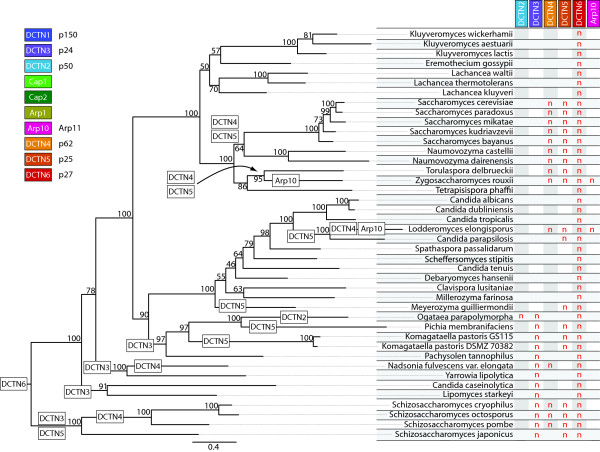
**Evolution of the dynactin complex with respect to the yeast species evolution.** The phylogenetic tree of the Saccharomycetes and Schizosaccharomycetes is based on the Maximum-Likelihood tree (RAxML) of the concatenated dynactin2, Cap1, Cap2, Arp1, and Arp10 subunits. Bootstrap support values (100 randomisations) are given for every node. The phylogenetic distribution of the sampled species is in overall agreement with other recent yeast phylogenies [106,107]. Small differences are most likely due to the different genes (LSU rRNA, SSU rRNA and EF-1α DNA sequences in [106], 542 putative orthologous proteins in [107], and dynactin protein sequences in our analysis) and methods used (NJ and MP in [106], ML in [107] and in our analysis). On the left the phylogenetic tree is shown with the corresponding species at each leaf. White boxes at branches represent gene loss events. On the right those subunits of the dynactin complex are tabulated that show differential inclusion within the analysed species. Dynactin subunits that are present in all species have been omitted for clarity. The abbreviation ‘n’ denotes the absence of the corresponding subunit in the respective genome while blanks indicate their presence.

In general, gene loss in yeasts only affects the pointed-end complex subunits and dynactin3, which mediates association of dynactin1 to dynactin2 (Figure 7) The Schizosaccharomycetes and Saccharomycotina both have lost the dynactin6 subunit. Dynactin5 and dynactin6 are predicted to fold into left-handed β-helical structures [103], and are supposed to form a tight heterodimeric complex in vertebrates [21]. They show low sequence similarity but can still be aligned to each other (data not shown). Therefore, it could be possible that dynactin5 forms homodimers in those species that do not encode dynactin6. Dynactin3 and dynactin5 have been lost in Schizosaccharomycetes and many Saccharomycotina subbranches. The loss of dynactin3 in yeasts is surprising because it has been found to be essential to recruit dynactin1 to the dynactin complex in *Saccharomyces cerevisiae*[40]. The dynactin1 genes in yeasts have about the same lengths, which is also true for the dynactin2 genes. A missing dynactin3 is therefore not compensated by additional domains in the other dynactin subunits. Either changes at the surface of dynactin1 or dynactin2 may supersede dynactin3 or we were not able to detect the missing dynactin3 subunits yet. Dynactin5 is required for the interaction of dynein with a subset but not all membranous vesicles, which is supported by the conserved basic pI of all dynactin5 subunits and by membrane-flotation essays [76]. This is also consistent with findings in *Saccharomyces cerevisiae*, which does not encode a dynactin5 subunit, that dynein is necessary for nuclear migration and spindle orientation but does not perform vesicle transport [104,105]. In addition, dynactin4, Arp10, and dynactin2 have been lost in several, two, and 1 branch of the yeasts, respectively. Arp10 is needed for the stability and capping of the Arp1 filament [41] and its absence should thus affect dynactins integrity. Both *Zygosaccharomyces* and *Lodderomyces* lack Arp10 and dynactin4, the other pointed-end capping protein and it is unclear how the Arp1 filament could be stabilized in these species.

## Conclusions

The dynactin complex is a very ancient complex that already existed in the last common ancestor of extant eukaryotes. It consists of eleven subunits of which at least seven comprise the core structure: dynactin1, dynactin2, dynactin4, dynactin5, the heterodimeric capping protein, and Arp1. The presence of the dynactin complex coincides with that of the cytoplasmic dynein heavy chain: Organisms that do not encode cytoplasmic dyneins like plants and diplomonads also do not encode dynactin subunits either. In the metazoan lineage, several of the dynactin subunits were duplicated independently in different branches. The largest repertoire is found in vertebrates. Also at the origin of the vertebrates, several alternatively spliced exons have been invented providing the basis for modulating the core functions. The most prominent example is the dynactin1 gene, from which 36 different transcripts could be generated. In contrast, ascomycetous yeasts have reduced subunit compositions. In general they have reduced pointed-end complexes, which in return let to the loss of the functions coupled to the specific subunits.

## Methods

### Identification and annotation of the genes of the dynactin subunits

Dynactin genes have been identified in iterated TBLASTN and PSI-BLAST searches of the completed or almost completed genomes of about 600 organisms starting with the protein sequences of the human dynactin subunits. All hits were manually analysed at the genomic DNA level. The correct coding sequences were identified with the help of multiple sequence alignments of the respective dynactin subunits. As the amount of dynactin sequences increased (especially the number of sequences from taxa with few representatives), many of the initially predicted sequences were reanalysed to correctly identify all exon borders. Where possible, EST data has been analysed to help in the annotation process. In addition to the analysis of these large-scale sequencing projects, all dynactin sequences in the “nr” database at NCBI have been collected and reanalysed.

Several of the genes contain alternative splice forms. The different splice forms were not considered independently in the analysis but in all cases the same splice forms were taken for homologous dynactin proteins. All sequence related data (names, corresponding species, GenBank ID's, alternative names, corresponding publications, domain predictions, sequences, and gene structure reconstructions) and references to genome sequencing centers are available through the CyMoBase ( http://www.cymobase.org, [108]). A list of the species analysed, their abbreviations as used in the alignments and trees, as well as detailed information and acknowledgments of the respective sequencing centers is also available as Additional file 8. Webscipio [53,109] was used to reconstruct the gene structure (exon/intron pattern) of each sequence.

### Generating the multiple sequence alignment

The multiple sequence alignments of the dynactin subunits have been built and extended during the process of annotating and assembling new sequences. The initial alignments have been generated from the first about 20 sequences obtained from NCBI using the ClustalW software with standard settings [110]. During the following correction of the sequences (removing wrongly annotated sequences and filling gaps) the alignment has been adjusted manually. Subsequently, every newly predicted sequence has preliminarily been aligned to its supposed closest relative using ClustalW, the aligned sequence added to the multiple sequence alignment of the respective dynactin subunit, and the alignment adjusted manually during the subsequent sequence validation process. Still, many gaps in sequences derived from low-coverage genomes remained. In those cases, the integrity of the exons next to gaps has been maintained (gaps in the genomic sequence are reflected as gaps in the multiple sequence alignment). The sequence alignments of the dynactin subunits can be obtained from CyMoBase or Additional file 1.

### Comparison of the sequence identities and similarities

Sequences designated “Fragment”, “Partial”, or “Pseudogene” were removed from the multiple sequence alignments of the dynactin subunits. Poorly aligned positions and divergent regions of the alignments were removed using Gblocks [111] with the following parameters: A) The minimum number of sequences for a conserved position and the minimum of sequences for a flank position were set to the minimum (e.g. half the number of sequences plus one). B) The maximum number of contiguous nonconserved positions was set to 32000 and the minimum length of a block was set to 2. C) The parameter for the allowed gap position was set to ‘all’.

Sequence identity matrices (2D-matrix tables containing sequence identities scores for each pair of sequences) were calculated for each alignment using the method implemented in BioEdit (Tom Hall, http://www.mbio.ncsu.edu/bioedit/bioedit.html). Shortly, the reported numbers represent the ratio of identities to the length of the longer of the two sequences after positions where both sequences contain a gap are removed. Sequence similarity matrices were calculated with MatGAT [112] using the BLOSUM62 substitution matrix and setting the gap opening and extending penalties to 12 and 2, respectively.

### Computing and visualising phylogenetic trees

For calculating phylogenetic trees of single dynactin subunits only complete and partial sequences were included in the dataset. For the calculation of the tree of the yeast species, the sequences of the Arp1, Arp10, Cap1, Cap2, and dynactin2 subunit of each species of the Saccharomyces and the Schizosaccharomyces branch were concatenated. Missing protein sequences (*Zygosaccharomyces rouxii* Arp10, *Lodderomyces elongisporus* Arp10, *Ogataea parapolymorpha* dynactin2, and *Naumovozyma dairenensis* Cap2) were substituted by gaps. The phylogenetic trees were generated using two different methods for each dataset: 1. ProtTest was used to determine the most appropriate of the available 112 possible amino acid substitution models [113]. The tree topology was calculated with the BioNJ algorithm and both the branch lengths and the model of protein evolution were optimized simultaneously. The Akaike Information Criterion with a modification to control for small sample size (AICc, with alignment length representing sample size) identified the LG model [114] to be the best for the dynactin3, dynactin5, dynactin6, Arp, and Cap datasets and the JTT model [115] for dynactin1, dynactin2, and dynactin4. Maximum likelihood (ML) analysis with estimated proportion of invariable sites and bootstrapping (1,000 replicates) were performed using RAxML [116]. 2. Posterior probabilities were generated using MrBayes v3.1.2 [117] with the MPI option [118]. Two independent runs with 5,000,000 generations, four chains, and a random starting tree were computed using the mixed amino-acid option. MrBayes used the WAG model [119] for all protein alignments. Trees were sampled every 1.000th generation and the first 25% of the trees were discarded as “burn-in” before generating a consensus tree. Phylogenetic trees were visualized with the CLC Sequence Viewer ( http://www.clcbio.com) and FigTree ( http://tree.bio.ed.ac.uk/software/figtree/) and are available as Additional file 1.

## Competing interests

The authors declare that they have no competing interests.

## Authors' contributions

BH performed all database related work, adjusted the CyMoBase software for specific dynactin related needs, did all data analysis and drafted the manuscript. MK assembled and annotated all sequences, and assisted in writing the manuscript. Both authors read and approved the final version of the manuscript.

## Supplementary Material

Additional file 1 **Zip archive of the Maximum Likelihood and Bayesian inference trees, and the sequence alignments of the dynactin subunits.** The file includes all Maximum Likelihood and Bayesian trees of all dynactin proteins in the Newick format. The sequence alignments of the proteins are included in fasta format. Click here for file

Additional file 2 **Dynactin inventory of the analysed species. **The file lists the presence and number of orthologs for each dynactin subunit for each analysed organism in taxonomic order. Click here for file

Additional file 3 **Phylogenetic tree of dynactin1.** The file contains the phylogenetic tree of dynactin1 highlighting the species- and branch-specific gene duplication events. Click here for file

Additional file 4 **Detailed description of the pseudo-transcripts of dynactin4.** The file contains details about the pseudo-transcripts of dynactin4 Click here for file

Additional file 5 **Sequence alignment of fungal dynactin4 proteins.** The file contains the sequence alignment of the N-termini of several fungal dynactin4 (p62) subunits showing that the upstream methionines in *Neurospora* and *Sordaria* are most likely not the translation start sites. Click here for file

Additional file 6 **Phylogenetic tree of Arp1.** The file contains the phylogenetic tree of Arp1 focused on the vertebrate branch highlighting the Arp1 gene duplication event and subsequent branch-specific losses of Arp1 subtypes. Click here for file

Additional file 7 **Discussion of the evolution of the Cap proteins.** The file contains a discussion of the gene duplications, which happened to the Capα (Cap1) subunit, and of alternative splice variants of the Capβ (Cap2) subunit [120,121]. Click here for file

Additional file 8 **Species table.** The file contains all species of the analysis, their scientific names, the abbreviation as used in the sequence alignments and trees, the species taxonomy, references to sequencing centers, and publications if genome analyses have already been published. Click here for file
