# Supplementary material for: Evolution of the eukaryotic dynactin complex, the activator of cytoplasmic dynein
Source: BMC Evol Biol. 2012 Jun 22;12:95. doi: 10.1186/1471-2148-12-95 (PMC3583065; doi:10.1186/1471-2148-12-95)
Supplement: Additional file 1 — Zip archive of the Maximum Likelihood and Bayesian inference trees, and the sequence alignments of the dynactin subunits. The file includes all Maximum Likelihood and Bayesian trees of all dynactin proteins in the Newick format. The sequence alignments of the proteins are included in fasta format. [file 1471-2148-12-95-S1.pdf]

Species	Arp1	Arp10	Dynactin1	Dynactin2	Dynactin3	Dynactin4	Dynactin5	Dynactin6	CAP
Plasmodium gallinaceum	-	-	-	1	-	-	1	-	-
Plasmodium falciparum Ghanaian Isolate	-	-	-	-	-	-	1	-	-
Plasmodium falciparum 3D7	1	-	-	1	1	1	1	-	2
Plasmodium falciparum Dd2	1	-	-	-	-	-	1	-	-
Plasmodium falciparum HB3	1	-	-	-	-	-	1	-	-
Plasmodium knowlesi	1	-	-	1	1	1	1	-	2
Plasmodium vivax str. Salvador I	1	-	-	1	1	1	1	-	2
Plasmodium chabaudi	1	-	-	1	-	1	1	-	2
Plasmodium berghei str. ANKA	1	-	-	1	1	1	1	-	2
Plasmodium yoelii yoelii 17XNL	1	-	-	1	1	1	1	-	2
Cryptosporidium parvum str. Iowa II	1	1	-	1	1	1	1	1	2
Cryptosporidium hominis TU502	1	1	-	1	1	1	1	1	2
Cryptosporidium muris RN66	1	-	-	1	1	1	1	1	2
Eimeria tenella	-	-	-	-	-	-	1	-	-
Neospora caninum	1	-	-	-	1	-	1	-	-
Toxoplasma gondii GT1	1	-	-	1	1	1	1	1	2
Toxoplasma gondii ME49	1	-	-	-	-	-	-	-	2
Toxoplasma gondii VEG	1	-	-	-	-	-	-	-	2
Ichthyophthirius multifiliis strain G5	1	-	1	1	-	1	1	1	2
Tetrahymena thermophila SB210	1	-	1	1	-	1	1	1	2
Paramecium tetraurelia	1	-	1	2	-	1	1	1	4
Entamoeba histolytica HM-1:IMSS	-	1	-	-	-	-	-	-	2
Entamoeba dispar SAW760	-	-	-	-	-	-	-	-	2
Entamoeba invadens IP1	-	-	-	-	-	-	-	-	3
Acanthamoeba castellanii	-	-	1	1	-	1	1	1	3
Dictyostelium fasciculatum	1	1	1	1	1	1	1	1	2
Dictyostelium purpureum	1	1	1	1	1	1	1	1	2
Dictyostelium discoideum AX4	1	1	1	1	1	1	1	1	2
Polysphondylium pallidum	1	1	1	1	1	1	1	1	2
Thecamonas trahens ATCC 50062	1	1	-	1	1	1	1	1	2
Guillardia theta	-	-	-	1	-	-	1	1	-
Leishmania infantum JPCM5	-	-	-	-	-	-	1	-	-
Leishmania major str. Friedlin	-	-	-	-	-	-	1	-	-
Leishmania braziliensis	-	-	-	-	-	-	1	-	-
Trypanosoma cruzi CL Brener	-	-	-	-	-	-	1	1	2
Cyanophora paradoxa	-	-	-	1	1	1	2	-	-
Naegleria gruberi NEG-M	1	-	-	1	-	1	1	1	2
Monosiga ovata	-	-	-	1	-	1	1	-	-
Monosiga brevicollis MX1	1	-	1	1	1	1	1	1	2
Proterospongia sp. ATCC 50818	1	1	1	1	1	1	1	1	2
Allomyces macrogynus ATCC 38327	1	1	1	1	-	1	1	1	2
Blastocladiella emersonii	-	-	-	-	-	-	1	1	-
Batrachochytrium dendrobatidis JAM81	1	1	1	1	-	1	1	1	2
Batrachochytrium dendrobatidis JEL423	1	1	1	1	-	1	1	1	2
Spizellomyces punctatus DAOM BR117	1	1	1	1	-	1	1	1	2
Mycosphaerella fijiensis	1	1	1	1	1	1	1	-	2
Mycosphaerella pini NZE10	1	1	-	-	-	-	-	-	-
Mycosphaerella populorum SO2202	1	1	-	-	-	-	-	-	-
Mycosphaerella graminicola IPO323	1	1	1	1	1	1	1	1	2
Aureobasidium pullulans	-	-	1	-	-	-	-	-	-
Phaeosphaeria nodorum SN15	1	1	1	1	1	1	1	1	2
Cochliobolus heterostrophus C5	1	1	1	1	1	1	1	1	2

Alternaria brassicicola ATCC 96866	1	1	1	1	1	1	1	1	2
Pyrenophora teres f. teres 0-1	1	1	-	-	-	-	-	-	-
Pyrenophora tritici-repentis Pt-1C-BFP	1	1	1	1	1	1	1	1	2
Emericella nidulans FGSC A4	1	1	1	1	1	1	1	1	2
Aspergillus aculeatus ATCC16872	1	1	-	-	-	-	-	-	-
Aspergillus carbonarius ITEM 5010	1	1	1	1	1	1	1	1	2
Aspergillus clavatus NRRL 1	1	1	1	1	1	1	1	1	2
Aspergillus flavus NRRL3357	1	1	1	1	1	1	1	1	2
Aspergillus fumigatus A1163	1	1	1	1	1	1	1	1	2
Aspergillus fumigatus Af293	1	1	1	1	1	1	1	1	2
Aspergillus niger ATCC 1015	1	1	1	1	1	1	1	1	2
Aspergillus niger CBS 513.88	1	1	1	1	1	1	1	1	2
Aspergillus oryzae RIB40	1	1	1	1	1	1	1	1	2
Aspergillus terreus NIH2624	1	1	1	1	1	1	1	1	2
Penicillium chrysogenum Wisconsin 54-1255	1	1	1	1	1	1	1	1	2
Penicillium marneffei ATCC 18224	1	1	1	1	1	1	1	1	2
Neosartorya fischeri NRRL 181	1	1	1	1	1	1	1	1	2
Talaromyces stipitatus ATCC 10500	1	1	1	1	1	1	1	1	2
Ajellomyces capsulatus NAmII G186AR	1	1	1	1	1	1	1	1	2
Ajellomyces capsulatus NAmII G217B	1	1	1	1	1	1	1	1	2
Ajellomyces capsulatus NAmI WU24	1	1	1	1	1	1	1	1	2
Ajellomyces capsulatus H143	1	1	-	-	-	-	-	-	-
Ajellomyces capsulatus H88	1	1	-	-	-	-	-	-	-
Ajellomyces dermatitidis ATCC 18188	1	1	-	-	-	-	-	-	-
Ajellomyces dermatitidis ATCC 26199	1	1	-	-	-	-	-	-	-
Ajellomyces dermatitidis ER-3	1	1	-	-	-	-	-	-	-
Ajellomyces dermatitidis SLH14081	1	1	-	-	-	-	-	-	-
Arthroderma benhamiae CBS 112371	1	1	1	1	1	1	1	1	2
Arthroderma gypseum CBS 118893	1	1	1	1	1	1	1	1	2
Arthroderma otae CBS 113480	1	1	1	1	1	1	1	1	2
Trichophyton equinum CBS 127.97	1	1	1	1	1	1	1	1	2
Trichophyton rubrum CBS 118892	1	1	1	1	1	1	1	1	2
Trichophyton tonsurans CBS 112818	1	1	1	1	1	1	1	1	2
Trichophyton verrucosum HKI 0517	1	1	-	1	-	-	-	-	-
Ascosphaera apis USDA-ARSEF 7405	1	1	1	1	1	1	1	1	2
Coccidioides posadasii C735	1	1	1	1	1	1	1	1	2
Coccidioides immitis H538.4	1	1	1	1	1	1	1	1	2
Coccidioides immitis RMSCC 2394	1	1	1	1	1	1	1	1	2
Coccidioides immitis RMSCC 3703	1	1	1	1	1	1	1	1	2
Coccidioides immitis RS	1	1	1	1	1	1	1	1	2
Coccidioides posadasii CPA 0066	-	-	1	-	-	-	-	-	-
Coccidioides posadasii RMSCC 3488	1	1	1	1	1	1	1	1	2
Coccidioides posadasii str. Silveira	1	1	1	1	1	1	1	1	2
Paracoccidioides brasiliensis Pb01	1	1	1	1	1	1	1	1	2
Paracoccidioides brasiliensis Pb03	1	1	1	1	1	1	1	1	2
Paracoccidioides brasiliensis Pb18	1	1	1	1	1	1	1	1	2
Uncinocarpus reesii 1704	1	1	1	1	1	1	1	1	2
Blumeria graminis f. sp. hordei DH14	1	1	1	1	1	1	1	1	2
Botryotinia fuckeliana B05.10	1	1	1	1	1	1	1	1	2
Sclerotinia sclerotiorum 1980	1	1	1	1	1	1	1	1	2
Geomyces pannorum	-	-	-	-	-	-	1	1	-
Geomyces destructans 20631-21	1	1	-	-	-	-	-	-	-
Glomerella graminicola	1	1	-	-	-	-	-	-	-
Verticillium albo-atrum VaMs.102	1	1	1	1	1	1	1	1	2

Verticillium dahliae VdLs.17	1	1	1	1	1	1	1	1	2
Epichloe festucae E2368	1	1	1	1	1	1	1	1	2
Metarhizium anisopliae ARSEF 23	-	-	-	1	-	-	-	-	-
Cordyceps bassiana	-	-	-	-	-	-	-	1	-
Hypocrea virens Gv29-8	1	1	1	1	1	1	1	1	2
Hypocrea jecorina QM6a	1	1	1	1	1	1	1	1	2
Trichoderma asperellum CECT 20268	-	-	-	1	-	-	-	-	-
Trichoderma atroviride IMI 206040	1	1	1	1	1	1	1	1	2
Fusarium sporotrichioides	-	-	1	-	-	-	-	-	-
Fusarium oxysporum f. sp. lycopersici 4287	1	1	1	1	1	1	1	1	2
Gibberella moniliformis 7600	1	1	1	1	1	1	1	1	2
Gibberella zeae PH-1	1	1	1	1	1	1	1	1	2
Nectria haematococca MPVI	1	1	1	1	1	1	1	1	2
Cryphonectria parasitica	1	1	1	1	1	1	1	1	2
Gaeumannomyces graminis var. tritici R3-111a-1	1	1	-	-	-	-	-	-	-
Magnaporthe grisea 70-15	1	1	1	1	1	1	1	1	2
Magnaporthe poae ATCC 64411	1	1	-	-	-	-	-	-	-
Grosmannia clavigera kw1407	1	1	-	-	-	-	-	1	-
Chaetomium globosum CBS 148.51	1	1	1	1	1	1	1	1	2
Thielavia heterothallica ATCC 42464	1	1	1	1	1	1	1	1	2
Thielavia terrestris NRRL 8126	1	1	1	1	1	1	1	1	2
Podospora anserina	1	1	1	1	1	1	1	1	2
Neurospora crassa OR74A	1	1	1	1	1	1	1	1	2
Neurospora discreta FGSC 8579	1	1	1	1	1	1	1	1	2
Neurospora tetrasperma FGSC 2508	1	1	1	1	1	1	1	1	2
Sordaria macrospora	1	1	-	-	-	1	-	-	-
Tuber melanosporum Mel28	1	1	1	1	1	1	1	1	2
Debaryomyces hansenii CBS767	1	1	1	1	1	1	1	-	2
Lodderomyces elongisporus NRRL YB-4239	1	-	1	1	1	-	-	-	2
Meyerozyma guilliermondii ATCC 6260	1	1	1	1	1	1	-	-	2
Millerozyma farinosa CBS7064	2	2	2	2	2	2	2	-	4
Scheffersomyces stipitis CBS 6054	1	1	1	1	1	1	1	-	2
Spathaspora passalidarum NRRL Y-27907	1	1	1	1	1	1	1	-	2
Yarrowia lipolytica CLIB122	1	1	1	1	-	1	1	-	2
Lipomyces starkeyi NRRL Y-11557	1	1	1	1	-	1	1	-	2
Clavispora lusitaniae ATCC 42720	1	1	1	1	1	1	1	-	2
Candida parapsilosis	1	1	1	1	1	1	-	-	2
Candida tenuis NRRL Y-1498	1	1	1	1	1	1	1	-	2
Candida albicans SC5314	2	1	2	1	1	1	1	-	4
Candida albicans WO-1	1	-	1	1	1	1	1	-	2
Candida caseinolytica NRRL Y-17796	1	1	1	1	-	1	1	-	2
Candida dubliniensis CD36	1	1	1	1	1	1	1	-	2
Candida tropicalis MYA-3404	1	1	1	1	1	1	1	-	2
Wickerhamomyces anomalus NRRL Y-366	2	-	1	1	-	1	1	-	2
Pichia membranifaciens	1	1	1	1	-	1	-	-	2
Eremothecium gossypii ATCC 10895	1	1	1	1	1	1	1	-	2
Kluyveromyces aestuarii ATCC 18862	1	1	1	1	1	1	1	-	2
Kluyveromyces lactis NRRL Y-1140	1	1	1	1	1	1	1	-	2
Kluyveromyces wickerhamii UCD 54-210	1	1	1	1	1	1	1	-	2
Komagataella pastoris DSMZ 70382	1	1	1	1	-	1	-	-	2
Komagataella pastoris GS115	1	1	1	1	-	1	-	-	2
Lachancea kluyveri CBS 3082	1	1	-	-	-	-	-	-	-
Lachancea kluyveri NRRL Y-12651	1	1	1	1	1	1	1	-	2
Lachancea thermotolerans CBS 6340	1	1	1	1	1	1	1	-	2

Lachancea waltii NCYC 2644	1	1	1	1	1	1	1	-	2
Candida glabrata CBS138	1	-	1	1	-	-	-	-	2
Naumovozyma castellii NRRL Y-12630	1	1	-	1	1	1	-	-	2
Naumovozyma dairenensis CBS 421	1	1	1	1	1	-	-	-	2
Pachysolen tannophilus NRRL Y-2460	1	1	1	1	-	1	1	-	2
Saccharomyces bayanus var. uvarum	1	-	-	-	-	-	-	-	-
Saccharomyces bayanus 623-6C	1	1	1	1	1	-	-	-	2
Saccharomyces bayanus MCYC 623	1	1	1	1	-	-	-	-	2
Saccharomyces cerevisiae M22	-	-	-	-	-	-	-	-	1
Saccharomyces cerevisiae RM11-1a	1	1	1	1	1	-	-	-	2
Saccharomyces cerevisiae S288c	1	1	1	1	1	-	-	-	2
Saccharomyces cerevisiae YJM789	1	1	1	1	1	-	-	-	2
Saccharomyces cerevisiae YPS163	-	-	-	-	-	-	-	-	1
Saccharomyces kudriavzevii IFO 1802	1	1	1	1	1	-	-	-	2
Saccharomyces mikatae IFO 1815	1	1	1	1	1	-	-	-	2
Saccharomyces paradoxus NRRL Y-17217	1	1	1	1	1	-	-	-	2
Saccharomyces pastorianus Weihenstephan 34/70	2	2	-	-	-	-	-	-	-
Tetrapisispora phaffii CBS 4417	1	1	1	1	1	1	1	-	2
Torulaspora delbrueckii CBS 1146	1	1	1	1	1	-	-	-	2
Vanderwaltozyma polyspora DSM 70294	-	-	-	-	-	-	1	-	2
Zygosaccharomyces rouxii CBS732	1	-	1	1	1	-	-	-	2
Ogataea angusta NCYC 495 leu1.1	1	1	1	-	-	1	1	-	2
Ogataea parapolymorpha DL-1	1	1	1	-	-	1	1	-	2
Ogataea polymorpha CBS 4732	-	1	-	-	-	-	-	-	-
Nadsonia fulvescens var. elongata DSM 6958	1	1	1	1	-	-	1	-	2
Pneumocystis carinii	1	-	-	-	-	-	-	-	-
Schizosaccharomyces cryophilus NRRL Y-48691	1	1	1	1	-	-	-	-	2
Schizosaccharomyces japonicus yFS275	1	1	1	1	-	1	-	-	2
Schizosaccharomyces octosporus yFS286	1	1	1	1	-	-	-	-	2
Schizosaccharomyces pombe 972h-	1	1	1	1	-	-	-	-	2
Phanerochaete chrysosporium RP-78	1	1	1	1	1	1	1	1	2
Postia placenta	1	1	1	1	1	1	1	1	2
Antrodia cinnamomea	-	-	-	-	-	-	-	1	-
Heterobasidion annosum	1	1	1	1	1	1	1	1	2
Lentinula edodes	-	-	-	1	-	-	-	-	-
Coprinopsis cinerea okayama7#130	1	1	1	1	1	1	1	1	2
Schizophyllum commune H4-8	1	1	1	1	1	1	1	1	2
Laccaria bicolor S238N	1	1	1	1	1	1	1	1	2
Pisolithus microcarpus	-	-	-	-	-	-	-	1	-
Cryptococcus laurentii	-	-	-	-	-	-	-	1	-
Filobasidiella neoformans var. bacillispora R265	1	1	1	1	1	1	1	1	2
Filobasidiella neoformans var. bacillispora WM276	1	1	-	-	-	-	-	-	-
Filobasidiella neoformans var. neoformans B-3501A	1	1	1	1	1	1	1	1	2
Filobasidiella neoformans var. neoformans H99	1	1	1	1	1	1	1	1	2
Filobasidiella neoformans var. neoformans JEC21	1	1	1	1	1	1	1	1	2
Tremella mesenterica	1	-	-	-	-	-	-	-	-
Leucosporidium scottii	-	-	-	-	1	-	-	-	-
Sporobolomyces roseus IAM 13481	1	1	1	1	1	1	1	1	2
Melampsora laricis-populina	1	1	1	1	1	1	1	1	2
Phakopsora pachyrhizi	-	-	-	-	1	-	-	1	-
Puccinia graminis f. sp. tritici CRL 75-36-700-3	1	1	-	1	1	1	1	1	1
Puccinia triticina 1-1 BBBD Race 1	2	1	-	-	-	-	-	-	-
Malassezia globosa CBS 7966	1	1	1	1	1	1	1	1	2

Malassezia restricta CBS 7877	1	1	1	1	1	1	1	1	2
Sporisorium reilianum	-	-	-	-	-	1	-	-	-
Ustilago maydis 521	1	1	1	1	-	1	1	1	2
Ustilago maydis FB1	-	-	1	1	-	1	1	1	-
Cunninghamella elegans	-	-	1	-	1	1	-	-	-
Mucor circinelloides	1	1	2	1	1	1	1	1	2
Phycomyces blakesleeanus	1	1	1	1	1	1	1	1	2
Rhizopus arrhizus RA 99-880	1	1	3	1	1	1	1	1	2
Glomus intraradices	-	-	-	-	-	-	-	1	-
Echinococcus granulosus	-	-	-	-	1	-	1	1	-
Echinococcus multilocularis	-	-	-	-	1	-	-	1	-
Taenia solium	-	-	-	1	1	1	1	1	-
Schistosoma japonicum	1	1	1	1	1	1	1	1	2
Schistosoma mansoni	1	1	1	1	1	1	1	1	2
Convoluta pulchra	-	-	1	-	-	-	-	-	-
Dugesia japonica	-	-	-	-	-	-	-	1	-
Schmidtea mediterranea str. S2F2	-	-	-	-	1	-	1	1	1
Branchiostoma floridae	1	1	1	1	1	1	1	1	2
Eptatretus burgeri	-	-	-	1	-	1	-	-	-
Leucoraja erinacea	-	-	-	1	-	1	1	-	-
Squalus acanthias	-	-	1	1	-	-	-	-	-
Callorhinchus milii	-	1	-	-	1	1	1	-	-
Haplochromis burtoni	-	-	-	-	-	1	1	1	-
Labeotropheus fuelleborni str. Domwe Island	-	-	1	-	-	-	-	-	-
Lipochromis sp. 'matumbi hunter'	-	-	-	-	1	1	1	-	-
Maylandia zebra str. Mazinzi Reef	-	-	1	-	-	-	-	-	-
Melanochromis auratus str. Domwe Island	-	-	1	-	-	-	-	-	-
Paralabidochromis chilotes	-	-	1	-	-	-	1	-	-
Ptyochromis sp. 'redtail sheller'	-	-	-	-	-	-	1	-	-
Oreochromis niloticus	1	1	2	1	1	1	1	2	4
Lithognathus mormyrus	-	-	-	-	-	-	-	1	-
Sparus aurata	-	-	2	-	-	-	-	-	-
Thunnus thynnus	-	-	-	-	-	-	-	1	-
Hippoglossus hippoglossus	-	-	2	-	-	-	-	-	2
Anoplopoma fimbria	-	-	-	-	-	-	-	-	2
Oryzias latipes str. Hd-rR	1	1	2	1	1	1	1	2	4
Oryzias latipes str. HNI	-	-	2	-	-	-	1	-	1
Fundulus heteroclitus	-	-	-	-	1	1	-	-	-
Poecilia reticulata	-	-	-	-	-	1	-	-	-
Xiphophorus maculatus x Xiphophorus helleri	-	-	1	-	-	-	-	-	-
Gasterosteus aculeatus	1	1	2	1	1	1	1	2	4
Takifugu rubripes	1	1	2	1	1	1	1	2	4
Tetraodon nigroviridis	1	1	2	1	1	1	1	2	3
Gadus morhua	-	-	-	-	-	-	1	1	-
Osmerus mordax	-	-	1	1	1	-	1	2	1
Oncorhynchus mykiss	-	-	2	1	1	2	-	2	-
Oncorhynchus nerka	-	-	-	1	-	-	-	-	-
Salmo salar	1	-	2	1	1	1	1	2	4
Salvelinus fontinalis	-	-	-	-	1	-	-	-	-
Cyprinus carpio	-	-	1	-	-	-	-	1	-
Brachydanio rerio str. Tuebingen	1	1	2	1	1	1	1	1	3
Gobiocypris rarus	-	-	-	-	1	-	-	-	-
Pimephales promelas	-	-	1	1	1	1	1	1	-
Rutilus rutilus	-	-	-	-	-	-	1	-	-

Ictalurus punctatus	-	-	-	-	1	-	-	1	-
Latimeria chalumnae	-	-	1	1	1	1	1	1	-
Ornithorhynchus anatinus	1	1	1	-	1	1	1	1	2
Loxodonta africana	-	1	1	2	1	1	1	-	-
Echinops telfairi	-	1	1	1	1	1	1	1	-
Oryctolagus cuniculus	-	1	1	1	1	1	1	-	-
Cavia porcellus	-	-	-	-	-	-	1	-	-
Cavia porcellus str. inbred	2	1	1	1	1	1	1	1	4
Mus musculus C57BL/6J	2	1	1	1	1	1	3	1	8
Mus musculus str. mixed	2	1	1	1	1	1	3	1	6
Rattus norvegicus BN/Sprague-Dawley	2	1	1	1	2	1	1	1	5
Rattus norvegicus BN/SsNHsdMCW	2	1	1	1	2	2	3	2	5
Spermophilus tridecemlineatus	-	1	1	1	1	1	1	1	-
Macaca fascicularis	1	-	1	-	1	-	1	-	4
Macaca mulatta Indian origin	2	1	1	1	1	1	1	1	6
Macaca nemestrina	-	-	-	-	1	-	-	-	-
Papio anubis	-	-	-	-	-	1	-	-	-
Gorilla gorilla gorilla	-	-	1	1	1	1	2	2	4
Homo sapiens	2	1	1	1	1	1	1	1	4
Homo sapiens JCVenter	-	-	1	-	-	-	-	-	-
Pan troglodytes	2	1	1	1	1	1	1	1	4
Pongo abelii	2	1	1	1	1	1	1	1	3
Callithrix jacchus	-	-	1	1	1	1	2	2	-
Microcebus murinus	-	1	1	-	1	-	1	-	-
Otolemur garnettii	-	1	1	-	1	1	1	-	-
Tupaia belangeri	-	1	1	-	1	2	1	-	-
Canis lupus familiaris breed beagle	-	-	-	-	1	-	-	-	-
Canis lupus familiaris breed boxer	2	1	1	3	1	1	1	1	8
Canis lupus familiaris breed poodle	-	1	1	-	1	-	1	-	-
Ailuropoda melanoleuca	-	-	1	1	1	1	1	2	-
Felis catus	-	1	2	1	1	1	1	-	-
Bos taurus	2	1	1	1	1	1	2	1	6
Capra hircus	-	-	1	1	-	1	-	-	-
Ovis aries	-	-	1	1	1	-	1	1	1
Sus scrofa domestica	1	-	1	1	1	1	1	1	4
Myotis lucifugus	-	1	1	1	1	1	1	-	-
Erinaceus europaeus	-	1	1	-	1	1	-	-	-
Sorex araneus	-	1	1	1	1	1	1	-	-
Equus caballus	2	1	1	1	1	1	1	1	5
Dasypus novemcinctus	-	1	1	-	1	1	1	-	-
Sminthopsis crassicaudata	-	-	-	-	1	-	-	-	-
Monodelphis domestica	2	1	1	1	1	1	1	1	4
Macropus eugenii	-	-	-	-	-	-	1	-	-
Trichosurus vulpecula	-	-	-	1	-	-	1	1	-
Meleagris gallopavo	-	-	1	-	-	1	1	1	-
Gallus gallus	1	2	1	1	1	1	1	1	4
Taeniopygia guttata	1	-	1	1	1	1	1	1	3
Anolis carolinensis	2	1	1	1	1	1	1	1	5
Gekko japonicus	-	-	-	-	1	-	-	-	-
Elaphe quadrivirgata	-	-	-	1	-	-	-	-	-
Xenopus tropicalis	1	1	1	1	1	1	1	1	3
Xenopus laevis	1	2	1	1	1	1	1	1	3
Ambystoma mexicanum	-	-	-	1	-	-	1	-	-

Ambystoma tigrinum tigrinum	-	-	-	1	-	-	1	1	-
Petromyzon marinus	-	-	1	1	1	1	-	1	1
Ciona intestinalis	1	1	1	1	1	1	1	1	2
Ciona savignyi	1	1	1	1	1	1	1	1	2
Molgula tectiformis	-	-	1	1	1	1	-	-	-
Halocynthia roretzi	-	-	1	1	-	1	-	1	-
Asterina pectinifera	-	-	-	1	1	-	-	-	-
Paracentrotus lividus	-	-	-	1	-	1	1	1	-
Strongylocentrotus purpuratus	1	1	1	1	1	1	1	1	2
Saccoglossus kowalevskii	-	-	-	-	-	1	-	-	1
Helobdella robusta	1	1	1	1	1	1	1	1	2
Platynereis dumerilii	-	-	-	-	-	-	-	-	1
Capitella teleta	1	1	1	1	1	1	1	1	2
Mytilus californianus	-	-	-	1	-	1	1	-	-
Crassostrea virginica	-	-	-	1	-	1	-	-	-
Argopecten irradians	-	-	-	-	1	-	-	-	-
Euprymna scolopes	-	-	-	-	1	1	-	-	-
Aplysia californica	1	1	1	1	1	1	1	1	2
Lottia gigantea	1	1	1	1	1	1	1	1	2
Tyrophagus putrescentiae	-	-	-	-	-	-	1	-	-
Amblyomma americanum	-	-	-	1	-	-	-	-	-
Ixodes scapularis str. Wikel	1	-	1	-	1	-	-	-	-
Boophilus microplus	-	-	-	-	-	-	1	-	-
Rhipicephalus appendiculatus	-	-	-	1	1	-	-	-	-
Daphnia magna	-	-	-	-	-	-	-	1	-
Daphnia pulex	1	1	1	1	1	1	1	1	2
Litopenaeus setiferus	-	-	-	-	-	1	-	-	-
Litopenaeus vannamei	-	-	-	1	-	-	-	-	-
Homarus americanus	-	-	-	1	1	-	1	1	-
Carcinus maenas	-	-	-	1	1	-	-	-	-
Calanus finmarchicus	-	-	-	-	-	1	-	-	-
Bombyx mori	1	-	-	-	1	-	-	1	2
Bombyx mori p50T	-	-	-	1	-	-	-	-	-
Bombyx mori str. Dazao	-	1	1	1	1	1	1	1	-
Spodoptera frugiperda	-	-	-	-	-	-	1	-	-
Heliconius erato	-	-	-	-	-	1	1	-	-
Papilio dardanus	-	-	-	-	1	-	-	-	-
Diabrotica virgifera virgifera	-	-	-	-	-	-	1	-	-
Tribolium castaneum str. Georgia GA2	1	1	1	1	1	1	1	1	2
Drosophila mojavensis TSC#15081-1352.22	1	1	2	1	1	1	1	1	2
Drosophila virilis TSC#15010-1051.87	1	1	2	1	1	1	1	1	2
Drosophila grimshawi TSC#15287-2541.00	1	1	2	1	1	1	1	1	2
Drosophila ananassae TSC#14024-0371.13	1	1	2	1	1	1	1	1	2
Drosophila erecta TSC#14021-0224.01	1	1	2	1	1	1	1	1	2
Drosophila melanogaster	1	1	2	1	1	1	1	1	2
Drosophila sechellia Rob3c	1	1	2	1	1	1	1	1	2
Drosophila simulans str. md199	-	-	1	-	-	-	1	-	-
Drosophila simulans str. Mosaic	1	-	-	-	-	-	-	-	2
Drosophila simulans str. sim4	-	-	-	-	-	-	1	-	-
Drosophila simulans str. sim6	-	-	-	-	-	-	1	-	-
Drosophila simulans str. white501	-	1	2	1	1	1	1	1	-
Drosophila yakuba Tai18E2	1	1	2	1	1	1	1	1	2
Drosophila persimilis MSH-3	1	1	2	1	1	1	1	1	2

Drosophila pseudoobscura MV2-25	1	1	2	1	1	1	1	1	2
Drosophila willistoni TSC#14030-0811.24	1	1	2	1	1	1	1	1	2
Glossina morsitans morsitans	-	-	-	1	1	1	-	-	-
Rhynchosciara americana	-	-	-	-	-	-	1	-	-
Culicoides sonorensis	-	-	-	-	1	-	-	-	-
Chironomus tentans	-	-	-	-	-	1	-	-	-
Anopheles gambiae str. PEST	1	1	1	1	1	1	1	1	2
Aedes aegypti str. Liverpool	1	1	1	1	1	1	1	1	2
Armigeres subalbatus	-	-	-	-	1	-	-	-	-
Culex pipiens quinquefasciatus str. JHB	1	1	1	1	1	16	1	1	2
Lutzomyia longipalpis	-	-	-	1	1	1	-	-	-
Apis florea	1	1	1	1	1	1	1	1	2
Apis mellifera str. DH4	1	1	1	1	1	1	1	1	2
Bombus terrestris	1	1	1	1	1	1	1	1	2
Bombus impatiens	1	1	1	1	1	1	1	1	2
Megachile rotundata	1	1	1	1	1	1	1	1	2
Linepithema humile	1	1	1	1	1	1	2	1	2
Camponotus floridanus	1	1	1	1	2	1	2	1	2
Acromyrmex echinatior	1	1	1	1	1	1	2	1	2
Atta cephalotes	1	1	1	1	1	1	1	1	2
Pogonomyrmex barbatus	1	1	1	1	1	2	1	1	2
Solenopsis invicta	1	1	1	1	1	1	1	1	2
Harpegnathos saltator	1	1	1	1	1	1	1	1	2
Nasonia vitripennis str. SymAX	1	1	1	1	1	1	1	1	2
Sphodromantis centralis	-	-	-	-	1	-	-	-	-
Locusta migratoria	-	-	-	1	-	-	-	-	-
Gryllus bimaculatus	-	-	-	-	-	1	-	-	-
Gryllus pennsylvanicus	-	-	-	-	-	-	1	-	-
Laupala kohalensis	-	-	-	-	-	1	-	-	-
Homalodisca coagulata	-	-	-	-	-	-	1	-	-
Rhodnius prolixus	-	-	-	-	-	-	-	-	2
Acyrthosiphon pisum LSR1	1	1	1	1	-	1	1	1	2
Myzus persicae	-	-	1	1	-	1	-	1	-
Maconellicoccus hirsutus	-	-	-	1	-	-	-	-	-
Pediculus humanus corporis str. USDA	1	1	1	1	1	1	1	1	2
Ascaris suum	-	-	-	1	-	1	-	1	-
Pristionchus pacificus	1	1	1	1	1	1	1	1	2
Strongyloides ratti	1	1	1	1	1	1	1	1	2
Strongyloides stercoralis	-	-	-	-	-	1	1	-	-
Heterorhabditis bacteriophora	1	1	1	1	1	1	1	1	2
Caenorhabditis brenneri	1	1	2	2	1	1	1	1	2
Caenorhabditis briggsae	1	1	1	1	1	1	1	1	2
Caenorhabditis elegans	1	1	1	1	1	1	1	1	2
Caenorhabditis japonica DF5081	-	-	1	-	-	-	-	-	1
Caenorhabditis remanei PB4641	1	1	1	1	1	2	1	1	2
Ancylostoma caninum	-	-	-	-	1	1	-	-	-
Haemonchus contortus	-	-	-	-	-	-	1	-	-
Nippostrongylus brasiliensis	-	-	-	-	-	-	1	-	-
Brugia malayi	1	1	2	1	1	1	1	1	2
Litomosoides sigmodontis	-	-	-	-	-	1	-	-	-
Loa loa	1	1	2	1	1	1	1	1	2
Onchocerca volvulus	-	-	-	-	1	-	-	-	-
Wuchereria bancrofti	1	1	2	1	1	1	1	1	2
Globodera rostochiensis	-	-	-	-	-	1	-	-	-

Heterodera glycines	-	-	-	-	-	1	1	-	1
Meloidogyne hapla	-	1	-	-	-	1	1	-	2
Meloidogyne incognita	-	1	-	-	-	-	-	-	1
Pratylenchus vulnus	-	-	-	-	-	-	1	-	-
Xiphinema index	-	-	-	-	-	-	1	1	-
Trichinella spiralis	1	1	1	1	1	1	1	1	2
Trichuris muris	-	-	-	-	-	-	-	1	-
Philodina roseola	-	-	-	-	-	-	1	-	-
Brachionus plicatilis	-	-	-	-	-	1	-	-	-
Nematostella vectensis CH2 x CH6	1	1	1	1	1	1	1	1	2
Acropora millepora	-	-	-	-	-	1	1	-	1
Acropora palmata	-	-	-	-	-	1	-	-	-
Hydra magnipapillata	1	1	-	1	1	-	1	1	2
Mnemiopsis leidyi	-	-	-	-	-	-	-	1	-
Trichoplax adhaerens Grell-BS-1999	1	1	1	1	1	1	1	1	2
Amphimedon queenslandica	1	1	1	-	1	1	1	1	2
Oscarella carmela	-	-	-	1	-	-	-	-	-
Capsaspora owczarzaki ATCC 30864	1	-	1	1	1	1	1	1	2
Sphaeroforma arctica	1	-	-	1	-	1	1	1	2
Trichomonas vaginalis G3	-	-	-	-	-	-	-	-	7
Bigelowiella natans	1	-	1	1	-	1	1	-	2
Fragilariopsis cylindrus	1	-	1	1	-	1	1	-	2
Phaeodactylum tricornutum CCAP1055/1	1	-	1	1	-	1	1	-	2
Thalassiosira pseudonana CCMP1335	1	-	1	1	-	1	1	-	-
Blastocystis hominis	-	-	-	-	-	-	-	-	2
Albugo laibachii Nc14	1	1	1	1	-	1	1	1	2
Hyaloperonospora arabidopsidis Emoy2	1	1	1	1	-	1	1	1	2
Phytophthora brassicae	-	-	-	-	-	-	-	1	-
Phytophthora ramorum Pr102	1	1	1	1	-	1	1	1	2
Phytophthora sojae P6497	1	1	1	1	-	1	1	1	2
Phytophthora infestans T30-4	1	1	1	1	-	1	1	1	2
Pythium ultimum DAOM BR144	1	1	1	1	-	1	1	1	2
Aphanomyces euteiches	-	-	-	1	-	1	-	-	-
Saprolegnia parasitica CBS 223.65	1	1	1	1	-	1	1	1	2
Aureococcus anophagefferens	-	-	-	-	-	1	1	1	3
Ectocarpus siliculosus	1	-	1	1	-	1	1	1	2
Trimastix pyriformis	-	-	-	-	1	-	-	-	-
Physcomitrella patens	-	-	-	-	-	-	-	-	3
Lycopersicon esculentum	-	-	-	-	-	-	-	-	2
Glycine max	-	-	-	-	-	-	-	-	2
Medicago truncatula	-	-	-	-	-	-	-	-	1
Ricinus communis	-	-	-	-	-	-	-	-	2
Populus trichocarpa	-	-	-	-	-	-	-	-	3
Brassica rapa	-	-	-	-	-	-	-	-	3
Arabidopsis lyrata	-	-	-	-	-	-	-	-	2
Arabidopsis thaliana	-	-	-	-	-	-	-	-	2
Capsella rubella	-	-	-	-	-	-	-	-	2
Eutrema halophilum	-	-	-	-	-	-	-	-	2
Eutrema parvulum	-	-	-	-	-	-	-	-	2
Carica papaya	-	-	-	-	-	-	-	-	2
Theobroma cacao	-	-	-	-	-	-	-	-	2
Gossypium raimondii	-	-	-	-	-	-	-	-	3
Eucalyptus camaldulensis	-	-	-	-	-	-	-	-	2
Eucalyptus grandis	-	-	-	-	-	-	-	-	2

Citrus clementina	-	-	-	-	-	-	-	-	2
Citrus sinensis	-	-	-	-	-	-	-	-	2
Vitis vinifera	-	-	-	-	-	-	1	-	2
Oryza barthii	-	-	-	-	-	-	-	-	1
Oryza sativa (indica cultivar-group)	-	-	-	-	-	-	-	-	2
Oryza sativa (japonica cultivar-group)	-	-	-	-	-	-	-	-	2
Triticum aestivum	-	-	-	-	-	-	-	-	1
Sorghum bicolor	-	-	-	-	-	-	-	-	2
Zea mays B73	-	-	-	-	-	-	-	-	2
