# Supplementary material for: Evolution of the eukaryotic dynactin complex, the activator of cytoplasmic dynein
Source: BMC Evol Biol. 2012 Jun 22;12:95. doi: 10.1186/1471-2148-12-95 (PMC3583065; doi:10.1186/1471-2148-12-95)
Supplement: Additional file 2 — Dynactin inventory of the analysed species. The file lists the presence and number of orthologs for each dynactin subunit for each analysed organism in taxonomic order. [file 1471-2148-12-95-S2.pdf]

Evidence, that the possible alternative splice forms of p24 are non-functional pseudo-transcripts

The human dynactin3 is encoded in 7 exons on chromosome 9. All of them are constitutively spliced. Dynactin3 of *Homo sapiens* can also be transcribed into pseudo-transcripts. The alternative transcription of exon 6 leads to pseudo-transcripts because of the following reasons:

(A) The resulting translation is much shorter and the alternative region does not have any homology to dynactin3 proteins from other species (Figure 1).

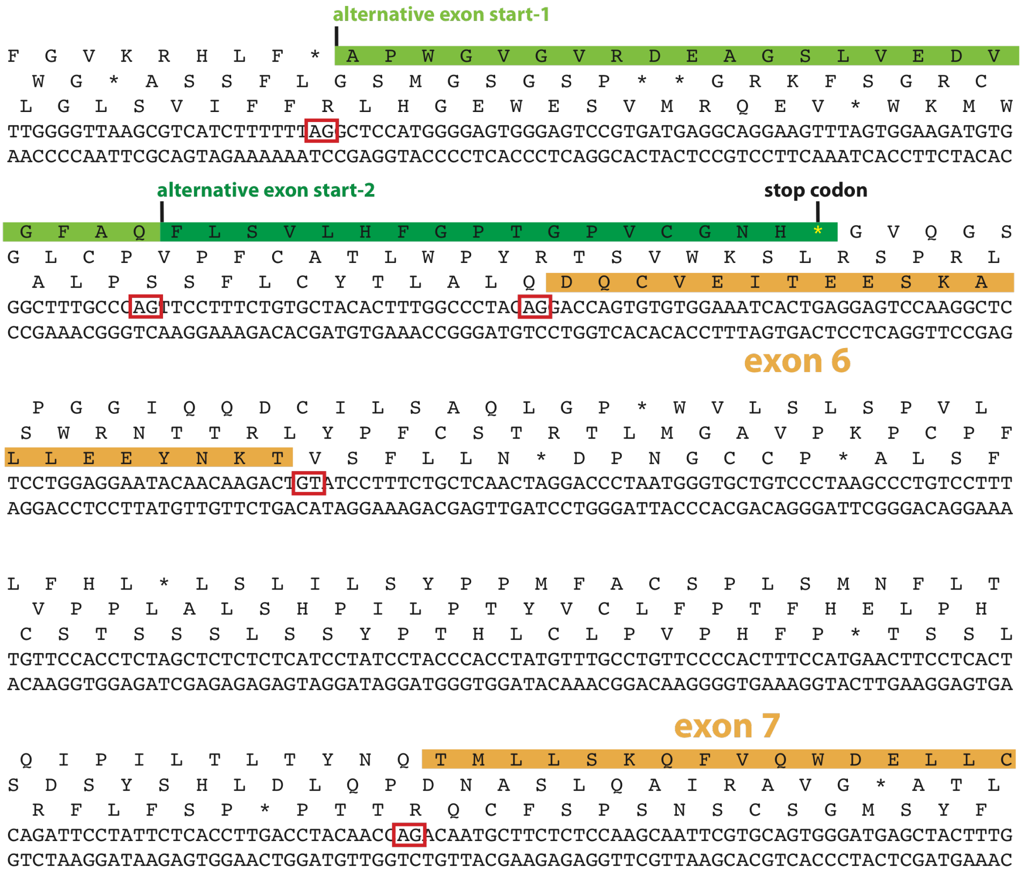

Figure 1: Six-frame translation of the genomic DNA around exon 6 and exon 7. Intron 3' splice sites ("AG") and 5' splice sites ("GT") are marked with red squares. The alternative pseudoexon might start at two different positions, alternative exon start-1 and alternative exon start-2, and ends with a stop codon.

(B) While the correct transcript is supported by hundreds of EST and cDNA clones covering all species there are only a few EST clones covering the alternative transcript.

ESTs/cDNAs found containing exon 6: hundreds covering all species

ESTs/cDNAs found containing the alternative exon start-1: 7 in *Homo sapiens*

ESTs/cDNAs found containing the alternative exon start-2: 1 in *Homo sapiens*
1 in *Canis familiaris*

(C) The alternative splice sites of the exon and the stop codon of the alternative transcript (Figure 1) are not even conserved in the genomes of the sequenced primates, and many of the sequenced mammals do not encode a potentially functional alternative transcript at all (Table 1).

Table 1: Possible alternative exon start-1 and start-2 in mammalian genomes (based on potentially correct splice sites, stop-codons at the end of the exon, no in-frame stop codons in the exons, no frame-shifts):

**Species**	**alt. start-1**	**alt. start-2**	**exon 6**
human, chimp, Pongo, dog	yes	yes	yes
Macaca, cat, panda bear	yes	no	yes
Gorilla, Callithrix	no	yes	yes
rabbit, horse, elephant, microbat, mouse, rat, cow	no	no	yes
