# Supplementary material for: Evolution of the eukaryotic dynactin complex, the activator of cytoplasmic dynein
Source: BMC Evol Biol. 2012 Jun 22;12:95. doi: 10.1186/1471-2148-12-95 (PMC3583065; doi:10.1186/1471-2148-12-95)
Supplement: Additional file 4 — Detailed description of the pseudo-transcripts of dynactin4. The file contains details about the pseudo-transcripts of dynactin4 [file 1471-2148-12-95-S4.pdf]

Evidence, that the possible alternative splice forms of dynactin3 (p24) are non-functional pseudo-transcripts

The human dynactin3 is encoded in 7 exons on chromosome 9. All of them are constitutively spliced. Dynactin3 of *Homo sapiens* can also be transcribed into pseudo-transcripts (Figure 1).

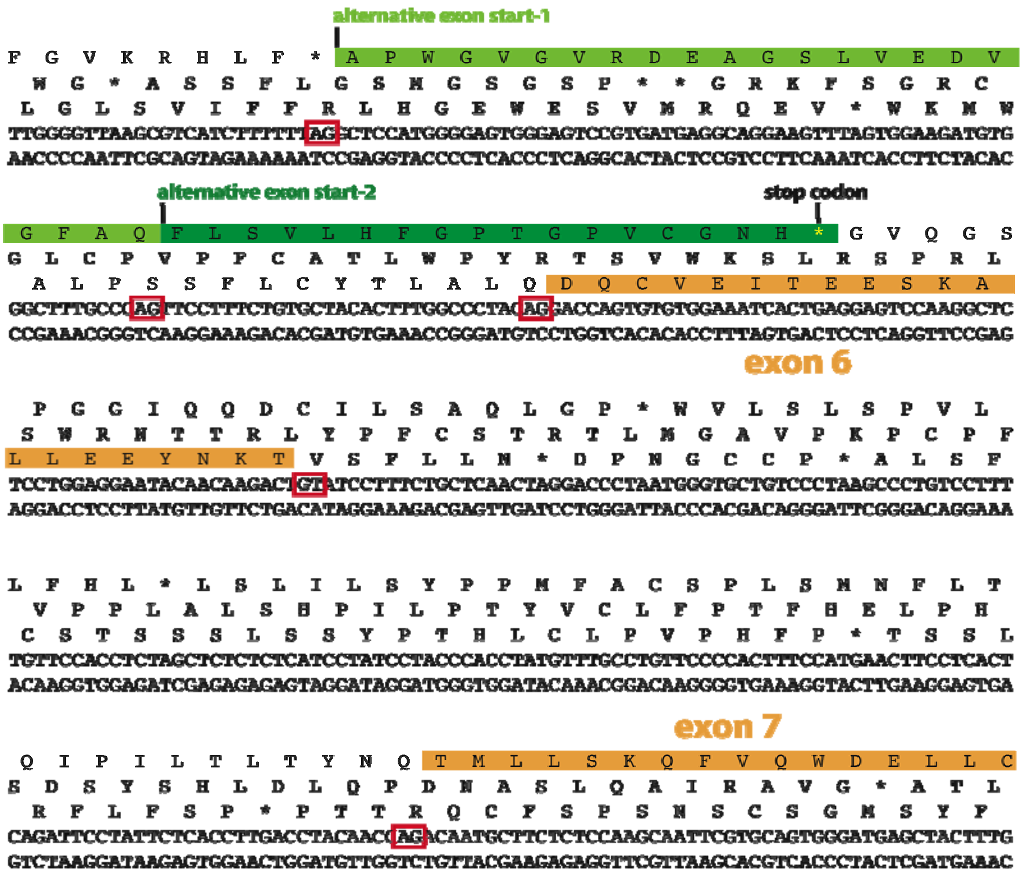

Figure 1: Three-frame translation of the genomic DNA around exon 6 and exon 7 from human dynactin3. Intron 3' splice sites ("AG") and 5' splice sites ("GT") are marked with red boxes. The alternative pseudoexon could start at two different positions, alternative exon start-1 and alternative exon start-2, and ends with a stop codon.

The alternative transcription of exon 6 leads to pseudo-transcripts because of the following reasons:

(A) The resulting alternative transcript would be much shorter than the correct transcript and the alternative region does not have any similarity to dynactin3 proteins from other species.

(B) While the correct transcript is supported by hundreds of EST and cDNA clones covering all species there are only a few EST clones covering the alternative transcript.

ESTs/cDNAs found containing exon 6: hundreds covering all species

ESTs/cDNAs found containing the alternative exon start-1: 7 in *Homo sapiens*

ESTs/cDNAs found containing the alternative exon start-2: 1 in *Homo sapiens*
1 in *Canis familiaris*

(C) The alternative splice sites of the exon and the stop codon of the alternative transcript (Figure 1) are not even conserved in the genomes of the sequenced primates, and potentially alternative transcripts of many of the sequenced mammals would contain disablements like frame-shifts and in-frame stop codons compared to the human alternative transcripts. Many of the sequenced mammals do not encode a potentially functional alternative transcript at all (Table 1).

Table 1: Possible alternative exon start-1 and start-2 in mammalian genomes (based on potentially correct splice sites, stop-codons at the end of the exon, no in-frame stop codons in the exons, no frame-shifts):

**Species**	**alt. start-1**	**alt. start-2**	**exon 6**
human, chimp, Pongo, dog	yes	yes	yes
Macaca, cat, panda bear	yes	no	yes
Gorilla, Callithrix	no	yes	yes
rabbit, horse, elephant, microbat, mouse, rat, cow	no	no	yes
