# Supplementary material for: Evolution of the eukaryotic dynactin complex, the activator of cytoplasmic dynein
Source: BMC Evol Biol. 2012 Jun 22;12:95. doi: 10.1186/1471-2148-12-95 (PMC3583065; doi:10.1186/1471-2148-12-95)
Supplement: Additional file 5 — Sequence alignment of fungal dynactin4 proteins. The file contains the sequence alignment of the N-termini of several fungal dynactin4 (p62) subunits showing that the upstream methionines in Neurospora and Sordaria are most likely not the translation start sites. [file 1471-2148-12-95-S5.pdf]

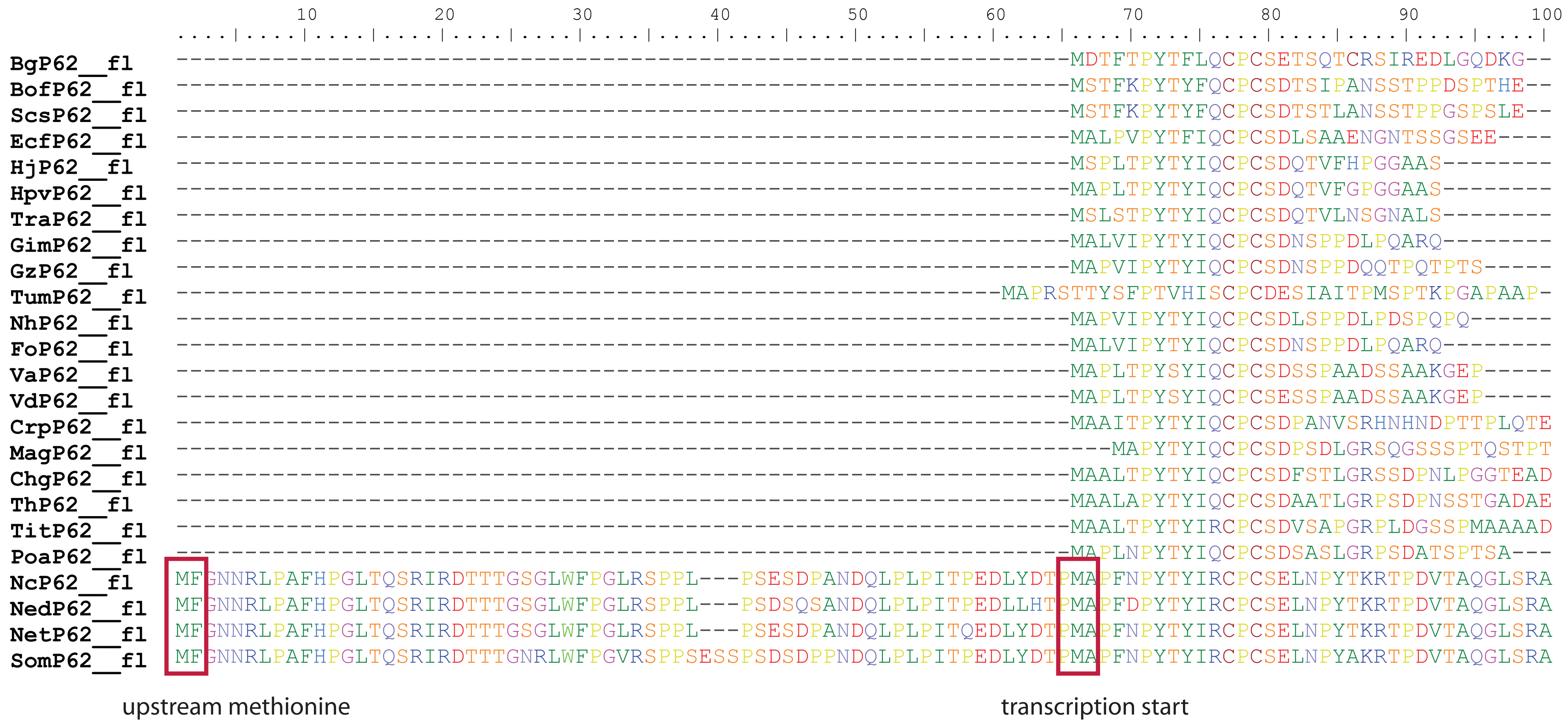

Nc = Neurospora crassa, Ned = Neurospora discreta, Net = Neurospora tetrasperma, Som = Sordaria macrospora
