# Supplementary material for: Evolution of the eukaryotic dynactin complex, the activator of cytoplasmic dynein
Source: BMC Evol Biol. 2012 Jun 22;12:95. doi: 10.1186/1471-2148-12-95 (PMC3583065; doi:10.1186/1471-2148-12-95)
Supplement: Additional file 6 — Phylogenetic tree of Arp1. The file contains the phylogenetic tree of Arp1 focused on the vertebrate branch highlighting the Arp1 gene duplication event and subsequent branch-specific losses of Arp1 subtypes. [file 1471-2148-12-95-S6.pdf]

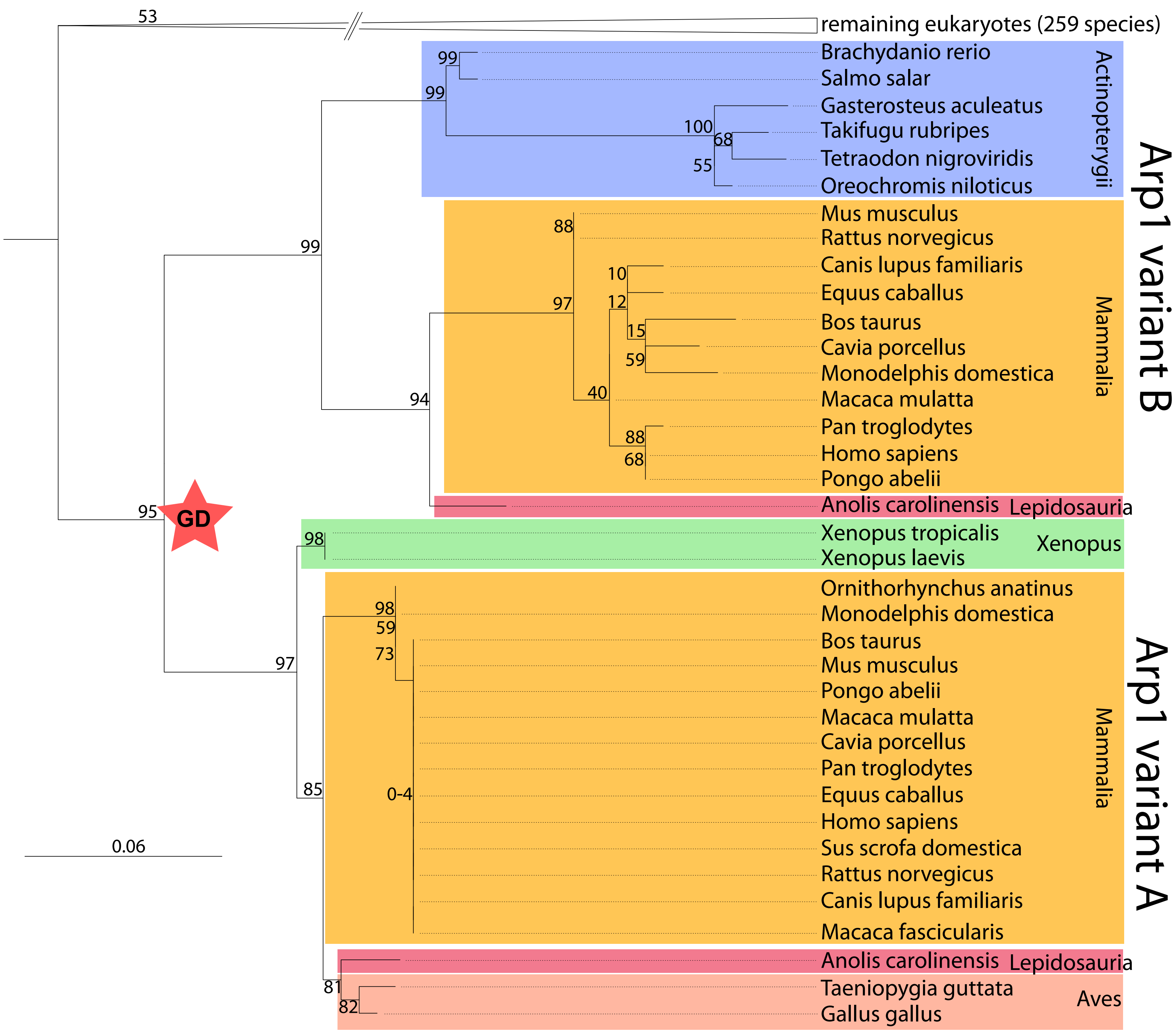

Phylogeny of Arp1 in vertebrates

Phylogenetic tree from Bayesian inference of the Arp1s illustrating the Arp1 duplication event at the origin of the vertebrates (GD), which may have been the result from the two whole genome duplications that happend at the origin of the vertebrates.. The branch containing the remaining eukaryotes has been collapsed. The numbers on branches indicate posterior probabiliy support values using the LG model. The complete tree including the posterior probabilities for all branches is available in Additional File 1. For vertebrates, two different variants were found, based on the duplication event at the origin of the vertebrates. Subsequently, most vertebrate branches have lost one of the versions. Mammalian variant A grouped together with the variants of birds and Amphibia, while mammalian variant B grouped together with the Arp1 of Actinopterygii. The lizard Anolis carolinensis also still encodes two Arp1 genes. The scale bar represents amino acid substitutions per site.
