# Supplementary material for: Evolution of the eukaryotic dynactin complex, the activator of cytoplasmic dynein
Source: BMC Evol Biol. 2012 Jun 22;12:95. doi: 10.1186/1471-2148-12-95 (PMC3583065; doi:10.1186/1471-2148-12-95)
Supplement: Additional file 7 — Discussion of the evolution of the Cap proteins. The file contains a discussion of the gene duplications, which happened to the Capα (Cap1) subunit, and of alternative splice variants of the Capβ (Cap2) subunit [120,121]. [file 1471-2148-12-95-S7.pdf]

Evolution of the conventional actin capping protein Cap

CapZ, the heterodimeric cytoplasmic actin-capping protein, caps the barbed-end of the Arp1 mini-filament. Because of their ubiquitous cytoplasmic function, the CapZ subunits Capα (Cap1) and Capβ (Cap2) have also been found in plants and algae that do not contain any other dynactin subunit. CapZ homologs have been found in all available eukaryotes except the diatom *Thalassiosira pseudonana*. The reason is unknown but unlikely to be an unexpectedly large divergence because homologs were identified in the closely related species *Fragilariopsis cylindrus* and *Phaeodactylum tricornutum*. The reason could be a gap in the genome assembly although it is unlikely that both capping proteins were missing because of gaps.

Cap1 (Capα)

368 Capα sequences were identified in 289 species (Table 1 in the main manuscript, Additional File 1). All eukaryotes encode at least one copy of Cap1 (Capα). Several Cap1 homologs have been identified in vertebrates (Figure 1). However, the duplication pattern of the homologs of the Actinopterygii, Amphibia, Sauropsida, and Mammalia branches cannot easily be explained by the two whole genome duplications that happened at the origin of the vertebrates [1]. Instead, there must have been either additional single gene duplications with further gene losses in certain branches, or the homologs must have diverged so far that their phylogenetic relationship cannot be resolved with current phylogenetic methods. Therefore, variant designations do not necessarily correspond to evolutionary relationships between these branches. The most remarkable exceptions in the branches listed above are: in-between the mammalia branch, the rodents show another Cap1 duplication, which is related to Cap1A (Capα1), the lizard *Anolis carolinensis* encodes an additional homolog compared to the other Sauropsidae, which is related to Cap1C (Capα3), and the Cap1 variants of *Brachydanio rerio* do not group to the variants of the other fish. Both major mammalian Cap1 isoforms, Cap1A (Capα1) and Cap1B (Capα2), are present in dynactin from brain [2] although their ratio is unknown.

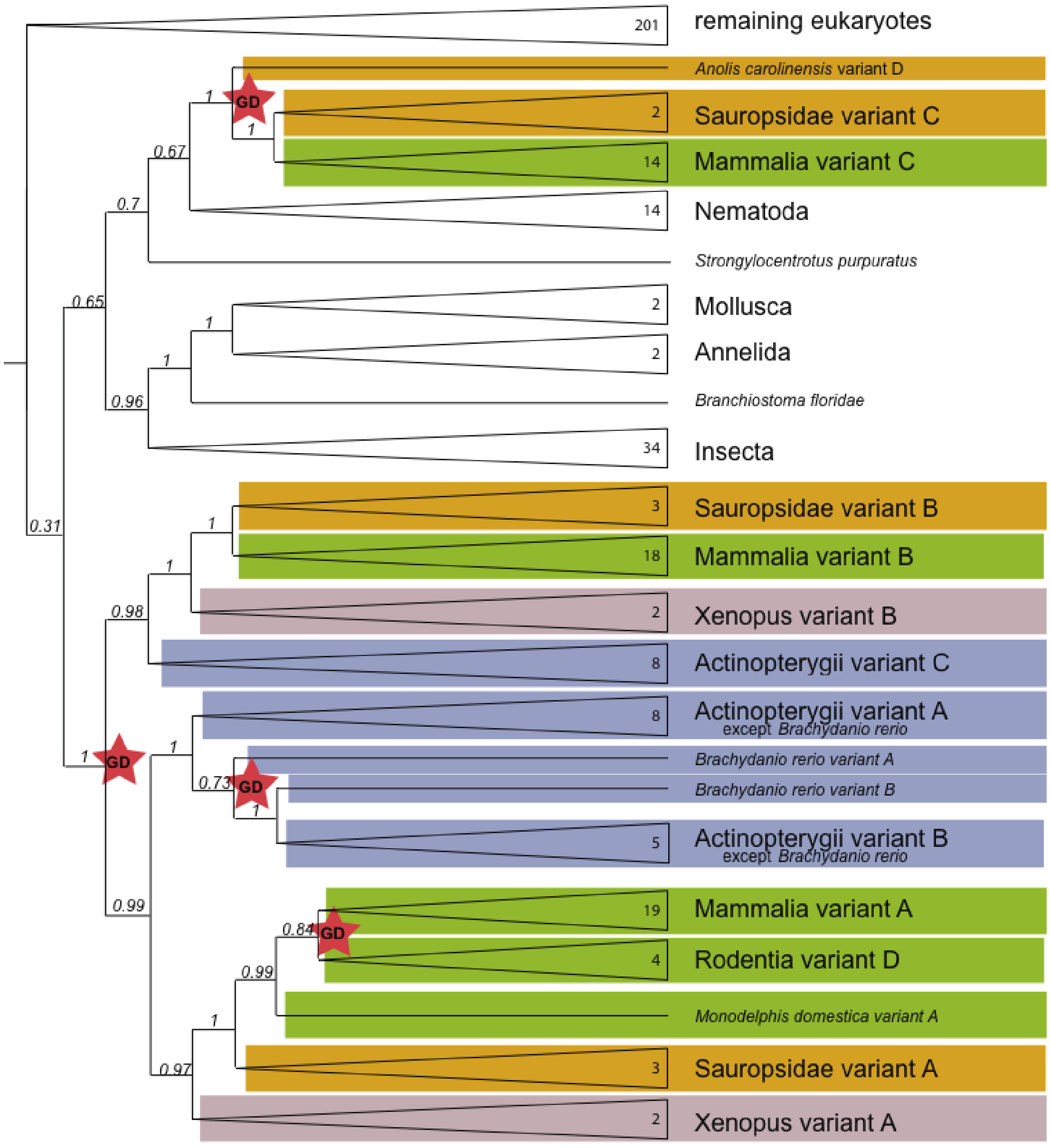

Figure 1 - Phylogeny of Cap1 (Capα) in vertebrates

Bayesian tree (MrBayes, WAG model) of the Cap1 protein dataset (368 sequences). Major branches have been collapsed to highlight the duplication events of Cap1 in Metazoa (red stars with "GD"). Small numbers within the triangles denote the number of included leafs. Branch support values correspond to Bayesian posterior probabilities. Mammals, Sauropsidae, frogs and fishes are coloured in green, orange, purple and blue, respectively, for better navigation through the tree.

The three Cap1 variants of *Homo sapiens* are located on chromosome 1 (variant A), chromosome 7 (variant B), and chromosome 12 (variant C), respectively (Figure 2). The variant A and variant B genes consist of 10 exons while variant C is encoded by a single exon. The identical gene structures of the variant A and variant B genes support

their origin from a common ancestor. Variant C most probably derived by retro-transciption of a processed ancestral Cap1 gene, a process leading to pseudo-genes in most cases. However, because several dozens of EST and cDNA clones strongly support its cellular expression, the ancestral variant C gene must have gained a new promoter region.

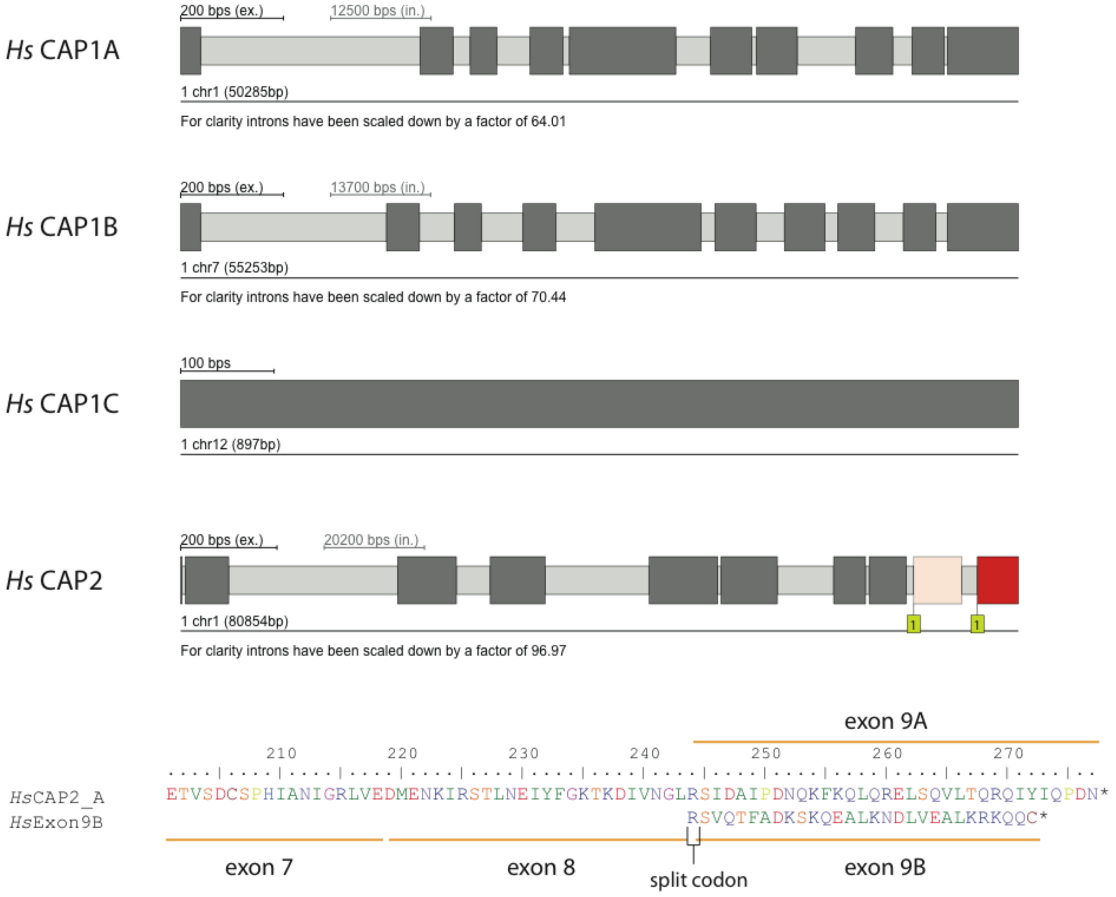

Figure 2 - Gene structures of the Capα and Capβ homologs of *Homo sapiens*

Three variants of Capα have been identified in *Homo sapiens*. Variant A (found on chromosome 1) and B (found on chromosome 7), both have 10 exons. Variant C (found on chromosome 12) consists of one exon. For Capβ, one copy was found in the human genome comprised of 10 exons, of which exons 9A and 9B are alternatively spliced.

Cap2 (Capβ)

299 Capβ sequences have been assembled from 292 species (Table 1 in the main manuscript, Additional File 1). Cap2 was found in all eukaryotic species except the

diatom *Thalassiosira pseudonana*. Duplicates have been identified in *Trichomonas vaginalis* (five Cap2 homologs) and *Paramecium tetraurelia* (two Cap2 homologs). Mutually exclusive splicing [3] increases the vertebrates Cap2 diversity. The Cap2 gene of *Homo sapiens* is located on chromosome 1 and consists of ten exons of which the first eight are constitutively transcribed and exons 9A and 9B are mutually exclusive spliced (Figure 2). The transcripts including exon 9A are also called Capβ1 and the transcripts including exon 9B Capβ2. Many EST and cDNA clones support both mutually exclusive spliced exons in most vertebrates while invertebrates do not encode alternatively spliced Cap2 genes. Unfortunately, the Cap2 gene is not included in the fragmented draft assembly of *Petromyzon marinus* so that it is not clear yet whether the alternatively spliced form has been invented at the origin of the vertebrates or the Gnathostomata. Of the two Cap2 isoforms, only the Capβ1 isoform (including exon 9A) has been found in the dynactin complex [4].

1. Van de Peer Y, Maere S, Meyer A: **2R or not 2R is not the question anymore**. *Nat Rev Genet* 2010, **11**:166.

2. Kozielski F, Riaz T, DeBonis S, Koehler CJ, Kroening M, Panse I, Strozynski M, Donaldson IM, Thiede B: **Proteome analysis of microtubule-associated proteins and their interacting partners from mammalian brain**. *Amino Acids* 2010, **41**:363–385.

3. Pillmann H, Hatje K, Odronitz F, Hammesfahr B, Kollmar M: **Predicting mutually exclusive spliced exons based on exon length, splice site and reading frame conservation, and exon sequence homology**. *BMC Bioinformatics* 2011, **12**:270.

4. Schafer DA, Korshunova YO, Schroer TA, Cooper JA: **Differential localization and sequence analysis of capping protein beta-subunit isoforms of vertebrates**. *J Cell Biol* 1994, **127**:453–465.
