# Supplementary material for: Evolution of the eukaryotic dynactin complex, the activator of cytoplasmic dynein
Source: BMC Evol Biol. 2012 Jun 22;12:95. doi: 10.1186/1471-2148-12-95 (PMC3583065; doi:10.1186/1471-2148-12-95)
Supplement: Additional file 8 — Species table. The file contains all species of the analysis, their scientific names, the abbreviation as used in the sequence alignments and trees, the species taxonomy, references to sequencing centers, and publications if genome analyses have already been published. [file 1471-2148-12-95-S8.pdf]

***Plasmodium gallinaceum* (Plg)**
Taxonomy:
cellular organisms | Eukaryota | Alveolata | Apicomplexa | Aconoidasida | Haemosporida | Plasmodium | Plasmodium (Haemamoeba)
Reference/s:
PlasmoDB The Plasmodium Genome Resource: PlasmoDB The Plasmodium Genome Resource
The Wellcome Trust Sanger Institute: Plasmodium gallinaceum, Partial Genome Shotgun

***Plasmodium falciparum Ghanaian Isolate* (Pf_b)**
Taxonomy:
cellular organisms | Eukaryota | Alveolata | Apicomplexa | Aconoidasida | Haemosporida | Plasmodium | Plasmodium (Laverania)
Reference/s:
International Species Sequencing Consortium: Plasmodium Sequencing Consortium
The Gene Index Project: DFCI P.falciparum Gene Index
The Wellcome Trust Sanger Institute: Plasmodium falciparum Genome Projects
Publication/s:
Jeffares DC *et. al.* , *Nat Genet* , **39** , 120 (2007).

***Plasmodium falciparum Dd2* (Pf_d)**
Taxonomy:
cellular organisms | Eukaryota | Alveolata | Apicomplexa | Aconoidasida | Haemosporida | Plasmodium | Plasmodium (Laverania) | Plasmodium falciparum
Reference/s:
Broad Institute of Harvard and MIT: Plasmodium falciparum species Database
International Species Sequencing Consortium: Plasmodium Sequencing Consortium
National Center for Biotechnology Information: NCBI Protozoa genomes
The Gene Index Project: DFCI P.falciparum Gene Index
Publication/s:
Volkman SK *et. al.* , *Nat Genet* , **39** , 113 (2007).

***Plasmodium falciparum HB3* (Pf_c)**
Taxonomy:
cellular organisms | Eukaryota | Alveolata | Apicomplexa | Aconoidasida | Haemosporida | Plasmodium | Plasmodium (Laverania) | Plasmodium falciparum
Reference/s:
Broad Institute of Harvard and MIT: Plasmodium falciparum species Database
International Species Sequencing Consortium: Plasmodium Sequencing Consortium
National Center for Biotechnology Information: NCBI Protozoa genomes
The Gene Index Project: DFCI P.falciparum Gene Index
Publication/s:
Volkman SK *et. al.* , *Nat Genet* , **39** , 113 (2007).

***Plasmodium falciparum 3D7* (Pf_a)**
Taxonomy:
cellular organisms | Eukaryota | Alveolata | Apicomplexa | Aconoidasida | Haemosporida | Plasmodium | Plasmodium (Laverania) | Plasmodium falciparum
Reference/s:
International Species Sequencing Consortium: Plasmodium falciparum Sequencing Consortium
National Center for Biotechnology Information: NCBI Protozoa genomes
National Center for Biotechnology Information Reference Sequences: Plasmodium falciparum genome view
PlasmoDB The Plasmodium Genome Resource: PlasmoDB The Plasmodium Genome Resource
The Gene Index Project: DFCI P.falciparum Gene Index
The Wellcome Trust Sanger Institute: Plasmodium falciparum Genome Projects
e! Ensembl: Plasmodium falciparum
Publication/s:
Gardner MJ *et. al.* , *Nature* , **419** , 498 (2002).
Hall N *et. al.* , *Nature* , **419** , 527 (2002).
Gardner MJ *et. al.* , *Nature* , **419** , 531 (2002).
Hyman RW *et. al.* , *Nature* , **419** , 534 (2002).
Bowman S *et. al.* , *Nature* , **400** , 532 (1999).
Gardner MJ *et. al.* , *Science* , **282** , 1126 (1998).
Wilson RJ *et. al.* , *J Mol Biol* , **261** , 155 (1996).

***Plasmodium knowlesi* (Pk)**
Taxonomy:
cellular organisms | Eukaryota | Alveolata | Apicomplexa | Aconoidasida | Haemosporida | Plasmodium | Plasmodium (Plasmodium)
Reference/s:
International Species Sequencing Consortium: Plasmodium knowlesi Sequencing Consortium
National Center for Biotechnology Information: NCBI Protozoa genomes
PlasmoDB The Plasmodium Genome Resource: PlasmoDB The Plasmodium Genome Resource
The Wellcome Trust Sanger Institute: Plasmodium knowlesi, Genome Shotgun
e! Ensembl: Plasmodium knowlesi
Publication/s:
Pain A *et. al.* , *Nature* , **455** , 799 (2008).

***Plasmodium vivax str. Salvador I* (Pv)**
Taxonomy:
cellular organisms | Eukaryota | Alveolata | Apicomplexa | Aconoidasida | Haemosporida | Plasmodium | Plasmodium (Plasmodium) | Plasmodium vivax
Reference/s:
GenBank - NIH genetic sequence database: GenBank species TBLASTN
International Species Sequencing Consortium: Plasmodium vivax Sequencing Consortium
National Center for Biotechnology Information: NCBI Protozoa genomes
PlasmoDB The Plasmodium Genome Resource: PlasmoDB The Plasmodium Genome Resource

The Gene Index Project: DFCI Plasmodium vivax Gene Index
The Institute for Genomic Research: Plasmodium vivax Genome Project
e! Ensembl: Plasmodium vivax
Publication/s:
Dharia NV *et. al.* , *Proc Natl Acad Sci U S A* , **107** , 20045 (2010).
Carlton JM *et. al.* , *Nature* , **455** , 757 (2008).
Cui L *et. al.* , *Mol Biochem Parasitol* , **144** , 1 (2005).

Plasmodium chabaudi **(Plc)**
Taxonomy:
cellular organisms | Eukaryota | Alveolata | Apicomplexa | Aconoidasida | Haemosporida | Plasmodium | Plasmodium (Vinckeia)
Reference/s:
GenBank - NIH genetic sequence database: GenBank species TBLASTN
National Center for Biotechnology Information: NCBI Protozoa genomes
PlasmoDB The Plasmodium Genome Resource: PlasmoDB The Plasmodium Genome Resource
The Wellcome Trust Sanger Institute: Plasmodium chabaudi genome project
e! Ensembl: Plasmodium chabaudi
Publication/s:
Hall N *et. al.* , *Science* , **307** , 82 (2005).

Plasmodium berghei str. ANKA **(Pb)**
Taxonomy:
cellular organisms | Eukaryota | Alveolata | Apicomplexa | Aconoidasida | Haemosporida | Plasmodium | Plasmodium (Vinckeia) | Plasmodium berghei
Reference/s:
GenBank - NIH genetic sequence database: GenBank species TBLASTN
National Center for Biotechnology Information: NCBI Protozoa genomes
PlasmoDB The Plasmodium Genome Resource: PlasmoDB The Plasmodium Genome Resource
The Gene Index Project: DFCI Plasmodium berghei Gene Index
The Wellcome Trust Sanger Institute: Plasmodium berghei, Genome Shotgun
e! Ensembl: Plasmodium berghei
Publication/s:
Hall N *et. al.* , *Science* , **307** , 82 (2005).

Plasmodium yoelii yoelii 17XNL **(Ply)**
Taxonomy:
cellular organisms | Eukaryota | Alveolata | Apicomplexa | Aconoidasida | Haemosporida | Plasmodium | Plasmodium (Vinckeia) | Plasmodium yoelii
Reference/s:
International Species Sequencing Consortium: Plasmodium yoelii yoelii Sequencing Consortium
National Center for Biotechnology Information: NCBI Protozoa genomes
PlasmoDB The Plasmodium Genome Resource: PlasmoDB The Plasmodium Genome Resource
The Gene Index Project: DFCI Plasmodium yoelii Gene Index
The Institute for Genomic Research: The Plasmodium yoelii yoelii Genome Sequencing Program
Publication/s:
Carlton JM *et. al.* , *Nature* , **419** , 512 (2002).

Babesia bovis str. Texas T2Bo **(Bb)**
Taxonomy:
cellular organisms | Eukaryota | Alveolata | Apicomplexa | Aconoidasida | Piroplasmida | Babesiidae | Babesia
Reference/s:
International Species Sequencing Consortium: Babesia bovis Sequencing Consortium
National Center for Biotechnology Information: NCBI Protozoa genomes
The Wellcome Trust Sanger Institute: Babesia bovis EST Sequencing Project
Washington State University: Babesia bovis Genome Sequencing Project
Publication/s:
Brayton KA *et. al.* , *PLoS Pathog* , **3** , 1401 (2007).

Theileria parva str. Muguga **(Tep)**
Taxonomy:
cellular organisms | Eukaryota | Alveolata | Apicomplexa | Aconoidasida | Piroplasmida | Theileriidae | Theileria | Theileria parva
Reference/s:
International Species Sequencing Consortium: Theileria parva Sequencing Consortium
National Center for Biotechnology Information: NCBI Protozoa genomes
The Institute for Genomic Research: The Theileria parva Genome Database
Publication/s:
Gardner MJ *et. al.* , *Science* , **309** , 134 (2005).

Cryptosporidium parvum str. Iowa II **(Cp)**
Taxonomy:
cellular organisms | Eukaryota | Alveolata | Apicomplexa | Coccidia | Eucoccidiorida | Eimeriorina | Cryptosporidiidae | Cryptosporidium
Reference/s:
CryptoDB: CryptoDB
International Species Sequencing Consortium: Cryptosporidium parvum Sequencing Consortium
National Center for Biotechnology Information: NCBI Protozoa genomes
The Gene Index Project: DFCI Cryptosporidium parvum Gene Index
Virginia Commonwealth University: Cryptosporidium hominis Research
Publication/s:
Abrahamsen MS *et. al.* , *Science* , **304** , 441 (2004).

Cryptosporidium hominis TU502 **(Ch)**
Taxonomy:

cellular organisms | Eukaryota | Alveolata | Apicomplexa | Coccidia | Eucoccidiorida | Eimeriorina | Cryptosporidiidae | Cryptosporidium | Cryptosporidium hominis
Reference/s:
CryptoDB: CryptoDB
National Center for Biotechnology Information: NCBI Protozoa genomes
Virginia Commonwealth University: Cryptosporidium hominis Research
Publication/s:
Xu P *et. al.* , *Nature* , **431** , 1107 (2004).

Cryptosporidium muris RN66 **(Crm)**
Taxonomy:
cellular organisms | Eukaryota | Alveolata | Apicomplexa | Coccidia | Eucoccidiorida | Eimeriorina | Cryptosporidiidae | Cryptosporidium | Cryptosporidium muris
Reference/s:
CryptoDB: CryptoDB
National Center for Biotechnology Information: NCBI Protozoa genomes

Eimeria tenella **(Et)**
Taxonomy:
cellular organisms | Eukaryota | Alveolata | Apicomplexa | Coccidia | Eucoccidiorida | Eimeriorina | Eimeriidae | Eimeria
Reference/s:
The Gene Index Project: DFCI Eimeria tenella Gene Index
The Wellcome Trust Sanger Institute: The Eimeria tenella Genome Project
ToxoDB: Toxoplasma gondii Genome resource

Neospora caninum **(Nca)**
Taxonomy:
cellular organisms | Eukaryota | Alveolata | Apicomplexa | Coccidia | Eucoccidiorida | Eimeriorina | Sarcocystidae | Neospora
Reference/s:
National Center for Biotechnology Information: NCBI Protozoa genomes
The Gene Index Project: DFCI Neospora caninum Gene Index
The Wellcome Trust Sanger Institute: Neospora caninum Sequencing
ToxoDB: Toxoplasma gondii Genome resource

Toxoplasma gondii GT1 **(Tg_a)**
Taxonomy:
cellular organisms | Eukaryota | Alveolata | Apicomplexa | Coccidia | Eucoccidiorida | Eimeriorina | Sarcocystidae | Toxoplasma | Toxoplasma gondii
Reference/s:
National Center for Biotechnology Information: NCBI Protozoa genomes
The Gene Index Project: DFCI Toxoplasma gondii Gene Index
The Institute for Genomic Research: The Toxoplasma gondii Genome Project
The Wellcome Trust Sanger Institute: Toxoplasma gondii Sequencing Project
ToxoDB: Toxoplasma gondii Genome resource

Toxoplasma gondii ME49 **(Tg_c)**
Taxonomy:
cellular organisms | Eukaryota | Alveolata | Apicomplexa | Coccidia | Eucoccidiorida | Eimeriorina | Sarcocystidae | Toxoplasma | Toxoplasma gondii
Reference/s:
National Center for Biotechnology Information: NCBI Protozoa genomes
ToxoDB: Toxoplasma gondii Genome resource

Toxoplasma gondii VEG **(Tg_b)**
Taxonomy:
cellular organisms | Eukaryota | Alveolata | Apicomplexa | Coccidia | Eucoccidiorida | Eimeriorina | Sarcocystidae | Toxoplasma | Toxoplasma gondii | Toxoplasma gondii type III
Reference/s:
National Center for Biotechnology Information: NCBI Protozoa genomes
ToxoDB: Toxoplasma gondii Genome resource

Ichthyophthirius multifiliis strain G5 **(Im)**
Taxonomy:
cellular organisms | Eukaryota | Alveolata | Ciliophora | Intramacronucleata | Oligohymenophorea | Hymenostomatida | Ophryoglenina | Ichthyophthirius | Ichthyophthirius multifiliis
Reference/s:
International Species Sequencing Consortium: Ichthyophthirius multifiliis Sequencing Consortium
National Center for Biotechnology Information: NCBI Protozoa genomes
Publication/s:
Coyne RS *et. al.* , *Genome Biol* , **12** , R100 (2011).

Tetrahymena thermophila SB210 **(Tet)**
Taxonomy:
cellular organisms | Eukaryota | Alveolata | Ciliophora | Intramacronucleata | Oligohymenophorea | Hymenostomatida | Tetrahymenina | Tetrahymenidae | Tetrahymena | Tetrahymena thermophila
Reference/s:
Broad Institute of Harvard and MIT: Tetrahymena Comparative Database
International Species Sequencing Consortium: Tetrahymena thermophila Sequencing Consortium
National Center for Biotechnology Information: NCBI Protozoa genomes
TBestDB - Taxonomically Broad EST Database: Tetrahymena thermophila
The Gene Index Project: DFCI Tetrahymena thermophila Gene Index
The Institute for Genomic Research: The Tetrahymena thermophila Genome Sequencing Project
Publication/s:
Eisen JA *et. al.* , *PLoS Biol* , **4** , e286 (2006).

***Paramecium tetraurelia* (Pt)**
Taxonomy:
cellular organisms | Eukaryota | Alveolata | Ciliophora | Intramacronucleata | Oligohymenophorea | Peniculida | Parameciidae | Paramecium
Reference/s:
Genoscope: Paramecium tetraurelia - a model ciliate
National Center for Biotechnology Information: NCBI Protozoa genomes
Publication/s:
Aury JM *et. al.* , *Nature* , **444** , 171 (2006).

***Perkinsus marinus ATCC 50983* (Prm)**
Taxonomy:
cellular organisms | Eukaryota | Alveolata | Perkinsea | Perkinsida | Perkinsidae | Perkinsus | Perkinsus marinus
Reference/s:
National Center for Biotechnology Information: NCBI Protozoa genomes
The Institute for Genomic Research: The Perkinsus marinus Genome Sequencing Project

***Entamoeba histolytica HM-1:IMSS* (Eh)**
Taxonomy:
cellular organisms | Eukaryota | Amoebozoa | Archamoebae | Entamoebidae | Entamoeba
Reference/s:
AmoebaDB: AmoebaDB
International Species Sequencing Consortium: Entamoeba histolytica Sequencing Consortium
National Center for Biotechnology Information: NCBI Protozoa genomes
The Institute for Genomic Research: Entamoeba histolytica Genome Project
The Wellcome Trust Sanger Institute: Entamoeba histolytica Whole Genome Shotgun
Publication/s:
Lorenzi HA *et. al.* , *PLoS Negl Trop Dis* , **4** , e716 (2010).
Loftus B *et. al.* , *Nature* , **2005** , 865 (2005).

***Entamoeba dispar SAW760* (Ed)**
Taxonomy:
cellular organisms | Eukaryota | Amoebozoa | Archamoebae | Entamoebidae | Entamoeba | Entamoeba dispar
Reference/s:
AmoebaDB: AmoebaDB
National Center for Biotechnology Information: NCBI Protozoa genomes

***Entamoeba invadens IP1* (Eti)**
Taxonomy:
cellular organisms | Eukaryota | Amoebozoa | Archamoebae | Entamoebidae | Entamoeba | Entamoeba invadens
Reference/s:
AmoebaDB: AmoebaDB
GenBank - NIH genetic sequence database: GenBank species TBLASTN
National Center for Biotechnology Information: NCBI Protozoa genomes
Publication/s:
Wang Z *et. al.* , *Mol Biochem Parasitol* , **129** , 23 (2003).

***Acanthamoeba castellanii* (Ac)**
Taxonomy:
cellular organisms | Eukaryota | Amoebozoa | Centramoebida | Acanthamoebidae | Acanthamoeba
Reference/s:
GenBank - NIH genetic sequence database: GenBank species TBLASTN WGS
Human Genome Sequencing Center at Baylor College of Medicine: Acanthamoeba castellani Neff
TBestDB - Taxonomically Broad EST Database: Acanthamoeba castellanii

***Dictyostelium fasciculatum* (Dif)**
Taxonomy:
cellular organisms | Eukaryota | Amoebozoa | Mycetozoa | Dictyosteliida | Dictyostelium
Reference/s:
International Species Sequencing Consortium: Dictyostelium Sequencing Consortium
National Center for Biotechnology Information: NCBI Protozoa genomes
Publication/s:
Heidel AJ *et. al.* , *Genome Res* , **21** , 1882 (2011).

***Dictyostelium purpureum* (Dcp)**
Taxonomy:
cellular organisms | Eukaryota | Amoebozoa | Mycetozoa | Dictyosteliida | Dictyostelium
Reference/s:
DOE Joint Genome Institute: Dictyostelium purpureum
International Species Sequencing Consortium: Dictyostelium purpureum Sequencing Consortium
National Center for Biotechnology Information: NCBI Protozoa Genomes Project
dictyBase: Dictyostelium purpureum
Publication/s:
Sucgang R *et. al.* , *Genome Biol* , **12** , R20 (2011).

***Dictyostelium discoideum AX4* (Dd)**
Taxonomy:
cellular organisms | Eukaryota | Amoebozoa | Mycetozoa | Dictyosteliida | Dictyostelium | Dictyostelium discoideum
Reference/s:
Dept. Genome Analysis, IMB Jena: Dictyostelium discoideum Genome Project
Dictyostelium cDNA Project: Dictyostelium cDNA Project

Human Genome Sequencing Center at Baylor College of Medicine: Functional Genomics of Dictyostelium
International Species Sequencing Consortium: Dictyostelium discoideum Sequencing Consortium
National Center for Biotechnology Information: NCBI Protozoa genomes
The Gene Index Project: DFCI Dictyostelium discoideum Gene Index
The Wellcome Trust Sanger Institute: The Dictyostelium discoideum Genome Project
dictyBase: Dictyostelium genome information, curated Dictyostelium literature
e! Ensembl: Dictyostelium discoideum
Publication/s:
Eichinger L *et. al.* , *Nature* , **435** , 43 (2005).
Urushihara H *et. al.* , *Nucleic Acids Res* , **32** , 1647 (2004).
Glockner G *et. al.* , *Nature* , **418** , 79 (2002).
Morio T *et. al.* , *DNA Res* , **5** , 335 (1998).

Polysphondylium pallidum **(Ppp)**
Taxonomy:
cellular organisms | Eukaryota | Amoebozoa | Mycetozoa | Dictyosteliida | Polysphondylium
Reference/s:
International Species Sequencing Consortium: Polysphondylium Sequencing Consortium
National Center for Biotechnology Information: NCBI Protozoa genomes
TBestDB - Taxonomically Broad EST Database: Polysphondylium pallidum
Publication/s:
Heidel AJ *et. al.* , *Genome Res* , **21** , 1882 (2011).

Thecamonas trahens ATCC 50062 **(Tct)**
Taxonomy:
cellular organisms | Eukaryota | Apusozoa | Apusomonadidae | Thecamonas | Thecamonas trahens
Reference/s:
Broad Institute of Harvard and MIT: Origins of Multicellularity Database
National Center for Biotechnology Information: NCBI Protozoa genomes

Guillardia theta **(Gt)**
Taxonomy:
cellular organisms | Eukaryota | Cryptophyta | Pyrenomonadales | Geminigeraceae | Guillardia
Reference/s:
DOE Joint Genome Institute: Guillardia theta CCMP2712 v1.0
National Center for Biotechnology Information: NCBI Eukaryotic Genomes Project
TBestDB - Taxonomically Broad EST Database: Guillardia theta
Publication/s:
Douglas S *et. al.* , *Nature* , **410** , 1091 (2001).

Leishmania infantum JPCM5 **(Lei)**
Taxonomy:
cellular organisms | Eukaryota | Euglenozoa | Kinetoplastida | Trypanosomatidae | Leishmania | Leishmania | Leishmania donovani species complex | Leishmania infantum
Reference/s:
International Species Sequencing Consortium: Leishmania Species Sequencing Consortium
National Center for Biotechnology Information: NCBI Protozoa genomes
The Gene Index Project: DFCI Leishmania sp. Gene Index
The Wellcome Trust Sanger Institute: The Leishmania infantum Genome Project
TriTrypDB - The Kinetoplastid Genome Resource: TriTrypDB
Publication/s:
Peacock CS *et. al.* , *Nat Genet* , **39** , 839 (2007).

Leishmania major str. Friedlin **(Lem)**
Taxonomy:
cellular organisms | Eukaryota | Euglenozoa | Kinetoplastida | Trypanosomatidae | Leishmania | Leishmania | Leishmania major species complex | Leishmania major
Reference/s:
International Species Sequencing Consortium: Leishmania major Sequencing Consortium
National Center for Biotechnology Information: NCBI Protozoa genomes
The Gene Index Project: DFCI Leishmania sp. Gene Index
The Wellcome Trust Sanger Institute: The Leishmania major Friedlin Genome Project
TriTrypDB - The Kinetoplastid Genome Resource: TriTrypDB
Publication/s:
El-Sayed NM *et. al.* , *Science* , **309** , 404 (2005).
Ivens AC *et. al.* , *Science* , **309** , 436 (2005).

Leishmania braziliensis **(Lb)**
Taxonomy:
cellular organisms | Eukaryota | Euglenozoa | Kinetoplastida | Trypanosomatidae | Leishmania | Viannia | Leishmania braziliensis species complex
Reference/s:
International Species Sequencing Consortium: Leishmania Species Sequencing Consortium
National Center for Biotechnology Information: NCBI Protozoa genomes
The Wellcome Trust Sanger Institute: The Leishmania braziliensis Genome Project
TriTrypDB - The Kinetoplastid Genome Resource: TriTrypDB
Publication/s:
Peacock CS *et. al.* , *Nat Genet* , **39** , 839 (2007).

Trypanosoma cruzi CL Brener **(Trc)**
Taxonomy:
cellular organisms | Eukaryota | Euglenozoa | Kinetoplastida | Trypanosomatidae | Trypanosoma | Schizotrypanum
Reference/s:

GenBank - NIH genetic sequence database: GenBank species TBLASTN
International Species Sequencing Consortium: Trypanosoma cruzi Sequencing Consortium
National Center for Biotechnology Information: NCBI Protozoa genomes
The Gene Index Project: DFCI Trypanosoma cruzi Gene Index
The Institute for Genomic Research: The Trypanosoma cruzi Genome Project
TriTrypDB - The Kinetoplastid Genome Resource: TriTrypDB
Publication/s:
El-Sayed NM *et. al.* , *Science* , **309** , 409 (2005).
El-Sayed NM *et. al.* , *Science* , **309** , 404 (2005).
Aguero F *et. al.* , *Mol Biochem Parasitol* , **136** , 221 (2004).
Porcel BM *et. al.* , *Genome Res* , **10** , 1103 (2000).

Trypanosoma brucei brucei strain 927/4 GUTat10.1 **(Tb)**
Taxonomy:
cellular organisms | Eukaryota | Euglenozoa | Kinetoplastida | Trypanosomatidae | Trypanosoma | Trypanozoon | Trypanosoma brucei | Trypanosoma brucei brucei
Reference/s:
International Species Sequencing Consortium: Trypanosoma brucei Sequencing Consortium
National Center for Biotechnology Information: NCBI Protozoa genomes
The Gene Index Project: DFCI Trypanosoma brucei Gene Index
The Institute for Genomic Research: The TIGR Trypanosoma brucei Genome Project
The Wellcome Trust Sanger Institute: The Trypanosoma brucei Genome Project
TriTrypDB - The Kinetoplastid Genome Resource: TriTrypDB
Publication/s:
El-Sayed NM *et. al.* , *Science* , **309** , 404 (2005).
Berriman M *et. al.* , *Science* , **309** , 416 (2005).
Hall N *et. al.* , *Nucleic Acids Res* , **31** , 4864 (2003).
El-Sayed NM *et. al.* , *Nucleic Acids Res* , **31** , 4856 (2003).

Giardia lamblia ATCC 50803 **(Gil)**
Taxonomy:
cellular organisms | Eukaryota | Fornicata | Diplomonadida | Hexamitidae | Giardiinae | Giardia | Giardia intestinalis
Reference/s:
GiardiaDB: The Giardia lamblia Genome Database
International Species Sequencing Consortium: Giardia lamblia Sequencing Consortium
National Center for Biotechnology Information: NCBI Protozoa genomes
Publication/s:
Morrison HG *et. al.* , *Science* , **317** , 1921 (2007).

Cyanophora paradoxa **(Cyp)**
Taxonomy:
cellular organisms | Eukaryota | Glaucocystophyceae | Cyanophoraceae | Cyanophora
Reference/s:
Cyanophora Genome Project: Cyanophora Genome Project
TBestDB - Taxonomically Broad EST Database: Cyanophora paradoxa [Loeffelhardt lab]
TBestDB - Taxonomically Broad EST Database: Cyanophora paradoxa [Durnford lab]

Emiliania huxleyi CCMP1516 **(Emh)**
Taxonomy:
cellular organisms | Eukaryota | Haptophyceae | Isochrysidales | Noelaerhabdaceae | Emiliania | Emiliania huxleyi
Reference/s:
DOE Joint Genome Institute: Emiliania huxleyi

Naegleria gruberi NEG-M **(Ng)**
Taxonomy:
cellular organisms | Eukaryota | Heterolobosea | Schizopyrenida | Vahlkampfiidae | Naegleria
Reference/s:
DOE Joint Genome Institute: Naegleria gruberi
International Species Sequencing Consortium: Naegleria gruberi Sequencing Consortium
National Center for Biotechnology Information: NCBI Protozoa genomes
Publication/s:
Fritz-Laylin LK *et. al.* , *Cell* , **140** , 631 (2010).

Monosiga ovata **(Mo)**
Taxonomy:
cellular organisms | Eukaryota | Opisthokonta | Choanoflagellida | Codonosigidae | Monosiga
Reference/s:
TBestDB - Taxonomically Broad EST Database: Monosiga ovata

Monosiga brevicollis MX1 **(Mb)**
Taxonomy:
cellular organisms | Eukaryota | Opisthokonta | Choanoflagellida | Codonosigidae | Monosiga | Monosiga brevicollis
Reference/s:
Broad Institute of Harvard and MIT: Origins of Multicellularity Database
DOE Joint Genome Institute: Monosiga brevicollis
International Species Sequencing Consortium: Monosiga brevicollis Sequencing Consortium
National Center for Biotechnology Information: NCBI Protozoa genomes
Publication/s:
King N *et. al.* , *Nature* , **451** , 783 (2008).

Proterospongia sp. ATCC 50818 **(Pro)**

Taxonomy:
cellular organisms | Eukaryota | Opisthokonta | Choanoflagellida | Salpingoecidae | Salpingoeca
Reference/s:
Broad Institute of Harvard and MIT: Origins of Multicellularity Database
GenBank - NIH genetic sequence database: GenBank species TBLASTN WGS

***Allomyces macrogynus ATCC 38327* (Alm)**
Taxonomy:
cellular organisms | Eukaryota | Opisthokonta | Fungi | Blastocladiomycota | Blastocladiomycetes | Blastocladiales | Blastocladiaceae | Allomyces | Allomyces macrogynus
Reference/s:
Broad Institute of Harvard and MIT: Origins of Multicellularity Database
GenBank - NIH genetic sequence database: GenBank species TBLASTN WGS
TBestDB - Taxonomically Broad EST Database: Allomyces macrogynus

***Blastocladiella emersonii* (Be)**
Taxonomy:
cellular organisms | Eukaryota | Opisthokonta | Fungi | Blastocladiomycota | Blastocladiomycetes | Blastocladiales | Blastocladiaceae | Blastocladiella
Reference/s:
GenBank - NIH genetic sequence database: GenBank species TBLASTN
Publication/s:
Ribichich KF *et. al.* , *Eukaryot Cell* , **4** , 455 (2005).

***Batrachochytrium dendrobatidis JEL423* (Bad_a)**
Taxonomy:
cellular organisms | Eukaryota | Opisthokonta | Fungi | Chytridiomycota | Chytridiomycetes | Rhizophydiales | Rhizophydiales incertae sedis | Batrachochytrium | Batrachochytrium dendrobatidis
Reference/s:
Broad Institute of Harvard and MIT: Batrachochytrium dendrobatidis Database
GenBank - NIH genetic sequence database: GenBank species TBLASTN WGS
TBestDB - Taxonomically Broad EST Database: Batrachochytrium dendrobatidis

***Batrachochytrium dendrobatidis JAM81* (Bad_b)**
Taxonomy:
cellular organisms | Eukaryota | Opisthokonta | Fungi | Chytridiomycota | Chytridiomycetes | Rhizophydiales | Rhizophydiales incertae sedis | Batrachochytrium | Batrachochytrium dendrobatidis
Reference/s:
DOE Joint Genome Institute: Batrachochytrium dendrobatidis
GenBank - NIH genetic sequence database: GenBank species TBLASTN WGS

***Spizellomyces punctatus DAOM BR117* (Spp)**
Taxonomy:
cellular organisms | Eukaryota | Opisthokonta | Fungi | Chytridiomycota | Chytridiomycetes | Spizellomycetales | Spizellomycetaceae | Spizellomyces | Spizellomyces punctatus
Reference/s:
Broad Institute of Harvard and MIT: Origins of Multicellularity Database
GenBank - NIH genetic sequence database: GenBank species TBLASTN WGS
TBestDB - Taxonomically Broad EST Database: Spizellomyces punctatus

***Pneumocystis carinii* (Puc)**
Taxonomy:
cellular organisms | Eukaryota | Opisthokonta | Fungi | Dikarya | Ascomycota | Taphrinomycotina | Pneumocystidomycetes | Pneumocystidales | Pneumocystidaceae | Pneumocystis

***Schizosaccharomyces cryophilus NRRL Y-48691* (Shc)**
Taxonomy:
cellular organisms | Eukaryota | Opisthokonta | Fungi | Dikarya | Ascomycota | Taphrinomycotina | Schizosaccharomycetes | Schizosaccharomycetales | Schizosaccharomycetaceae | Schizosaccharomyces | Schizosaccharomyces cryophilus
Reference/s:
International Species Sequencing Consortium: Fission yeast Sequencing Consortium
National Center for Biotechnology Information: NCBI Fungi Genomes Project
Publication/s:
Rhind N *et. al.* , *Science* , **332** , 930 (2011).

***Schizosaccharomyces japonicus yFS275* (Sj)**
Taxonomy:
cellular organisms | Eukaryota | Opisthokonta | Fungi | Dikarya | Ascomycota | Taphrinomycotina | Schizosaccharomycetes | Schizosaccharomycetales | Schizosaccharomycetaceae | Schizosaccharomyces | Schizosaccharomyces japonicus
Reference/s:
Broad Institute of Harvard and MIT: Schizosaccharomyces group Database
International Species Sequencing Consortium: Fission yeast Sequencing Consortium
National Center for Biotechnology Information: NCBI Fungi Genomes Project
Publication/s:
Rhind N *et. al.* , *Science* , **332** , 930 (2011).

***Schizosaccharomyces octosporus yFS286* (Sho)**
Taxonomy:
cellular organisms | Eukaryota | Opisthokonta | Fungi | Dikarya | Ascomycota | Taphrinomycotina | Schizosaccharomycetes | Schizosaccharomycetales | Schizosaccharomycetaceae | Schizosaccharomyces | Schizosaccharomyces octosporus
Reference/s:
Broad Institute of Harvard and MIT: Schizosaccharomyces group Database

International Species Sequencing Consortium: Fission yeast Sequencing Consortium
National Center for Biotechnology Information: NCBI Fungi Genomes Project
Publication/s:
Rhind N *et. al.* , *Science* , **332** , 930 (2011).

Schizosaccharomyces pombe 972h- **(Sp)**
Taxonomy:
cellular organisms | Eukaryota | Opisthokonta | Fungi | Dikarya | Ascomycota | Taphrinomycotina | Schizosaccharomycetes | Schizosaccharomycetales | Schizosaccharomycetaceae | Schizosaccharomyces | Schizosaccharomyces pombe
Reference/s:
Ashbya Genome Database: Ashbya Genome Database
GenBank - NIH genetic sequence database: GenBank species TBLASTN
International Species Sequencing Consortium: Schizosaccharomyces pombe Sequencing Consortium
National Center for Biotechnology Information: NCBI Fungi Genomes Project
National Center for Biotechnology Information Reference Sequences: Schizosaccharomyces pombe (fission yeast) genome view
The Gene Index Project: DFCI Schizosaccharomyces pombe Gene Index
The Wellcome Trust Sanger Institute: Schizosaccharomyces pombe Genome Project
e! Ensembl: Schizosaccharomyces pombe
Publication/s:
Kupfer DM *et. al.* , *Eukaryot Cell* , **3** , 1088 (2004).
Wood V *et. al.* , *Nature* , **415** , 871 (2002).

Tuber melanosporum Mel28 **(Tum)**
Taxonomy:
cellular organisms | Eukaryota | Opisthokonta | Fungi | Dikarya | Ascomycota | saccharomyceta | Pezizomycotina | Pezizomycetes | Pezizales | Tuberaceae | Tuber | Tuber melanosporum
Reference/s:
Genoscope: The Black Truffle of Perigord
National Center for Biotechnology Information: NCBI Fungi Genomes Project
Publication/s:
Martin F *et. al.* , *Nature* , **464** , 1033 (2010).

Emericella nidulans FGSC A4 **(En)**
Taxonomy:
cellular organisms | Eukaryota | Opisthokonta | Fungi | Dikarya | Ascomycota | saccharomyceta | Pezizomycotina | leotiomyceta | Eurotiomycetes | Eurotiomycetidae | Eurotiales | Trichocomaceae | Emericella | Emericella nidulans | mitosporic Emericella nidulans
Reference/s:
Broad Institute of Harvard and MIT: Aspergillus Comparative Database
Central Aspergillus Data REpository (CADRE): A. nidulans Genome
GenBank - NIH genetic sequence database: GenBank species TBLASTN
International Species Sequencing Consortium: Emericella nidulans Sequencing Consortium
National Center for Biotechnology Information: NCBI Fungi Genomes Project
The Aspergillus Website: Aspergillus nidulans Genome
The Gene Index Project: DFCI Aspergillus nidulans Gene Index
e! Ensembl: Aspergillus nidulans
Publication/s:
Galagan JE *et. al.* , *Nature* , **438** , 1105 (2005).
Kupfer DM *et. al.* , *Eukaryot Cell* , **3** , 1088 (2004).

Neosartorya fischeri NRRL 181 **(Nef)**
Taxonomy:
cellular organisms | Eukaryota | Opisthokonta | Fungi | Dikarya | Ascomycota | saccharomyceta | Pezizomycotina | leotiomyceta | Eurotiomycetes | Eurotiomycetidae | Eurotiales | Trichocomaceae | Neosartorya | Neosartorya fischeri group | Neosartorya fischeri
Reference/s:
Central Aspergillus Data REpository (CADRE): N. fischeri Genome
International Species Sequencing Consortium: Aspergillus Sequencing Consortium
National Center for Biotechnology Information: NCBI Fungi Genomes Project
The Aspergillus Website: Neosartorya fischeri / Aspergillus fischerianus
The Institute for Genomic Research: Neosartorya fischeri Genome Project
e! Ensembl: Neosartorya fischeri
Publication/s:
Fedorova ND *et. al.* , *PLoS Genet* , **4** , e1000046 (2008).

Talaromyces stipitatus ATCC 10500 **(Tls)**
Taxonomy:
cellular organisms | Eukaryota | Opisthokonta | Fungi | Dikarya | Ascomycota | saccharomyceta | Pezizomycotina | leotiomyceta | Eurotiomycetes | Eurotiomycetidae | Eurotiales | Trichocomaceae | Talaromyces | Talaromyces stipitatus
Reference/s:
National Center for Biotechnology Information: NCBI Fungi Genomes Project

Aspergillus aculeatus ATCC16872 **(Apa)**
Taxonomy:
cellular organisms | Eukaryota | Opisthokonta | Fungi | Dikarya | Ascomycota | saccharomyceta | Pezizomycotina | leotiomyceta | Eurotiomycetes | Eurotiomycetidae | Eurotiales | Trichocomaceae | mitosporic Trichocomaceae | Aspergillus | Aspergillus aculeatus
Reference/s:
DOE Joint Genome Institute: Aspergillus aculeatus ATCC16872 v1.1

Aspergillus carbonarius ITEM 5010 **(Aec)**
Taxonomy:
cellular organisms | Eukaryota | Opisthokonta | Fungi | Dikarya | Ascomycota | saccharomyceta | Pezizomycotina | leotiomyceta | Eurotiomycetes | Eurotiomycetidae |

Eurotiales | Trichocomaceae | mitosporic Trichocomaceae | Aspergillus | Aspergillus carbonarius
Reference/s:
DOE Joint Genome Institute: Aspergillus carbonarius ITEM 5010 v3
DOE Joint Genome Institute: Aspergillus carbonarius

***Aspergillus clavatus NRRL 1* (Asc)**
Taxonomy:
cellular organisms | Eukaryota | Opisthokonta | Fungi | Dikarya | Ascomycota | saccharomyceta | Pezizomycotina | leotiomyceta | Eurotiomycetes | Eurotiomycetidae | Eurotiales | Trichocomaceae | mitosporic Trichocomaceae | Aspergillus | Aspergillus clavatus
Reference/s:
Central Aspergillus Data REpository (CADRE): A. clavatus Genome
International Species Sequencing Consortium: Aspergillus Sequencing Consortium
National Center for Biotechnology Information: NCBI Fungi Genomes Project
The Aspergillus Website: Aspergillus clavatus Genome
The Institute for Genomic Research: Aspergillus clavatus Genome Project
e! Ensembl: Aspergillus clavatus
Publication/s:
Fedorova ND *et. al.* , *PLoS Genet* , **4** , e1000046 (2008).

***Aspergillus flavus NRRL3357* (Af)**
Taxonomy:
cellular organisms | Eukaryota | Opisthokonta | Fungi | Dikarya | Ascomycota | saccharomyceta | Pezizomycotina | leotiomyceta | Eurotiomycetes | Eurotiomycetidae | Eurotiales | Trichocomaceae | mitosporic Trichocomaceae | Aspergillus | Aspergillus flavus
Reference/s:
Center for Integrated Fungal Research: Aspergillus flavus and aflatoxin
Central Aspergillus Data REpository (CADRE): A. flavus Genome
GenBank - NIH genetic sequence database: GenBank species TBLASTN
National Center for Biotechnology Information: NCBI Fungi Genomes Project
The Aspergillus Website: Aspergillus flavus Genome
The Gene Index Project: DFCI Aspergillus flavus Gene Index
e! Ensembl: Aspergillus flavus
Publication/s:
Yu J *et. al.* , *FEMS Microbiol Lett* , **237** , 333 (2004).

***Aspergillus fumigatus Af293* (Asf_a)**
Taxonomy:
cellular organisms | Eukaryota | Opisthokonta | Fungi | Dikarya | Ascomycota | saccharomyceta | Pezizomycotina | leotiomyceta | Eurotiomycetes | Eurotiomycetidae | Eurotiales | Trichocomaceae | mitosporic Trichocomaceae | Aspergillus | Aspergillus fumigatus
Reference/s:
Central Aspergillus Data REpository (CADRE): A. fumigatus Genome
International Species Sequencing Consortium: Aspergillus fumigatus Sequencing Consortium
National Center for Biotechnology Information: NCBI Fungi Genomes Project
The Aspergillus Website: Aspergillus fumigatus AF293 Genomme
The Institute for Genomic Research: Aspergillus fumigatus Genome Project
The Wellcome Trust Sanger Institute: Aspergillus fumigatus Genome Project
e! Ensembl: Aspergillus fumigatus Af293
Publication/s:
Nierman WC *et. al.* , *Nature* , **438** , 1151 (2005).

***Aspergillus fumigatus A1163* (Asf_b)**
Taxonomy:
cellular organisms | Eukaryota | Opisthokonta | Fungi | Dikarya | Ascomycota | saccharomyceta | Pezizomycotina | leotiomyceta | Eurotiomycetes | Eurotiomycetidae | Eurotiales | Trichocomaceae | mitosporic Trichocomaceae | Aspergillus | Aspergillus fumigatus
Reference/s:
Central Aspergillus Data REpository (CADRE): A. fumigatus (A1163) Genome
International Species Sequencing Consortium: Aspergillus fumigatus Sequencing Consortium
National Center for Biotechnology Information: NCBI Fungi Genomes Project
The Aspergillus Website: Aspergillus fumigatus A1163 Genome
e! Ensembl: Aspergillus fumigatus A1163
Publication/s:
Fedorova ND *et. al.* , *PLoS Genet* , **4** , e1000046 (2008).

***Aspergillus niger ATCC 1015* (An_a)**
Taxonomy:
cellular organisms | Eukaryota | Opisthokonta | Fungi | Dikarya | Ascomycota | saccharomyceta | Pezizomycotina | leotiomyceta | Eurotiomycetes | Eurotiomycetidae | Eurotiales | Trichocomaceae | mitosporic Trichocomaceae | Aspergillus | Aspergillus niger
Reference/s:
DOE Joint Genome Institute: Aspergillus niger v3.0
DOE Joint Genome Institute: Aspergillus niger
Fungal Genomics Project: Aspergillus niger
International Species Sequencing Consortium: Aspergillus niger Sequencing Consortium
National Center for Biotechnology Information: NCBI Fungi Genomes Project
The Aspergillus Website: Aspergillus niger Genome
Publication/s:
Andersen MR *et. al.* , *Genome Res* , **21** , 885 (2011).

***Aspergillus niger CBS 513.88* (An_b)**
Taxonomy:
cellular organisms | Eukaryota | Opisthokonta | Fungi | Dikarya | Ascomycota | saccharomyceta | Pezizomycotina | leotiomyceta | Eurotiomycetes | Eurotiomycetidae |

Eurotiales | Trichocomaceae | mitosporic Trichocomaceae | Aspergillus | Aspergillus niger
Reference/s:
Central Aspergillus Data REpository (CADRE): A. niger Genome
International Species Sequencing Consortium: Aspergillus niger Sequencing Consortium
National Center for Biotechnology Information: NCBI Fungi Genomes Project
e! Ensembl: Aspergillus niger
Publication/s:
Pel HJ *et. al.* , *Nat Biotechnol* , **25** , 221 (2007).

Aspergillus oryzae RIB40 **(Ao)**
Taxonomy:
cellular organisms | Eukaryota | Opisthokonta | Fungi | Dikarya | Ascomycota | saccharomyceta | Pezizomycotina | leotiomyceta | Eurotiomycetes | Eurotiomycetidae | Eurotiales | Trichocomaceae | mitosporic Trichocomaceae | Aspergillus | Aspergillus oryzae
Reference/s:
Central Aspergillus Data REpository (CADRE): Aspergillus oryzae Genome
Database of the Genomes Analyzed at NITE: Aspergillus oryzae RIB40
GenBank - NIH genetic sequence database: GenBank species TBLASTN NR
The Aspergillus Website: Aspergillus oryzae Genome
e! Ensembl: Aspergillus oryzae
Publication/s:
Machida M *et. al.* , *Nature* , **438** , 1157 (2005).

Aspergillus terreus NIH2624 **(Ast)**
Taxonomy:
cellular organisms | Eukaryota | Opisthokonta | Fungi | Dikarya | Ascomycota | saccharomyceta | Pezizomycotina | leotiomyceta | Eurotiomycetes | Eurotiomycetidae | Eurotiales | Trichocomaceae | mitosporic Trichocomaceae | Aspergillus | Aspergillus terreus
Reference/s:
Broad Institute of Harvard and MIT: Aspergillus Comparative Database
Central Aspergillus Data REpository (CADRE): A. terreus Genome
National Center for Biotechnology Information: NCBI Fungi Genomes Project
The Aspergillus Website: Aspergillus terreus Genome
e! Ensembl: Aspergillus terreus

Penicillium chrysogenum Wisconsin 54-1255 **(Pch)**
Taxonomy:
cellular organisms | Eukaryota | Opisthokonta | Fungi | Dikarya | Ascomycota | saccharomyceta | Pezizomycotina | leotiomyceta | Eurotiomycetes | Eurotiomycetidae | Eurotiales | Trichocomaceae | mitosporic Trichocomaceae | Penicillium | Penicillium chrysogenum complex | Penicillium chrysogenum
Reference/s:
International Species Sequencing Consortium: Penicillium chrysogenum Sequencing Consortium
National Center for Biotechnology Information: NCBI Fungi Genomes Project
Publication/s:
van den Berg MA *et. al.* , *Nat Biotechnol* , **26** , 1161 (2008).

Penicillium marneffei ATCC 18224 **(Pcm)**
Taxonomy:
cellular organisms | Eukaryota | Opisthokonta | Fungi | Dikarya | Ascomycota | saccharomyceta | Pezizomycotina | leotiomyceta | Eurotiomycetes | Eurotiomycetidae | Eurotiales | Trichocomaceae | mitosporic Trichocomaceae | Penicillium | Penicillium marneffei
Reference/s:
National Center for Biotechnology Information: NCBI Fungi Genomes Project

Ajellomyces capsulatus NAmI WU24 **(Ajc_c)**
Taxonomy:
cellular organisms | Eukaryota | Opisthokonta | Fungi | Dikarya | Ascomycota | saccharomyceta | Pezizomycotina | leotiomyceta | Eurotiomycetes | Eurotiomycetidae | Onygenales | Ajellomycetaceae | Ajellomyces
Reference/s:
Broad Institute of Harvard and MIT: Histoplasma capsulatum Database
International Species Sequencing Consortium: Histoplasma Sequencing Consortium
National Center for Biotechnology Information: NCBI Fungi Genomes Project
Publication/s:
Sharpton TJ *et. al.* , *Genome Res* , **19** , 1722 (2009).

Ajellomyces capsulatus NAmII G217B **(Ajc_b)**
Taxonomy:
cellular organisms | Eukaryota | Opisthokonta | Fungi | Dikarya | Ascomycota | saccharomyceta | Pezizomycotina | leotiomyceta | Eurotiomycetes | Eurotiomycetidae | Onygenales | Ajellomycetaceae | Ajellomyces
Reference/s:
National Center for Biotechnology Information: NCBI Fungi Genomes Project
The Genome Sequencing Center at Washington University: Histoplasma capsulatum Sequencing

Ajellomyces capsulatus NAmII G186AR **(Ajc_a)**
Taxonomy:
cellular organisms | Eukaryota | Opisthokonta | Fungi | Dikarya | Ascomycota | saccharomyceta | Pezizomycotina | leotiomyceta | Eurotiomycetes | Eurotiomycetidae | Onygenales | Ajellomycetaceae | Ajellomyces
Reference/s:
Broad Institute of Harvard and MIT: Histoplasma capsulatum Database
National Center for Biotechnology Information: NCBI Fungi Genomes Project
The Genome Sequencing Center at Washington University: Histoplasma capsulatum Sequencing

Ajellomyces capsulatus H143 **(Ajc_e)**
Taxonomy:

cellular organisms | Eukaryota | Opisthokonta | Fungi | Dikarya | Ascomycota | saccharomyceta | Pezizomycotina | leotiomyceta | Eurotiomycetes | Eurotiomycetidae | Onygenales | Ajellomycetaceae | Ajellomyces | Ajellomyces capsulatus
Reference/s:
Broad Institute of Harvard and MIT: Histoplasma capsulatum Database
National Center for Biotechnology Information: NCBI Fungi Genomes Project

***Ajellomyces capsulatus H88* (Ajc_d)**
Taxonomy:
cellular organisms | Eukaryota | Opisthokonta | Fungi | Dikarya | Ascomycota | saccharomyceta | Pezizomycotina | leotiomyceta | Eurotiomycetes | Eurotiomycetidae | Onygenales | Ajellomycetaceae | Ajellomyces | Ajellomyces capsulatus
Reference/s:
Broad Institute of Harvard and MIT: Histoplasma capsulatum Database
National Center for Biotechnology Information: NCBI Fungi Genomes Project

***Ajellomyces dermatitidis ER-3* (Ajd_a)**
Taxonomy:
cellular organisms | Eukaryota | Opisthokonta | Fungi | Dikarya | Ascomycota | saccharomyceta | Pezizomycotina | leotiomyceta | Eurotiomycetes | Eurotiomycetidae | Onygenales | Ajellomycetaceae | Ajellomyces | Ajellomyces dermatitidis
Reference/s:
Broad Institute of Harvard and MIT: Blastomyces dermatitidis Database
National Center for Biotechnology Information: NCBI Fungi Genomes Project

***Ajellomyces dermatitidis ATCC 18188* (Ajd_c)**
Taxonomy:
cellular organisms | Eukaryota | Opisthokonta | Fungi | Dikarya | Ascomycota | saccharomyceta | Pezizomycotina | leotiomyceta | Eurotiomycetes | Eurotiomycetidae | Onygenales | Ajellomycetaceae | Ajellomyces | Ajellomyces dermatitidis
Reference/s:
Broad Institute of Harvard and MIT: Blastomyces dermatitidis Database
National Center for Biotechnology Information: NCBI Fungi Genomes Project

***Ajellomyces dermatitidis SLH14081* (Ajd_b)**
Taxonomy:
cellular organisms | Eukaryota | Opisthokonta | Fungi | Dikarya | Ascomycota | saccharomyceta | Pezizomycotina | leotiomyceta | Eurotiomycetes | Eurotiomycetidae | Onygenales | Ajellomycetaceae | Ajellomyces | Ajellomyces dermatitidis
Reference/s:
Broad Institute of Harvard and MIT: Blastomyces dermatitidis Database
National Center for Biotechnology Information: NCBI Fungi Genomes Project

***Ajellomyces dermatitidis ATCC 26199* (Ajd_d)**
Taxonomy:
cellular organisms | Eukaryota | Opisthokonta | Fungi | Dikarya | Ascomycota | saccharomyceta | Pezizomycotina | leotiomyceta | Eurotiomycetes | Eurotiomycetidae | Onygenales | Ajellomycetaceae | Ajellomyces | Ajellomyces dermatitidis
Reference/s:
Broad Institute of Harvard and MIT: Blastomyces dermatitidis Database
National Center for Biotechnology Information: NCBI Fungi Genomes Project

***Arthroderma benhamiae CBS 112371* (Arb)**
Taxonomy:
cellular organisms | Eukaryota | Opisthokonta | Fungi | Dikarya | Ascomycota | saccharomyceta | Pezizomycotina | leotiomyceta | Eurotiomycetes | Eurotiomycetidae | Onygenales | Arthrodermataceae | Arthroderma | Arthroderma benhamiae
Reference/s:
Broad Institute of Harvard and MIT: Dermatophyte Comparative Database
National Center for Biotechnology Information: NCBI Fungi Genomes Project

***Arthroderma gypseum CBS 118893* (Arg)**
Taxonomy:
cellular organisms | Eukaryota | Opisthokonta | Fungi | Dikarya | Ascomycota | saccharomyceta | Pezizomycotina | leotiomyceta | Eurotiomycetes | Eurotiomycetidae | Onygenales | Arthrodermataceae | Arthroderma | Arthroderma gypseum
Reference/s:
Broad Institute of Harvard and MIT: Dermatophyte Comparative Database
National Center for Biotechnology Information: NCBI Fungi Genomes Project

***Arthroderma otae CBS 113480* (Aro)**
Taxonomy:
cellular organisms | Eukaryota | Opisthokonta | Fungi | Dikarya | Ascomycota | saccharomyceta | Pezizomycotina | leotiomyceta | Eurotiomycetes | Eurotiomycetidae | Onygenales | Arthrodermataceae | Arthroderma | Arthroderma otae
Reference/s:
Broad Institute of Harvard and MIT: Dermatophyte Comparative Database
National Center for Biotechnology Information: NCBI Fungi Genomes Project

***Trichophyton equinum CBS 127.97* (Te)**
Taxonomy:
cellular organisms | Eukaryota | Opisthokonta | Fungi | Dikarya | Ascomycota | saccharomyceta | Pezizomycotina | leotiomyceta | Eurotiomycetes | Eurotiomycetidae | Onygenales | Arthrodermataceae | mitosporic Arthrodermataceae | Trichophyton | Trichophyton equinum
Reference/s:
Broad Institute of Harvard and MIT: Dermatophyte Comparative Database
National Center for Biotechnology Information: NCBI Fungi Genomes Project

***Trichophyton rubrum CBS 118892* (Trr)**
Taxonomy:

cellular organisms | Eukaryota | Opisthokonta | Fungi | Dikarya | Ascomycota | saccharomyceta | Pezizomycotina | leotiomyceta | Eurotiomycetes | Eurotiomycetidae | Onygenales | Arthrodermataceae | mitosporic Arthrodermataceae | Trichophyton | Trichophyton rubrum
Reference/s:
Broad Institute of Harvard and MIT: Dermatophyte Comparative Database
GenBank - NIH genetic sequence database: GenBank species TBLASTN
National Center for Biotechnology Information: NCBI Fungi Genomes Project
Publication/s:
Wang L *et. al.* , *BMC Genomics* , **7** , 255 (2006).

Trichophyton tonsurans CBS 112818 **(Trt)**
Taxonomy:
cellular organisms | Eukaryota | Opisthokonta | Fungi | Dikarya | Ascomycota | saccharomyceta | Pezizomycotina | leotiomyceta | Eurotiomycetes | Eurotiomycetidae | Onygenales | Arthrodermataceae | mitosporic Arthrodermataceae | Trichophyton | Trichophyton tonsurans
Reference/s:
Broad Institute of Harvard and MIT: Dermatophyte Comparative Database
National Center for Biotechnology Information: NCBI Fungi Genomes Project

Trichophyton verrucosum HKI 0517 **(Thv)**
Taxonomy:
cellular organisms | Eukaryota | Opisthokonta | Fungi | Dikarya | Ascomycota | saccharomyceta | Pezizomycotina | leotiomyceta | Eurotiomycetes | Eurotiomycetidae | Onygenales | Arthrodermataceae | mitosporic Arthrodermataceae | Trichophyton | Trichophyton verrucosum
Reference/s:
Broad Institute of Harvard and MIT: Dermatophyte Comparative Database
National Center for Biotechnology Information: NCBI Fungi Genomes Project

Ascosphaera apis USDA-ARSEF 7405 **(Asa)**
Taxonomy:
cellular organisms | Eukaryota | Opisthokonta | Fungi | Dikarya | Ascomycota | saccharomyceta | Pezizomycotina | leotiomyceta | Eurotiomycetes | Eurotiomycetidae | Onygenales | Ascosphaeraceae | Ascosphaera | Ascosphaera apis
Reference/s:
Human Genome Sequencing Center at Baylor College of Medicine: Ascosphaera apis
National Center for Biotechnology Information: NCBI Fungi Genomes Project
Publication/s:
Qin X *et. al.* , *Insect Mol Biol* , **15** , 715 (2006).

Uncinocarpus reesii 1704 **(Ur)**
Taxonomy:
cellular organisms | Eukaryota | Opisthokonta | Fungi | Dikarya | Ascomycota | saccharomyceta | Pezizomycotina | leotiomyceta | Eurotiomycetes | Eurotiomycetidae | Onygenales | Onygenaceae | Uncinocarpus | Uncinocarpus reesii
Reference/s:
Broad Institute of Harvard and MIT: Uncinocarpus reesii Sequencing Project
International Species Sequencing Consortium: Coccidioides Sequencing Consortium
National Center for Biotechnology Information: NCBI Fungi Genomes Project
Publication/s:
Sharpton TJ *et. al.* , *Genome Res* , **19** , 1722 (2009).

Coccidioides posadasii C735 **(Cop_a)**
Taxonomy:
cellular organisms | Eukaryota | Opisthokonta | Fungi | Dikarya | Ascomycota | saccharomyceta | Pezizomycotina | leotiomyceta | Eurotiomycetes | Eurotiomycetidae | Onygenales | mitosporic Onygenales | Coccidioides
Reference/s:
International Species Sequencing Consortium: Coccidioides Sequencing Consortium
The Gene Index Project: DFCI Coccidioides posadasii Gene Index
The Institute for Genomic Research: Coccidioides posadasii Genome Project
Publication/s:
Neafsey DE *et. al.* , *Genome Res* , **20** , 938 (2010).
Sharpton TJ *et. al.* , *Genome Res* , **19** , 1722 (2009).

Coccidioides immitis H538.4 **(Coi_b)**
Taxonomy:
cellular organisms | Eukaryota | Opisthokonta | Fungi | Dikarya | Ascomycota | saccharomyceta | Pezizomycotina | leotiomyceta | Eurotiomycetes | Eurotiomycetidae | Onygenales | mitosporic Onygenales | Coccidioides | Coccidioides immitis
Reference/s:
Broad Institute of Harvard and MIT: Coccidioides group Database
International Species Sequencing Consortium: Coccidioides Sequencing Consortium
National Center for Biotechnology Information: NCBI Fungi Genomes Project
Publication/s:
Neafsey DE *et. al.* , *Genome Res* , **20** , 938 (2010).

Coccidioides immitis RS **(Coi_a)**
Taxonomy:
cellular organisms | Eukaryota | Opisthokonta | Fungi | Dikarya | Ascomycota | saccharomyceta | Pezizomycotina | leotiomyceta | Eurotiomycetes | Eurotiomycetidae | Onygenales | mitosporic Onygenales | Coccidioides | Coccidioides immitis
Reference/s:
Broad Institute of Harvard and MIT: Coccidioides group Database
International Species Sequencing Consortium: Coccidioides Sequencing Consortium
National Center for Biotechnology Information: NCBI Fungi Genomes Project
Publication/s:
Neafsey DE *et. al.* , *Genome Res* , **20** , 938 (2010).
Sharpton TJ *et. al.* , *Genome Res* , **19** , 1722 (2009).

***Coccidioides immitis RMSCC 3703* (Coi_d)**
Taxonomy:
cellular organisms | Eukaryota | Opisthokonta | Fungi | Dikarya | Ascomycota | saccharomyceta | Pezizomycotina | leotiomyceta | Eurotiomycetes | Eurotiomycetidae | Onygenales | mitosporic Onygenales | Coccidioides | Coccidioides immitis
Reference/s:
Broad Institute of Harvard and MIT: Coccidioides group Database
International Species Sequencing Consortium: Coccidioides Sequencing Consortium
National Center for Biotechnology Information: NCBI Fungi Genomes Project
Publication/s:
Neafsey DE *et. al.* , *Genome Res* , **20** , 938 (2010).

***Coccidioides immitis RMSCC 2394* (Coi_c)**
Taxonomy:
cellular organisms | Eukaryota | Opisthokonta | Fungi | Dikarya | Ascomycota | saccharomyceta | Pezizomycotina | leotiomyceta | Eurotiomycetes | Eurotiomycetidae | Onygenales | mitosporic Onygenales | Coccidioides | Coccidioides immitis
Reference/s:
Broad Institute of Harvard and MIT: Coccidioides group Database
International Species Sequencing Consortium: Coccidioides Sequencing Consortium
National Center for Biotechnology Information: NCBI Fungi Genomes Project
Publication/s:
Neafsey DE *et. al.* , *Genome Res* , **20** , 938 (2010).

***Coccidioides posadasii RMSCC 3488* (Cop_c)**
Taxonomy:
cellular organisms | Eukaryota | Opisthokonta | Fungi | Dikarya | Ascomycota | saccharomyceta | Pezizomycotina | leotiomyceta | Eurotiomycetes | Eurotiomycetidae | Onygenales | mitosporic Onygenales | Coccidioides | Coccidioides posadasii
Reference/s:
Broad Institute of Harvard and MIT: Coccidioides group Database
International Species Sequencing Consortium: Coccidioides Sequencing Consortium
National Center for Biotechnology Information: NCBI Fungi Genomes Project
Publication/s:
Neafsey DE *et. al.* , *Genome Res* , **20** , 938 (2010).

***Coccidioides posadasii CPA 0066* (Cop_k)**
Taxonomy:
cellular organisms | Eukaryota | Opisthokonta | Fungi | Dikarya | Ascomycota | saccharomyceta | Pezizomycotina | leotiomyceta | Eurotiomycetes | Eurotiomycetidae | Onygenales | mitosporic Onygenales | Coccidioides | Coccidioides posadasii
Reference/s:
Broad Institute of Harvard and MIT: Coccidioides group Database
International Species Sequencing Consortium: Coccidioides Sequencing Consortium
National Center for Biotechnology Information: NCBI Fungi Genomes Project
Publication/s:
Neafsey DE *et. al.* , *Genome Res* , **20** , 938 (2010).

***Coccidioides posadasii str. Silveira* (Cop_b)**
Taxonomy:
cellular organisms | Eukaryota | Opisthokonta | Fungi | Dikarya | Ascomycota | saccharomyceta | Pezizomycotina | leotiomyceta | Eurotiomycetes | Eurotiomycetidae | Onygenales | mitosporic Onygenales | Coccidioides | Coccidioides posadasii
Reference/s:
Broad Institute of Harvard and MIT: Coccidioides group Database
International Species Sequencing Consortium: Coccidioides Sequencing Consortium
National Center for Biotechnology Information: NCBI Fungi Genomes Project
Publication/s:
Neafsey DE *et. al.* , *Genome Res* , **20** , 938 (2010).

***Paracoccidioides brasiliensis Pb03* (Pab_a)**
Taxonomy:
cellular organisms | Eukaryota | Opisthokonta | Fungi | Dikarya | Ascomycota | saccharomyceta | Pezizomycotina | leotiomyceta | Eurotiomycetes | Eurotiomycetidae | Onygenales | mitosporic Onygenales | Paracoccidioides | Paracoccidioides brasiliensis
Reference/s:
Broad Institute of Harvard and MIT: Paracoccidioides brasiliensis Database
GenBank - NIH genetic sequence database: GenBank species TBLASTN
International Species Sequencing Consortium: Paracoccidioides Sequencing Consortium
National Center for Biotechnology Information: NCBI Fungi Genomes Project
Publication/s:
Desjardins CA *et. al.* , *PLoS Genet* , **7** , e1002345 (2011).
Felipe MS *et. al.* , *J Biol Chem* , **280** , 24706 (2005).

***Paracoccidioides brasiliensis Pb01* (Pab_b)**
Taxonomy:
cellular organisms | Eukaryota | Opisthokonta | Fungi | Dikarya | Ascomycota | saccharomyceta | Pezizomycotina | leotiomyceta | Eurotiomycetes | Eurotiomycetidae | Onygenales | mitosporic Onygenales | Paracoccidioides | Paracoccidioides brasiliensis
Reference/s:
Broad Institute of Harvard and MIT: Paracoccidioides brasiliensis Database
GenBank - NIH genetic sequence database: GenBank species TBLASTN
International Species Sequencing Consortium: Paracoccidioides Sequencing Consortium
National Center for Biotechnology Information: NCBI Fungi Genomes Project
Publication/s:
Desjardins CA *et. al.* , *PLoS Genet* , **7** , e1002345 (2011).

***Paracoccidioides brasiliensis Pb18* (Pab_c)**
Taxonomy:
cellular organisms | Eukaryota | Opisthokonta | Fungi | Dikarya | Ascomycota | saccharomyceta | Pezizomycotina | leotiomyceta | Eurotiomycetes | Eurotiomycetidae | Onygenales | mitosporic Onygenales | Paracoccidioides | Paracoccidioides brasiliensis
Reference/s:
Broad Institute of Harvard and MIT: Paracoccidioides brasiliensis Database
GenBank - NIH genetic sequence database: GenBank species TBLASTN
International Species Sequencing Consortium: Paracoccidioides Sequencing Consortium
National Center for Biotechnology Information: NCBI Fungi Genomes Project
Publication/s:
Desjardins CA *et. al.* , *PLoS Genet* , **7** , e1002345 (2011).

***Mycosphaerella fijiensis* (Myf)**
Taxonomy:
cellular organisms | Eukaryota | Opisthokonta | Fungi | Dikarya | Ascomycota | saccharomyceta | Pezizomycotina | leotiomyceta | dothideomyceta | Dothideomycetes | Dothideomycetidae | Capnodiales | Mycosphaerellaceae | Mycosphaerella
Reference/s:
DOE Joint Genome Institute: Mycosphaerella fijiensis v2.0
DOE Joint Genome Institute: Mycosphaerella fijiensis

***Mycosphaerella pini NZE10* (Mcp)**
Taxonomy:
cellular organisms | Eukaryota | Opisthokonta | Fungi | Dikarya | Ascomycota | saccharomyceta | Pezizomycotina | leotiomyceta | dothideomyceta | Dothideomycetes | Dothideomycetidae | Capnodiales | Mycosphaerellaceae | Mycosphaerella
Reference/s:
DOE Joint Genome Institute: Dothistroma septosporum NZE10 v1.0

***Mycosphaerella populorum SO2202* (Msp)**
Taxonomy:
cellular organisms | Eukaryota | Opisthokonta | Fungi | Dikarya | Ascomycota | saccharomyceta | Pezizomycotina | leotiomyceta | dothideomyceta | Dothideomycetes | Dothideomycetidae | Capnodiales | Mycosphaerellaceae | Mycosphaerella | Mycosphaerella populorum
Reference/s:
DOE Joint Genome Institute: Septoria musiva SO2202 v1.0

***Mycosphaerella graminicola IPO323* (Mg)**
Taxonomy:
cellular organisms | Eukaryota | Opisthokonta | Fungi | Dikarya | Ascomycota | saccharomyceta | Pezizomycotina | leotiomyceta | dothideomyceta | Dothideomycetes | Dothideomycetidae | Capnodiales | Mycosphaerellaceae | Zymoseptoria | Zymoseptoria tritici
Reference/s:
DOE Joint Genome Institute: Mycosphaerella graminicola
DOE Joint Genome Institute: Mycosphaerella graminicola v2.0
GenBank - NIH genetic sequence database: GenBank species TBLASTN
International Species Sequencing Consortium: Mycosphaerella Sequencing Consortium
International Species Sequencing Consortium: Mycosphaerella graminicola Sequencing Consortium
National Center for Biotechnology Information: NCBI Fungi Genomes Project
Publication/s:
Goodwin SB *et. al.* , *PLoS Genet* , **7** , e1002070 (2011).
Stukenbrock EH *et. al.* , *PLoS Genet* , **6** , e1001189 (2010).
Keon J *et. al.* , *Fungal Genet Biol* , **42** , 376 (2005).

***Aureobasidium pullulans* (Aup)**
Taxonomy:
cellular organisms | Eukaryota | Opisthokonta | Fungi | Dikarya | Ascomycota | saccharomyceta | Pezizomycotina | leotiomyceta | dothideomyceta | Dothideomycetes | Dothideomycetidae | Dothideales | Dothioraceae | mitosporic Dothioraceae | Aureobasidium
Reference/s:
Fungal Genomics Project: Aureobasidium pullulans

***Phaeosphaeria nodorum SN15* (Pn)**
Taxonomy:
cellular organisms | Eukaryota | Opisthokonta | Fungi | Dikarya | Ascomycota | saccharomyceta | Pezizomycotina | leotiomyceta | dothideomyceta | Dothideomycetes | Pleosporomycetidae | Pleosporales | Pleosporineae | Phaeosphaeriaceae | Phaeosphaeria | Phaeosphaeria nodorum
Reference/s:
Broad Institute of Harvard and MIT: Stagonospora nodorum Database
DOE Joint Genome Institute: Stagonospora nodorum SN15
International Species Sequencing Consortium: Stagonospora Sequencing Consortium
National Center for Biotechnology Information: NCBI Fungi Genomes Project
Publication/s:
Hane JK *et. al.* , *Plant Cell* , **19** , 3347 (2007).

***Cochliobolus heterostrophus C5* (Coh)**
Taxonomy:
cellular organisms | Eukaryota | Opisthokonta | Fungi | Dikarya | Ascomycota | saccharomyceta | Pezizomycotina | leotiomyceta | dothideomyceta | Dothideomycetes | Pleosporomycetidae | Pleosporales | Pleosporineae | Pleosporaceae | Cochliobolus | Cochliobolus heterostrophus
Reference/s:
DOE Joint Genome Institute: Cochliobolus heterostrophus C5 v2.0
DOE Joint Genome Institute: Cochliobolus heterostrophus

***Pyrenophora teres f. teres 0-1* (Pyt)**
Taxonomy:
cellular organisms | Eukaryota | Opisthokonta | Fungi | Dikarya | Ascomycota | saccharomyceta | Pezizomycotina | leotiomyceta | dothideomyceta | Dothideomycetes |

Pleosporomycetidae | Pleosporales | Pleosporineae | Pleosporaceae | Pyrenophora | Pyrenophora teres | Pyrenophora teres f. teres
Reference/s:
International Species Sequencing Consortium: Pyrenophora Sequencing Consortium
National Center for Biotechnology Information: NCBI Fungi Genomes Project
Publication/s:
Ellwood SR *et. al.* , *Genome Biol* , **11** , R109 (2010).

Pyrenophora tritici-repentis Pt-1C-BFP **(Ptr)**
Taxonomy:
cellular organisms | Eukaryota | Opisthokonta | Fungi | Dikarya | Ascomycota | saccharomyceta | Pezizomycotina | leotiomyceta | dothideomyceta | Dothideomycetes | Pleosporomycetidae | Pleosporales | Pleosporineae | Pleosporaceae | Pyrenophora | Pyrenophora tritici-repentis
Reference/s:
Broad Institute of Harvard and MIT: Pyrenophora tritici-repentis Database
DOE Joint Genome Institute: Pyrenophora tritici-repentis
National Center for Biotechnology Information: NCBI Fungi Genomes Project

Alternaria brassicicola ATCC 96866 **(Alb)**
Taxonomy:
cellular organisms | Eukaryota | Opisthokonta | Fungi | Dikarya | Ascomycota | saccharomyceta | Pezizomycotina | leotiomyceta | dothideomyceta | Dothideomycetes | Pleosporomycetidae | Pleosporales | Pleosporineae | Pleosporaceae | mitosporic Pleosporaceae | Alternaria
Reference/s:
DOE Joint Genome Institute: Alternaria brassicicola
National Center for Biotechnology Information: NCBI Fungi Genomes Project
The Genome Sequencing Center at Washington University: Alternaria brassicicola

Blumeria graminis f. sp. hordei DH14 **(Bg)**
Taxonomy:
cellular organisms | Eukaryota | Opisthokonta | Fungi | Dikarya | Ascomycota | saccharomyceta | Pezizomycotina | leotiomyceta | sordariomyceta | Leotiomycetes | Erysiphales | Erysiphaceae | Blumeria | Blumeria graminis | Blumeria graminis f. sp. hordei
Reference/s:
International Species Sequencing Consortium: Blumeria graminis Sequencing Consortium
National Center for Biotechnology Information: NCBI Fungi Genomes Project
Publication/s:
Spanu PD *et. al.* , *Science* , **330** , 1543 (2010).

Botryotinia fuckeliana B05.10 **(Bof)**
Taxonomy:
cellular organisms | Eukaryota | Opisthokonta | Fungi | Dikarya | Ascomycota | saccharomyceta | Pezizomycotina | leotiomyceta | sordariomyceta | Leotiomycetes | Helotiales | Sclerotiniaceae | Botryotinia | Botryotinia fuckeliana
Reference/s:
Broad Institute of Harvard and MIT: Botrytis cinerea Database
International Species Sequencing Consortium: Botrytis cinerea Sequencing Consortium
National Center for Biotechnology Information: NCBI Fungi Genomes Project
Publication/s:
Amselem J *et. al.* , *PLoS Genet* , **7** , e1002230 (2011).

Sclerotinia sclerotiorum 1980 **(Scs)**
Taxonomy:
cellular organisms | Eukaryota | Opisthokonta | Fungi | Dikarya | Ascomycota | saccharomyceta | Pezizomycotina | leotiomyceta | sordariomyceta | Leotiomycetes | Helotiales | Sclerotiniaceae | Sclerotinia | Sclerotinia sclerotiorum
Reference/s:
Broad Institute of Harvard and MIT: Sclerotinia sclerotiorum Sequencing Project
GenBank - NIH genetic sequence database: GenBank species TBLASTN
International Species Sequencing Consortium: Sclerotinia sclerotiorum Sequencing Consortium
National Center for Biotechnology Information: NCBI Fungi Genomes Project
Publication/s:
Amselem J *et. al.* , *PLoS Genet* , **7** , e1002230 (2011).
Li R *et. al.* , *Fungal Genet Biol* , **41** , 735 (2004).

Geomyces pannorum **(Gep)**
Taxonomy:
cellular organisms | Eukaryota | Opisthokonta | Fungi | Dikarya | Ascomycota | saccharomyceta | Pezizomycotina | leotiomyceta | sordariomyceta | Leotiomycetes | Leotiomycetes incertae sedis | Myxotrichaceae | mitosporic Myxotrichaceae | Geomyces
Reference/s:
Fungal Genomics Project: Geomyces pannorum

Geomyces destructans 20631-21 **(Ged)**
Taxonomy:
cellular organisms | Eukaryota | Opisthokonta | Fungi | Dikarya | Ascomycota | saccharomyceta | Pezizomycotina | leotiomyceta | sordariomyceta | Leotiomycetes | Leotiomycetes incertae sedis | Myxotrichaceae | mitosporic Myxotrichaceae | Geomyces | Geomyces destructans
Reference/s:
Broad Institute of Harvard and MIT: Geomyces destructans Database
National Center for Biotechnology Information: NCBI Fungi Genomes Project

Glomerella graminicola **(Glg)**
Taxonomy:
cellular organisms | Eukaryota | Opisthokonta | Fungi | Dikarya | Ascomycota | saccharomyceta | Pezizomycotina | leotiomyceta | sordariomyceta | Sordariomycetes | Hypocreomycetidae | Glomerellales | Glomerellaceae | Glomerella
Reference/s:
Broad Institute of Harvard and MIT: Colletotrichum graminicola Database

National Center for Biotechnology Information: NCBI Fungi Genomes Project

***Verticillium albo-atrum VaMs.102* (Va)**
Taxonomy:
cellular organisms | Eukaryota | Opisthokonta | Fungi | Dikarya | Ascomycota | saccharomyceta | Pezizomycotina | leotiomyceta | sordariomyceta | Sordariomycetes | Hypocreomycetidae | Glomerellales | Plectosphaerellaceae | mitosporic Plectosphaerellaceae | Verticillium | Verticillium albo-atrum
Reference/s:
Broad Institute of Harvard and MIT: Verticillium group Database
National Center for Biotechnology Information: NCBI Fungi Genomes Project

***Verticillium dahliae VdLs.17* (Vd)**
Taxonomy:
cellular organisms | Eukaryota | Opisthokonta | Fungi | Dikarya | Ascomycota | saccharomyceta | Pezizomycotina | leotiomyceta | sordariomyceta | Sordariomycetes | Hypocreomycetidae | Glomerellales | Plectosphaerellaceae | mitosporic Plectosphaerellaceae | Verticillium | Verticillium dahliae
Reference/s:
Broad Institute of Harvard and MIT: Verticillium group Database
National Center for Biotechnology Information: NCBI Fungi Genomes Project

***Epichloe festucae E2368* (Ecf)**
Taxonomy:
cellular organisms | Eukaryota | Opisthokonta | Fungi | Dikarya | Ascomycota | saccharomyceta | Pezizomycotina | leotiomyceta | sordariomyceta | Sordariomycetes | Hypocreomycetidae | Hypocreales | Clavicipitaceae | Epichloe | Epichloe festucae
Reference/s:
National Center for Biotechnology Information: NCBI Fungi Genomes Project
University of Oklahoma's Advanced Center for Genome Technology: P. omnivora and E. festucae Genomic Sequencing

***Metarhizium anisopliae ARSEF 23* (Mra)**
Taxonomy:
cellular organisms | Eukaryota | Opisthokonta | Fungi | Dikarya | Ascomycota | saccharomyceta | Pezizomycotina | leotiomyceta | sordariomyceta | Sordariomycetes | Hypocreomycetidae | Hypocreales | Clavicipitaceae | mitosporic Clavicipitaceae | Metarhizium | Metarhizium anisopliae
Reference/s:
International Species Sequencing Consortium: Metarhizium Sequencing Consortium
National Center for Biotechnology Information: NCBI Fungi Genomes Project
Publication/s:
Gao Q *et. al.* , *PLoS Genet* , **7** , e1001264 (2011).

***Cordyceps bassiana* (Cob)**
Taxonomy:
cellular organisms | Eukaryota | Opisthokonta | Fungi | Dikarya | Ascomycota | saccharomyceta | Pezizomycotina | leotiomyceta | sordariomyceta | Sordariomycetes | Hypocreomycetidae | Hypocreales | Cordycipitaceae | Cordyceps

***Hypocrea virens Gv29-8* (Hpv)**
Taxonomy:
cellular organisms | Eukaryota | Opisthokonta | Fungi | Dikarya | Ascomycota | saccharomyceta | Pezizomycotina | leotiomyceta | sordariomyceta | Sordariomycetes | Hypocreomycetidae | Hypocreales | Hypocreaceae | Hypocrea
Reference/s:
DOE Joint Genome Institute: Trichoderma virens
DOE Joint Genome Institute: Trichoderma virens Gv29-8 v2.0
International Species Sequencing Consortium: Trichoderma Sequencing Consortium
National Center for Biotechnology Information: NCBI Fungi Genomes Project
Publication/s:
Kubicek CP *et. al.* , *Genome Biol* , **12** , R40 (2011).

***Hypocrea jecorina QM6a* (Hj)**
Taxonomy:
cellular organisms | Eukaryota | Opisthokonta | Fungi | Dikarya | Ascomycota | saccharomyceta | Pezizomycotina | leotiomyceta | sordariomyceta | Sordariomycetes | Hypocreomycetidae | Hypocreales | Hypocreaceae | Hypocrea | Hypocrea jecorina
Reference/s:
DOE Joint Genome Institute: Trichoderma reesei
GenBank - NIH genetic sequence database: GenBank species TBLASTN
International Species Sequencing Consortium: Hypocrea jecorina Sequencing Consortium
National Center for Biotechnology Information: NCBI Fungi Genomes Project
Publication/s:
Martinez D *et. al.* , *Nat Biotechnol* , **26** , 553 (2008).
Diener SE *et. al.* , *FEMS Microbiol Lett* , **230** , 275 (2004).
Foreman PK *et. al.* , *J Biol Chem* , **278** , 31988 (2003).

***Trichoderma asperellum CECT 20268* (Tca_a)**
Taxonomy:
cellular organisms | Eukaryota | Opisthokonta | Fungi | Dikarya | Ascomycota | saccharomyceta | Pezizomycotina | leotiomyceta | sordariomyceta | Sordariomycetes | Hypocreomycetidae | Hypocreales | Hypocreaceae | Hypocrea | mitosporic Hypocrea | Trichoderma

***Trichoderma atroviride IMI 206040* (Tra)**
Taxonomy:
cellular organisms | Eukaryota | Opisthokonta | Fungi | Dikarya | Ascomycota | saccharomyceta | Pezizomycotina | leotiomyceta | sordariomyceta | Sordariomycetes | Hypocreomycetidae | Hypocreales | Hypocreaceae | Hypocrea | mitosporic Hypocrea | Trichoderma | Trichoderma atroviride
Reference/s:
DOE Joint Genome Institute: Trichoderma atroviride
DOE Joint Genome Institute: Trichoderma atroviride v2.0
International Species Sequencing Consortium: Trichoderma Sequencing Consortium

National Center for Biotechnology Information: NCBI Fungi Genomes Project
Publication/s:
Kubicek CP *et. al.* , *Genome Biol* , **12** , R40 (2011).

Gibberella moniliformis 7600 **(Gim)**
Taxonomy:
cellular organisms | Eukaryota | Opisthokonta | Fungi | Dikarya | Ascomycota | saccharomyceta | Pezizomycotina | leotiomyceta | sordariomyceta | Sordariomycetes | Hypocreomycetidae | Hypocreales | Nectriaceae | Gibberella | Gibberella moniliformis
Reference/s:
Broad Institute of Harvard and MIT: Fusarium Comparative Database
GenBank - NIH genetic sequence database: GenBank species TBLASTN
International Species Sequencing Consortium: Fusarium Sequencing Consortium
National Center for Biotechnology Information: NCBI Fungi Genomes Project
The Gene Index Project: DFCI Fusarium verticillioides Gene Index
Publication/s:
Ma LJ *et. al.* , *Nature* , **464** , 367 (2010).
Brown DW *et. al.* , *Fungal Genet Biol* , **42** , 848 (2005).

Gibberella zeae PH-1 **(Gz)**
Taxonomy:
cellular organisms | Eukaryota | Opisthokonta | Fungi | Dikarya | Ascomycota | saccharomyceta | Pezizomycotina | leotiomyceta | sordariomyceta | Sordariomycetes | Hypocreomycetidae | Hypocreales | Nectriaceae | Gibberella | Gibberella zeae
Reference/s:
Broad Institute of Harvard and MIT: Fusarium Comparative Database
International Species Sequencing Consortium: Fusarium graminearum Sequencing Consortium
National Center for Biotechnology Information: NCBI Fungi Genomes Project
Publication/s:
Cuomo CA *et. al.* , *Science* , **317** , 1400 (2007).

Nectria haematococca MPVI **(Nh)**
Taxonomy:
cellular organisms | Eukaryota | Opisthokonta | Fungi | Dikarya | Ascomycota | saccharomyceta | Pezizomycotina | leotiomyceta | sordariomyceta | Sordariomycetes | Hypocreomycetidae | Hypocreales | Nectriaceae | Nectria | Nectria haematococca complex | Nectria haematococca
Reference/s:
DOE Joint Genome Institute: Nectria haematococca v2.0
DOE Joint Genome Institute: Nectria haematococca
International Species Sequencing Consortium: Nectria haematococca Sequencing Consortium
National Center for Biotechnology Information: NCBI Fungi Genomes Project
Publication/s:
Coleman JJ *et. al.* , *PLoS Genet* , **5** , e1000618 (2009).

Fusarium sporotrichioides **(Fus)**
Taxonomy:
cellular organisms | Eukaryota | Opisthokonta | Fungi | Dikarya | Ascomycota | saccharomyceta | Pezizomycotina | leotiomyceta | sordariomyceta | Sordariomycetes | Hypocreomycetidae | Hypocreales | mitosporic Hypocreales | Fusarium

Fusarium oxysporum f. sp. lycopersici 4287 **(Fo)**
Taxonomy:
cellular organisms | Eukaryota | Opisthokonta | Fungi | Dikarya | Ascomycota | saccharomyceta | Pezizomycotina | leotiomyceta | sordariomyceta | Sordariomycetes | Hypocreomycetidae | Hypocreales | mitosporic Hypocreales | Fusarium | Fusarium oxysporum species complex | Fusarium oxysporum | Fusarium oxysporum f. sp. lycopersici
Reference/s:
Broad Institute of Harvard and MIT: Fusarium Comparative Database
International Species Sequencing Consortium: Fusarium Sequencing Consortium
National Center for Biotechnology Information: NCBI Fungi Genomes Project
Publication/s:
Ma LJ *et. al.* , *Nature* , **464** , 367 (2010).

Cryphonectria parasitica **(Crp)**
Taxonomy:
cellular organisms | Eukaryota | Opisthokonta | Fungi | Dikarya | Ascomycota | saccharomyceta | Pezizomycotina | leotiomyceta | sordariomyceta | Sordariomycetes | Sordariomycetidae | Diaporthales | Cryphonectriaceae | Cryphonectria-Endothia complex | Cryphonectria
Reference/s:
DOE Joint Genome Institute: Cryphonectria parasitica EP155 v2.0
DOE Joint Genome Institute: Cryphonectria parasitica

Gaeumannomyces graminis var. tritici R3-111a-1 **(Ggt)**
Taxonomy:
cellular organisms | Eukaryota | Opisthokonta | Fungi | Dikarya | Ascomycota | saccharomyceta | Pezizomycotina | leotiomyceta | sordariomyceta | Sordariomycetes | Sordariomycetidae | Magnaporthales | Magnaporthaceae | Gaeumannomyces | Gaeumannomyces graminis | Gaeumannomyces graminis var. tritici
Reference/s:
Broad Institute of Harvard and MIT: Magnaporthe comparative Database
National Center for Biotechnology Information: NCBI Fungi Genomes Project

Magnaporthe grisea 70-15 **(Mag)**
Taxonomy:
cellular organisms | Eukaryota | Opisthokonta | Fungi | Dikarya | Ascomycota | saccharomyceta | Pezizomycotina | leotiomyceta | sordariomyceta | Sordariomycetes | Sordariomycetidae | Magnaporthales | Magnaporthaceae | Magnaporthe | Magnaporthe oryzae
Reference/s:
Broad Institute of Harvard and MIT: Magnaporthe comparative Database

GenBank - NIH genetic sequence database: GenBank species TBLASTN
International Species Sequencing Consortium: Magnaporthe grisea Sequencing Consortium
National Center for Biotechnology Information: NCBI Fungi Genomes Project
The Gene Index Project: DFCI Magnaporthe grisea Gene Index
Publication/s:
Dean RA *et. al.* , *Nature* , **434** , 980 (2005).
Ebbole DJ *et. al.* , *Mol Plant Microbe Interact* , **17** , 1337 (2004).

Magnaporthe poae ATCC 64411 **(Map)**
Taxonomy:
cellular organisms | Eukaryota | Opisthokonta | Fungi | Dikarya | Ascomycota | saccharomyceta | Pezizomycotina | leotiomyceta | sordariomyceta | Sordariomycetes | Sordariomycetidae | Magnaporthales | Magnaporthaceae | Magnaporthe | Magnaporthe poae
Reference/s:
Broad Institute of Harvard and MIT: Magnaporthe comparative Database
National Center for Biotechnology Information: NCBI Fungi Genomes Project

Grosmannia clavigera kw1407 **(Grc)**
Taxonomy:
cellular organisms | Eukaryota | Opisthokonta | Fungi | Dikarya | Ascomycota | saccharomyceta | Pezizomycotina | leotiomyceta | sordariomyceta | Sordariomycetes | Sordariomycetidae | Ophiostomatales | Ophiostomataceae | Grosmannia | Grosmannia clavigera
Reference/s:
International Species Sequencing Consortium: Grosmania Sequencing Consortium
National Center for Biotechnology Information: NCBI Fungi Genomes Project
Publication/s:
Diguistini S *et. al.* , *Genome Biol* , **10** , R94 (2009).

Chaetomium globosum CBS 148.51 **(Chg)**
Taxonomy:
cellular organisms | Eukaryota | Opisthokonta | Fungi | Dikarya | Ascomycota | saccharomyceta | Pezizomycotina | leotiomyceta | sordariomyceta | Sordariomycetes | Sordariomycetidae | Sordariales | Chaetomiaceae | Chaetomium | Chaetomium globosum
Reference/s:
Broad Institute of Harvard and MIT: Chaetomium globosum Sequencing Project
DOE Joint Genome Institute: Chaetomium globosum
National Center for Biotechnology Information: NCBI Fungi Genomes Project

Thielavia terrestris NRRL 8126 **(Tit)**
Taxonomy:
cellular organisms | Eukaryota | Opisthokonta | Fungi | Dikarya | Ascomycota | saccharomyceta | Pezizomycotina | leotiomyceta | sordariomyceta | Sordariomycetes | Sordariomycetidae | Sordariales | Chaetomiaceae | Thielavia | Thielavia terrestris
Reference/s:
DOE Joint Genome Institute: Thielavia terrestris
GenBank - NIH genetic sequence database: GenBank species TBLASTN WGS
International Species Sequencing Consortium: Thielavia Sequencing Consortium
Publication/s:
Berka RM *et. al.* , *Nat Biotechnol* , **29** , 922 (2011).

Thielavia heterothallica ATCC 42464 **(Th)**
Taxonomy:
cellular organisms | Eukaryota | Opisthokonta | Fungi | Dikarya | Ascomycota | saccharomyceta | Pezizomycotina | leotiomyceta | sordariomyceta | Sordariomycetes | Sordariomycetidae | Sordariales | Chaetomiaceae | mitosporic Chaetomiaceae | Myceliophthora
Reference/s:
DOE Joint Genome Institute: Sporotrichum thermophile
GenBank - NIH genetic sequence database: GenBank species TBLASTN WGS
International Species Sequencing Consortium: Thielavia Sequencing Consortium
Publication/s:
Berka RM *et. al.* , *Nat Biotechnol* , **29** , 922 (2011).

Podospora anserina **(Poa)**
Taxonomy:
cellular organisms | Eukaryota | Opisthokonta | Fungi | Dikarya | Ascomycota | saccharomyceta | Pezizomycotina | leotiomyceta | sordariomyceta | Sordariomycetes | Sordariomycetidae | Sordariales | Lasiosphaeriaceae | Podospora
Reference/s:
Genoscope: Podospora anserina Genome Project
International Species Sequencing Consortium: Podospora anserina Sequencing Consortium
National Center for Biotechnology Information: NCBI Fungi Genomes Project
Publication/s:
Espagne E *et. al.* , *Genome Biol* , **9** , R77 (2008).

Neurospora crassa OR74A **(Nc)**
Taxonomy:
cellular organisms | Eukaryota | Opisthokonta | Fungi | Dikarya | Ascomycota | saccharomyceta | Pezizomycotina | leotiomyceta | sordariomyceta | Sordariomycetes | Sordariomycetidae | Sordariales | Sordariaceae | Neurospora | Neurospora crassa
Reference/s:
Ashbya Genome Database: Ashbya Genome Database
Broad Institute of Harvard and MIT: Neurospora crassa Sequencing Project
GenBank - NIH genetic sequence database: GenBank species TBLASTN
International Species Sequencing Consortium: Neurospora crassa Sequencing Consortium
National Center for Biotechnology Information: NCBI Fungi Genomes Project
The Gene Index Project: DFCI Neurospora crassa Gene Index
e! Ensembl: Neurospora crassa

Publication/s:
Kupfer DM *et. al.* , *Eukaryot Cell* , **3** , 1088 (2004).
Galagan JE *et. al.* , *Nature* , **422** , 859 (2003).

Neurospora discreta FGSC 8579 **(Ned)**
Taxonomy:
cellular organisms | Eukaryota | Opisthokonta | Fungi | Dikarya | Ascomycota | saccharomyceta | Pezizomycotina | leotiomyceta | sordariomyceta | Sordariomycetes | Sordariomycetidae | Sordariales | Sordariaceae | Neurospora | Neurospora discreta
Reference/s:
DOE Joint Genome Institute: Neurospora discreta

Neurospora tetrasperma FGSC 2508 **(Net)**
Taxonomy:
cellular organisms | Eukaryota | Opisthokonta | Fungi | Dikarya | Ascomycota | saccharomyceta | Pezizomycotina | leotiomyceta | sordariomyceta | Sordariomycetes | Sordariomycetidae | Sordariales | Sordariaceae | Neurospora | Neurospora tetrasperma
Reference/s:
DOE Joint Genome Institute: Neurospora tetrasperma
DOE Joint Genome Institute: Neurospora tetrasperma FGSC 2508 mat A v2.0
International Species Sequencing Consortium: Neurospora tetrasperma Sequencing Consortium
National Center for Biotechnology Information: NCBI Fungi Genomes Project

Sordaria macrospora **(Som)**
Taxonomy:
cellular organisms | Eukaryota | Opisthokonta | Fungi | Dikarya | Ascomycota | saccharomyceta | Pezizomycotina | leotiomyceta | sordariomyceta | Sordariomycetes | Sordariomycetidae | Sordariales | Sordariaceae | Sordaria
Reference/s:
International Species Sequencing Consortium: Sordaria Sequencing Consortium
National Center for Biotechnology Information: NCBI Fungi Genomes Project
Publication/s:
Nowrousian M *et. al.* , *PLoS Genet* , **6** , e1000891 (2010).

Debaryomyces hansenii CBS767 **(Deh)**
Taxonomy:
cellular organisms | Eukaryota | Opisthokonta | Fungi | Dikarya | Ascomycota | saccharomyceta | Saccharomycotina | Saccharomycetes | Saccharomycetales | Debaryomycetaceae | Debaryomyces | Debaryomyces hansenii | Debaryomyces hansenii var. hansenii
Reference/s:
Genolevures: Genomic Exploration of the Hemiascomycete Yeasts
International Species Sequencing Consortium: Debaryomyces hansenii Sequencing Consortium
National Center for Biotechnology Information: NCBI Fungi Genomes Project
National Center for Biotechnology Information Reference Sequences: Debaryomyces hansenii genome view
Publication/s:
Butler G *et. al.* , *Nature* , **459** , 657 (2009).
Dujon B *et. al.* , *Nature* , **430** , 35 (2004).
Lepingle A *et. al.* , *FEBS Lett* , **487** , 82 (2000).

Lodderomyces elongisporus NRRL YB-4239 **(Loe)**
Taxonomy:
cellular organisms | Eukaryota | Opisthokonta | Fungi | Dikarya | Ascomycota | saccharomyceta | Saccharomycotina | Saccharomycetes | Saccharomycetales | Debaryomycetaceae | Lodderomyces | Lodderomyces elongisporus
Reference/s:
Broad Institute of Harvard and MIT: Lodderomyces elongisporus Database
International Species Sequencing Consortium: Candida Sequencing Consortium
National Center for Biotechnology Information: NCBI Fungi Genomes Project
Publication/s:
Butler G *et. al.* , *Nature* , **459** , 657 (2009).

Meyerozyma guilliermondii ATCC 6260 **(Mrg)**
Taxonomy:
cellular organisms | Eukaryota | Opisthokonta | Fungi | Dikarya | Ascomycota | saccharomyceta | Saccharomycotina | Saccharomycetes | Saccharomycetales | Debaryomycetaceae | Meyerozyma | Meyerozyma guilliermondii
Reference/s:
Broad Institute of Harvard and MIT: Candida guilliermondii Sequencing Project
International Species Sequencing Consortium: Pichia guilliermondii Sequencing Consortium
National Center for Biotechnology Information: NCBI Fungi Genomes Project
Publication/s:
Butler G *et. al.* , *Nature* , **459** , 657 (2009).

Millerozyma farinosa CBS7064 **(Mif)**
Taxonomy:
cellular organisms | Eukaryota | Opisthokonta | Fungi | Dikarya | Ascomycota | saccharomyceta | Saccharomycotina | Saccharomycetes | Saccharomycetales | Debaryomycetaceae | Millerozyma
Reference/s:
GenBank - NIH genetic sequence database: GenBank species TBLASTN WGS
Genolevures: Genomic Exploration of the Hemiascomycete Yeasts
Publication/s:
de Montigny J *et. al.* , *FEBS Lett* , **487** , 87 (2000).

Scheffersomyces stipitis CBS 6054 **(Shs)**
Taxonomy:
cellular organisms | Eukaryota | Opisthokonta | Fungi | Dikarya | Ascomycota | saccharomyceta | Saccharomycotina | Saccharomycetes | Saccharomycetales |

Debaryomycetaceae | Scheffersomyces | Scheffersomyces stipitis
Reference/s:
DOE Joint Genome Institute: Pichia stipitis
International Species Sequencing Consortium: Pichia stipitis Sequencing Consortium
National Center for Biotechnology Information: NCBI Fungi Genomes Project
Publication/s:
Jeffries TW *et. al.* , *Nat Biotechnol* , **25** , 319 (2007).

***Spathaspora passalidarum NRRL Y-27907* (Shp)**
Taxonomy:
cellular organisms | Eukaryota | Opisthokonta | Fungi | Dikarya | Ascomycota | saccharomyceta | Saccharomycotina | Saccharomycetes | Saccharomycetales | Debaryomycetaceae | Spathaspora | Spathaspora passalidarum
Reference/s:
DOE Joint Genome Institute: Spathaspora passalidarum NRRL Y-27907 v2.0
International Species Sequencing Consortium: Spathaspora Sequencing Consortium
National Center for Biotechnology Information: NCBI Fungi Genomes Project
Publication/s:
Wohlbach DJ *et. al.* , *Proc Natl Acad Sci U S A* , **108** , 13212 (2011).

***Yarrowia lipolytica CLIB122* (Yl)**
Taxonomy:
cellular organisms | Eukaryota | Opisthokonta | Fungi | Dikarya | Ascomycota | saccharomyceta | Saccharomycotina | Saccharomycetes | Saccharomycetales | Dipodascaceae | Yarrowia | Yarrowia lipolytica
Reference/s:
Genolevures: Genomic Exploration of the Hemiascomycete Yeasts
National Center for Biotechnology Information: NCBI Fungi Genomes Project
Publication/s:
Dujon B *et. al.* , *Nature* , **430** , 35 (2004).
Casaregola S *et. al.* , *FEBS Lett* , **487** , 95 (2000).

***Lipomyces starkeyi NRRL Y-11557* (Lms)**
Taxonomy:
cellular organisms | Eukaryota | Opisthokonta | Fungi | Dikarya | Ascomycota | saccharomyceta | Saccharomycotina | Saccharomycetes | Saccharomycetales | Lipomycetaceae | Lipomyces | Lipomyces starkeyi
Reference/s:
DOE Joint Genome Institute: Lipomyces starkeyi NRRL Y-11557 v1.0

***Clavispora lusitaniae ATCC 42720* (Cll)**
Taxonomy:
cellular organisms | Eukaryota | Opisthokonta | Fungi | Dikarya | Ascomycota | saccharomyceta | Saccharomycotina | Saccharomycetes | Saccharomycetales | Metschnikowiaceae | Clavispora | Clavispora lusitaniae
Reference/s:
Broad Institute of Harvard and MIT: Candida lusitaniae Sequencing Project
International Species Sequencing Consortium: Clavispora lusitaniae Sequencing Consortium
National Center for Biotechnology Information: NCBI Fungi Genomes Project
Publication/s:
Butler G *et. al.* , *Nature* , **459** , 657 (2009).

***Wickerhamomyces anomalus NRRL Y-366* (Wa)**
Taxonomy:
cellular organisms | Eukaryota | Opisthokonta | Fungi | Dikarya | Ascomycota | saccharomyceta | Saccharomycotina | Saccharomycetes | Saccharomycetales | Phaffomycetaceae | Wickerhamomyces | Wickerhamomyces anomalus
Reference/s:
National Center for Biotechnology Information: NCBI Fungi Genomes Project

***Pichia membranifaciens* (Phm)**
Taxonomy:
cellular organisms | Eukaryota | Opisthokonta | Fungi | Dikarya | Ascomycota | saccharomyceta | Saccharomycotina | Saccharomycetes | Saccharomycetales | Pichiaceae | Pichia
Reference/s:
DOE Joint Genome Institute: Pichia membranifaciens v2.0
DOE Joint Genome Institute: Pichia membranifaciens v1.0

***Eremothecium gossypii ATCC 10895* (Erg)**
Taxonomy:
cellular organisms | Eukaryota | Opisthokonta | Fungi | Dikarya | Ascomycota | saccharomyceta | Saccharomycotina | Saccharomycetes | Saccharomycetales | Saccharomycetaceae | Eremothecium | Eremothecium gossypii
Reference/s:
Ashbya Genome Database: Ashbya Genome Database
International Species Sequencing Consortium: Eremothecium gossypii Sequencing Consortium
National Center for Biotechnology Information: NCBI Fungi Genomes Project
National Center for Biotechnology Information Reference Sequences: Eremothecium gossypii genome view
Publication/s:
Dietrich FS *et. al.* , *Science* , **304** , 304 (2004).

***Kluyveromyces aestuarii ATCC 18862* (Ka)**
Taxonomy:
cellular organisms | Eukaryota | Opisthokonta | Fungi | Dikarya | Ascomycota | saccharomyceta | Saccharomycotina | Saccharomycetes | Saccharomycetales | Saccharomycetaceae | Kluyveromyces | Kluyveromyces aestuarii
Reference/s:

International Species Sequencing Consortium: Kluyveromyces Sequencing Consortium
National Center for Biotechnology Information: NCBI Fungi Genomes Project
Publication/s:
Baker CR, Tuch BB, Johnson AD , *Proc Natl Acad Sci U S A* , **108** , 7493 (2011).

Kluyveromyces lactis NRRL Y-1140 **(Kl)**
Taxonomy:
cellular organisms | Eukaryota | Opisthokonta | Fungi | Dikarya | Ascomycota | saccharomyceta | Saccharomycotina | Saccharomycetes | Saccharomycetales | Saccharomycetaceae | Kluyveromyces | Kluyveromyces lactis
Reference/s:
Genolevures: Genomic Exploration of the Hemiascomycete Yeasts
National Center for Biotechnology Information: NCBI Fungi Genomes Project
Publication/s:
Dujon B *et. al.* , *Nature* , **430** , 35 (2004).
Bolotin-Fukuhara M *et. al.* , *FEBS Lett* , **487** , 66 (2000).

Kluyveromyces wickerhamii UCD 54-210 **(Klw)**
Taxonomy:
cellular organisms | Eukaryota | Opisthokonta | Fungi | Dikarya | Ascomycota | saccharomyceta | Saccharomycotina | Saccharomycetes | Saccharomycetales | Saccharomycetaceae | Kluyveromyces | Kluyveromyces wickerhamii
Reference/s:
International Species Sequencing Consortium: Kluyveromyces Sequencing Consortium
National Center for Biotechnology Information: NCBI Fungi Genomes Project
Publication/s:
Baker CR, Tuch BB, Johnson AD , *Proc Natl Acad Sci U S A* , **108** , 7493 (2011).

Komagataella pastoris GS115 **(Kop_b)**
Taxonomy:
cellular organisms | Eukaryota | Opisthokonta | Fungi | Dikarya | Ascomycota | saccharomyceta | Saccharomycotina | Saccharomycetes | Saccharomycetales | Saccharomycetaceae | Komagataella | Komagataella pastoris
Reference/s:
International Species Sequencing Consortium: Pichia pastoris Sequencing Consortium
National Center for Biotechnology Information: NCBI Fungi Genomes Project
Publication/s:
De Schutter K *et. al.* , *Nat Biotechnol* , **27** , 561 (2009).

Komagataella pastoris DSMZ 70382 **(Kop_a)**
Taxonomy:
cellular organisms | Eukaryota | Opisthokonta | Fungi | Dikarya | Ascomycota | saccharomyceta | Saccharomycotina | Saccharomycetes | Saccharomycetales | Saccharomycetaceae | Komagataella | Komagataella pastoris
Reference/s:
International Species Sequencing Consortium: Pichia pastoris Sequencing Consortium
National Center for Biotechnology Information: NCBI Fungi Genomes Project
Publication/s:
Mattanovich D *et. al.* , *Microb Cell Fact* , **8** , 29 (2009).

Lachancea kluyveri CBS 3082 **(Lak_b)**
Taxonomy:
cellular organisms | Eukaryota | Opisthokonta | Fungi | Dikarya | Ascomycota | saccharomyceta | Saccharomycotina | Saccharomycetes | Saccharomycetales | Saccharomycetaceae | Lachancea
Reference/s:
Genolevures: Genomic Exploration of the Hemiascomycete Yeasts
Publication/s:
Souciet JL *et. al.* , *Genome Res* , **19** , 1696 (2009).
Neuveglise C *et. al.* , *FEBS Lett* , **487** , 56 (2000).

Lachancea kluyveri NRRL Y-12651 **(Lak_a)**
Taxonomy:
cellular organisms | Eukaryota | Opisthokonta | Fungi | Dikarya | Ascomycota | saccharomyceta | Saccharomycotina | Saccharomycetes | Saccharomycetales | Saccharomycetaceae | Lachancea | Lachancea kluyveri
Reference/s:
International Species Sequencing Consortium: Washington University Saccharomyces kluyveri Sequencing
National Center for Biotechnology Information: NCBI Fungi Genomes Project
The Genome Sequencing Center at Washington University: Saccharomyces kluyveri
Publication/s:
Cliften *et. al.* , *Science* , **301** , 71 (2003).

Lachancea thermotolerans CBS 6340 **(Lat)**
Taxonomy:
cellular organisms | Eukaryota | Opisthokonta | Fungi | Dikarya | Ascomycota | saccharomyceta | Saccharomycotina | Saccharomycetes | Saccharomycetales | Saccharomycetaceae | Lachancea | Lachancea thermotolerans
Reference/s:
Genolevures: Genomic Exploration of the Hemiascomycete Yeasts
National Center for Biotechnology Information: NCBI Fungi Genomes Project
Publication/s:
Souciet JL *et. al.* , *Genome Res* , **19** , 1696 (2009).
Malpertuy A *et. al.* , *FEBS Letters* , **487** , 61 (2000).

Lachancea waltii NCYC 2644 **(Lw)**
Taxonomy:

cellular organisms | Eukaryota | Opisthokonta | Fungi | Dikarya | Ascomycota | saccharomyceta | Saccharomycotina | Saccharomycetes | Saccharomycetales | Saccharomycetaceae | Lachancea | Lachancea waltii
Reference/s:
International Species Sequencing Consortium: The Broad Institute Kluyveromyces waltii Sequencing Initiative
National Center for Biotechnology Information: NCBI Fungi Genomes Project
Publication/s:
Manolis Kellis, Bruce W. Birren, Eric S. Lander , *Nature* , **428** , 617 (2004).

Candida glabrata CBS138 **(Cgl)**
Taxonomy:
cellular organisms | Eukaryota | Opisthokonta | Fungi | Dikarya | Ascomycota | saccharomyceta | Saccharomycotina | Saccharomycetes | Saccharomycetales | Saccharomycetaceae | Nakaseomyces | mitosporic Nakaseomyces | Candida glabrata
Reference/s:
Genolevures: Genomic Exploration of the Hemiascomycete Yeasts
National Center for Biotechnology Information: NCBI Fungi Genomes Project
Publication/s:
Dujon B *et. al.* , *Nature* , **430** , 35 (2004).

Naumovozyma castellii NRRL Y-12630 **(Nac)**
Taxonomy:
cellular organisms | Eukaryota | Opisthokonta | Fungi | Dikarya | Ascomycota | saccharomyceta | Saccharomycotina | Saccharomycetes | Saccharomycetales | Saccharomycetaceae | Naumovozyma | Naumovozyma castellii
Reference/s:
National Center for Biotechnology Information: NCBI Fungi Genomes Project
The Genome Sequencing Center at Washington University: Finding functional features in Saccharomyces Genomes by phylogenetic footprinting
Publication/s:
Cliften *et. al.* , *Science* , **301** , 71 (2003).

Naumovozyma dairenensis CBS 421 **(Nad)**
Taxonomy:
cellular organisms | Eukaryota | Opisthokonta | Fungi | Dikarya | Ascomycota | saccharomyceta | Saccharomycotina | Saccharomycetes | Saccharomycetales | Saccharomycetaceae | Naumovozyma | Naumovozyma dairenensis
Reference/s:
GenBank - NIH genetic sequence database: GenBank species TBLASTN WGS

Pachysolen tannophilus NRRL Y-2460 **(Pta)**
Taxonomy:
cellular organisms | Eukaryota | Opisthokonta | Fungi | Dikarya | Ascomycota | saccharomyceta | Saccharomycotina | Saccharomycetes | Saccharomycetales | Saccharomycetaceae | Pachysolen | Pachysolen tannophilus
Reference/s:
DOE Joint Genome Institute: Pachysolen tannophilus NRRL Y-2460 v1.2

Saccharomyces bayanus var. uvarum **(Suv)**
Taxonomy:
cellular organisms | Eukaryota | Opisthokonta | Fungi | Dikarya | Ascomycota | saccharomyceta | Saccharomycotina | Saccharomycetes | Saccharomycetales | Saccharomycetaceae | Saccharomyces
Reference/s:
Genolevures: Genomic Exploration of the Hemiascomycete Yeasts
Publication/s:
Bon E *et. al.* , *FEBS Letters* , **487** , 37 (2000).

Saccharomyces bayanus 623-6C **(Sab_a)**
Taxonomy:
cellular organisms | Eukaryota | Opisthokonta | Fungi | Dikarya | Ascomycota | saccharomyceta | Saccharomycotina | Saccharomycetes | Saccharomycetales | Saccharomycetaceae | Saccharomyces | Saccharomyces bayanus
Reference/s:
National Center for Biotechnology Information: NCBI Fungi Genomes Project
The Genome Sequencing Center at Washington University: Finding functional features in Saccharomyces Genomes by phylogenetic footprinting
Publication/s:
Cliften *et. al.* , *Science* , **301** , 71 (2003).

Saccharomyces bayanus MCYC 623 **(Sab_b)**
Taxonomy:
cellular organisms | Eukaryota | Opisthokonta | Fungi | Dikarya | Ascomycota | saccharomyceta | Saccharomycotina | Saccharomycetes | Saccharomycetales | Saccharomycetaceae | Saccharomyces | Saccharomyces bayanus
Reference/s:
Broad Institute of Harvard and MIT: Yeast Comparative Genomics
National Center for Biotechnology Information: NCBI Fungi Genomes Project
Publication/s:
Kellis *et. al.* , *Nature* , **423** , 241 (2003).

Saccharomyces cerevisiae S288c **(Sc_c)**
Taxonomy:
cellular organisms | Eukaryota | Opisthokonta | Fungi | Dikarya | Ascomycota | saccharomyceta | Saccharomycotina | Saccharomycetes | Saccharomycetales | Saccharomycetaceae | Saccharomyces | Saccharomyces cerevisiae
Reference/s:
Ashbya Genome Database: Ashbya Genome Database
Genolevures: Re-Annotation of the Saccharomyces cerevisiae Genome
International Species Sequencing Consortium: Saccharomyces cerevisiae Sequencing Consortium
National Center for Biotechnology Information: NCBI Fungi Genomes Project

National Center for Biotechnology Information Reference Sequences: Saccharomyces Genome Resources
Saccharomyces Genome Database: Database of the molecular biology and genetics of the yeast Saccharomyces cerevisiae
The Gene Index Project: DFCI Saccharomyces cerevisiae Gene Index
The Wellcome Trust Sanger Institute: Saccharomyces Genome Resequencing Project
e! Ensembl: S.cerevisiae
Publication/s:
Liti G *et. al.* , *Nature* , **458** , 337 (2009).
Blandin G *et. al.* , *FEBS Lett* , **487** , 31 (2000).
Foury F *et. al.* , *FEBS Lett* , **440** , 325 (1998).
Dujon B *et. al.* , *Nature* , **387** , 98 (1997).
Tettelin *et. al.* , *Nature* , **387** , 81 (1997).
Goffeau *et. al.* , *Science* , **274** , 546 (1996).
Johnston M *et. al.* , *Science* , **265** , 2077 (1994).

Saccharomyces cerevisiae M22 **(Sc_al)**
Taxonomy:
cellular organisms | Eukaryota | Opisthokonta | Fungi | Dikarya | Ascomycota | saccharomyceta | Saccharomycotina | Saccharomycetes | Saccharomycetales | Saccharomycetaceae | Saccharomyces | Saccharomyces cerevisiae
Reference/s:
National Center for Biotechnology Information: NCBI Fungi Genomes Project
Publication/s:
Doniger SW *et. al.* , *PLoS Genet* , **4** , e1000183 (2008).
Doniger SW *et. al.* , *PLoS Genet* , **4** , e1000183 (2008).

Saccharomyces cerevisiae RM11-1a **(Sc_b)**
Taxonomy:
cellular organisms | Eukaryota | Opisthokonta | Fungi | Dikarya | Ascomycota | saccharomyceta | Saccharomycotina | Saccharomycetes | Saccharomycetales | Saccharomycetaceae | Saccharomyces | Saccharomyces cerevisiae
Reference/s:
Broad Institute of Harvard and MIT: Saccharomyces cerevisiae RM11-1a Sequencing Project
National Center for Biotechnology Information: NCBI Fungi Genomes Project
The Gene Index Project: DFCI Saccharomyces cerevisiae Gene Index

Saccharomyces cerevisiae YJM789 **(Sc_a)**
Taxonomy:
cellular organisms | Eukaryota | Opisthokonta | Fungi | Dikarya | Ascomycota | saccharomyceta | Saccharomycotina | Saccharomycetes | Saccharomycetales | Saccharomycetaceae | Saccharomyces | Saccharomyces cerevisiae
Reference/s:
International Species Sequencing Consortium: Saccharomyces cerevisiae YJM789 Sequencing Consortium
National Center for Biotechnology Information: NCBI Fungi Genomes Project
Stanford Genome Technology Center: YJM789 genome sequence
The Gene Index Project: DFCI Saccharomyces cerevisiae Gene Index
Publication/s:
Wei W *et. al.* , *Proc Natl Acad Sci U S A* , **104** , 12825 (2007).

Saccharomyces cerevisiae YPS163 **(Sc_am)**
Taxonomy:
cellular organisms | Eukaryota | Opisthokonta | Fungi | Dikarya | Ascomycota | saccharomyceta | Saccharomycotina | Saccharomycetes | Saccharomycetales | Saccharomycetaceae | Saccharomyces | Saccharomyces cerevisiae
Reference/s:
International Species Sequencing Consortium: Saccharomyces Genome Sequencing
National Center for Biotechnology Information: NCBI Fungi Genomes Project
Publication/s:
Doniger SW *et. al.* , *PLoS Genet* , **4** , e1000183 (2008).

Saccharomyces kudriavzevii IFO 1802 **(Sak)**
Taxonomy:
cellular organisms | Eukaryota | Opisthokonta | Fungi | Dikarya | Ascomycota | saccharomyceta | Saccharomycotina | Saccharomycetes | Saccharomycetales | Saccharomycetaceae | Saccharomyces | Saccharomyces kudriavzevii
Reference/s:
National Center for Biotechnology Information: NCBI Fungi Genomes Project
The Genome Sequencing Center at Washington University: Finding functional features in Saccharomyces Genomes by phylogenetic footprinting
Publication/s:
Cliften *et. al.* , *Science* , **301** , 71 (2003).

Saccharomyces mikatae IFO 1815 **(Smi)**
Taxonomy:
cellular organisms | Eukaryota | Opisthokonta | Fungi | Dikarya | Ascomycota | saccharomyceta | Saccharomycotina | Saccharomycetes | Saccharomycetales | Saccharomycetaceae | Saccharomyces | Saccharomyces mikatae
Reference/s:
Broad Institute of Harvard and MIT: Yeast Comparative Genomics
National Center for Biotechnology Information: NCBI Fungi Genomes Project
The Genome Sequencing Center at Washington University: Finding functional features in Saccharomyces Genomes by phylogenetic footprinting
Publication/s:
Kellis *et. al.* , *Nature* , **423** , 241 (2003).
Cliften *et. al.* , *Science* , **301** , 71 (2003).
Cliften *et. al.* , *Science* , **301** , 71 (2003).

Saccharomyces paradoxus NRRL Y-17217 **(Sap_a)**
Taxonomy:

cellular organisms | Eukaryota | Opisthokonta | Fungi | Dikarya | Ascomycota | saccharomyceta | Saccharomycotina | Saccharomycetes | Saccharomycetales | Saccharomycetaceae | Saccharomyces | Saccharomyces paradoxus
Reference/s:
Broad Institute of Harvard and MIT: Yeast Comparative Genomics
National Center for Biotechnology Information: NCBI Fungi Genomes Project
Publication/s:
Kellis *et. al.* , *Nature* , **423** , 241 (2003).

Saccharomyces pastorianus Weihenstephan 34/70 **(Scp)**
Taxonomy:
cellular organisms | Eukaryota | Opisthokonta | Fungi | Dikarya | Ascomycota | saccharomyceta | Saccharomycotina | Saccharomycetes | Saccharomycetales | Saccharomycetaceae | Saccharomyces | Saccharomyces pastorianus
Reference/s:
International Species Sequencing Consortium: Saccharomyces pastorianus Weihenstephan Sequencing Consortium
National Center for Biotechnology Information: NCBI Fungi Genomes Project
Publication/s:
Nakao Y *et. al.* , *DNA Res* , **16** , 115 (2009).

Tetrapisispora phaffii CBS 4417 **(Ttp)**
Taxonomy:
cellular organisms | Eukaryota | Opisthokonta | Fungi | Dikarya | Ascomycota | saccharomyceta | Saccharomycotina | Saccharomycetes | Saccharomycetales | Saccharomycetaceae | Tetrapisispora | Tetrapisispora phaffii
Reference/s:
GenBank - NIH genetic sequence database: GenBank species TBLASTN WGS

Torulaspora delbrueckii CBS 1146 **(Tod)**
Taxonomy:
cellular organisms | Eukaryota | Opisthokonta | Fungi | Dikarya | Ascomycota | saccharomyceta | Saccharomycotina | Saccharomycetes | Saccharomycetales | Saccharomycetaceae | Torulaspora | Torulaspora delbrueckii
Reference/s:
GenBank - NIH genetic sequence database: GenBank species TBLASTN WGS

Vanderwaltozyma polyspora DSM 70294 **(Vp)**
Taxonomy:
cellular organisms | Eukaryota | Opisthokonta | Fungi | Dikarya | Ascomycota | saccharomyceta | Saccharomycotina | Saccharomycetes | Saccharomycetales | Saccharomycetaceae | Vanderwaltozyma | Vanderwaltozyma polyspora
Reference/s:
International Species Sequencing Consortium: Vanderwaltozyma Sequencing Consortium
National Center for Biotechnology Information: NCBI Fungi Genomes Project
Publication/s:
Scannell DR *et. al.* , *Proc Natl Acad Sci U S A* , **104** , 8397 (2007).

Zygosaccharomyces rouxii CBS732 **(Zr)**
Taxonomy:
cellular organisms | Eukaryota | Opisthokonta | Fungi | Dikarya | Ascomycota | saccharomyceta | Saccharomycotina | Saccharomycetes | Saccharomycetales | Saccharomycetaceae | Zygosaccharomyces
Reference/s:
Genolevures: Genomic Exploration of the Hemiascomycete Yeasts
National Center for Biotechnology Information: NCBI Fungi Genomes Project
Publication/s:
Souciet JL *et. al.* , *Genome Res* , **19** , 1696 (2009).
de Montigny J *et. al.* , *FEBS Letters* , **487** , 52 (2000).

Ogataea angusta NCYC 495 leu1.1 **(Oga)**
Taxonomy:
cellular organisms | Eukaryota | Opisthokonta | Fungi | Dikarya | Ascomycota | saccharomyceta | Saccharomycotina | Saccharomycetes | Saccharomycetales | Saccharomycetales incertae sedis | Ogataea | Ogataea angusta
Reference/s:
DOE Joint Genome Institute: Hansenula polymorpha NCYC 495 leu1.1 v2.0
DOE Joint Genome Institute: Hansenula polymorpha NCYC 495 leu1.1

Ogataea parapolymorpha DL-1 **(Ogp)**
Taxonomy:
cellular organisms | Eukaryota | Opisthokonta | Fungi | Dikarya | Ascomycota | saccharomyceta | Saccharomycotina | Saccharomycetes | Saccharomycetales | Saccharomycetales incertae sedis | Ogataea | Ogataea parapolymorpha
Reference/s:
National Center for Biotechnology Information: NCBI Fungi Genomes Project

Ogataea polymorpha CBS 4732 **(Oap)**
Taxonomy:
cellular organisms | Eukaryota | Opisthokonta | Fungi | Dikarya | Ascomycota | saccharomyceta | Saccharomycotina | Saccharomycetes | Saccharomycetales | Saccharomycetales incertae sedis | Ogataea | Ogataea polymorpha
Reference/s:
Genolevures: Genomic Exploration of the Hemiascomycete Yeasts
Publication/s:
Blandin G *et. al.* , *FEBS Letters* , **487** , 76 (2000).

Nadsonia fulvescens var. elongata DSM 6958 **(Nfe)**
Taxonomy:
cellular organisms | Eukaryota | Opisthokonta | Fungi | Dikarya | Ascomycota | saccharomyceta | Saccharomycotina | Saccharomycetes | Saccharomycetales |

Saccharomycodaceae | Nadsonia | Nadsonia fulvescens | Nadsonia fulvescens var. elongata
Reference/s:
DOE Joint Genome Institute: Nadsonia fulvescens var. elongata DSM 6958 v1.0

***Candida parapsilosis* (Cap)**
Taxonomy:
cellular organisms | Eukaryota | Opisthokonta | Fungi | Dikarya | Ascomycota | saccharomyceta | Saccharomycotina | Saccharomycetes | Saccharomycetales | mitosporic Saccharomycetales | Candida
Reference/s:
International Species Sequencing Consortium: Candida Sequencing Consortium
National Center for Biotechnology Information: NCBI Fungi Genomes Project
The Wellcome Trust Sanger Institute: Candida parapsilosis Sequencing
Publication/s:
Butler G *et. al.* , *Nature* , **459** , 657 (2009).

***Candida tenuis NRRL Y-1498* (Cat)**
Taxonomy:
cellular organisms | Eukaryota | Opisthokonta | Fungi | Dikarya | Ascomycota | saccharomyceta | Saccharomycotina | Saccharomycetes | Saccharomycetales | mitosporic Saccharomycetales | Candida
Reference/s:
DOE Joint Genome Institute: Candida tenuis NRRL Y-1498 v1.0
International Species Sequencing Consortium: Candida tenuis Sequencing Consortium
National Center for Biotechnology Information: NCBI Fungi Genomes Project
Publication/s:
Wohlbach DJ *et. al.* , *Proc Natl Acad Sci U S A* , **108** , 13212 (2011).

***Candida albicans WO-1* (Ca_b)**
Taxonomy:
cellular organisms | Eukaryota | Opisthokonta | Fungi | Dikarya | Ascomycota | saccharomyceta | Saccharomycotina | Saccharomycetes | Saccharomycetales | mitosporic Saccharomycetales | Candida | Candida albicans
Reference/s:
Broad Institute of Harvard and MIT: Candida albicans Database
International Species Sequencing Consortium: Candida Sequencing Consortium
National Center for Biotechnology Information: NCBI Fungi Genomes Project
Publication/s:
Butler G *et. al.* , *Nature* , **459** , 657 (2009).

***Candida albicans SC5314* (Ca_a)**
Taxonomy:
cellular organisms | Eukaryota | Opisthokonta | Fungi | Dikarya | Ascomycota | saccharomyceta | Saccharomycotina | Saccharomycetes | Saccharomycetales | mitosporic Saccharomycetales | Candida | Candida albicans
Reference/s:
Candida Genome Database: A resource for genomic sequence data and gene and protein information for Candida albicans
International Species Sequencing Consortium: Candida albicans Sequencing Consortium
National Center for Biotechnology Information: NCBI Fungi Genomes Project
Stanford Genome Technology Center: Sequencing of Candida Albicans
Publication/s:
Butler G *et. al.* , *Nature* , **459** , 657 (2009).
van het Hoog M *et. al.* , *Genome Biol* , **8** , R52 (2007).
Braun BR *et. al.* , *PLoS Genet* , **1** , 36 (2005).
Jones T *et. al.* , *Proc Natl Acad Sci U S A* , **101** , 7329 (2004).

***Candida caseinolytica NRRL Y-17796* (Cac)**
Taxonomy:
cellular organisms | Eukaryota | Opisthokonta | Fungi | Dikarya | Ascomycota | saccharomyceta | Saccharomycotina | Saccharomycetes | Saccharomycetales | mitosporic Saccharomycetales | Candida | Candida caseinolytica
Reference/s:
DOE Joint Genome Institute: Candida caseinolytica Y-17796 v1.0

***Candida dubliniensis CD36* (Cad)**
Taxonomy:
cellular organisms | Eukaryota | Opisthokonta | Fungi | Dikarya | Ascomycota | saccharomyceta | Saccharomycotina | Saccharomycetes | Saccharomycetales | mitosporic Saccharomycetales | Candida | Candida dubliniensis
Reference/s:
International Species Sequencing Consortium: Candida Sequencing Consortium
National Center for Biotechnology Information: NCBI Fungi Genomes Project
The Wellcome Trust Sanger Institute: Candida dubliniensis Genome Sequencing
Publication/s:
Jackson AP *et. al.* , *Genome Res* , **19** , 2231 (2009).

***Candida tropicalis MYA-3404* (Ct_a)**
Taxonomy:
cellular organisms | Eukaryota | Opisthokonta | Fungi | Dikarya | Ascomycota | saccharomyceta | Saccharomycotina | Saccharomycetes | Saccharomycetales | mitosporic Saccharomycetales | Candida | Candida tropicalis
Reference/s:
Broad Institute of Harvard and MIT: Candida tropicalis Sequencing Project
International Species Sequencing Consortium: Candida tropicalis MYA-3404 Sequencing Consortium
National Center for Biotechnology Information: NCBI Fungi Genomes Project
Publication/s:
Butler G *et. al.* , *Nature* , **459** , 657 (2009).

Phanerochaete chrysosporium RP-78 **(Phc)**
Taxonomy:
cellular organisms | Eukaryota | Opisthokonta | Fungi | Dikarya | Basidiomycota | Agaricomycotina | Agaricomycetes | Agaricomycetes incertae sedis | Corticiales | Corticiaceae | Phanerochaete | Phanerochaete chrysosporium
Reference/s:
DOE Joint Genome Institute: Phanerochaete chrysosporium
Fungal Genomics Project: Phanerochaete chrysosporium
International Species Sequencing Consortium: Phanerochaete chrysosporium Sequencing Consortium
National Center for Biotechnology Information: NCBI Fungi Genomes Project
Publication/s:
Martinez D *et. al.* , *Nat Biotechnol* , **22** , 695 (2004).

Punctularia strigosozonata **(Pus)**
Taxonomy:
cellular organisms | Eukaryota | Opisthokonta | Fungi | Dikarya | Basidiomycota | Agaricomycotina | Agaricomycetes | Agaricomycetes incertae sedis | Corticiales | Punctulariaceae | Punctularia
Reference/s:
DOE Joint Genome Institute: Punctularia strigosozonata v1.0

Gloeophyllum trabeum **(Glt)**
Taxonomy:
cellular organisms | Eukaryota | Opisthokonta | Fungi | Dikarya | Basidiomycota | Agaricomycotina | Agaricomycetes | Agaricomycetes incertae sedis | Gloeophyllales | Gloeophyllaceae | Gloeophyllum
Reference/s:
DOE Joint Genome Institute: Gloeophyllum trabeum
Fungal Genomics Project: Gloeophyllum trabeum

Fomitopsis pinicola **(Fp)**
Taxonomy:
cellular organisms | Eukaryota | Opisthokonta | Fungi | Dikarya | Basidiomycota | Agaricomycotina | Agaricomycetes | Agaricomycetes incertae sedis | Polyporales | Coriolaceae | Fomitopsis
Reference/s:
DOE Joint Genome Institute: Fomitopsis pinicola SS1 v1.0

Postia placenta **(Ppl)**
Taxonomy:
cellular organisms | Eukaryota | Opisthokonta | Fungi | Dikarya | Basidiomycota | Agaricomycotina | Agaricomycetes | Agaricomycetes incertae sedis | Polyporales | Coriolaceae | Postia
Reference/s:
DOE Joint Genome Institute: The Genome Sequence of the Brown Rot Fungus Postia placenta
International Species Sequencing Consortium: Postia placenta Sequencing Consortium
National Center for Biotechnology Information: NCBI Fungi Genomes Project
Publication/s:
Martinez D *et. al.* , *Proc Natl Acad Sci U S A* , **106** , 1954 (2009).

Antrodia cinnamomea **(Atc)**
Taxonomy:
cellular organisms | Eukaryota | Opisthokonta | Fungi | Dikarya | Basidiomycota | Agaricomycotina | Agaricomycetes | Agaricomycetes incertae sedis | Polyporales | Coriolaceae | Taiwanofungus

Trametes versicolor **(Tav)**
Taxonomy:
cellular organisms | Eukaryota | Opisthokonta | Fungi | Dikarya | Basidiomycota | Agaricomycotina | Agaricomycetes | Agaricomycetes incertae sedis | Polyporales | Coriolaceae | Trametes
Reference/s:
DOE Joint Genome Institute: Trametes versicolor v1.0
Fungal Genomics Project: Trametes versicolor

Wolfiporia cocos MD-104 SS10 **(Wc)**
Taxonomy:
cellular organisms | Eukaryota | Opisthokonta | Fungi | Dikarya | Basidiomycota | Agaricomycotina | Agaricomycetes | Agaricomycetes incertae sedis | Polyporales | Coriolaceae | Wolfiporia | Wolfiporia cocos
Reference/s:
DOE Joint Genome Institute: Wolfiporia cocos MD-104 SS10

Dichomitus squalens **(Dis)**
Taxonomy:
cellular organisms | Eukaryota | Opisthokonta | Fungi | Dikarya | Basidiomycota | Agaricomycotina | Agaricomycetes | Agaricomycetes incertae sedis | Polyporales | Polyporaceae | Dichomitus
Reference/s:
DOE Joint Genome Institute: Dichomitus squalens v1.0

Heterobasidion annosum **(Hta)**
Taxonomy:
cellular organisms | Eukaryota | Opisthokonta | Fungi | Dikarya | Basidiomycota | Agaricomycotina | Agaricomycetes | Agaricomycetes incertae sedis | Russulales | Bondarzewiaceae | Heterobasidion | Heterobasidion annosum species complex
Reference/s:
DOE Joint Genome Institute: Heterobasidion annosum v2.0
DOE Joint Genome Institute: Heterobasidion annosum
GenBank - NIH genetic sequence database: GenBank species TBLASTN

Publication/s:
Karlsson M, Olson A, Stenlid J , *Fungal Genet Biol* , **39** , 51 (2003).

Gelatoporia subvermispora **(Ges)**
Taxonomy:
cellular organisms | Eukaryota | Opisthokonta | Fungi | Dikarya | Basidiomycota | Agaricomycotina | Agaricomycetes | Agaricomycetes incertae sedis | Russulales | Meruliaceae | Ceriporiopsis
Reference/s:
DOE Joint Genome Institute: Ceriporiopsis subvermispora B
International Species Sequencing Consortium: Ceriporiopsis subvermispora Sequencing Consortium
National Center for Biotechnology Information: NCBI Fungi Genomes Project
Publication/s:
Fernandez-Fueyo E *et. al.* , *Proc Natl Acad Sci U S A* , **109** , 5458 (2012).

Stereum hirsutum FP-91666 SS1 **(Sth)**
Taxonomy:
cellular organisms | Eukaryota | Opisthokonta | Fungi | Dikarya | Basidiomycota | Agaricomycotina | Agaricomycetes | Agaricomycetes incertae sedis | Russulales | Stereaceae | Stereum | Stereum hirsutum
Reference/s:
DOE Joint Genome Institute: Stereum hirsutum FP-91666 SS1 v1.0

Agaricus bisporus var. bisporus **(Abb)**
Taxonomy:
cellular organisms | Eukaryota | Opisthokonta | Fungi | Dikarya | Basidiomycota | Agaricomycotina | Agaricomycetes | Agaricomycetidae | Agaricales | Agaricaceae | Agaricus | Agaricus bisporus
Reference/s:
DOE Joint Genome Institute: Agaricus bisporus var bisporus (H97)

Agaricus bisporus var. burnettii JB137-S8 **(Agb)**
Taxonomy:
cellular organisms | Eukaryota | Opisthokonta | Fungi | Dikarya | Basidiomycota | Agaricomycotina | Agaricomycetes | Agaricomycetidae | Agaricales | Agaricaceae | Agaricus | Agaricus bisporus | Agaricus bisporus var. burnettii
Reference/s:
DOE Joint Genome Institute: Agaricus bisporus var. burnettii JB137-S8

Lentinula edodes **(Lne)**
Taxonomy:
cellular organisms | Eukaryota | Opisthokonta | Fungi | Dikarya | Basidiomycota | Agaricomycotina | Agaricomycetes | Agaricomycetidae | Agaricales | Marasmiaceae | Lentinula
Reference/s:
Fungal Genomics Project: Lentinula edodes

Moniliophthora perniciosa FA553 **(Mop)**
Taxonomy:
cellular organisms | Eukaryota | Opisthokonta | Fungi | Dikarya | Basidiomycota | Agaricomycotina | Agaricomycetes | Agaricomycetidae | Agaricales | Marasmiaceae | mitosporic Marasmiaceae | Moniliophthora | Moniliophthora perniciosa
Reference/s:
International Species Sequencing Consortium: Moniliophthora perniciosa Sequencing Consortium
National Center for Biotechnology Information: NCBI Fungi Genomes Project
Publication/s:
Mondego JM *et. al.* , *BMC Genomics* , **9** , 548 (2008).

Pleurotus ostreatus PC15 **(Plo)**
Taxonomy:
cellular organisms | Eukaryota | Opisthokonta | Fungi | Dikarya | Basidiomycota | Agaricomycotina | Agaricomycetes | Agaricomycetidae | Agaricales | Pleurotaceae | Pleurotus
Reference/s:
DOE Joint Genome Institute: Pleurotus ostreatus PC15 v2.0
DOE Joint Genome Institute: Pleurotus ostreatus PC15

Pleurotus ostreatus PC9 **(Plo_a)**
Taxonomy:
cellular organisms | Eukaryota | Opisthokonta | Fungi | Dikarya | Basidiomycota | Agaricomycotina | Agaricomycetes | Agaricomycetidae | Agaricales | Pleurotaceae | Pleurotus
Reference/s:
DOE Joint Genome Institute: Pleurotus ostreatus PC9 v1.0

Coprinopsis cinerea okayama7#130 **(Cpc)**
Taxonomy:
cellular organisms | Eukaryota | Opisthokonta | Fungi | Dikarya | Basidiomycota | Agaricomycotina | Agaricomycetes | Agaricomycetidae | Agaricales | Psathyrellaceae | Coprinopsis
Reference/s:
Broad Institute of Harvard and MIT: Coprinus cinereus Sequencing Project
DOE Joint Genome Institute: Coprinopsis cinerea
Fungal Genomics Project: Coprinus cinereus
International Species Sequencing Consortium: Coprinopsis cinerea Sequencing Consortium
National Center for Biotechnology Information: NCBI Fungi Genomes Project
Publication/s:
Stajich JE *et. al.* , *Proc Natl Acad Sci U S A* , **107** , 11889 (2010).

***Schizophyllum commune H4-8* (Scc)**
Taxonomy:
cellular organisms | Eukaryota | Opisthokonta | Fungi | Dikarya | Basidiomycota | Agaricomycotina | Agaricomycetes | Agaricomycetidae | Agaricales | Schizophyllaceae | Schizophyllum | Schizophyllum commune
Reference/s:
DOE Joint Genome Institute: Schizophyllum commune v2.0
DOE Joint Genome Institute: Schizophyllum commune
International Species Sequencing Consortium: Schizophyllum Sequencing Consortium
National Center for Biotechnology Information: NCBI Fungi Genomes Project
Publication/s:
Ohm RA *et. al.* , *Nat Biotechnol* , **28** , 957 (2010).

***Laccaria bicolor S238N* (Lab)**
Taxonomy:
cellular organisms | Eukaryota | Opisthokonta | Fungi | Dikarya | Basidiomycota | Agaricomycotina | Agaricomycetes | Agaricomycetidae | Agaricales | Tricholomataceae | Laccaria
Reference/s:
DOE Joint Genome Institute: Laccaria bicolor
GenBank - NIH genetic sequence database: GenBank species TBLASTN
International Species Sequencing Consortium: Laccaria bicolor Sequencing Consortium
L'Institut National de la Recherche Agronomique: EctomycorrhizaDB
L'Institut National de la Recherche Agronomique: Laccaria Genome Resources
National Center for Biotechnology Information: NCBI Fungi Genomes Project
Publication/s:
Martin F *et. al.* , *Nature* , **452** , 88 (2008).
Peter M *et. al.* , *New Phytol* , **159** , 117 (2003).

***Coniophora puteana* (Chp)**
Taxonomy:
cellular organisms | Eukaryota | Opisthokonta | Fungi | Dikarya | Basidiomycota | Agaricomycotina | Agaricomycetes | Agaricomycetidae | Boletales | Coniophorineae | Coniophoraceae | Coniophora
Reference/s:
DOE Joint Genome Institute: Coniophora puteana v1.0

***Serpula lacrymans var. lacrymans S7.9* (Sll)**
Taxonomy:
cellular organisms | Eukaryota | Opisthokonta | Fungi | Dikarya | Basidiomycota | Agaricomycotina | Agaricomycetes | Agaricomycetidae | Boletales | Coniophorineae | Serpulaceae | Serpula | Serpula lacrymans | Serpula lacrymans var. lacrymans
Reference/s:
DOE Joint Genome Institute: Serpula lacrymans S7.9
International Species Sequencing Consortium: Serpula lacrymans Sequencing Consortium
National Center for Biotechnology Information: NCBI Fungi Genomes Project
Publication/s:
Eastwood DC *et. al.* , *Science* , **333** , 762 (2011).

***Pisolithus microcarpus* (Pim)**
Taxonomy:
cellular organisms | Eukaryota | Opisthokonta | Fungi | Dikarya | Basidiomycota | Agaricomycotina | Agaricomycetes | Agaricomycetidae | Boletales | Sclerodermatineae | Pisolithaceae | Pisolithus

***Filobasidiella neoformans var. bacillispora WM276* (Fnb_c)**
Taxonomy:
cellular organisms | Eukaryota | Opisthokonta | Fungi | Dikarya | Basidiomycota | Agaricomycotina | Tremellomycetes | Tremellales | Tremellaceae | Filobasidiella | Filobasidiella/Cryptococcus neoformans species complex
Reference/s:
National Center for Biotechnology Information: NCBI Fungi Genomes Project

***Filobasidiella neoformans var. bacillispora R265* (Fnb_b)**
Taxonomy:
cellular organisms | Eukaryota | Opisthokonta | Fungi | Dikarya | Basidiomycota | Agaricomycotina | Tremellomycetes | Tremellales | Tremellaceae | Filobasidiella | Filobasidiella/Cryptococcus neoformans species complex
Reference/s:
Broad Institute of Harvard and MIT: Cryptococcus neoformans Serotype B Database
National Center for Biotechnology Information: NCBI Fungi Genomes Project

***Filobasidiella neoformans var. neoformans H99* (Fna_b)**
Taxonomy:
cellular organisms | Eukaryota | Opisthokonta | Fungi | Dikarya | Basidiomycota | Agaricomycotina | Tremellomycetes | Tremellales | Tremellaceae | Filobasidiella | Filobasidiella/Cryptococcus neoformans species complex | Cryptococcus neoformans
Reference/s:
Broad Institute of Harvard and MIT: Cryptococcus neoformans Serotype A Database
DOE Joint Genome Institute: Cryptococcus neoformans var. grubii H99
National Center for Biotechnology Information: NCBI Fungi Genomes Project
The Gene Index Project: DFCI Cryptococcus sp. (Filobasidiella neoformans) Gene Index

***Filobasidiella neoformans var. neoformans JEC21* (Fnd_c)**
Taxonomy:
cellular organisms | Eukaryota | Opisthokonta | Fungi | Dikarya | Basidiomycota | Agaricomycotina | Tremellomycetes | Tremellales | Tremellaceae | Filobasidiella | Filobasidiella/Cryptococcus neoformans species complex | Cryptococcus neoformans
Reference/s:

International Species Sequencing Consortium: Filobasidiella neoformans var. neoformans JEC21 Sequencing Consortium
National Center for Biotechnology Information: NCBI Fungi Genomes Project
The Gene Index Project: DFCI Cryptococcus sp. (Filobasidiella neoformans) Gene Index
The Institute for Genomic Research: Cryptococcus neoformans Genome Project
Publication/s:
Loftus BJ *et. al.* , *Science* , **307** , 1321 (2005).

***Filobasidiella neoformans var. neoformans B-3501A* (Fnd_b)**
Taxonomy:
cellular organisms | Eukaryota | Opisthokonta | Fungi | Dikarya | Basidiomycota | Agaricomycotina | Tremellomycetes | Tremellales | Tremellaceae | Filobasidiella | Filobasidiella/Cryptococcus neoformans species complex | Cryptococcus neoformans
Reference/s:
GenBank - NIH genetic sequence database: GenBank species TBLASTN
International Species Sequencing Consortium: Filobasidiella Sequencing Consortium
National Center for Biotechnology Information: NCBI Fungi Genomes Project
Stanford Genome Technology Center: Cryptococcus neoformans Genome Project
The Gene Index Project: DFCI Cryptococcus sp. (Filobasidiella neoformans) Gene Index
Publication/s:
Loftus BJ *et. al.* , *Science* , **307** , 1321 (2005).
Kupfer DM *et. al.* , *Eukaryot Cell* , **3** , 1088 (2004).

***Tremella mesenterica* (Tem)**
Taxonomy:
cellular organisms | Eukaryota | Opisthokonta | Fungi | Dikarya | Basidiomycota | Agaricomycotina | Tremellomycetes | Tremellales | Tremellaceae | Tremella
Reference/s:
DOE Joint Genome Institute: Tremella mesenterica Fries

***Cryptococcus laurentii* (Crl)**
Taxonomy:
cellular organisms | Eukaryota | Opisthokonta | Fungi | Dikarya | Basidiomycota | Agaricomycotina | Tremellomycetes | Tremellales | mitosporic Tremellales | Cryptococcus
Reference/s:
Fungal Genomics Project: Cryptococcus laurentii

***Leucosporidium scottii* (Lsc)**
Taxonomy:
cellular organisms | Eukaryota | Opisthokonta | Fungi | Dikarya | Basidiomycota | Pucciniomycotina | Microbotryomycetes | Leucosporidiales | Leucosporidiaceae | Leucosporidium
Reference/s:
Fungal Genomics Project: Leucosporidium scottii

***Microbotryum violaceum* (Mv)**
Taxonomy:
cellular organisms | Eukaryota | Opisthokonta | Fungi | Dikarya | Basidiomycota | Pucciniomycotina | Microbotryomycetes | Microbotryales | Microbotryaceae | Microbotryum
Reference/s:
Broad Institute of Harvard and MIT: Microbotryum violaceum Database
National Center for Biotechnology Information: NCBI Fungi Genomes Project

***Rhodotorula graminis WP1* (Rg)**
Taxonomy:
cellular organisms | Eukaryota | Opisthokonta | Fungi | Dikarya | Basidiomycota | Pucciniomycotina | Microbotryomycetes | Sporidiobolales | mitosporic Sporidiobolales | Rhodotorula | Rhodotorula graminis
Reference/s:
DOE Joint Genome Institute: Rhodotorula graminis strain WP1 v1.1
DOE Joint Genome Institute: Rhodotorula graminis strain WP1

***Sporobolomyces roseus IAM 13481* (Spr)**
Taxonomy:
cellular organisms | Eukaryota | Opisthokonta | Fungi | Dikarya | Basidiomycota | Pucciniomycotina | Microbotryomycetes | Sporidiobolales | mitosporic Sporidiobolales | Sporobolomyces | Sporobolomyces roseus
Reference/s:
DOE Joint Genome Institute: Sporobolomyces roseus

***Melampsora laricis-populina* (Mlp)**
Taxonomy:
cellular organisms | Eukaryota | Opisthokonta | Fungi | Dikarya | Basidiomycota | Pucciniomycotina | Pucciniomycetes | Pucciniales | Melampsoraceae | Melampsora
Reference/s:
DOE Joint Genome Institute: Melampsora laricis-populina
International Species Sequencing Consortium: Rust fungi Sequencing Consortium
National Center for Biotechnology Information: NCBI Fungi Genomes Project
Publication/s:
Duplessis S *et. al.* , *Proc Natl Acad Sci U S A* , **108** , 9166 (2011).

***Phakopsora pachyrhizi* (Pkp)**
Taxonomy:
cellular organisms | Eukaryota | Opisthokonta | Fungi | Dikarya | Basidiomycota | Pucciniomycotina | Pucciniomycetes | Pucciniales | Phakopsoraceae | Phakopsora

***Puccinia graminis f. sp. tritici CRL 75-36-700-3* (Pug)**
Taxonomy:
cellular organisms | Eukaryota | Opisthokonta | Fungi | Dikarya | Basidiomycota | Pucciniomycotina | Pucciniomycetes | Pucciniales | Pucciniaceae | Puccinia | Puccinia

graminis | Puccinia graminis f. sp. tritici
Reference/s:
Broad Institute of Harvard and MIT: Puccinia Group Database
DOE Joint Genome Institute: Puccinia graminis
International Species Sequencing Consortium: Rust fungi Sequencing Consortium
National Center for Biotechnology Information: NCBI Fungi Genomes Project
e! Ensembl: Puccinia graminis f sp tritici (Puccinia graministritici)
Publication/s:
Duplessis S *et. al.* , *Proc Natl Acad Sci U S A* , **108** , 9166 (2011).

Puccinia triticina 1-1 BBBD Race 1 **(Put)**
Taxonomy:
cellular organisms | Eukaryota | Opisthokonta | Fungi | Dikarya | Basidiomycota | Pucciniomycotina | Pucciniomycetes | Pucciniales | Pucciniaceae | Puccinia | Puccinia triticina
Reference/s:
Broad Institute of Harvard and MIT: Puccinia Group Database
National Center for Biotechnology Information: NCBI Fungi Genomes Project

Malassezia globosa CBS 7966 **(Mlg)**
Taxonomy:
cellular organisms | Eukaryota | Opisthokonta | Fungi | Dikarya | Basidiomycota | Ustilaginomycotina | Exobasidiomycetes | Malasseziales | Malasseziaceae | Malassezia | Malassezia globosa
Reference/s:
DOE Joint Genome Institute: Malassezia globosa
National Center for Biotechnology Information: NCBI Fungi Genomes Project
Publication/s:
Xu J *et. al.* , *Proc Natl Acad Sci U S A* , **104** , 18730 (2007).

Malassezia restricta CBS 7877 **(Mr)**
Taxonomy:
cellular organisms | Eukaryota | Opisthokonta | Fungi | Dikarya | Basidiomycota | Ustilaginomycotina | Exobasidiomycetes | Malasseziales | Malasseziaceae | Malassezia | Malassezia restricta
Reference/s:
National Center for Biotechnology Information: NCBI Fungi Genomes Project
Publication/s:
Xu J *et. al.* , *Proc Natl Acad Sci U S A* , **104** , 18730 (2007).

Sporisorium reilianum **(Sor)**
Taxonomy:
cellular organisms | Eukaryota | Opisthokonta | Fungi | Dikarya | Basidiomycota | Ustilaginomycotina | Ustilaginomycetes | Ustilaginales | Ustilaginaceae | Sporisorium
Reference/s:
GenBank - NIH genetic sequence database: GenBank species TBLASTN WGS
International Species Sequencing Consortium: Sporisorium Sequencing Consortium
Publication/s:
Schirawski J *et. al.* , *Science* , **330** , 1546 (2010).

Ustilago maydis FB1 **(Um_b)**
Taxonomy:
cellular organisms | Eukaryota | Opisthokonta | Fungi | Dikarya | Basidiomycota | Ustilaginomycotina | Ustilaginomycetes | Ustilaginales | Ustilaginaceae | Ustilago | Ustilago maydis
Reference/s:
Broad Institute of Harvard and MIT: Ustilago maydis Database

Ustilago maydis 521 **(Um_a)**
Taxonomy:
cellular organisms | Eukaryota | Opisthokonta | Fungi | Dikarya | Basidiomycota | Ustilaginomycotina | Ustilaginomycetes | Ustilaginales | Ustilaginaceae | Ustilago | Ustilago maydis
Reference/s:
Broad Institute of Harvard and MIT: Ustilago maydis Sequencing Project
DOE Joint Genome Institute: Ustilago maydis
International Species Sequencing Consortium: Ustilago maydis Sequencing Consortium
National Center for Biotechnology Information: NCBI Fungi Genomes Project
Publication/s:
Kamper J *et. al.* , *Nature* , **444** , 97 (2006).

Cunninghamella elegans **(Cue)**
Taxonomy:
cellular organisms | Eukaryota | Opisthokonta | Fungi | Fungi incertae sedis | Early diverging fungal lineages | Mucoromycotina | Mucorales | Cunninghamellaceae | Cunninghamella
Reference/s:
Fungal Genomics Project: Cunninghamella elegans

Mucor circinelloides **(Muc)**
Taxonomy:
cellular organisms | Eukaryota | Opisthokonta | Fungi | Fungi incertae sedis | Early diverging fungal lineages | Mucoromycotina | Mucorales | Mucoraceae | Mucor
Reference/s:
DOE Joint Genome Institute: Mucor circinelloides CBS277.49 v2.0
DOE Joint Genome Institute: Mucor circinelloides

Phycomyces blakesleeanus **(Phb)**

Taxonomy:
cellular organisms | Eukaryota | Opisthokonta | Fungi | Fungi incertae sedis | Early diverging fungal lineages | Mucoromycotina | Mucorales | Mucoraceae | Phycomyces
Reference/s:
DOE Joint Genome Institute: Phycomyces blakesleeanus NRRL1555 v2.0
DOE Joint Genome Institute: Phycomyces blakesleeanus

Rhizopus arrhizus RA 99-880 **(Rha)**
Taxonomy:
cellular organisms | Eukaryota | Opisthokonta | Fungi | Fungi incertae sedis | Early diverging fungal lineages | Mucoromycotina | Mucorales | Mucoraceae | Rhizopus
Reference/s:
Broad Institute of Harvard and MIT: Rhizopus oryzae Database
GenBank - NIH genetic sequence database: GenBank species TBLASTN WGS
International Species Sequencing Consortium: Rhizopus arrihius Sequencing Consortium
TBestDB - Taxonomically Broad EST Database: Rhizopus oryzae
Publication/s:
Ma LJ *et. al.* , *PLoS Genet* , **5** , e1000549 (2009).

Glomus intraradices **(Gi)**
Taxonomy:
cellular organisms | Eukaryota | Opisthokonta | Fungi | Glomeromycota | Glomeromycetes | Glomerales | Glomeraceae | Rhizophagus

Enterocytozoon bieneusi H348 **(Enb)**
Taxonomy:
cellular organisms | Eukaryota | Opisthokonta | Fungi | Microsporidia | Apansporoblastina | Enterocytozoonidae | Enterocytozoon | Enterocytozoon bieneusi
Reference/s:
International Species Sequencing Consortium: Enterocytozoon Sequencing Consortium
MicrosporidiaDB: MicrosporidiaDB
National Center for Biotechnology Information: NCBI Fungi Genomes Project
Publication/s:
Akiyoshi DE *et. al.* , *PLoS Pathog* , **5** , e1000261 (2009).
Corradi N *et. al.* , *PLoS ONE* , **2** , e1277 (2007).
Corradi N *et. al.* , *PLoS ONE* , **2** , e1277 (2007).

Nosema ceranae BRL01 **(Nsc)**
Taxonomy:
cellular organisms | Eukaryota | Opisthokonta | Fungi | Microsporidia | Apansporoblastina | Nosematidae | Nosema | Nosema ceranae
Reference/s:
International Species Sequencing Consortium: Nosema ceranae Sequencing Consortium
MicrosporidiaDB: MicrosporidiaDB
National Center for Biotechnology Information: NCBI Fungi Genomes Project
Publication/s:
Cornman RS *et. al.* , *PLoS Pathog* , **5** , e1000466 (2009).

Encephalitozoon intestinalis **(Eni)**
Taxonomy:
cellular organisms | Eukaryota | Opisthokonta | Fungi | Microsporidia | Apansporoblastina | Unikaryonidae | Encephalitozoon
Reference/s:
International Species Sequencing Consortium: Encephalitozoon Sequencing Consortium
MicrosporidiaDB: MicrosporidiaDB
National Center for Biotechnology Information: NCBI Fungi Genomes Project
Publication/s:
Corradi N *et. al.* , *Nat Commun* , **1** , 77 (2010).

Encephalitozoon cuniculi GB-M1 **(Ec)**
Taxonomy:
cellular organisms | Eukaryota | Opisthokonta | Fungi | Microsporidia | Apansporoblastina | Unikaryonidae | Encephalitozoon | Encephalitozoon cuniculi
Reference/s:
International Species Sequencing Consortium: Genoscope WGS project
MicrosporidiaDB: MicrosporidiaDB
National Center for Biotechnology Information: NCBI Fungi Genomes Project
Publication/s:
Katinka MD *et. al.* , *Nature* , **414** , 450 (2001).

Nematocida parisii ERTm1 **(Nep)**
Taxonomy:
cellular organisms | Eukaryota | Opisthokonta | Fungi | Microsporidia | Microsporidia incertae sedis | Nematocida | Nematocida parisii
Reference/s:
Broad Institute of Harvard and MIT: Microsporidia Comparative Database
MicrosporidiaDB: MicrosporidiaDB
National Center for Biotechnology Information: NCBI Fungi Genomes Project

Octosporea bayeri OER-3-3 **(Ocb)**
Taxonomy:
cellular organisms | Eukaryota | Opisthokonta | Fungi | Microsporidia | Pansporoblastina | Dubosqiidae | Hamiltosporidium | Hamiltosporidium tvaerminnensis
Reference/s:
International Species Sequencing Consortium: Octosporea Sequencing Consortium
MicrosporidiaDB: MicrosporidiaDB
National Center for Biotechnology Information: NCBI Fungi Genomes Project
Publication/s:
Corradi N *et. al.* , *Genome Biol* , **10** , R106 (2009).

***Echinococcus multilocularis* (Em)**
Taxonomy:
cellular organisms | Eukaryota | Opisthokonta | Metazoa | Eumetazoa | Bilateria | Acoelomata | Platyhelminthes | Cestoda | Eucestoda | Cyclophyllidea | Taeniidae | Echinococcus
Reference/s:
The Wellcome Trust Sanger Institute: Echinococcus Genome Project

***Echinococcus granulosus* (Ecg)**
Taxonomy:
cellular organisms | Eukaryota | Opisthokonta | Metazoa | Eumetazoa | Bilateria | Acoelomata | Platyhelminthes | Cestoda | Eucestoda | Cyclophyllidea | Taeniidae | Echinococcus
Reference/s:
GenBank - NIH genetic sequence database: GenBank species TBLASTN
The Wellcome Trust Sanger Institute: Echinococcus granulosus
Publication/s:
Fernandez C *et. al.* , *Mol Biochem Parasitol* , **122** , 171 (2002).

***Taenia solium* (Tas)**
Taxonomy:
cellular organisms | Eukaryota | Opisthokonta | Metazoa | Eumetazoa | Bilateria | Acoelomata | Platyhelminthes | Cestoda | Eucestoda | Cyclophyllidea | Taeniidae | Taenia

***Schistosoma mansoni* (Sm)**
Taxonomy:
cellular organisms | Eukaryota | Opisthokonta | Metazoa | Eumetazoa | Bilateria | Acoelomata | Platyhelminthes | Trematoda | Digenea | Strigeidida | Schistosomatoidea | Schistosomatidae | Schistosoma
Reference/s:
GenBank - NIH genetic sequence database: GenBank species TBLASTN
International Species Sequencing Consortium: Schistosoma Sequencing Consortium
National Center for Biotechnology Information: NCBI Eukaryotic Genomes Project
SchistoDB: Schistosoma Genomic Resources
The Gene Index Project: DFCI Schistosoma mansoni Gene Index
The Institute for Genomic Research: The TIGR Schistosoma mansoni Genome Project
The Wellcome Trust Sanger Institute: The Schistosoma mansoni Genome Project
Publication/s:
Berriman M *et. al.* , *Nature* , **460** , 352 (2009).
Dillon GP *et. al.* , *Int J Parasitol* , **36** , 1 (2006).
Verjovski-Almeida S *et. al.* , *Nat Genet* , **35** , 148 (2003).

***Schistosoma japonicum* (Shj)**
Taxonomy:
cellular organisms | Eukaryota | Opisthokonta | Metazoa | Eumetazoa | Bilateria | Acoelomata | Platyhelminthes | Trematoda | Digenea | Strigeidida | Schistosomatoidea | Schistosomatidae | Schistosoma
Reference/s:
GenBank - NIH genetic sequence database: GenBank species TBLASTN
International Species Sequencing Consortium: Schistosoma japonicum Sequencing Consortium
National Center for Biotechnology Information: NCBI Eukaryotic Genomes Project
SchistoDB: Schistosoma Genomic Resources
Publication/s:
Zhou Y *et. al.* , *Nature* , **460** , 345 (2009).
Liu F *et. al.* , *PLoS Pathog* , **2** , e29 (2006).
Hu W *et. al.* , *Nat Genet* , **35** , 139 (2003).

***Convoluta pulchra* (Cnp)**
Taxonomy:
cellular organisms | Eukaryota | Opisthokonta | Metazoa | Eumetazoa | Bilateria | Acoelomata | Platyhelminthes | Turbellaria | Acoelomorpha | Acoela | Convolutidae | Convoluta

***Dugesia japonica* (Dj)**
Taxonomy:
cellular organisms | Eukaryota | Opisthokonta | Metazoa | Eumetazoa | Bilateria | Acoelomata | Platyhelminthes | Turbellaria | Seriata | Tricladida | Paludicola | Dugesiidae | Dugesia
Reference/s:
GenBank - NIH genetic sequence database: GenBank species TBLASTN
Publication/s:
Mineta K *et. al.* , *Proc Natl Acad Sci U S A* , **100** , 7666 (2003).

***Schmidtea mediterranea str. S2F2* (Scm)**
Taxonomy:
cellular organisms | Eukaryota | Opisthokonta | Metazoa | Eumetazoa | Bilateria | Acoelomata | Platyhelminthes | Turbellaria | Seriata | Tricladida | Paludicola | Dugesiidae | Schmidtea
Reference/s:
GenBank - NIH genetic sequence database: GenBank species TBLASTN WGS
GenBank - NIH genetic sequence database: GenBank species TBLASTN
SmedGD: SmedGD: the Schmidtea mediterranea Genome Database
The Genome Sequencing Center at Washington University: Schmidtea mediterranea
Publication/s:
Zayas RM *et. al.* , *Proc Natl Acad Sci U S A* , **102** , 18491 (2005).

***Branchiostoma floridae* (Bf)**
Taxonomy:

cellular organisms | Eukaryota | Opisthokonta | Metazoa | Eumetazoa | Bilateria | Coelomata | Deuterostomia | Chordata | Cephalochordata | Branchiostomidae | Branchiostoma
Reference/s:
DOE Joint Genome Institute: Branchiostoma floridae
GenBank - NIH genetic sequence database: GenBank species TBLASTN
International Species Sequencing Consortium: Branchiostoma floridae Sequencing Consortium
National Center for Biotechnology Information: NCBI Eukaryotic Genomes Project
UCSC Genome Bioinformatics: Lancelet (Branchiostoma floridae) Genome Browser Gateway
Publication/s:
Putnam NH *et. al.* , *Nature* , **453** , 1064 (2008).
Yu JK *et. al.* , *Nature* , **445** , 613 (2007).
Panopoulou G *et. al.* , *Genome Res* , **13** , 1056 (2003).

***Eptatretus burgeri* (Eb)**
Taxonomy:
cellular organisms | Eukaryota | Opisthokonta | Metazoa | Eumetazoa | Bilateria | Coelomata | Deuterostomia | Chordata | Craniata | Hyperotreti | Myxiniformes | Myxinidae | Eptatretinae | Eptatretus
Reference/s:
GenBank - NIH genetic sequence database: GenBank species TBLASTN
Publication/s:
Suzuki T *et. al.* , *Dev Comp Immunol* , **28** , 993 (2004).

***Leucoraja erinacea* (Lee)**
Taxonomy:
cellular organisms | Eukaryota | Opisthokonta | Metazoa | Eumetazoa | Bilateria | Coelomata | Deuterostomia | Chordata | Craniata | Vertebrata | Gnathostomata | Chondrichthyes | Elasmobranchii | Batoidea | Rajiformes | Rajidae | Leucoraja
Reference/s:
International Species Sequencing Consortium: Leucoraja erinacea Sequencing Consortium
National Center for Biotechnology Information: NCBI Eukaryotic Genomes Project
The Marine Genomics Project: Leucoraja erinacea

***Triakis scyllium* (Tis)**
Taxonomy:
cellular organisms | Eukaryota | Opisthokonta | Metazoa | Eumetazoa | Bilateria | Coelomata | Deuterostomia | Chordata | Craniata | Vertebrata | Gnathostomata | Chondrichthyes | Elasmobranchii | Selachii | Galeomorphii | Galeoidea | Carcharhiniformes | Triakidae | Triakis

***Squalus acanthias* (Sqa)**
Taxonomy:
cellular organisms | Eukaryota | Opisthokonta | Metazoa | Eumetazoa | Bilateria | Coelomata | Deuterostomia | Chordata | Craniata | Vertebrata | Gnathostomata | Chondrichthyes | Elasmobranchii | Selachii | Squalimorphii | Squaliformes | Squalidae | Squalus
Reference/s:
The Marine Genomics Project: Squalus acanthias

***Callorhinchus milii* (Clm)**
Taxonomy:
cellular organisms | Eukaryota | Opisthokonta | Metazoa | Eumetazoa | Bilateria | Coelomata | Deuterostomia | Chordata | Craniata | Vertebrata | Gnathostomata | Chondrichthyes | Holocephali | Chimaeriformes | Callorhinchidae | Callorhinchus
Reference/s:
National Center for Biotechnology Information: NCBI Eukaryotic Genomes Project
Singapore Institute of Molecular and Cell Biology: Elephant Shark Genome Project Webpage
Publication/s:
Venkatesh B *et. al.* , *PLoS Biol* , **5** , e101 (2007).

***Haplochromis burtoni* (Hab)**
Taxonomy:
cellular organisms | Eukaryota | Opisthokonta | Metazoa | Eumetazoa | Bilateria | Coelomata | Deuterostomia | Chordata | Craniata | Vertebrata | Gnathostomata | Teleostomi | Euteleostomi | Actinopterygii | Actinopteri | Neopterygii | Teleostei | Elopocephala | Clupeocephala | Euteleostei | Neognathi | Neoteleostei | Eurypterygii | Ctenosquamata | Acanthomorpha | Euacanthomorpha | Holacanthopterygii | Acanthopterygii | Euacanthopterygii | Percomorpha | Perciformes | Labroidei | Cichlidae | African cichlids | Pseudocrenilabrinae | Haplochromini | Haplochromis
Reference/s:
Broad Institute of Harvard and MIT: Tilapia Genome Project
The Gene Index Project: DFCI Astatotilapia burtoni Gene Index

***Labeotropheus fuelleborni str. Domwe Island* (Lf)**
Taxonomy:
cellular organisms | Eukaryota | Opisthokonta | Metazoa | Eumetazoa | Bilateria | Coelomata | Deuterostomia | Chordata | Craniata | Vertebrata | Gnathostomata | Teleostomi | Euteleostomi | Actinopterygii | Actinopteri | Neopterygii | Teleostei | Elopocephala | Clupeocephala | Euteleostei | Neognathi | Neoteleostei | Eurypterygii | Ctenosquamata | Acanthomorpha | Euacanthomorpha | Holacanthopterygii | Acanthopterygii | Euacanthopterygii | Percomorpha | Perciformes | Labroidei | Cichlidae | African cichlids | Pseudocrenilabrinae | Haplochromini | Labeotropheus
Reference/s:
National Center for Biotechnology Information: NCBI Eukaryotic Genomes Project

***Lipochromis sp. 'matumbi hunter'* (Lsmh)**
Taxonomy:
cellular organisms | Eukaryota | Opisthokonta | Metazoa | Eumetazoa | Bilateria | Coelomata | Deuterostomia | Chordata | Craniata | Vertebrata | Gnathostomata | Teleostomi | Euteleostomi | Actinopterygii | Actinopteri | Neopterygii | Teleostei | Elopocephala | Clupeocephala | Euteleostei | Neognathi | Neoteleostei | Eurypterygii | Ctenosquamata | Acanthomorpha | Euacanthomorpha | Holacanthopterygii | Acanthopterygii | Euacanthopterygii | Percomorpha | Perciformes | Labroidei | Cichlidae | African cichlids | Pseudocrenilabrinae | Haplochromini | Lipochromis

***Maylandia zebra str. Mazinzi Reef* (Mz)**

Taxonomy:
cellular organisms | Eukaryota | Opisthokonta | Metazoa | Eumetazoa | Bilateria | Coelomata | Deuterostomia | Chordata | Craniata | Vertebrata | Gnathostomata | Teleostomi | Euteleostomi | Actinopterygii | Actinopteri | Neopterygii | Teleostei | Elopocephala | Clupeocephala | Euteleostei | Neognathi | Neoteleostei | Eurypterygii | Ctenosquamata | Acanthomorpha | Euacanthomorpha | Holacanthopterygii | Acanthopterygii | Euacanthopterygii | Percomorpha | Perciformes | Labroidei | Cichlidae | African cichlids | Pseudocrenilabrinae | Haplochromini | Maylandia | Maylandia zebra complex
Reference/s:
National Center for Biotechnology Information: NCBI Eukaryotic Genomes Project

***Melanochromis auratus str. Domwe Island* (Mla)**
Taxonomy:
cellular organisms | Eukaryota | Opisthokonta | Metazoa | Eumetazoa | Bilateria | Coelomata | Deuterostomia | Chordata | Craniata | Vertebrata | Gnathostomata | Teleostomi | Euteleostomi | Actinopterygii | Actinopteri | Neopterygii | Teleostei | Elopocephala | Clupeocephala | Euteleostei | Neognathi | Neoteleostei | Eurypterygii | Ctenosquamata | Acanthomorpha | Euacanthomorpha | Holacanthopterygii | Acanthopterygii | Euacanthopterygii | Percomorpha | Perciformes | Labroidei | Cichlidae | African cichlids | Pseudocrenilabrinae | Haplochromini | Melanochromis
Reference/s:
National Center for Biotechnology Information: NCBI Eukaryotic Genomes Project

***Paralabidochromis chilotes* (Pac)**
Taxonomy:
cellular organisms | Eukaryota | Opisthokonta | Metazoa | Eumetazoa | Bilateria | Coelomata | Deuterostomia | Chordata | Craniata | Vertebrata | Gnathostomata | Teleostomi | Euteleostomi | Actinopterygii | Actinopteri | Neopterygii | Teleostei | Elopocephala | Clupeocephala | Euteleostei | Neognathi | Neoteleostei | Eurypterygii | Ctenosquamata | Acanthomorpha | Euacanthomorpha | Holacanthopterygii | Acanthopterygii | Euacanthopterygii | Percomorpha | Perciformes | Labroidei | Cichlidae | African cichlids | Pseudocrenilabrinae | Haplochromini | Paralabidochromis

***Ptyochromis sp. 'redtail sheller'* (Psrs)**
Taxonomy:
cellular organisms | Eukaryota | Opisthokonta | Metazoa | Eumetazoa | Bilateria | Coelomata | Deuterostomia | Chordata | Craniata | Vertebrata | Gnathostomata | Teleostomi | Euteleostomi | Actinopterygii | Actinopteri | Neopterygii | Teleostei | Elopocephala | Clupeocephala | Euteleostei | Neognathi | Neoteleostei | Eurypterygii | Ctenosquamata | Acanthomorpha | Euacanthomorpha | Holacanthopterygii | Acanthopterygii | Euacanthopterygii | Percomorpha | Perciformes | Labroidei | Cichlidae | African cichlids | Pseudocrenilabrinae | Haplochromini | Ptyochromis

***Oreochromis niloticus* (Orn)**
Taxonomy:
cellular organisms | Eukaryota | Opisthokonta | Metazoa | Eumetazoa | Bilateria | Coelomata | Deuterostomia | Chordata | Craniata | Vertebrata | Gnathostomata | Teleostomi | Euteleostomi | Actinopterygii | Actinopteri | Neopterygii | Teleostei | Elopocephala | Clupeocephala | Euteleostei | Neognathi | Neoteleostei | Eurypterygii | Ctenosquamata | Acanthomorpha | Euacanthomorpha | Holacanthopterygii | Acanthopterygii | Euacanthopterygii | Percomorpha | Perciformes | Labroidei | Cichlidae | African cichlids | Pseudocrenilabrinae | Tilapiini | Oreochromis
Reference/s:
Broad Institute of Harvard and MIT: Tilapia Genome Project
National Center for Biotechnology Information: NCBI Eukaryotic Genomes Project

***Perca flavescens* (Prf)**
Taxonomy:
cellular organisms | Eukaryota | Opisthokonta | Metazoa | Eumetazoa | Bilateria | Coelomata | Deuterostomia | Chordata | Craniata | Vertebrata | Gnathostomata | Teleostomi | Euteleostomi | Actinopterygii | Actinopteri | Neopterygii | Teleostei | Elopocephala | Clupeocephala | Euteleostei | Neognathi | Neoteleostei | Eurypterygii | Ctenosquamata | Acanthomorpha | Euacanthomorpha | Holacanthopterygii | Acanthopterygii | Euacanthopterygii | Percomorpha | Perciformes | Percoidei | Percidae | Percinae | Perca

***Lithognathus mormyrus* (Lim)**
Taxonomy:
cellular organisms | Eukaryota | Opisthokonta | Metazoa | Eumetazoa | Bilateria | Coelomata | Deuterostomia | Chordata | Craniata | Vertebrata | Gnathostomata | Teleostomi | Euteleostomi | Actinopterygii | Actinopteri | Neopterygii | Teleostei | Elopocephala | Clupeocephala | Euteleostei | Neognathi | Neoteleostei | Eurypterygii | Ctenosquamata | Acanthomorpha | Euacanthomorpha | Holacanthopterygii | Acanthopterygii | Euacanthopterygii | Percomorpha | Perciformes | Percoidei | Sparidae | Lithognathus

***Sparus aurata* (Sa)**
Taxonomy:
cellular organisms | Eukaryota | Opisthokonta | Metazoa | Eumetazoa | Bilateria | Coelomata | Deuterostomia | Chordata | Craniata | Vertebrata | Gnathostomata | Teleostomi | Euteleostomi | Actinopterygii | Actinopteri | Neopterygii | Teleostei | Elopocephala | Clupeocephala | Euteleostei | Neognathi | Neoteleostei | Eurypterygii | Ctenosquamata | Acanthomorpha | Euacanthomorpha | Holacanthopterygii | Acanthopterygii | Euacanthopterygii | Percomorpha | Perciformes | Percoidei | Sparidae | Sparus

***Thunnus thynnus* (Tht)**
Taxonomy:
cellular organisms | Eukaryota | Opisthokonta | Metazoa | Eumetazoa | Bilateria | Coelomata | Deuterostomia | Chordata | Craniata | Vertebrata | Gnathostomata | Teleostomi | Euteleostomi | Actinopterygii | Actinopteri | Neopterygii | Teleostei | Elopocephala | Clupeocephala | Euteleostei | Neognathi | Neoteleostei | Eurypterygii | Ctenosquamata | Acanthomorpha | Euacanthomorpha | Holacanthopterygii | Acanthopterygii | Euacanthopterygii | Percomorpha | Perciformes | Scombroidei | Scombridae | Scombrinae | Thunnini | Thunnus

***Hippoglossus hippoglossus* (Hh)**
Taxonomy:
cellular organisms | Eukaryota | Opisthokonta | Metazoa | Eumetazoa | Bilateria | Coelomata | Deuterostomia | Chordata | Craniata | Vertebrata | Gnathostomata | Teleostomi | Euteleostomi | Actinopterygii | Actinopteri | Neopterygii | Teleostei | Elopocephala | Clupeocephala | Euteleostei | Neognathi | Neoteleostei | Eurypterygii | Ctenosquamata | Acanthomorpha | Euacanthomorpha | Holacanthopterygii | Acanthopterygii | Euacanthopterygii | Percomorpha | Pleuronectiformes | Pleuronectoidei | Pleuronectidae | Pleuronectinae | Hippoglossus

***Anoplopoma fimbria* (Anf)**
Taxonomy:
cellular organisms | Eukaryota | Opisthokonta | Metazoa | Eumetazoa | Bilateria | Coelomata | Deuterostomia | Chordata | Craniata | Vertebrata | Gnathostomata | Teleostomi | Euteleostomi | Actinopterygii | Actinopteri | Neopterygii | Teleostei | Elopocephala | Clupeocephala | Euteleostei | Neognathi | Neoteleostei | Eurypterygii | Ctenosquamata | Acanthomorpha | Euacanthomorpha | Holacanthopterygii | Acanthopterygii | Euacanthopterygii | Percomorpha | Scorpaeniformes | Anoplopomatoidei | Anoplopomatidae | Anoplopoma

***Oryzias latipes str. Hd-rR* (Ol_a)**

Taxonomy:
cellular organisms | Eukaryota | Opisthokonta | Metazoa | Eumetazoa | Bilateria | Coelomata | Deuterostomia | Chordata | Craniata | Vertebrata | Gnathostomata | Teleostomi | Euteleostomi | Actinopterygii | Actinopteri | Neopterygii | Teleostei | Elopocephala | Clupeocephala | Euteleostei | Neognathi | Neoteleostei | Eurypterygii | Ctenosquamata | Acanthomorpha | Euacanthomorpha | Holacanthopterygii | Acanthopterygii | Euacanthopterygii | Percomorpha | Smegmamorpha | Atherinomorpha | Beloniformes | Adrianichthyoidei | Adrianichthyidae | Oryziinae | Oryzias
Reference/s:
GenBank - NIH genetic sequence database: GenBank species TBLASTN
NIG DNA Sequencing Center: Medaka Genome Sequencing Project
National Center for Biotechnology Information: NCBI Eukaryotic Genomes Project
The Gene Index Project: DFCI Oryzias latipes Gene Index
UCSC Genome Bioinformatics: Medaka (Oryzias latipes) Genome Browser Gateway
e! Ensembl: Medaka
Publication/s:
Kasahara M *et. al.* , *Nature* , **447** , 714 (2007).
Kimura T *et. al.* , *Mech Dev* , **121** , 915 (2004).

Oryzias latipes str. HNI **(Ol_b)**
Taxonomy:
cellular organisms | Eukaryota | Opisthokonta | Metazoa | Eumetazoa | Bilateria | Coelomata | Deuterostomia | Chordata | Craniata | Vertebrata | Gnathostomata | Teleostomi | Euteleostomi | Actinopterygii | Actinopteri | Neopterygii | Teleostei | Elopocephala | Clupeocephala | Euteleostei | Neognathi | Neoteleostei | Eurypterygii | Ctenosquamata | Acanthomorpha | Euacanthomorpha | Holacanthopterygii | Acanthopterygii | Euacanthopterygii | Percomorpha | Smegmamorpha | Atherinomorpha | Beloniformes | Adrianichthyoidei | Adrianichthyidae | Oryziinae | Oryzias
Reference/s:
GenBank - NIH genetic sequence database: GenBank species TBLASTN
National Center for Biotechnology Information: NCBI Eukaryotic Genomes Project
The Gene Index Project: DFCI Oryzias latipes Gene Index
Publication/s:
Kasahara M *et. al.* , *Nature* , **447** , 714 (2007).
Kimura T *et. al.* , *Mech Dev* , **121** , 915 (2004).

Fundulus heteroclitus **(Fh)**
Taxonomy:
cellular organisms | Eukaryota | Opisthokonta | Metazoa | Eumetazoa | Bilateria | Coelomata | Deuterostomia | Chordata | Craniata | Vertebrata | Gnathostomata | Teleostomi | Euteleostomi | Actinopterygii | Actinopteri | Neopterygii | Teleostei | Elopocephala | Clupeocephala | Euteleostei | Neognathi | Neoteleostei | Eurypterygii | Ctenosquamata | Acanthomorpha | Euacanthomorpha | Holacanthopterygii | Acanthopterygii | Euacanthopterygii | Percomorpha | Smegmamorpha | Atherinomorpha | Cyprinodontiformes | Cyprinodontoidei | Fundulidae | Fundulus
Reference/s:
FunnyBase Expressed Gene Database: Annotated Fundulus Heteroclitus EST Gene Expression Database
The Gene Index Project: DFCI Fundulus heteroclitus (Killifish) Gene Index
The Marine Genomics Project: Fundulus species
Publication/s:
Paschall JE *et. al.* , *BMC Genomics* , **5** , 96 (2004).

Poecilia reticulata **(Por)**
Taxonomy:
cellular organisms | Eukaryota | Opisthokonta | Metazoa | Eumetazoa | Bilateria | Coelomata | Deuterostomia | Chordata | Craniata | Vertebrata | Gnathostomata | Teleostomi | Euteleostomi | Actinopterygii | Actinopteri | Neopterygii | Teleostei | Elopocephala | Clupeocephala | Euteleostei | Neognathi | Neoteleostei | Eurypterygii | Ctenosquamata | Acanthomorpha | Euacanthomorpha | Holacanthopterygii | Acanthopterygii | Euacanthopterygii | Percomorpha | Smegmamorpha | Atherinomorpha | Cyprinodontiformes | Cyprinodontoidei | Poeciliidae | Poeciliinae | Poecilia

Xiphophorus maculatus x Xiphophorus helleri **(Xx)**
Taxonomy:
cellular organisms | Eukaryota | Opisthokonta | Metazoa | Eumetazoa | Bilateria | Coelomata | Deuterostomia | Chordata | Craniata | Vertebrata | Gnathostomata | Teleostomi | Euteleostomi | Actinopterygii | Actinopteri | Neopterygii | Teleostei | Elopocephala | Clupeocephala | Euteleostei | Neognathi | Neoteleostei | Eurypterygii | Ctenosquamata | Acanthomorpha | Euacanthomorpha | Holacanthopterygii | Acanthopterygii | Euacanthopterygii | Percomorpha | Smegmamorpha | Atherinomorpha | Cyprinodontiformes | Cyprinodontoidei | Poeciliidae | Poeciliinae | Xiphophorus

Gasterosteus aculeatus **(Ga)**
Taxonomy:
cellular organisms | Eukaryota | Opisthokonta | Metazoa | Eumetazoa | Bilateria | Coelomata | Deuterostomia | Chordata | Craniata | Vertebrata | Gnathostomata | Teleostomi | Euteleostomi | Actinopterygii | Actinopteri | Neopterygii | Teleostei | Elopocephala | Clupeocephala | Euteleostei | Neognathi | Neoteleostei | Eurypterygii | Ctenosquamata | Acanthomorpha | Euacanthomorpha | Holacanthopterygii | Acanthopterygii | Euacanthopterygii | Percomorpha | Smegmamorpha | Gasterosteiformes/Syngnathiformes group | Gasterosteiformes | Gasterosteidae | Gasterosteus
Reference/s:
Broad Institute of Harvard and MIT: Stickleback Genome Sequencing Project
National Center for Biotechnology Information: NCBI Eukaryotic Genomes Project
UCSC Genome Bioinformatics: Stickleback (Gasterosteus aculeatus) Genome Browser Gateway
e! Ensembl: Stickleback

Takifugu rubripes **(Tar)**
Taxonomy:
cellular organisms | Eukaryota | Opisthokonta | Metazoa | Eumetazoa | Bilateria | Coelomata | Deuterostomia | Chordata | Craniata | Vertebrata | Gnathostomata | Teleostomi | Euteleostomi | Actinopterygii | Actinopteri | Neopterygii | Teleostei | Elopocephala | Clupeocephala | Euteleostei | Neognathi | Neoteleostei | Eurypterygii | Ctenosquamata | Acanthomorpha | Euacanthomorpha | Holacanthopterygii | Acanthopterygii | Euacanthopterygii | Percomorpha | Tetraodontiformes | Tetraodontoidei | Tetradontoidea | Tetraodontidae | Takifugu
Reference/s:
DOE Joint Genome Institute: Fugu rubripes
GenBank - NIH genetic sequence database: GenBank species TBLASTN
International Species Sequencing Consortium: Takifugu rubripes Sequencing Consortium

National Center for Biotechnology Information: NCBI Eukaryotic Genomes Project
School of Biological & Chemical Sciences: The Fugu Genomics Project
Singapore Institute of Molecular and Cell Biology: Fugu Genome Project
The Gene Index Project: DFCI Takifugu Gene Index
UCSC Genome Bioinformatics: Takifugu rubripes Genome Browser Gateway
e! Ensembl: Fugu
Publication/s:
Kai W *et. al.* , *Genome Biol Evol* , **3** , 424 (2011).
Clark MS *et. al.* , *Genome Res* , **13** , 2747 (2003).
Aparicio S *et. al.* , *Science* , **297** , 1301 (2002).

***Tetraodon nigroviridis* (Tn)**
Taxonomy:
cellular organisms | Eukaryota | Opisthokonta | Metazoa | Eumetazoa | Bilateria | Coelomata | Deuterostomia | Chordata | Craniata | Vertebrata | Gnathostomata | Teleostomi | Euteleostomi | Actinopterygii | Actinopteri | Neopterygii | Teleostei | Elopocephala | Clupeocephala | Euteleostei | Neognathi | Neoteleostei | Eurypterygii | Ctenosquamata | Acanthomorpha | Euacanthomorpha | Holacanthopterygii | Acanthopterygii | Euacanthopterygii | Percomorpha | Tetraodontiformes | Tetraodontoidei | Tetradontoidea | Tetraodontidae | Tetraodon
Reference/s:
Broad Institute of Harvard and MIT: Tetraodon nigroviridis Database
Genoscope: Tetraodon nigroviridis - A fish with a compact genome
International Species Sequencing Consortium: Tetraodon nigroviridis Sequencing Consortium
National Center for Biotechnology Information: NCBI Eukaryotic Genomes Project
UCSC Genome Bioinformatics: Tetraodon nigroviridis Genome Browser Gateway
e! Ensembl: Tetraodon
Publication/s:
Jaillon O *et. al.* , *Nature* , **431** , 946 (2004).

***Gadus morhua* (Gm)**
Taxonomy:
cellular organisms | Eukaryota | Opisthokonta | Metazoa | Eumetazoa | Bilateria | Coelomata | Deuterostomia | Chordata | Craniata | Vertebrata | Gnathostomata | Teleostomi | Euteleostomi | Actinopterygii | Actinopteri | Neopterygii | Teleostei | Elopocephala | Clupeocephala | Euteleostei | Neognathi | Neoteleostei | Eurypterygii | Ctenosquamata | Acanthomorpha | Euacanthomorpha | Holacanthopterygii | Paracanthopterygii | Gadiformes | Gadidae | Gadus
Reference/s:
Codgenome: The cod genome project
International Species Sequencing Consortium: Atlantic Cod Sequencing Consortium
National Center for Biotechnology Information: NCBI Eukaryotic Genomes Project
Publication/s:
Star B *et. al.* , *Nature* , **477** , 207 (2011).

***Osmerus mordax* (Osm)**
Taxonomy:
cellular organisms | Eukaryota | Opisthokonta | Metazoa | Eumetazoa | Bilateria | Coelomata | Deuterostomia | Chordata | Craniata | Vertebrata | Gnathostomata | Teleostomi | Euteleostomi | Actinopterygii | Actinopteri | Neopterygii | Teleostei | Elopocephala | Clupeocephala | Euteleostei | Protacanthopterygii | Osmeriformes | Osmeroidei | Osmeroidea | Osmeridae | Osmerinae | Osmerini | Osmerus

***Oncorhynchus mykiss* (Om)**
Taxonomy:
cellular organisms | Eukaryota | Opisthokonta | Metazoa | Eumetazoa | Bilateria | Coelomata | Deuterostomia | Chordata | Craniata | Vertebrata | Gnathostomata | Teleostomi | Euteleostomi | Actinopterygii | Actinopteri | Neopterygii | Teleostei | Elopocephala | Clupeocephala | Euteleostei | Protacanthopterygii | Salmoniformes | Salmonoidei | Salmonidae | Salmoninae | Oncorhynchus
Reference/s:
GenBank - NIH genetic sequence database: GenBank species TBLASTN
The Gene Index Project: DFCI Oncorhynchus mykiss (Rainbow trout) Gene Index
Publication/s:
Rise ML *et. al.* , *Genome Res* , **14** , 478 (2004).
Rexroad CE 3rd *et. al.* , *Cytogenet Genome Res* , **102** , 347 (2003).

***Oncorhynchus nerka* (On)**
Taxonomy:
cellular organisms | Eukaryota | Opisthokonta | Metazoa | Eumetazoa | Bilateria | Coelomata | Deuterostomia | Chordata | Craniata | Vertebrata | Gnathostomata | Teleostomi | Euteleostomi | Actinopterygii | Actinopteri | Neopterygii | Teleostei | Elopocephala | Clupeocephala | Euteleostei | Protacanthopterygii | Salmoniformes | Salmonoidei | Salmonidae | Salmoninae | Oncorhynchus

***Salmo salar* (Sas)**
Taxonomy:
cellular organisms | Eukaryota | Opisthokonta | Metazoa | Eumetazoa | Bilateria | Coelomata | Deuterostomia | Chordata | Craniata | Vertebrata | Gnathostomata | Teleostomi | Euteleostomi | Actinopterygii | Actinopteri | Neopterygii | Teleostei | Elopocephala | Clupeocephala | Euteleostei | Protacanthopterygii | Salmoniformes | Salmonoidei | Salmonidae | Salmoninae | Salmo
Reference/s:
GenBank - NIH genetic sequence database: GenBank species TBLASTN
National Center for Biotechnology Information: NCBI Eukaryotic Genomes Project
The Gene Index Project: DFCI Salmo salar Gene Index
Publication/s:
Rise ML *et. al.* , *Genome Res* , **14** , 478 (2004).

***Salvelinus fontinalis* (Saf)**
Taxonomy:
cellular organisms | Eukaryota | Opisthokonta | Metazoa | Eumetazoa | Bilateria | Coelomata | Deuterostomia | Chordata | Craniata | Vertebrata | Gnathostomata | Teleostomi | Euteleostomi | Actinopterygii | Actinopteri | Neopterygii | Teleostei | Elopocephala | Clupeocephala | Euteleostei | Protacanthopterygii | Salmoniformes | Salmonoidei | Salmonidae | Salmoninae | Salvelinus

***Cyprinus carpio* (Cyc)**
Taxonomy:
cellular organisms | Eukaryota | Opisthokonta | Metazoa | Eumetazoa | Bilateria | Coelomata | Deuterostomia | Chordata | Craniata | Vertebrata | Gnathostomata | Teleostomi | Euteleostomi | Actinopterygii | Actinopteri | Neopterygii | Teleostei | Elopocephala | Clupeocephala | Otocephala | Ostariophysi | Otophysi | Cypriniphysi | Cypriniformes | Cyprinoidea | Cyprinidae | Cyprinus
Reference/s:
GenBank - NIH genetic sequence database: GenBank species TBLASTN
Publication/s:
Gracey AY *et. al.* , *Proc Natl Acad Sci U S A* , **101** , 16970 (2004).

***Brachydanio rerio str. Tuebingen* (Br)**
Taxonomy:
cellular organisms | Eukaryota | Opisthokonta | Metazoa | Eumetazoa | Bilateria | Coelomata | Deuterostomia | Chordata | Craniata | Vertebrata | Gnathostomata | Teleostomi | Euteleostomi | Actinopterygii | Actinopteri | Neopterygii | Teleostei | Elopocephala | Clupeocephala | Otocephala | Ostariophysi | Otophysi | Cypriniphysi | Cypriniformes | Cyprinoidea | Cyprinidae | Danio
Reference/s:
GenBank - NIH genetic sequence database: GenBank species TBLASTN
National Center for Biotechnology Information: Zebrafish Sequencing Project
National Center for Biotechnology Information: Zebrafish Gene Collection
National Center for Biotechnology Information Reference Sequences: Danio rerio (zebrafish) genome view
The Gene Index Project: DFCI Zebrafish (Danio rerio) Gene Index
The Wellcome Trust Sanger Institute: The Danio rerio Sequencing Project
UCSC Genome Bioinformatics: Danio rerio Genome Browser Gateway
ZFIN The Zebrafish Information Network: The Zebrafish Model Organism Database
e! Ensembl: Zebrafish
Publication/s:
Gerhard DS *et. al.* , *Genome Res* , **14** , 2121 (2004).
Lo J *et. al.* , *Genome Res* , **13** , 455 (2003).
Strausberg RL *et. al.* , *Proc Natl Acad Sci U S A* , **99** , 16899 (2002).

***Gobiocypris rarus* (Gbr)**
Taxonomy:
cellular organisms | Eukaryota | Opisthokonta | Metazoa | Eumetazoa | Bilateria | Coelomata | Deuterostomia | Chordata | Craniata | Vertebrata | Gnathostomata | Teleostomi | Euteleostomi | Actinopterygii | Actinopteri | Neopterygii | Teleostei | Elopocephala | Clupeocephala | Otocephala | Ostariophysi | Otophysi | Cypriniphysi | Cypriniformes | Cyprinoidea | Cyprinidae | Gobiocypris

***Pimephales promelas* (Pip)**
Taxonomy:
cellular organisms | Eukaryota | Opisthokonta | Metazoa | Eumetazoa | Bilateria | Coelomata | Deuterostomia | Chordata | Craniata | Vertebrata | Gnathostomata | Teleostomi | Euteleostomi | Actinopterygii | Actinopteri | Neopterygii | Teleostei | Elopocephala | Clupeocephala | Otocephala | Ostariophysi | Otophysi | Cypriniphysi | Cypriniformes | Cyprinoidea | Cyprinidae | Pimephales

***Rutilus rutilus* (Rr)**
Taxonomy:
cellular organisms | Eukaryota | Opisthokonta | Metazoa | Eumetazoa | Bilateria | Coelomata | Deuterostomia | Chordata | Craniata | Vertebrata | Gnathostomata | Teleostomi | Euteleostomi | Actinopterygii | Actinopteri | Neopterygii | Teleostei | Elopocephala | Clupeocephala | Otocephala | Ostariophysi | Otophysi | Cypriniphysi | Cypriniformes | Cyprinoidea | Cyprinidae | Rutilus

***Ictalurus punctatus* (Ip)**
Taxonomy:
cellular organisms | Eukaryota | Opisthokonta | Metazoa | Eumetazoa | Bilateria | Coelomata | Deuterostomia | Chordata | Craniata | Vertebrata | Gnathostomata | Teleostomi | Euteleostomi | Actinopterygii | Actinopteri | Neopterygii | Teleostei | Elopocephala | Clupeocephala | Otocephala | Ostariophysi | Otophysi | Siluriphysi | Siluriformes | Ictaluridae | Ictalurus
Reference/s:
GenBank - NIH genetic sequence database: GenBank species TBLASTN
International Species Sequencing Consortium: Channel catfish Sequencing Consortium
The Gene Index Project: DFCI Ictalurus punctatus (Catfish) Gene Index
Publication/s:
Ju Z *et. al.* , *Gene* , **261** , 373 (2000).

***Latimeria chalumnae* (Lac)**
Taxonomy:
cellular organisms | Eukaryota | Opisthokonta | Metazoa | Eumetazoa | Bilateria | Coelomata | Deuterostomia | Chordata | Craniata | Vertebrata | Gnathostomata | Teleostomi | Euteleostomi | Sarcopterygii | Coelacanthimorpha | Coelacanthiformes | Coelacanthidae | Latimeria
Reference/s:
Broad Institute of Harvard and MIT: Coelacanth Genome Project
GenBank - NIH genetic sequence database: GenBank species TBLASTN WGS

***Ornithorhynchus anatinus* (Ora)**
Taxonomy:
cellular organisms | Eukaryota | Opisthokonta | Metazoa | Eumetazoa | Bilateria | Coelomata | Deuterostomia | Chordata | Craniata | Vertebrata | Gnathostomata | Teleostomi | Euteleostomi | Sarcopterygii | Tetrapoda | Amniota | Mammalia | Prototheria | Monotremata | Ornithorhynchidae | Ornithorhynchus
Reference/s:
International Species Sequencing Consortium: Ornithorhynchus anatinus Sequencing Consortium
National Center for Biotechnology Information: NCBI Eukaryotic Genomes Project
The Genome Sequencing Center at Washington University: Ornithorhynchus anatinus
UCSC Genome Bioinformatics: Platypus (Ornithorhynchus anatinus) Genome Browser Gateway
e! Ensembl: Platypus
Publication/s:
Warren WC *et. al.* , *Nature* , **453** , 175 (2008).

***Loxodonta africana* (La)**
Taxonomy:
cellular organisms | Eukaryota | Opisthokonta | Metazoa | Eumetazoa | Bilateria | Coelomata | Deuterostomia | Chordata | Craniata | Vertebrata | Gnathostomata | Teleostomi | Euteleostomi | Sarcopterygii | Tetrapoda | Amniota | Mammalia | Theria | Eutheria | Afrotheria | Proboscidea | Elephantidae | Loxodonta
Reference/s:
Broad Institute of Harvard and MIT: Elephant
National Center for Biotechnology Information: NCBI Eukaryotic Genomes Project
UCSC Genome Bioinformatics: Elephant (Loxodonta africana) Genome Browser Gateway
e! Ensembl: Elephant

***Echinops telfairi* (Ect)**
Taxonomy:
cellular organisms | Eukaryota | Opisthokonta | Metazoa | Eumetazoa | Bilateria | Coelomata | Deuterostomia | Chordata | Craniata | Vertebrata | Gnathostomata | Teleostomi | Euteleostomi | Sarcopterygii | Tetrapoda | Amniota | Mammalia | Theria | Eutheria | Afrotheria | Tenrecidae | Tenrecinae | Echinops
Reference/s:
Broad Institute of Harvard and MIT: Mammalian Genome Project
National Center for Biotechnology Information: NCBI Eukaryotic Genomes Project
e! Ensembl: Lesser Hedgehog Tenrec

***Oryctolagus cuniculus* (Oc)**
Taxonomy:
cellular organisms | Eukaryota | Opisthokonta | Metazoa | Eumetazoa | Bilateria | Coelomata | Deuterostomia | Chordata | Craniata | Vertebrata | Gnathostomata | Teleostomi | Euteleostomi | Sarcopterygii | Tetrapoda | Amniota | Mammalia | Theria | Eutheria | Euarchontoglires | Glires | Lagomorpha | Leporidae | Oryctolagus
Reference/s:
Broad Institute of Harvard and MIT: Rabbit Genome Sequencing Project
National Center for Biotechnology Information: NCBI Eukaryotic Genomes Project
National Center for Biotechnology Information Reference Sequences: Oryctolagus cuniculus (European rabbit) genome view
UCSC Genome Bioinformatics: Rabbit (Oryctolagus cuniculus) Genome Browser Gateway
e! Ensembl: Rabbit

***Cavia porcellus str. inbred* (Cvp)**
Taxonomy:
cellular organisms | Eukaryota | Opisthokonta | Metazoa | Eumetazoa | Bilateria | Coelomata | Deuterostomia | Chordata | Craniata | Vertebrata | Gnathostomata | Teleostomi | Euteleostomi | Sarcopterygii | Tetrapoda | Amniota | Mammalia | Theria | Eutheria | Euarchontoglires | Glires | Rodentia | Hystricognathi | Caviidae | Cavia
Reference/s:
Broad Institute of Harvard and MIT: Guinea pig
National Center for Biotechnology Information: NCBI Eukaryotic Genomes Project
UCSC Genome Bioinformatics: Guinea pig (Cavia porcellus) Genome Browser Gateway
e! Ensembl: Guinea Pig

***Cavia porcellus* (Cav)**
Taxonomy:
cellular organisms | Eukaryota | Opisthokonta | Metazoa | Eumetazoa | Bilateria | Coelomata | Deuterostomia | Chordata | Craniata | Vertebrata | Gnathostomata | Teleostomi | Euteleostomi | Sarcopterygii | Tetrapoda | Amniota | Mammalia | Theria | Eutheria | Euarchontoglires | Glires | Rodentia | Hystricognathi | Caviidae | Cavia

***Mus musculus str. mixed* (Mm_a)**
Taxonomy:
cellular organisms | Eukaryota | Opisthokonta | Metazoa | Eumetazoa | Bilateria | Coelomata | Deuterostomia | Chordata | Craniata | Vertebrata | Gnathostomata | Teleostomi | Euteleostomi | Sarcopterygii | Tetrapoda | Amniota | Mammalia | Theria | Eutheria | Euarchontoglires | Glires | Rodentia | Sciurognathi | Muroidea | Muridae | Murinae | Mus | Mus
Reference/s:
National Center for Biotechnology Information: NCBI Eukaryotic Genomes Project
National Center for Biotechnology Information Reference Sequences: Mouse Genome Resources

***Mus musculus C57BL/6J* (Mm)**
Taxonomy:
cellular organisms | Eukaryota | Opisthokonta | Metazoa | Eumetazoa | Bilateria | Coelomata | Deuterostomia | Chordata | Craniata | Vertebrata | Gnathostomata | Teleostomi | Euteleostomi | Sarcopterygii | Tetrapoda | Amniota | Mammalia | Theria | Eutheria | Euarchontoglires | Glires | Rodentia | Sciurognathi | Muroidea | Muridae | Murinae | Mus | Mus
Reference/s:
Broad Institute of Harvard and MIT: Mouse Genome Data
GenBank - NIH genetic sequence database: GenBank species TBLASTN
Human Genome Sequencing Center at Baylor College of Medicine: Mouse Genome Project
International Species Sequencing Consortium: Mus musculus Sequencing Consortium
NCBI Mammalian Gene Collection: Mus musculus
National Center for Biotechnology Information: Mouse Sequencing Project
National Center for Biotechnology Information Reference Sequences: Mouse Genome Resources
The Gene Index Project: DFCI Mouse (Mus musculus) Gene Index
The Genome Sequencing Center at Washington University: Mus musculus
The Jackson Laboratory: Mouse Genome Informatics
UCSC Genome Bioinformatics: Mus musculus Genome Browser Gateway
e! Ensembl: Mouse
Publication/s:
Gnerre S *et. al.* , *Proc Natl Acad Sci U S A* , **108** , 1513 (2010).
Carninci P *et. al.* , *Science* , **309** , 1559 (2005).
Gerhard DS *et. al.* , *Genome Res* , **14** , 2121 (2004).
Strausberg RL *et. al.* , *Proc Natl Acad Sci U S A* , **99** , 16899 (2002).
Okazaki Y *et. al.* , *Nature* , **420** , 563 (2002).
Waterston RH *et. al.* , *Nature* , **420** , 520 (2002).

Kawai J *et. al.* , *Nature* , **409** , 685 (2001).

***Rattus norvegicus BN/Sprague-Dawley* (Rn_a)**
Taxonomy:
cellular organisms | Eukaryota | Opisthokonta | Metazoa | Eumetazoa | Bilateria | Coelomata | Deuterostomia | Chordata | Craniata | Vertebrata | Gnathostomata | Teleostomi | Euteleostomi | Sarcopterygii | Tetrapoda | Amniota | Mammalia | Theria | Eutheria | Euarchontoglires | Glires | Rodentia | Sciurognathi | Muroidea | Muridae | Murinae | Rattus
Reference/s:
National Center for Biotechnology Information: NCBI Eukaryotic Genomes Project
National Center for Biotechnology Information Reference Sequences: Rattus norvegicus (rat) genome view

***Rattus norvegicus BN/SsNHsdMCW* (Rn)**
Taxonomy:
cellular organisms | Eukaryota | Opisthokonta | Metazoa | Eumetazoa | Bilateria | Coelomata | Deuterostomia | Chordata | Craniata | Vertebrata | Gnathostomata | Teleostomi | Euteleostomi | Sarcopterygii | Tetrapoda | Amniota | Mammalia | Theria | Eutheria | Euarchontoglires | Glires | Rodentia | Sciurognathi | Muroidea | Muridae | Murinae | Rattus
Reference/s:
Bioinformatics Program, HMGC: Rat Genome Database
Human Genome Sequencing Center at Baylor College of Medicine: Rat Genome Project
International Species Sequencing Consortium: Rattus norvegicus Sequencing Consortium
NCBI Mammalian Gene Collection: Rattus norvegicus
National Center for Biotechnology Information: Rat Sequencing Project
National Center for Biotechnology Information Reference Sequences: Rattus norvegicus (rat) genome view
The Gene Index Project: DFCI Rat (Rattus norvegicus) Gene Index
UCSC Genome Bioinformatics: Rattus norvegicus Genome Browser Gateway
e! Ensembl: Rat
Publication/s:
Gerhard DS *et. al.* , *Genome Res* , **14** , 2121 (2004).
Gibbs RA *et. al.* , *Nature* , **428** , 493 (2004).
Strausberg RL *et. al.* , *Proc Natl Acad Sci U S A* , **99** , 16899 (2002).

***Spermophilus tridecemlineatus* (Spt)**
Taxonomy:
cellular organisms | Eukaryota | Opisthokonta | Metazoa | Eumetazoa | Bilateria | Coelomata | Deuterostomia | Chordata | Craniata | Vertebrata | Gnathostomata | Teleostomi | Euteleostomi | Sarcopterygii | Tetrapoda | Amniota | Mammalia | Theria | Eutheria | Euarchontoglires | Glires | Rodentia | Sciurognathi | Sciuridae | Xerinae | Marmotini | Ictidomys
Reference/s:
Broad Institute of Harvard and MIT: Mammalian Genome Project
National Center for Biotechnology Information: NCBI Eukaryotic Genomes Project
e! Ensembl: Squirrel

***Macaca nemestrina* (Mn)**
Taxonomy:
cellular organisms | Eukaryota | Opisthokonta | Metazoa | Eumetazoa | Bilateria | Coelomata | Deuterostomia | Chordata | Craniata | Vertebrata | Gnathostomata | Teleostomi | Euteleostomi | Sarcopterygii | Tetrapoda | Amniota | Mammalia | Theria | Eutheria | Euarchontoglires | Primates | Haplorrhini | Simiiformes | Catarrhini | Cercopithecoidea | Cercopithecidae | Cercopithecinae | Macaca

***Macaca mulatta Indian origin* (Mam)**
Taxonomy:
cellular organisms | Eukaryota | Opisthokonta | Metazoa | Eumetazoa | Bilateria | Coelomata | Deuterostomia | Chordata | Craniata | Vertebrata | Gnathostomata | Teleostomi | Euteleostomi | Sarcopterygii | Tetrapoda | Amniota | Mammalia | Theria | Eutheria | Euarchontoglires | Primates | Haplorrhini | Simiiformes | Catarrhini | Cercopithecoidea | Cercopithecidae | Cercopithecinae | Macaca
Reference/s:
GenBank - NIH genetic sequence database: GenBank species TBLASTN
Human Genome Sequencing Center at Baylor College of Medicine: Rhesus Monkey Genome Project
International Species Sequencing Consortium: Macaca mulatta Sequencing Consortium
National Center for Biotechnology Information: NCBI Eukaryotic Genomes Project
National Center for Biotechnology Information Reference Sequences: Rhesus Macaque Genome Resources
The Genome Sequencing Center at Washington University: Macaca mulatta
UCSC Genome Bioinformatics: Macaca mulatta Genome Browser Gateway
e! Ensembl: Rhesus macaque
Publication/s:
Gibbs RA *et. al.* , *Science* , **316** , 222 (2007).
Magness CL *et. al.* , *Genome Biol* , **6** , R60 (2005).

***Macaca fascicularis* (Mf)**
Taxonomy:
cellular organisms | Eukaryota | Opisthokonta | Metazoa | Eumetazoa | Bilateria | Coelomata | Deuterostomia | Chordata | Craniata | Vertebrata | Gnathostomata | Teleostomi | Euteleostomi | Sarcopterygii | Tetrapoda | Amniota | Mammalia | Theria | Eutheria | Euarchontoglires | Primates | Haplorrhini | Simiiformes | Catarrhini | Cercopithecoidea | Cercopithecidae | Cercopithecinae | Macaca
Reference/s:
GenBank - NIH genetic sequence database: GenBank species TBLASTN
National Center for Biotechnology Information: NCBI Eukaryotic Genomes Project
Publication/s:
Wang HY *et. al.* , *PLoS Biol* , **5** , e13 (2007).
Osada N *et. al.* , *Mol Biol Evol* , **22** , 1976 (2005).
Magness CL *et. al.* , *Genome Biol* , **6** , R60 (2005).

***Papio anubis* (Pan)**
Taxonomy:

cellular organisms | Eukaryota | Opisthokonta | Metazoa | Eumetazoa | Bilateria | Coelomata | Deuterostomia | Chordata | Craniata | Vertebrata | Gnathostomata | Teleostomi | Euteleostomi | Sarcopterygii | Tetrapoda | Amniota | Mammalia | Theria | Eutheria | Euarchontoglires | Primates | Haplorrhini | Simiiformes | Catarrhini | Cercopithecoidea | Cercopithecidae | Cercopithecinae | Papio

***Gorilla gorilla gorilla* (Ggg)**
Taxonomy:
cellular organisms | Eukaryota | Opisthokonta | Metazoa | Eumetazoa | Bilateria | Coelomata | Deuterostomia | Chordata | Craniata | Vertebrata | Gnathostomata | Teleostomi | Euteleostomi | Sarcopterygii | Tetrapoda | Amniota | Mammalia | Theria | Eutheria | Euarchontoglires | Primates | Haplorrhini | Simiiformes | Catarrhini | Hominoidea | Hominidae | Homininae | Gorilla | Gorilla gorilla
Reference/s:
International Species Sequencing Consortium: Gorilla Sequencing Consortium
National Center for Biotechnology Information: NCBI Eukaryotic Genomes Project
e! Ensembl: Gorilla (Gorilla gorilla)
Publication/s:
Scally A *et. al.* , *Nature* , **483** , 169 (2012).

***Homo sapiens JCVenter* (Hs_a)**
Taxonomy:
cellular organisms | Eukaryota | Opisthokonta | Metazoa | Eumetazoa | Bilateria | Coelomata | Deuterostomia | Chordata | Craniata | Vertebrata | Gnathostomata | Teleostomi | Euteleostomi | Sarcopterygii | Tetrapoda | Amniota | Mammalia | Theria | Eutheria | Euarchontoglires | Primates | Haplorrhini | Simiiformes | Catarrhini | Hominoidea | Hominidae | Homininae | Homo
Reference/s:
International Species Sequencing Consortium: JCVenter Genome Sequencing
National Center for Biotechnology Information: NCBI Eukaryotic Genomes Project
Publication/s:
Levy S *et. al.* , *PLoS Biol* , **5** , e254 (2007).

***Homo sapiens* (Hs)**
Taxonomy:
cellular organisms | Eukaryota | Opisthokonta | Metazoa | Eumetazoa | Bilateria | Coelomata | Deuterostomia | Chordata | Craniata | Vertebrata | Gnathostomata | Teleostomi | Euteleostomi | Sarcopterygii | Tetrapoda | Amniota | Mammalia | Theria | Eutheria | Euarchontoglires | Primates | Haplorrhini | Simiiformes | Catarrhini | Hominoidea | Hominidae | Homininae | Homo
Reference/s:
GenBank - NIH genetic sequence database: GenBank species TBLASTN
Human Genome Sequencing Center at Baylor College of Medicine: HapMap 3 and ENCODE 3
International Species Sequencing Consortium: Homo sapiens Sequencing Consortium
NCBI Mammalian Gene Collection: Homo sapiens
National Center for Biotechnology Information: Human Sequencing Project
National Center for Biotechnology Information Reference Sequences: Human Genome Resource
The Gene Index Project: DFCI Human (Homo sapiens) Gene Index
The Genome Sequencing Center at Washington University: Homo sapiens
UCSC Genome Bioinformatics: Homo sapiens Genome Browser Gateway
e! Ensembl: Human
Publication/s:
Gnerre S *et. al.* , *Proc Natl Acad Sci U S A* , **108** , 1513 (2010).
Zody MC *et. al.* , *Nature* , **440** , 671 (2006).
Zody MC *et. al.* , *Nature* , **440** , 1045 (2006).
Scherer SE *et. al.* , *Nature* , **440** , 346 (2006).
Muzny DM *et. al.* , *Nature* , **440** , 1194 (2006).
Kimura K *et. al.* , *Genome Res* , **16** , 55 (2006).
Gregory SG *et. al.* , *Nature* , **441** , 315 (2006).
Taylor TD *et. al.* , *Nature* , **440** , 497 (2006).
Nusbaum C *et. al.* , *Nature* , **439** , 331 (2006).
Nusbaum C *et. al.* , *Nature* , **437** , 551 (2005).
Hillier LW *et. al.* , *Nature* , **434** , 724 (2005).
Ross MT *et. al.* , *Nature* , **434** , 325 (2005).
Gerhard DS *et. al.* , *Genome Res* , **14** , 2121 (2004).
Rual JF *et. al.* , *Genome Res* , **14** , 2128 (2004).
Brandenberger R *et. al.* , *Nat Biotechnol* , **22** , 707 (2004).
Grimwood J *et. al.* , *Nature* , **428** , 529 (2004).
Collins JE *et. al.* , *Genome Biol* , **5** , R84 (2004).
Ota T *et. al.* , *Nat Genet* , **36** , 40 (2004).
Dunham A *et. al.* , *Nature* , **428** , 522 (2004).
Deloukas P *et. al.* , *Nature* , **429** , 375 (2004).
Humphray SJ *et. al.* , *Nature* , **429** , 369 (2004).
Imanishi T *et. al.* , *PLoS Biol* , **2** , e162 (2004).
Schmutz J *et. al.* , *Nature* , **431** , 268 (2004).
Martin J *et. al.* , *Nature* , **432** , 988 (2004).
Mungall AJ *et. al.* , *Nature* , **425** , 805 (2003).
Hillier LW *et. al.* , *Nature* , **424** , 157 (2003).
Heilig R *et. al.* , *Nature* , **421** , 601 (2003).
Strausberg RL *et. al.* , *Proc Natl Acad Sci U S A* , **99** , 16899 (2002).
Deloukas P *et. al.* , *Nature* , **414** , 865 (2001).
Venter JC *et. al.* , *Science* , **291** , 1304 (2001).
Lander ES *et. al.* , *Nature* , **409** , 860 (2001).
Dias Neto E *et. al.* , *Proc Natl Acad Sci U S A* , **97** , 3491 (2000).
Hattori M *et. al.* , *Nature* , **405** , 311 (2000).
Dunham I *et. al.* , *Nature* , **402** , 489 (1999).

***Pan troglodytes* (Pat)**
Taxonomy:
cellular organisms | Eukaryota | Opisthokonta | Metazoa | Eumetazoa | Bilateria | Coelomata | Deuterostomia | Chordata | Craniata | Vertebrata | Gnathostomata | Teleostomi | Euteleostomi | Sarcopterygii | Tetrapoda | Amniota | Mammalia | Theria | Eutheria | Euarchontoglires | Primates | Haplorrhini | Simiiformes | Catarrhini | Hominoidea | Hominidae | Homininae | Pan
Reference/s:
GenBank - NIH genetic sequence database: GenBank species TBLASTN
Human Genome Sequencing Center at Baylor College of Medicine: Chimpanzee Genome Analysis
International Species Sequencing Consortium: Pan troglodytes Sequencing Consortium
National Center for Biotechnology Information: Chimpanzee Sequencing Project
National Center for Biotechnology Information Reference Sequences: Pan troglodytes (chimpanzee) genome view
The Genome Sequencing Center at Washington University: Pan troglodytes
UCSC Genome Bioinformatics: Pan troglodytes Genome Browser Gateway
e! Ensembl: Chimp
Publication/s:
Hughes JF *et. al.* , *Nature* , **437** , 100 (2005).
Chimpanzee Sequencing and Analysis Consortium , *Nature* , **437** , 69 (2005).
Watanabe H *et. al.* , *Nature* , **429** , 382 (2004).
Sakate R *et. al.* , *Genome Res* , **13** , 1022 (2003).
Hellmann I *et. al.* , *Genome Res* , **13** , 831 (2003).

***Pongo abelii* (Pna)**
Taxonomy:
cellular organisms | Eukaryota | Opisthokonta | Metazoa | Eumetazoa | Bilateria | Coelomata | Deuterostomia | Chordata | Craniata | Vertebrata | Gnathostomata | Teleostomi | Euteleostomi | Sarcopterygii | Tetrapoda | Amniota | Mammalia | Theria | Eutheria | Euarchontoglires | Primates | Haplorrhini | Simiiformes | Catarrhini | Hominoidea | Hominidae | Ponginae | Pongo
Reference/s:
Human Genome Sequencing Center at Baylor College of Medicine: Orangutan Genome Project
International Species Sequencing Consortium: Orangutan Sequencing Consortium
National Center for Biotechnology Information: NCBI Eukaryotic Genomes Project
National Center for Biotechnology Information Reference Sequences: Pongo abelii (Sumatran orangutan) genome view
The Genome Sequencing Center at Washington University: Sumatran Orangutan
UCSC Genome Bioinformatics: Orangutan (Pongo pygmaeus abelii) Genome Browser Gateway
e! Ensembl: Orangutan (Pongo pygmaeus)
Publication/s:
Locke DP *et. al.* , *Nature* , **469** , 529 (2011).

***Callithrix jacchus* (Caj)**
Taxonomy:
cellular organisms | Eukaryota | Opisthokonta | Metazoa | Eumetazoa | Bilateria | Coelomata | Deuterostomia | Chordata | Craniata | Vertebrata | Gnathostomata | Teleostomi | Euteleostomi | Sarcopterygii | Tetrapoda | Amniota | Mammalia | Theria | Eutheria | Euarchontoglires | Primates | Haplorrhini | Simiiformes | Platyrrhini | Cebidae | Callitrichinae | Callithrix
Reference/s:
Human Genome Sequencing Center at Baylor College of Medicine: Marmoset Genome Project
National Center for Biotechnology Information: NCBI Eukaryotic Genomes Project
National Center for Biotechnology Information Reference Sequences: Callithrix jacchus (white-tufted-ear marmoset) genome view
The Genome Sequencing Center at Washington University: Callithrix jacchus
UCSC Genome Bioinformatics: Marmoset (Callithrix jacchus) Genome Browser Gateway
e! Ensembl: Marmoset (Callithrix jacchus)

***Microcebus murinus* (Mim)**
Taxonomy:
cellular organisms | Eukaryota | Opisthokonta | Metazoa | Eumetazoa | Bilateria | Coelomata | Deuterostomia | Chordata | Craniata | Vertebrata | Gnathostomata | Teleostomi | Euteleostomi | Sarcopterygii | Tetrapoda | Amniota | Mammalia | Theria | Eutheria | Euarchontoglires | Primates | Strepsirrhini | Lemuriformes | Cheirogaleidae | Microcebus
Reference/s:
Broad Institute of Harvard and MIT: Mammalian Genome Project
National Center for Biotechnology Information: NCBI Eukaryotic Genomes Project
e! Ensembl: Mouse Lemur (Microcebus murinus)

***Otolemur garnettii* (Otg)**
Taxonomy:
cellular organisms | Eukaryota | Opisthokonta | Metazoa | Eumetazoa | Bilateria | Coelomata | Deuterostomia | Chordata | Craniata | Vertebrata | Gnathostomata | Teleostomi | Euteleostomi | Sarcopterygii | Tetrapoda | Amniota | Mammalia | Theria | Eutheria | Euarchontoglires | Primates | Strepsirrhini | Lorisiformes | Galagidae | Otolemur
Reference/s:
Broad Institute of Harvard and MIT: Mammalian Genome Project
National Center for Biotechnology Information: NCBI Eukaryotic Genomes Project
e! Ensembl: Bushbaby

***Tupaia belangeri* (Tub)**
Taxonomy:
cellular organisms | Eukaryota | Opisthokonta | Metazoa | Eumetazoa | Bilateria | Coelomata | Deuterostomia | Chordata | Craniata | Vertebrata | Gnathostomata | Teleostomi | Euteleostomi | Sarcopterygii | Tetrapoda | Amniota | Mammalia | Theria | Eutheria | Euarchontoglires | Scandentia | Tupaiidae | Tupaia
Reference/s:
Broad Institute of Harvard and MIT: Mammalian Genome Project
National Center for Biotechnology Information: NCBI Eukaryotic Genomes Project
e! Ensembl: Treeshrew

***Canis lupus familiaris breed poodle* (Caf_a)**
Taxonomy:

cellular organisms | Eukaryota | Opisthokonta | Metazoa | Eumetazoa | Bilateria | Coelomata | Deuterostomia | Chordata | Craniata | Vertebrata | Gnathostomata | Teleostomi | Euteleostomi | Sarcopterygii | Tetrapoda | Amniota | Mammalia | Theria | Eutheria | Laurasiatheria | Carnivora | Caniformia | Canidae | Canis | Canis lupus
Reference/s:
National Center for Biotechnology Information: NCBI Eukaryotic Genomes Project
Publication/s:
Kirkness EF *et. al.* , *Science* , **301** , 1898 (2003).

Canis lupus familiaris breed boxer **(Caf)**
Taxonomy:
cellular organisms | Eukaryota | Opisthokonta | Metazoa | Eumetazoa | Bilateria | Coelomata | Deuterostomia | Chordata | Craniata | Vertebrata | Gnathostomata | Teleostomi | Euteleostomi | Sarcopterygii | Tetrapoda | Amniota | Mammalia | Theria | Eutheria | Laurasiatheria | Carnivora | Caniformia | Canidae | Canis | Canis lupus
Reference/s:
Broad Institute of Harvard and MIT: Dog Genome Sequencing Project
International Species Sequencing Consortium: Canis familiaris Sequencing Consortium
National Center for Biotechnology Information: Dog Sequencing Project
National Center for Biotechnology Information Reference Sequences: Dog Genome Resources
The Gene Index Project: DFCI Canis familiaris Gene Index
UCSC Genome Bioinformatics: Canis familiaris Genome Browser Gateway
e! Ensembl: Dog
Publication/s:
Lindblad-Toh K *et. al.* , *Nature* , **438** , 803 (2005).

Canis lupus familiaris breed beagle **(Caf_b)**
Taxonomy:
cellular organisms | Eukaryota | Opisthokonta | Metazoa | Eumetazoa | Bilateria | Coelomata | Deuterostomia | Chordata | Craniata | Vertebrata | Gnathostomata | Teleostomi | Euteleostomi | Sarcopterygii | Tetrapoda | Amniota | Mammalia | Theria | Eutheria | Laurasiatheria | Carnivora | Caniformia | Canidae | Canis | Canis lupus

Ailuropoda melanoleuca **(Aim)**
Taxonomy:
cellular organisms | Eukaryota | Opisthokonta | Metazoa | Eumetazoa | Bilateria | Coelomata | Deuterostomia | Chordata | Craniata | Vertebrata | Gnathostomata | Teleostomi | Euteleostomi | Sarcopterygii | Tetrapoda | Amniota | Mammalia | Theria | Eutheria | Laurasiatheria | Carnivora | Caniformia | Ursidae | Ailuropoda
Reference/s:
International Species Sequencing Consortium: Giant Panda Sequencing Consortium
National Center for Biotechnology Information: NCBI Eukaryotic Genomes Project
National Center for Biotechnology Information Reference Sequences: Ailuropoda melanoleuca (giant panda) genome view
UCSC Genome Bioinformatics: Panda (Ailuropoda melanoleuca) Genome Browser Gateway
e! Ensembl: Giant Panda (Ailuropoda melanoleuca)
Publication/s:
Li R *et. al.* , *Nature* , **463** , 311 (2010).

Felis catus **(Fc)**
Taxonomy:
cellular organisms | Eukaryota | Opisthokonta | Metazoa | Eumetazoa | Bilateria | Coelomata | Deuterostomia | Chordata | Craniata | Vertebrata | Gnathostomata | Teleostomi | Euteleostomi | Sarcopterygii | Tetrapoda | Amniota | Mammalia | Theria | Eutheria | Laurasiatheria | Carnivora | Feliformia | Felidae | Felinae | Felis
Reference/s:
Broad Institute of Harvard and MIT: Mammalian Genome Project
International Species Sequencing Consortium: Felis catus Sequencing Consortium
National Center for Biotechnology Information: Cat Sequencing Project
UCSC Genome Bioinformatics: Cat (Felis catus) Genome Browser Gateway
e! Ensembl: Cat
Publication/s:
Pontius JU *et. al.* , *Genome Res* , **17** , 1675 (2007).

Bos taurus **(Bt)**
Taxonomy:
cellular organisms | Eukaryota | Opisthokonta | Metazoa | Eumetazoa | Bilateria | Coelomata | Deuterostomia | Chordata | Craniata | Vertebrata | Gnathostomata | Teleostomi | Euteleostomi | Sarcopterygii | Tetrapoda | Amniota | Mammalia | Theria | Eutheria | Laurasiatheria | Cetartiodactyla | Ruminantia | Pecora | Bovidae | Bovinae | Bos
Reference/s:
GenBank - NIH genetic sequence database: GenBank species TBLASTN
Human Genome Sequencing Center at Baylor College of Medicine: Bovine Genome Project
International Species Sequencing Consortium: Bos taurus Sequencing Consortium
NCBI Mammalian Gene Collection: Bos taurus
National Center for Biotechnology Information: Cow Sequencing Project
National Center for Biotechnology Information Reference Sequences: Bos taurus (cattle) genome view
The Gene Index Project: DFCI Cattle (Bos taurus) Gene Index
UCSC Genome Bioinformatics: Bos taurus Genome Browser Gateway
e! Ensembl: Cow
Publication/s:
Zimin AV *et. al.* , *Genome Biol* , **10** , R42 (2009).
Elsik CG *et. al.* , *Science* , **324** , 522 (2009).
Sonstegard TS *et. al.* , *Mamm Genome* , **13** , 373 (2002).
Takasuga A *et. al.* , *Nucleic Acids Res* , **29** , E108 (2001).
Smith TP *et. al.* , *Genome Res* , **11** , 626 (2001).

Capra hircus **(Cah)**
Taxonomy:
cellular organisms | Eukaryota | Opisthokonta | Metazoa | Eumetazoa | Bilateria | Coelomata | Deuterostomia | Chordata | Craniata | Vertebrata | Gnathostomata | Teleostomi | Euteleostomi | Sarcopterygii | Tetrapoda | Amniota | Mammalia | Theria | Eutheria | Laurasiatheria | Cetartiodactyla | Ruminantia | Pecora | Bovidae | Caprinae | Capra

Ovis aries **(Oa)**

Taxonomy:
cellular organisms | Eukaryota | Opisthokonta | Metazoa | Eumetazoa | Bilateria | Coelomata | Deuterostomia | Chordata | Craniata | Vertebrata | Gnathostomata | Teleostomi | Euteleostomi | Sarcopterygii | Tetrapoda | Amniota | Mammalia | Theria | Eutheria | Laurasiatheria | Cetartiodactyla | Ruminantia | Pecora | Bovidae | Caprinae | Ovis
Reference/s:
GenBank - NIH genetic sequence database: GenBank species TBLASTN
National Center for Biotechnology Information: NCBI Eukaryotic Genomes Project
Publication/s:
Hecht J *et. al.* , *BMC Genomics* , **7** , 172 (2006).
Adelson DL *et. al.* , *Genomics* , **83** , 95 (2004).

***Sus scrofa domestica* (Ss)**
Taxonomy:
cellular organisms | Eukaryota | Opisthokonta | Metazoa | Eumetazoa | Bilateria | Coelomata | Deuterostomia | Chordata | Craniata | Vertebrata | Gnathostomata | Teleostomi | Euteleostomi | Sarcopterygii | Tetrapoda | Amniota | Mammalia | Theria | Eutheria | Laurasiatheria | Cetartiodactyla | Suina | Suidae | Sus | Sus scrofa
Reference/s:
GenBank - NIH genetic sequence database: GenBank species TBLASTN
National Center for Biotechnology Information: Pig Sequencing Project
PEDE: Pig Expression Data Explorer
The Gene Index Project: DFCI Porcine (Sus scrofa) Gene Index
The Wellcome Trust Sanger Institute: Porcine Genome Sequencing Project
UCSC Genome Bioinformatics: Pig (Sus scrofa) Genome Browser Gateway
e! Ensembl: Pig (Sus scrofa)
Publication/s:
Uenishi H *et. al.* , *Nucleic Acids Res* , **32** , D484 (2004).
Fahrenkrug SC *et. al.* , *Mamm Genome* , **13** , 475 (2002).
Davoli R *et. al.* , *Anim Genet* , **33** , 3 (2002).

***Myotis lucifugus* (Myl)**
Taxonomy:
cellular organisms | Eukaryota | Opisthokonta | Metazoa | Eumetazoa | Bilateria | Coelomata | Deuterostomia | Chordata | Craniata | Vertebrata | Gnathostomata | Teleostomi | Euteleostomi | Sarcopterygii | Tetrapoda | Amniota | Mammalia | Theria | Eutheria | Laurasiatheria | Chiroptera | Microchiroptera | Vespertilionidae | Myotis
Reference/s:
Broad Institute of Harvard and MIT: Little brown bat
National Center for Biotechnology Information: NCBI Eukaryotic Genomes Project
e! Ensembl: Microbat

***Erinaceus europaeus* (Ere)**
Taxonomy:
cellular organisms | Eukaryota | Opisthokonta | Metazoa | Eumetazoa | Bilateria | Coelomata | Deuterostomia | Chordata | Craniata | Vertebrata | Gnathostomata | Teleostomi | Euteleostomi | Sarcopterygii | Tetrapoda | Amniota | Mammalia | Theria | Eutheria | Laurasiatheria | Insectivora | Erinaceidae | Erinaceinae | Erinaceus
Reference/s:
Broad Institute of Harvard and MIT: Mammalian Genome Project
National Center for Biotechnology Information: NCBI Eukaryotic Genomes Project
e! Ensembl: Hedgehog

***Sorex araneus* (Soa)**
Taxonomy:
cellular organisms | Eukaryota | Opisthokonta | Metazoa | Eumetazoa | Bilateria | Coelomata | Deuterostomia | Chordata | Craniata | Vertebrata | Gnathostomata | Teleostomi | Euteleostomi | Sarcopterygii | Tetrapoda | Amniota | Mammalia | Theria | Eutheria | Laurasiatheria | Insectivora | Soricidae | Soricinae | Sorex
Reference/s:
Broad Institute of Harvard and MIT: Mammalian Genome Project
National Center for Biotechnology Information: NCBI Eukaryotic Genomes Project
e! Ensembl: Common Shrew

***Equus caballus* (Eqc)**
Taxonomy:
cellular organisms | Eukaryota | Opisthokonta | Metazoa | Eumetazoa | Bilateria | Coelomata | Deuterostomia | Chordata | Craniata | Vertebrata | Gnathostomata | Teleostomi | Euteleostomi | Sarcopterygii | Tetrapoda | Amniota | Mammalia | Theria | Eutheria | Laurasiatheria | Perissodactyla | Equidae | Equus | Equus subg. Equus
Reference/s:
Broad Institute of Harvard and MIT: Horse Genome Project
International Species Sequencing Consortium: Domestic horse Sequencing Consortium
National Center for Biotechnology Information: NCBI Eukaryotic Genomes Project
UCSC Genome Bioinformatics: Horse (Equus caballus) Genome Browser Gateway
e! Ensembl: Horse (Equus caballus)
Publication/s:
Wade CM *et. al.* , *Science* , **326** , 865 (2009).

***Dasypus novemcinctus* (Dn)**
Taxonomy:
cellular organisms | Eukaryota | Opisthokonta | Metazoa | Eumetazoa | Bilateria | Coelomata | Deuterostomia | Chordata | Craniata | Vertebrata | Gnathostomata | Teleostomi | Euteleostomi | Sarcopterygii | Tetrapoda | Amniota | Mammalia | Theria | Eutheria | Xenarthra | Cingulata | Dasypodidae | Dasypus
Reference/s:
Broad Institute of Harvard and MIT: Mammalian Genome Project
National Center for Biotechnology Information: NCBI Eukaryotic Genomes Project
e! Ensembl: Armadillo

***Sminthopsis crassicaudata* (Smc)**
Taxonomy:
cellular organisms | Eukaryota | Opisthokonta | Metazoa | Eumetazoa | Bilateria | Coelomata | Deuterostomia | Chordata | Craniata | Vertebrata | Gnathostomata | Teleostomi | Euteleostomi | Sarcopterygii | Tetrapoda | Amniota | Mammalia | Theria | Metatheria | Dasyuromorphia | Dasyuridae | Sminthopsis

***Monodelphis domestica* (Md)**
Taxonomy:
cellular organisms | Eukaryota | Opisthokonta | Metazoa | Eumetazoa | Bilateria | Coelomata | Deuterostomia | Chordata | Craniata | Vertebrata | Gnathostomata | Teleostomi | Euteleostomi | Sarcopterygii | Tetrapoda | Amniota | Mammalia | Theria | Metatheria | Didelphimorphia | Didelphidae | Didelphinae | Monodelphis
Reference/s:
Broad Institute of Harvard and MIT: Opossum Genome
International Species Sequencing Consortium: Monodelphis domestica Sequencing Consortium
National Center for Biotechnology Information: NCBI Eukaryotic Genomes Project
National Center for Biotechnology Information Reference Sequences: Gray Short-tailed Opossum Genome Resources
UCSC Genome Bioinformatics: Monodelphis domestica Genome Browser Gateway
e! Ensembl: Opossum
Publication/s:
Mikkelsen TS *et. al.* , *Nature* , **447** , 167 (2007).

***Macropus eugenii* (Mae)**
Taxonomy:
cellular organisms | Eukaryota | Opisthokonta | Metazoa | Eumetazoa | Bilateria | Coelomata | Deuterostomia | Chordata | Craniata | Vertebrata | Gnathostomata | Teleostomi | Euteleostomi | Sarcopterygii | Tetrapoda | Amniota | Mammalia | Theria | Metatheria | Diprotodontia | Macropodidae | Macropus
Reference/s:
Human Genome Sequencing Center at Baylor College of Medicine: Wallaby Genome Project
International Species Sequencing Consortium: Wallaby Sequencing Consortium
National Center for Biotechnology Information: NCBI Eukaryotic Genomes Project
e! Ensembl: Wallaby (Macropus eugenii)
Publication/s:
Renfree MB *et. al.* , *Genome Biol* , **12** , R81 (2011).

***Trichosurus vulpecula* (Tcv)**
Taxonomy:
cellular organisms | Eukaryota | Opisthokonta | Metazoa | Eumetazoa | Bilateria | Coelomata | Deuterostomia | Chordata | Craniata | Vertebrata | Gnathostomata | Teleostomi | Euteleostomi | Sarcopterygii | Tetrapoda | Amniota | Mammalia | Theria | Metatheria | Diprotodontia | Phalangeridae | Trichosurus

***Meleagris gallopavo* (Meg)**
Taxonomy:
cellular organisms | Eukaryota | Opisthokonta | Metazoa | Eumetazoa | Bilateria | Coelomata | Deuterostomia | Chordata | Craniata | Vertebrata | Gnathostomata | Teleostomi | Euteleostomi | Sarcopterygii | Tetrapoda | Amniota | Sauropsida | Sauria | Archosauria | Dinosauria | Saurischia | Theropoda | Coelurosauria | Aves | Neognathae | Galliformes | Phasianidae | Meleagridinae | Meleagris
Reference/s:
International Species Sequencing Consortium: Meleagris Sequencing Consortium
National Center for Biotechnology Information: NCBI Eukaryotic Genomes Project
Publication/s:
Dalloul RA *et. al.* , *PLoS Biol* , **8** , e1000475 (2010).

***Gallus gallus* (Gg)**
Taxonomy:
cellular organisms | Eukaryota | Opisthokonta | Metazoa | Eumetazoa | Bilateria | Coelomata | Deuterostomia | Chordata | Craniata | Vertebrata | Gnathostomata | Teleostomi | Euteleostomi | Sarcopterygii | Tetrapoda | Amniota | Sauropsida | Sauria | Archosauria | Dinosauria | Saurischia | Theropoda | Coelurosauria | Aves | Neognathae | Galliformes | Phasianidae | Phasianinae | Gallus
Reference/s:
ChickEST Database: Gallus gallus EST database
GenBank - NIH genetic sequence database: GenBank species TBLASTN
International Species Sequencing Consortium: Gallus gallus Sequencing Consortium
National Center for Biotechnology Information: Chicken Sequencing Project
The Gene Index Project: DFCI Gallus gallus (Chicken) Gene Index
The Genome Sequencing Center at Washington University: Gallus gallus
UCSC Genome Bioinformatics: Gallus gallus Genome Browser Gateway
e! Ensembl: Chicken
Publication/s:
Shin JH *et. al.* , *Anim Genet* , **37** , 85 (2006).
Shin JH *et. al.* , *Anim Genet* , **36** , 346 (2005).
Savolainen P *et. al.* , *Cytogenet Genome Res* , **111** , 79 (2005).
Caldwell RB *et. al.* , *Genome Biol* , **6** , R6 (2005).
Hubbard SJ *et. al.* , *Genome Res* , **15** , 174 (2005).
Hillier LW *et. al.* , *Nature* , **432** , 695 (2004).
Boardman PE *et. al.* , *Curr Bio* , **12** , 1965 (2002).

***Taeniopygia guttata* (Tag)**
Taxonomy:
cellular organisms | Eukaryota | Opisthokonta | Metazoa | Eumetazoa | Bilateria | Coelomata | Deuterostomia | Chordata | Craniata | Vertebrata | Gnathostomata | Teleostomi | Euteleostomi | Sarcopterygii | Tetrapoda | Amniota | Sauropsida | Sauria | Archosauria | Dinosauria | Saurischia | Theropoda | Coelurosauria | Aves | Neognathae | Passeriformes | Passeroidea | Estrildidae | Estrildinae | Taeniopygia
Reference/s:
International Species Sequencing Consortium: Taeniopygia Sequencing Consortium
National Center for Biotechnology Information: NCBI Eukaryotic Genomes Project
National Center for Biotechnology Information Reference Sequences: Taeniopygia guttata (zebra finch) genome view
The Genome Sequencing Center at Washington University: Taeniopygia guttata
UCSC Genome Bioinformatics: Zebra finch (Taeniopygia guttata) Genome Browser Gateway
e! Ensembl: Zebra Finch (Taeniopygia guttata)
Publication/s:
Warren WC *et. al.* , *Nature* , **464** , 757 (2010).

***Anolis carolinensis* (Aoc)**
Taxonomy:
cellular organisms | Eukaryota | Opisthokonta | Metazoa | Eumetazoa | Bilateria | Coelomata | Deuterostomia | Chordata | Craniata | Vertebrata | Gnathostomata | Teleostomi | Euteleostomi | Sarcopterygii | Tetrapoda | Amniota | Sauropsida | Sauria | Lepidosauria | Squamata | Iguania | Iguanidae | Polychrotinae | Anolis
Reference/s:
Broad Institute of Harvard and MIT: Anolis Genome Sequencing Project
International Species Sequencing Consortium: Anolis carolinensis Sequencing Consortium
National Center for Biotechnology Information: NCBI Eukaryotic Genomes Project
UCSC Genome Bioinformatics: Lizard (Anolis carolinensis) Genome Browser Gateway
e! Ensembl: Anole Lizard (Anolis carolinensis)
Publication/s:
Alfoldi J *et. al.* , *Nature* , **477** , 587 (2011).

***Gekko japonicus* (Gj)**
Taxonomy:
cellular organisms | Eukaryota | Opisthokonta | Metazoa | Eumetazoa | Bilateria | Coelomata | Deuterostomia | Chordata | Craniata | Vertebrata | Gnathostomata | Teleostomi | Euteleostomi | Sarcopterygii | Tetrapoda | Amniota | Sauropsida | Sauria | Lepidosauria | Squamata | Scleroglossa | Gekkota | Gekkonidae | Gekkoninae | Gekko

***Elaphe quadrivirgata* (Eq)**
Taxonomy:
cellular organisms | Eukaryota | Opisthokonta | Metazoa | Eumetazoa | Bilateria | Coelomata | Deuterostomia | Chordata | Craniata | Vertebrata | Gnathostomata | Teleostomi | Euteleostomi | Sarcopterygii | Tetrapoda | Amniota | Sauropsida | Sauria | Lepidosauria | Squamata | Scleroglossa | Serpentes | Colubroidea | Colubridae | Colubrinae | Elaphe

***Xenopus tropicalis* (Xt)**
Taxonomy:
cellular organisms | Eukaryota | Opisthokonta | Metazoa | Eumetazoa | Bilateria | Coelomata | Deuterostomia | Chordata | Craniata | Vertebrata | Gnathostomata | Teleostomi | Euteleostomi | Sarcopterygii | Tetrapoda | Amphibia | Batrachia | Anura | Pipoidea | Pipidae | Xenopodinae | Xenopus | Silurana
Reference/s:
DOE Joint Genome Institute: Xenopus tropicalis
International Species Sequencing Consortium: Xenopus Sequencing Consortium
National Center for Biotechnology Information: Xenopus Gene Collection
National Center for Biotechnology Information: NCBI Eukaryotic Genomes Project
National Center for Biotechnology Information Reference Sequences: Xenopus (Silurana) tropicalis (western clawed frog) genome view
The Gene Index Project: DFCI Xenopus tropicalis Gene Index
The Genome Sequencing Center at Washington University: Xenopus tropicalis
UCSC Genome Bioinformatics: Xenopus tropicalis Genome Browser Gateway
Xenbase: a Xenopus web resource: Xenopus laevis and tropicalis biology and genomics resource
e! Ensembl: X.tropicalis
Publication/s:
Hellsten U *et. al.* , *Science* , **328** , 633 (2010).
Gerhard DS *et. al.* , *Genome Res* , **14** , 2121 (2004).
Strausberg RL *et. al.* , *Proc Natl Acad Sci U S A* , **99** , 16899 (2002).

***Xenopus laevis* (Xl)**
Taxonomy:
cellular organisms | Eukaryota | Opisthokonta | Metazoa | Eumetazoa | Bilateria | Coelomata | Deuterostomia | Chordata | Craniata | Vertebrata | Gnathostomata | Teleostomi | Euteleostomi | Sarcopterygii | Tetrapoda | Amphibia | Batrachia | Anura | Pipoidea | Pipidae | Xenopodinae | Xenopus | Xenopus
Reference/s:
GenBank - NIH genetic sequence database: GenBank species TBLASTN
National Center for Biotechnology Information: Xenopus Gene Collection
The Gene Index Project: DFCI African clawed frog (Xenopus laevis) Gene Index
Xenbase: a Xenopus web resource: Xenopus laevis and tropicalis biology and genomics resource
Publication/s:
Pollet N *et. al.* , *Mech Dev* , **122** , 365 (2005).
Gerhard DS *et. al.* , *Genome Res* , **14** , 2121 (2004).
Klein SL *et. al.* , *Dev Dyn* , **225** , 384 (2002).
Strausberg RL *et. al.* , *Proc Natl Acad Sci U S A* , **99** , 16899 (2002).
Blackshear PJ *et. al.* , *Gene* , **267** , 71 (2001).

***Ambystoma mexicanum* (Amm)**
Taxonomy:
cellular organisms | Eukaryota | Opisthokonta | Metazoa | Eumetazoa | Bilateria | Coelomata | Deuterostomia | Chordata | Craniata | Vertebrata | Gnathostomata | Teleostomi | Euteleostomi | Sarcopterygii | Tetrapoda | Amphibia | Batrachia | Caudata | Salamandroidea | Ambystomatidae | Ambystoma
Reference/s:
GenBank - NIH genetic sequence database: GenBank species TBLASTN
Publication/s:
Putta S *et. al.* , *BMC Genomics* , **5** , 54 (2004).

***Ambystoma tigrinum tigrinum* (Att)**
Taxonomy:
cellular organisms | Eukaryota | Opisthokonta | Metazoa | Eumetazoa | Bilateria | Coelomata | Deuterostomia | Chordata | Craniata | Vertebrata | Gnathostomata | Teleostomi | Euteleostomi | Sarcopterygii | Tetrapoda | Amphibia | Batrachia | Caudata | Salamandroidea | Ambystomatidae | Ambystoma | Ambystoma tigrinum
Reference/s:
GenBank - NIH genetic sequence database: GenBank species TBLASTN
Publication/s:
Putta S *et. al.* , *BMC Genomics* , **5** , 54 (2004).

***Lethenteron japonicum* (Lj)**
Taxonomy:
cellular organisms | Eukaryota | Opisthokonta | Metazoa | Eumetazoa | Bilateria | Coelomata | Deuterostomia | Chordata | Craniata | Vertebrata | Hyperoartia |

Petromyzontiformes | Petromyzontidae | Lethenteron

***Petromyzon marinus* (Ptm)**
Taxonomy:
cellular organisms | Eukaryota | Opisthokonta | Metazoa | Eumetazoa | Bilateria | Coelomata | Deuterostomia | Chordata | Craniata | Vertebrata | Hyperoartia | Petromyzontiformes | Petromyzontidae | Petromyzon
Reference/s:
The Genome Sequencing Center at Washington University: Petromyzon marinus
UCSC Genome Bioinformatics: Lamprey (Petromyzon marinus) Genome Browser Gateway

***Ciona savignyi* (Cis)**
Taxonomy:
cellular organisms | Eukaryota | Opisthokonta | Metazoa | Eumetazoa | Bilateria | Coelomata | Deuterostomia | Chordata | Tunicata | Ascidiacea | Enterogona | Phlebobranchia | Cionidae | Ciona
Reference/s:
Broad Institute of Harvard and MIT: Ciona savignyi Database
International Species Sequencing Consortium: Ciona savignyi Sequencing Consortium
National Center for Biotechnology Information: NCBI Eukaryotic Genomes Project
e! Ensembl: C.savignyi
Publication/s:
Small KS *et. al.* , *Genome Biol* , **8** , R41 (2007).

***Ciona intestinalis* (Ci)**
Taxonomy:
cellular organisms | Eukaryota | Opisthokonta | Metazoa | Eumetazoa | Bilateria | Coelomata | Deuterostomia | Chordata | Tunicata | Ascidiacea | Enterogona | Phlebobranchia | Cionidae | Ciona
Reference/s:
DOE Joint Genome Institute: Ciona intestinalis v2.0
DOE Joint Genome Institute: Ciona intestinalis
GenBank - NIH genetic sequence database: GenBank species TBLASTN
Ghost Database: Ciona intestinalis genomic and cDNA resources
International Species Sequencing Consortium: Ciona intestinalis Sequencing Consortium
National Center for Biotechnology Information: NCBI Eukaryotic Genomes Project
National Center for Biotechnology Information Reference Sequences: Ciona intestinalis genome view
The Gene Index Project: DFCI Ciona intestinalis Gene Index
UCSC Genome Bioinformatics: Ciona intestinalias Genome Browser Gateway
e! Ensembl: C.intestinalis
Publication/s:
Satou Y *et. al.* , *Genome Biol* , **9** , R152 (2008).
Dehal P *et. al.* , *Science* , **298** , 2157 (2002).
Satou Y *et. al.* , *Genesis* , **33** , 153 (2002).

***Molgula tectiformis* (Mot)**
Taxonomy:
cellular organisms | Eukaryota | Opisthokonta | Metazoa | Eumetazoa | Bilateria | Coelomata | Deuterostomia | Chordata | Tunicata | Ascidiacea | Stolidobranchia | Molgulidae | Molgula
Reference/s:
GenBank - NIH genetic sequence database: GenBank species TBLASTN
Publication/s:
Gyoja F *et. al.* , *Dev Biol* , **307** , 460 (2007).

***Halocynthia roretzi* (Hr)**
Taxonomy:
cellular organisms | Eukaryota | Opisthokonta | Metazoa | Eumetazoa | Bilateria | Coelomata | Deuterostomia | Chordata | Tunicata | Ascidiacea | Stolidobranchia | Pyuridae | Halocynthia

***Asterina pectinifera* (Asp)**
Taxonomy:
cellular organisms | Eukaryota | Opisthokonta | Metazoa | Eumetazoa | Bilateria | Coelomata | Deuterostomia | Echinodermata | Eleutherozoa | Asterozoa | Asteroidea | Valvatacea | Valvatida | Asterinidae | Patiria

***Paracentrotus lividus* (Pl)**
Taxonomy:
cellular organisms | Eukaryota | Opisthokonta | Metazoa | Eumetazoa | Bilateria | Coelomata | Deuterostomia | Echinodermata | Eleutherozoa | Echinozoa | Echinoidea | Euechinoidea | Echinacea | Echinoida | Echinidae | Paracentrotus

***Strongylocentrotus purpuratus* (Stp)**
Taxonomy:
cellular organisms | Eukaryota | Opisthokonta | Metazoa | Eumetazoa | Bilateria | Coelomata | Deuterostomia | Echinodermata | Eleutherozoa | Echinozoa | Echinoidea | Euechinoidea | Echinacea | Echinoida | Strongylocentrotidae | Strongylocentrotus
Reference/s:
GenBank - NIH genetic sequence database: GenBank species TBLASTN
Human Genome Sequencing Center at Baylor College of Medicine: Sea Urchin Genome Project
International Species Sequencing Consortium: Strongylocentrotus purpuratus Sequencing Consortium
National Center for Biotechnology Information: NCBI Eukaryotic Genomes Project
UCSC Genome Bioinformatics: Strongylocentrotus purpuratus Genome Browser Gateway
Publication/s:
Sodergren E *et. al.* , *Science* , **314** , 941 (2006).
Poustka AJ *et. al.* , *Genome Res* , **13** , 2736 (2003).

***Saccoglossus kowalevskii* (Sck)**
Taxonomy:
cellular organisms | Eukaryota | Opisthokonta | Metazoa | Eumetazoa | Bilateria | Coelomata | Deuterostomia | Hemichordata | Enteropneusta | Harrimaniidae | Saccoglossus
Reference/s:
Human Genome Sequencing Center at Baylor College of Medicine: Acorn Worm Genome Project
National Center for Biotechnology Information: NCBI Eukaryotic Genomes Project
National Center for Biotechnology Information Reference Sequences: Saccoglossus kowalevskii genome view

***Helobdella robusta* (Her)**
Taxonomy:
cellular organisms | Eukaryota | Opisthokonta | Metazoa | Eumetazoa | Bilateria | Coelomata | Protostomia | Annelida/Echiura/Pogonophora group | Annelida | Clitellata | Hirudinida | Hirudinea | Rhynchobdellida | Glossiphoniidae | Helobdella
Reference/s:
DOE Joint Genome Institute: Helobdella robusta

***Platynereis dumerilii* (Pld)**
Taxonomy:
cellular organisms | Eukaryota | Opisthokonta | Metazoa | Eumetazoa | Bilateria | Coelomata | Protostomia | Annelida/Echiura/Pogonophora group | Annelida | Polychaeta | Palpata | Aciculata | Phyllodocida | Nereididae | Platynereis
Reference/s:
GenBank - NIH genetic sequence database: GenBank species TBLASTN

***Capitella teleta* (Cpt)**
Taxonomy:
cellular organisms | Eukaryota | Opisthokonta | Metazoa | Eumetazoa | Bilateria | Coelomata | Protostomia | Annelida/Echiura/Pogonophora group | Annelida | Polychaeta | Scolecida | Capitellida | Capitellidae | Capitella
Reference/s:
DOE Joint Genome Institute: Capitella sp. I

***Mytilus californianus* (Myc)**
Taxonomy:
cellular organisms | Eukaryota | Opisthokonta | Metazoa | Eumetazoa | Bilateria | Coelomata | Protostomia | Mollusca | Bivalvia | Pteriomorphia | Mytiloida | Mytiloidea | Mytilidae | Mytilinae | Mytilus

***Crassostrea virginica* (Cv)**
Taxonomy:
cellular organisms | Eukaryota | Opisthokonta | Metazoa | Eumetazoa | Bilateria | Coelomata | Protostomia | Mollusca | Bivalvia | Pteriomorphia | Ostreoida | Ostreoidea | Ostreidae | Crassostrea
Reference/s:
BaNG Nematode and Neglected Genomics: MolluscDB
The Marine Genomics Project: Crassostrea virginica

***Argopecten irradians* (Ai)**
Taxonomy:
cellular organisms | Eukaryota | Opisthokonta | Metazoa | Eumetazoa | Bilateria | Coelomata | Protostomia | Mollusca | Bivalvia | Pteriomorphia | Pectinoida | Pectinoidea | Pectinidae | Argopecten
Reference/s:
BaNG Nematode and Neglected Genomics: Argopecten
The Marine Genomics Project: Argopecten irradians

***Euprymna scolopes* (Eus)**
Taxonomy:
cellular organisms | Eukaryota | Opisthokonta | Metazoa | Eumetazoa | Bilateria | Coelomata | Protostomia | Mollusca | Cephalopoda | Coleoidea | Neocoleoidea | Decapodiformes | Sepiolida | Sepiolidae | Euprymna

***Aplysia californica* (Apc)**
Taxonomy:
cellular organisms | Eukaryota | Opisthokonta | Metazoa | Eumetazoa | Bilateria | Coelomata | Protostomia | Mollusca | Gastropoda | Heterobranchia | Euthyneura | Euopisthobranchia | Aplysiomorpha | Aplysioidea | Aplysiidae | Aplysia
Reference/s:
Broad Institute of Harvard and MIT: Aplysia Genome Sequencing Project
National Center for Biotechnology Information: NCBI Eukaryotic Genomes Project
UCSC Genome Bioinformatics: Sea hare (Aplysia californica) Genome Browser Gateway

***Lottia gigantea* (Lg)**
Taxonomy:
cellular organisms | Eukaryota | Opisthokonta | Metazoa | Eumetazoa | Bilateria | Coelomata | Protostomia | Mollusca | Gastropoda | Patellogastropoda | Lottioidea | Lottiidae | Lottia
Reference/s:
DOE Joint Genome Institute: Lottia gigantea

***Tyrophagus putrescentiae* (Typ)**
Taxonomy:
cellular organisms | Eukaryota | Opisthokonta | Metazoa | Eumetazoa | Bilateria | Coelomata | Protostomia | Panarthropoda | Arthropoda | Chelicerata | Arachnida | Acari | Acariformes | Sarcoptiformes | Astigmata | Acaroidea | Acaridae | Tyrophaginae | Tyrophagus

***Ornithodoros moubata* (Onm)**
Taxonomy:
cellular organisms | Eukaryota | Opisthokonta | Metazoa | Eumetazoa | Bilateria | Coelomata | Protostomia | Panarthropoda | Arthropoda | Chelicerata | Arachnida | Acari | Parasitiformes | Ixodida | Ixodoidea | Argasidae | Ornithodorinae | Ornithodoros

Amblyomma americanum **(Ama)**
Taxonomy:
cellular organisms | Eukaryota | Opisthokonta | Metazoa | Eumetazoa | Bilateria | Coelomata | Protostomia | Panarthropoda | Arthropoda | Chelicerata | Arachnida | Acari | Parasitiformes | Ixodida | Ixodoidea | Ixodidae | Amblyomminae | Amblyomma
Reference/s:
BaNG Nematode and Neglected Genomics: CHELICERATA

Haemaphysalis longicornis **(Hml)**
Taxonomy:
cellular organisms | Eukaryota | Opisthokonta | Metazoa | Eumetazoa | Bilateria | Coelomata | Protostomia | Panarthropoda | Arthropoda | Chelicerata | Arachnida | Acari | Parasitiformes | Ixodida | Ixodoidea | Ixodidae | Haemaphysalinae | Haemaphysalis
Reference/s:
GenBank - NIH genetic sequence database: GenBank species TBLASTN
Publication/s:
da Silva Vaz I Jr *et. al.* , *Vet Parasitol* , **127** , 147 (2005).

Ixodes ricinus **(Ixr)**
Taxonomy:
cellular organisms | Eukaryota | Opisthokonta | Metazoa | Eumetazoa | Bilateria | Coelomata | Protostomia | Panarthropoda | Arthropoda | Chelicerata | Arachnida | Acari | Parasitiformes | Ixodida | Ixodoidea | Ixodidae | Ixodinae | Ixodes

Ixodes scapularis str. Wikel **(Is)**
Taxonomy:
cellular organisms | Eukaryota | Opisthokonta | Metazoa | Eumetazoa | Bilateria | Coelomata | Protostomia | Panarthropoda | Arthropoda | Chelicerata | Arachnida | Acari | Parasitiformes | Ixodida | Ixodoidea | Ixodidae | Ixodinae | Ixodes
Reference/s:
National Center for Biotechnology Information: NCBI Insect Genomes Project
Purdue University: The Ixodes scapularis Genome Project
The Gene Index Project: DFCI Black tick Gene Index
VectorBase: Ixodes scapularis@vectorbase.org
e! Ensembl: Ixodes scapularis

Boophilus microplus **(Bom)**
Taxonomy:
cellular organisms | Eukaryota | Opisthokonta | Metazoa | Eumetazoa | Bilateria | Coelomata | Protostomia | Panarthropoda | Arthropoda | Chelicerata | Arachnida | Acari | Parasitiformes | Ixodida | Ixodoidea | Ixodidae | Rhipicephalinae | Rhipicephalus | Boophilus
Reference/s:
BaNG Nematode and Neglected Genomics: CHELICERATA
GenBank - NIH genetic sequence database: GenBank species TBLASTN
International Species Sequencing Consortium: Boophilus Sequencing Consortium
National Center for Biotechnology Information: NCBI Insect Genomes Project
The Gene Index Project: DFCI Boophilus microplus Gene Index
Publication/s:
Guerrero FD *et. al.* , *BMC Genomics* , **11** , 374 (2010).
da Silva Vaz I Jr *et. al.* , *Vet Parasitol* , **127** , 147 (2005).
Guerrero FD *et. al.* , *Insect Biochem Mol Biol* , **35** , 585 (2005).

Rhipicephalus appendiculatus **(Ra)**
Taxonomy:
cellular organisms | Eukaryota | Opisthokonta | Metazoa | Eumetazoa | Bilateria | Coelomata | Protostomia | Panarthropoda | Arthropoda | Chelicerata | Arachnida | Acari | Parasitiformes | Ixodida | Ixodoidea | Ixodidae | Rhipicephalinae | Rhipicephalus | Rhipicephalus
Reference/s:
BaNG Nematode and Neglected Genomics: CHELICERATA
The Gene Index Project: DFCI Rhipicephalus appendiculatus Gene Index
Publication/s:
Nene V *et. al.* , *Insect Biochem Mol Biol* , **34** , 1117 (2004).

Limulus polyphemus **(Lip)**
Taxonomy:
cellular organisms | Eukaryota | Opisthokonta | Metazoa | Eumetazoa | Bilateria | Coelomata | Protostomia | Panarthropoda | Arthropoda | Chelicerata | Merostomata | Xiphosura | Limulidae | Limulus
Reference/s:
GenBank - NIH genetic sequence database: GenBank species TBLASTN
Publication/s:
Way M *et. al.* , *J Cell Biol* , **128** , 51 (1995).

Daphnia magna **(Dam)**
Taxonomy:
cellular organisms | Eukaryota | Opisthokonta | Metazoa | Eumetazoa | Bilateria | Coelomata | Protostomia | Panarthropoda | Arthropoda | Mandibulata | Pancrustacea | Crustacea | Branchiopoda | Phyllopoda | Diplostraca | Cladocera | Anomopoda | Daphniidae | Daphnia
Reference/s:
BaNG Nematode and Neglected Genomics: CRUSTACEA
GenBank - NIH genetic sequence database: GenBank species TBLASTN
wFleaBase: Daphnia Water Flea Genome Database
Publication/s:
Watanabe H *et. al.* , *Genome* , **48** , 606 (2005).

Daphnia pulex **(Dap)**
Taxonomy:
cellular organisms | Eukaryota | Opisthokonta | Metazoa | Eumetazoa | Bilateria | Coelomata | Protostomia | Panarthropoda | Arthropoda | Mandibulata | Pancrustacea |

Crustacea | Branchiopoda | Phyllopoda | Diplostraca | Cladocera | Anomopoda | Daphniidae | Daphnia
Reference/s:
BaNG Nematode and Neglected Genomics: CRUSTACEA
DOE Joint Genome Institute: Daphnia Genome Sequencing
International Species Sequencing Consortium: Daphnia Sequencing Consortium
National Center for Biotechnology Information: NCBI Insect Genomes Project
wFleaBase: Daphnia Water Flea Genome Database
Publication/s:
Colbourne JK *et. al.* , *Science* , **331** , 555 (2011).

Artemia franciscana **(Arf)**
Taxonomy:
cellular organisms | Eukaryota | Opisthokonta | Metazoa | Eumetazoa | Bilateria | Coelomata | Protostomia | Panarthropoda | Arthropoda | Mandibulata | Pancrustacea | Crustacea | Branchiopoda | Sarsostraca | Anostraca | Artemiidae | Artemia
Reference/s:
BaNG Nematode and Neglected Genomics: CRUSTACEA

Artemia sp. **(Ats)**
Taxonomy:
cellular organisms | Eukaryota | Opisthokonta | Metazoa | Eumetazoa | Bilateria | Coelomata | Protostomia | Panarthropoda | Arthropoda | Mandibulata | Pancrustacea | Crustacea | Branchiopoda | Sarsostraca | Anostraca | Artemiidae | Artemia

Fenneropenaeus chinensis **(Fec)**
Taxonomy:
cellular organisms | Eukaryota | Opisthokonta | Metazoa | Eumetazoa | Bilateria | Coelomata | Protostomia | Panarthropoda | Arthropoda | Mandibulata | Pancrustacea | Crustacea | Malacostraca | Eumalacostraca | Eucarida | Decapoda | Dendrobranchiata | Penaeoidea | Penaeidae | Fenneropenaeus
Reference/s:
GenBank - NIH genetic sequence database: GenBank species TBLASTN
Publication/s:
Zhang J *et. al.* , *J Biotechnol* , **125** , 173 (2006).

Litopenaeus vannamei **(Liv)**
Taxonomy:
cellular organisms | Eukaryota | Opisthokonta | Metazoa | Eumetazoa | Bilateria | Coelomata | Protostomia | Panarthropoda | Arthropoda | Mandibulata | Pancrustacea | Crustacea | Malacostraca | Eumalacostraca | Eucarida | Decapoda | Dendrobranchiata | Penaeoidea | Penaeidae | Litopenaeus
Reference/s:
BaNG Nematode and Neglected Genomics: CRUSTACEA
The Marine Genomics Project: Litopenaeus vannamei

Litopenaeus setiferus **(Los)**
Taxonomy:
cellular organisms | Eukaryota | Opisthokonta | Metazoa | Eumetazoa | Bilateria | Coelomata | Protostomia | Panarthropoda | Arthropoda | Mandibulata | Pancrustacea | Crustacea | Malacostraca | Eumalacostraca | Eucarida | Decapoda | Dendrobranchiata | Penaeoidea | Penaeidae | Litopenaeus
Reference/s:
BaNG Nematode and Neglected Genomics: CRUSTACEA
GenBank - NIH genetic sequence database: GenBank species TBLASTN
The Marine Genomics Project: Litopenaeus setiferus
Publication/s:
Gross PS *et. al.* , *Dev Comp Immunol* , **25** , 565 (2001).

Marsupenaeus japonicus **(Mrj)**
Taxonomy:
cellular organisms | Eukaryota | Opisthokonta | Metazoa | Eumetazoa | Bilateria | Coelomata | Protostomia | Panarthropoda | Arthropoda | Mandibulata | Pancrustacea | Crustacea | Malacostraca | Eumalacostraca | Eucarida | Decapoda | Dendrobranchiata | Penaeoidea | Penaeidae | Marsupenaeus
Reference/s:
BaNG Nematode and Neglected Genomics: CRUSTACEA

Pacifastacus leniusculus **(Pcl)**
Taxonomy:
cellular organisms | Eukaryota | Opisthokonta | Metazoa | Eumetazoa | Bilateria | Coelomata | Protostomia | Panarthropoda | Arthropoda | Mandibulata | Pancrustacea | Crustacea | Malacostraca | Eumalacostraca | Eucarida | Decapoda | Pleocyemata | Astacidea | Astacoidea | Astacidae | Pacifastacus

Homarus americanus **(Ha)**
Taxonomy:
cellular organisms | Eukaryota | Opisthokonta | Metazoa | Eumetazoa | Bilateria | Coelomata | Protostomia | Panarthropoda | Arthropoda | Mandibulata | Pancrustacea | Crustacea | Malacostraca | Eumalacostraca | Eucarida | Decapoda | Pleocyemata | Astacidea | Nephropoidea | Nephropidae | Homarus
Reference/s:
BaNG Nematode and Neglected Genomics: CRUSTACEA
The Marine Genomics Project: Homarus americanus

Homarus gammarus **(Hg)**
Taxonomy:
cellular organisms | Eukaryota | Opisthokonta | Metazoa | Eumetazoa | Bilateria | Coelomata | Protostomia | Panarthropoda | Arthropoda | Mandibulata | Pancrustacea | Crustacea | Malacostraca | Eumalacostraca | Eucarida | Decapoda | Pleocyemata | Astacidea | Nephropoidea | Nephropidae | Homarus

Callinectes sapidus **(Cas)**
Taxonomy:
cellular organisms | Eukaryota | Opisthokonta | Metazoa | Eumetazoa | Bilateria | Coelomata | Protostomia | Panarthropoda | Arthropoda | Mandibulata | Pancrustacea | Crustacea | Malacostraca | Eumalacostraca | Eucarida | Decapoda | Pleocyemata | Brachyura | Eubrachyura | Heterotremata/Thoracotremata group | Heterotremata | Portunoidea | Portunidae | Callinectes

Reference/s:
BaNG Nematode and Neglected Genomics: CRUSTACEA
The Marine Genomics Project: Callinectes sapidus

Carcinus maenas **(Cam)**
Taxonomy:
cellular organisms | Eukaryota | Opisthokonta | Metazoa | Eumetazoa | Bilateria | Coelomata | Protostomia | Panarthropoda | Arthropoda | Mandibulata | Pancrustacea | Crustacea | Malacostraca | Eumalacostraca | Eucarida | Decapoda | Pleocyemata | Brachyura | Eubrachyura | Heterotremata/Thoracotremata group | Heterotremata | Portunoidea | Portunidae | Carcinus
Reference/s:
BaNG Nematode and Neglected Genomics: CRUSTACEA

Macrobrachium rosenbergii **(Mar)**
Taxonomy:
cellular organisms | Eukaryota | Opisthokonta | Metazoa | Eumetazoa | Bilateria | Coelomata | Protostomia | Panarthropoda | Arthropoda | Mandibulata | Pancrustacea | Crustacea | Malacostraca | Eumalacostraca | Eucarida | Decapoda | Pleocyemata | Caridea | Palaemonoidea | Palaemonidae | Macrobrachium

Calanus finmarchicus **(Cnf)**
Taxonomy:
cellular organisms | Eukaryota | Opisthokonta | Metazoa | Eumetazoa | Bilateria | Coelomata | Protostomia | Panarthropoda | Arthropoda | Mandibulata | Pancrustacea | Crustacea | Maxillopoda | Copepoda | Neocopepoda | Gymnoplea | Calanoida | Calanidae | Calanus
Reference/s:
The Marine Genomics Project: Calanus finmarchicus

Lepeophtheirus salmonis **(Lhs)**
Taxonomy:
cellular organisms | Eukaryota | Opisthokonta | Metazoa | Eumetazoa | Bilateria | Coelomata | Protostomia | Panarthropoda | Arthropoda | Mandibulata | Pancrustacea | Crustacea | Maxillopoda | Copepoda | Neocopepoda | Podoplea | Siphonostomatoida | Caligidae | Lepeophtheirus
Reference/s:
National Center for Biotechnology Information: NCBI Insect Genomes Project

Bombyx mori 703 **(Bm_c)**
Taxonomy:
cellular organisms | Eukaryota | Opisthokonta | Metazoa | Eumetazoa | Bilateria | Coelomata | Protostomia | Panarthropoda | Arthropoda | Mandibulata | Pancrustacea | Hexapoda | Insecta | Dicondylia | Pterygota | Neoptera | Endopterygota | Amphiesmenoptera | Lepidoptera | Glossata | Neolepidoptera | Heteroneura | Ditrysia | Obtectomera | Bombycoidea | Bombyciformes | Bombycidae | Bombycinae | Bombyx

Bombyx mori str. Dazao **(Bm_b)**
Taxonomy:
cellular organisms | Eukaryota | Opisthokonta | Metazoa | Eumetazoa | Bilateria | Coelomata | Protostomia | Panarthropoda | Arthropoda | Mandibulata | Pancrustacea | Hexapoda | Insecta | Dicondylia | Pterygota | Neoptera | Endopterygota | Amphiesmenoptera | Lepidoptera | Glossata | Neolepidoptera | Heteroneura | Ditrysia | Obtectomera | Bombycoidea | Bombyciformes | Bombycidae | Bombycinae | Bombyx
Reference/s:
International Species Sequencing Consortium: Bombyx mori Sequencing Consortium
National Center for Biotechnology Information: NCBI Insect Genomes Project
SilkBase: EST Database of the Silkworm, Bombyx mori
SilkDB: Silkworm Knowledgebase: Bombyx mori project
Publication/s:
Xia *et. al.* , *Science* , **306** , 1937 (2004).
Mita K *et. al.* , *Proc Natl Acad Sci U S A* , **100** , 14121 (2003).

Bombyx mori p50T **(Bm_a)**
Taxonomy:
cellular organisms | Eukaryota | Opisthokonta | Metazoa | Eumetazoa | Bilateria | Coelomata | Protostomia | Panarthropoda | Arthropoda | Mandibulata | Pancrustacea | Hexapoda | Insecta | Dicondylia | Pterygota | Neoptera | Endopterygota | Amphiesmenoptera | Lepidoptera | Glossata | Neolepidoptera | Heteroneura | Ditrysia | Obtectomera | Bombycoidea | Bombyciformes | Bombycidae | Bombycinae | Bombyx
Reference/s:
National Center for Biotechnology Information: NCBI Insect Genomes Project
SilkBase: EST Database of the Silkworm, Bombyx mori
The Silkworm Genome Research Program: Bombyx mori project
Publication/s:
Mita K *et. al.* , *DNA Res* , **11** , 27 (2004).

Bombyx mori **(Bm)**
Taxonomy:
cellular organisms | Eukaryota | Opisthokonta | Metazoa | Eumetazoa | Bilateria | Coelomata | Protostomia | Panarthropoda | Arthropoda | Mandibulata | Pancrustacea | Hexapoda | Insecta | Dicondylia | Pterygota | Neoptera | Endopterygota | Amphiesmenoptera | Lepidoptera | Glossata | Neolepidoptera | Heteroneura | Ditrysia | Obtectomera | Bombycoidea | Bombyciformes | Bombycidae | Bombycinae | Bombyx
Reference/s:
International Species Sequencing Consortium: Bombyx mori Sequencing Consortium
National Center for Biotechnology Information: NCBI Insect Genomes Project
SilkBase: EST Database of the Silkworm, Bombyx mori
SilkDB: Silkworm Knowledgebase: Bombyx mori project
Publication/s:
Xia Q *et. al.* , *Insect Biochem Mol Biol* , **38** , 1036 (2008).

Lonomia obliqua **(Loo)**
Taxonomy:
cellular organisms | Eukaryota | Opisthokonta | Metazoa | Eumetazoa | Bilateria | Coelomata | Protostomia | Panarthropoda | Arthropoda | Mandibulata | Pancrustacea | Hexapoda | Insecta | Dicondylia | Pterygota | Neoptera | Endopterygota | Amphiesmenoptera | Lepidoptera | Glossata | Neolepidoptera | Heteroneura | Ditrysia | Obtectomera |

Bombycoidea | Saturniiformes | Saturniidae | Hemileucinae | Lonomia

***Manduca sexta* (Ms)**
Taxonomy:
cellular organisms | Eukaryota | Opisthokonta | Metazoa | Eumetazoa | Bilateria | Coelomata | Protostomia | Panarthropoda | Arthropoda | Mandibulata | Pancrustacea | Hexapoda | Insecta | Dicondylia | Pterygota | Neoptera | Endopterygota | Amphiesmenoptera | Lepidoptera | Glossata | Neolepidoptera | Heteroneura | Ditrysia | Obtectomera | Bombycoidea | Sphingidae | Sphinginae | Sphingini | Manduca
Reference/s:
Agricultural Pest Genomics Resource Database: Manduca Base
GenBank - NIH genetic sequence database: GenBank species TBLASTN
National Center for Biotechnology Information: NCBI Insect Genomes Project
Publication/s:
Vitavska O, Wieczorek H, Merzendorfer H , *J Biol Chem* , **278** , 18499 (2003).

***Orgyia thyellina* (Ort)**
Taxonomy:
cellular organisms | Eukaryota | Opisthokonta | Metazoa | Eumetazoa | Bilateria | Coelomata | Protostomia | Panarthropoda | Arthropoda | Mandibulata | Pancrustacea | Hexapoda | Insecta | Dicondylia | Pterygota | Neoptera | Endopterygota | Amphiesmenoptera | Lepidoptera | Glossata | Neolepidoptera | Heteroneura | Ditrysia | Obtectomera | Noctuoidea | Lymantriidae | Orgyia

***Spodoptera frugiperda* (Sf)**
Taxonomy:
cellular organisms | Eukaryota | Opisthokonta | Metazoa | Eumetazoa | Bilateria | Coelomata | Protostomia | Panarthropoda | Arthropoda | Mandibulata | Pancrustacea | Hexapoda | Insecta | Dicondylia | Pterygota | Neoptera | Endopterygota | Amphiesmenoptera | Lepidoptera | Glossata | Neolepidoptera | Heteroneura | Ditrysia | Obtectomera | Noctuoidea | Noctuidae | Amphipyrinae | Spodoptera
Reference/s:
GenBank - NIH genetic sequence database: GenBank species TBLASTN
SPODOBASE: Spodoptera frugiperda
Publication/s:
Deng Y *et. al.* , *BMC Genomics* , **7** , 264 (2006).

***Heliothis virescens* (Hev)**
Taxonomy:
cellular organisms | Eukaryota | Opisthokonta | Metazoa | Eumetazoa | Bilateria | Coelomata | Protostomia | Panarthropoda | Arthropoda | Mandibulata | Pancrustacea | Hexapoda | Insecta | Dicondylia | Pterygota | Neoptera | Endopterygota | Amphiesmenoptera | Lepidoptera | Glossata | Neolepidoptera | Heteroneura | Ditrysia | Obtectomera | Noctuoidea | Noctuidae | Heliothinae | Heliothis
Reference/s:
GenBank - NIH genetic sequence database: GenBank species TBLASTN
Publication/s:
Borovsky D *et. al.* , *Proc Natl Acad Sci U S A* , **103** , 18963 (2006).

***Heliconius erato* (He)**
Taxonomy:
cellular organisms | Eukaryota | Opisthokonta | Metazoa | Eumetazoa | Bilateria | Coelomata | Protostomia | Panarthropoda | Arthropoda | Mandibulata | Pancrustacea | Hexapoda | Insecta | Dicondylia | Pterygota | Neoptera | Endopterygota | Amphiesmenoptera | Lepidoptera | Glossata | Neolepidoptera | Heteroneura | Ditrysia | Obtectomera | Papilionoidea | Nymphalidae | Heliconiinae | Heliconiini | Heliconius
Reference/s:
GenBank - NIH genetic sequence database: GenBank species TBLASTN
Publication/s:
Pringle EG *et. al.* , *Genetics* , **177** , 417 (2007).
Papanicolaou A *et. al.* , *Mol Ecol* , **14** , 2883 (2005).

***Papilio dardanus* (Pad)**
Taxonomy:
cellular organisms | Eukaryota | Opisthokonta | Metazoa | Eumetazoa | Bilateria | Coelomata | Protostomia | Panarthropoda | Arthropoda | Mandibulata | Pancrustacea | Hexapoda | Insecta | Dicondylia | Pterygota | Neoptera | Endopterygota | Amphiesmenoptera | Lepidoptera | Glossata | Neolepidoptera | Heteroneura | Ditrysia | Obtectomera | Papilionoidea | Papilionidae | Papilioninae | Papilionini | Papilio
Reference/s:
BaNG Nematode and Neglected Genomics: HEXAPODA

***Plutella xylostella* (Px)**
Taxonomy:
cellular organisms | Eukaryota | Opisthokonta | Metazoa | Eumetazoa | Bilateria | Coelomata | Protostomia | Panarthropoda | Arthropoda | Mandibulata | Pancrustacea | Hexapoda | Insecta | Dicondylia | Pterygota | Neoptera | Endopterygota | Amphiesmenoptera | Lepidoptera | Glossata | Neolepidoptera | Heteroneura | Ditrysia | Yponomeutoidea | Plutellidae | Plutella

***Apriona germari* (Apg)**
Taxonomy:
cellular organisms | Eukaryota | Opisthokonta | Metazoa | Eumetazoa | Bilateria | Coelomata | Protostomia | Panarthropoda | Arthropoda | Mandibulata | Pancrustacea | Hexapoda | Insecta | Dicondylia | Pterygota | Neoptera | Endopterygota | Coleoptera | Polyphaga | Cucujiformia | Chrysomeloidea | Cerambycidae | Lamiinae | Batocerini | Apriona

***Diabrotica virgifera virgifera* (Dvv)**
Taxonomy:
cellular organisms | Eukaryota | Opisthokonta | Metazoa | Eumetazoa | Bilateria | Coelomata | Protostomia | Panarthropoda | Arthropoda | Mandibulata | Pancrustacea | Hexapoda | Insecta | Dicondylia | Pterygota | Neoptera | Endopterygota | Coleoptera | Polyphaga | Cucujiformia | Chrysomeloidea | Chrysomelidae | Galerucinae | Luperini | Diabroticina | Diabroticites | Diabrotica | Diabrotica virgifera

***Tribolium castaneum str. Georgia GA2* (Tic)**
Taxonomy:

cellular organisms | Eukaryota | Opisthokonta | Metazoa | Eumetazoa | Bilateria | Coelomata | Protostomia | Panarthropoda | Arthropoda | Mandibulata | Pancrustacea | Hexapoda | Insecta | Dicondylia | Pterygota | Neoptera | Endopterygota | Coleoptera | Polyphaga | Cucujiformia | Tenebrionoidea | Tenebrionidae | Tribolium
Reference/s:
BeetleBase: Tribolium Genome Database
Human Genome Sequencing Center at Baylor College of Medicine: Tribolium castaneum Genome Project
International Species Sequencing Consortium: Tribolium castaneum Sequencing Consortium
National Center for Biotechnology Information: NCBI Insect Genomes Project
National Center for Biotechnology Information Reference Sequences: Tribolium castaneum (red flour beetle) genome view
Publication/s:
Richards S *et. al.* , *Nature* , **452** , 949 (2008).

Drosophila mojavensis TSC#15081-1352.22 **(Dmo)**
Taxonomy:
cellular organisms | Eukaryota | Opisthokonta | Metazoa | Eumetazoa | Bilateria | Coelomata | Protostomia | Panarthropoda | Arthropoda | Mandibulata | Pancrustacea | Hexapoda | Insecta | Dicondylia | Pterygota | Neoptera | Endopterygota | Diptera | Brachycera | Muscomorpha | Eremoneura | Cyclorrhapha | Schizophora | Acalyptratae | Ephydroidea | Drosophilidae | Drosophilinae | Drosophilini | Drosophilina | Drosophiliti | Drosophila | Drosophila | repleta group | mulleri subgroup | mojavensis species complex
Reference/s:
FlyBase: A Database of the Drosophila Genome
International Species Sequencing Consortium: Drosophila Sequencing Consortium
National Center for Biotechnology Information: NCBI Insect Genomes Project
UCSC Genome Bioinformatics: Drosophila mojavensis Genome Browser Gateway
e! Ensembl: Drosophila mojavensis
Publication/s:
Clark AG *et. al.* , *Nature* , **450** , 203 (2007).

Drosophila virilis TSC#15010-1051.87 **(Dv)**
Taxonomy:
cellular organisms | Eukaryota | Opisthokonta | Metazoa | Eumetazoa | Bilateria | Coelomata | Protostomia | Panarthropoda | Arthropoda | Mandibulata | Pancrustacea | Hexapoda | Insecta | Dicondylia | Pterygota | Neoptera | Endopterygota | Diptera | Brachycera | Muscomorpha | Eremoneura | Cyclorrhapha | Schizophora | Acalyptratae | Ephydroidea | Drosophilidae | Drosophilinae | Drosophilini | Drosophilina | Drosophiliti | Drosophila | Drosophila | virilis group
Reference/s:
FlyBase: A Database of the Drosophila Genome
International Species Sequencing Consortium: Drosophila Sequencing Consortium
National Center for Biotechnology Information: NCBI Insect Genomes Project
UCSC Genome Bioinformatics: Drosophila virilis Genome Browser Gateway
e! Ensembl: Drosophila virilis
Publication/s:
Clark AG *et. al.* , *Nature* , **450** , 203 (2007).

Drosophila grimshawi TSC#15287-2541.00 **(Dg)**
Taxonomy:
cellular organisms | Eukaryota | Opisthokonta | Metazoa | Eumetazoa | Bilateria | Coelomata | Protostomia | Panarthropoda | Arthropoda | Mandibulata | Pancrustacea | Hexapoda | Insecta | Dicondylia | Pterygota | Neoptera | Endopterygota | Diptera | Brachycera | Muscomorpha | Eremoneura | Cyclorrhapha | Schizophora | Acalyptratae | Ephydroidea | Drosophilidae | Drosophilinae | Drosophilini | Drosophilina | Drosophiliti | Drosophila | Hawaiian Drosophila | picture wing clade | grimshawi clade | grimshawi group | grimshawi subgroup
Reference/s:
FlyBase: A Database of the Drosophila Genome
International Species Sequencing Consortium: Drosophila Sequencing Consortium
National Center for Biotechnology Information: NCBI Insect Genomes Project
UCSC Genome Bioinformatics: Drosophila grimshawi Genome Browser Gateway
e! Ensembl: Drosophila grimshawi
Publication/s:
Clark AG *et. al.* , *Nature* , **450** , 203 (2007).

Drosophila ananassae TSC#14024-0371.13 **(Da)**
Taxonomy:
cellular organisms | Eukaryota | Opisthokonta | Metazoa | Eumetazoa | Bilateria | Coelomata | Protostomia | Panarthropoda | Arthropoda | Mandibulata | Pancrustacea | Hexapoda | Insecta | Dicondylia | Pterygota | Neoptera | Endopterygota | Diptera | Brachycera | Muscomorpha | Eremoneura | Cyclorrhapha | Schizophora | Acalyptratae | Ephydroidea | Drosophilidae | Drosophilinae | Drosophilini | Drosophilina | Drosophiliti | Drosophila | Sophophora | melanogaster group | ananassae subgroup | ananassae species complex
Reference/s:
FlyBase: A Database of the Drosophila Genome
International Species Sequencing Consortium: Drosophila Sequencing Consortium
National Center for Biotechnology Information: NCBI Insect Genomes Project
UCSC Genome Bioinformatics: Drosophila ananassae Genome Browser Gateway
e! Ensembl: Drosophila ananassae
Publication/s:
Clark AG *et. al.* , *Nature* , **450** , 203 (2007).

Drosophila melanogaster **(Dm)**
Taxonomy:
cellular organisms | Eukaryota | Opisthokonta | Metazoa | Eumetazoa | Bilateria | Coelomata | Protostomia | Panarthropoda | Arthropoda | Mandibulata | Pancrustacea | Hexapoda | Insecta | Dicondylia | Pterygota | Neoptera | Endopterygota | Diptera | Brachycera | Muscomorpha | Eremoneura | Cyclorrhapha | Schizophora | Acalyptratae | Ephydroidea | Drosophilidae | Drosophilinae | Drosophilini | Drosophilina | Drosophiliti | Drosophila | Sophophora | melanogaster group | melanogaster subgroup
Reference/s:
Berkeley Drosophila Genome Project: Berkeley Drosophila Genome Project
FlyBase: A Database of the Drosophila Genome
GenBank - NIH genetic sequence database: GenBank species TBLASTN

Human Genome Sequencing Center at Baylor College of Medicine: Drosophila Genome Project
International Species Sequencing Consortium: Drosophila melanogaster Sequencing Consortium
National Center for Biotechnology Information: Drosophila melanogaster Sequencing Project
National Center for Biotechnology Information Reference Sequences: Drosophila melanogaster (fruit fly) genome view
The Gene Index Project: DFCI Drosophila Gene Index
UCSC Genome Bioinformatics: Drosophila melanogaster Genome Browser Gateway
e! Ensembl: Fruitfly
Publication/s:
Misra S *et. al.* , *Genome Biol* , **3** , RESEARCH0083 (2002).
Celniker SE *et. al.* , *Genome Biol* , **3** , RESEARCH0079 (2002).
Andrews J *et. al.* , *Genome Res* , **10** , 2030 (2000).
Adams MD *et. al.* , *Science* , **287** , 2185 (2000).

Drosophila simulans str. sim4 **(Dss_d)**
Taxonomy:
cellular organisms | Eukaryota | Opisthokonta | Metazoa | Eumetazoa | Bilateria | Coelomata | Protostomia | Panarthropoda | Arthropoda | Mandibulata | Pancrustacea | Hexapoda | Insecta | Dicondylia | Pterygota | Neoptera | Endopterygota | Diptera | Brachycera | Muscomorpha | Eremoneura | Cyclorrhapha | Schizophora | Acalyptratae | Ephydroidea | Drosophilidae | Drosophilinae | Drosophilini | Drosophilina | Drosophiliti | Drosophila | Sophophora | melanogaster group | melanogaster subgroup
Reference/s:
International Species Sequencing Consortium: Drosophila Sequencing Consortium
National Center for Biotechnology Information: NCBI Insect Genomes Project
The Genome Sequencing Center at Washington University: Drosophila simulans Sequencing
Publication/s:
Clark AG *et. al.* , *Nature* , **450** , 203 (2007).

Drosophila simulans str. sim6 **(Dss_c)**
Taxonomy:
cellular organisms | Eukaryota | Opisthokonta | Metazoa | Eumetazoa | Bilateria | Coelomata | Protostomia | Panarthropoda | Arthropoda | Mandibulata | Pancrustacea | Hexapoda | Insecta | Dicondylia | Pterygota | Neoptera | Endopterygota | Diptera | Brachycera | Muscomorpha | Eremoneura | Cyclorrhapha | Schizophora | Acalyptratae | Ephydroidea | Drosophilidae | Drosophilinae | Drosophilini | Drosophilina | Drosophiliti | Drosophila | Sophophora | melanogaster group | melanogaster subgroup
Reference/s:
International Species Sequencing Consortium: Drosophila Sequencing Consortium
National Center for Biotechnology Information: NCBI Insect Genomes Project
The Genome Sequencing Center at Washington University: Drosophila simulans Sequencing
Publication/s:
Clark AG *et. al.* , *Nature* , **450** , 203 (2007).

Drosophila yakuba Tai18E2 **(Dy)**
Taxonomy:
cellular organisms | Eukaryota | Opisthokonta | Metazoa | Eumetazoa | Bilateria | Coelomata | Protostomia | Panarthropoda | Arthropoda | Mandibulata | Pancrustacea | Hexapoda | Insecta | Dicondylia | Pterygota | Neoptera | Endopterygota | Diptera | Brachycera | Muscomorpha | Eremoneura | Cyclorrhapha | Schizophora | Acalyptratae | Ephydroidea | Drosophilidae | Drosophilinae | Drosophilini | Drosophilina | Drosophiliti | Drosophila | Sophophora | melanogaster group | melanogaster subgroup
Reference/s:
FlyBase: A Database of the Drosophila Genome
GenBank - NIH genetic sequence database: GenBank species TBLASTN
International Species Sequencing Consortium: Drosophila Sequencing Consortium
National Center for Biotechnology Information: NCBI Insect Genomes Project
The Genome Sequencing Center at Washington University: Drosophila yakuba Sequencing
UCSC Genome Bioinformatics: Drosophila yakuba Genome Browser Gateway
e! Ensembl: Drosophila yakuba
Publication/s:
Clark AG *et. al.* , *Nature* , **450** , 203 (2007).
Domazet-Loso T, Tautz D , *Genome Res* , **13** , 2213 (2003).

Drosophila simulans str. md199 **(Dss_g)**
Taxonomy:
cellular organisms | Eukaryota | Opisthokonta | Metazoa | Eumetazoa | Bilateria | Coelomata | Protostomia | Panarthropoda | Arthropoda | Mandibulata | Pancrustacea | Hexapoda | Insecta | Dicondylia | Pterygota | Neoptera | Endopterygota | Diptera | Brachycera | Muscomorpha | Eremoneura | Cyclorrhapha | Schizophora | Acalyptratae | Ephydroidea | Drosophilidae | Drosophilinae | Drosophilini | Drosophilina | Drosophiliti | Drosophila | Sophophora | melanogaster group | melanogaster subgroup
Reference/s:
International Species Sequencing Consortium: Drosophila Sequencing Consortium
National Center for Biotechnology Information: NCBI Insect Genomes Project
The Genome Sequencing Center at Washington University: Drosophila simulans Sequencing
Publication/s:
Clark AG *et. al.* , *Nature* , **450** , 203 (2007).

Drosophila simulans str. nc48 **(Dss_e)**
Taxonomy:
cellular organisms | Eukaryota | Opisthokonta | Metazoa | Eumetazoa | Bilateria | Coelomata | Protostomia | Panarthropoda | Arthropoda | Mandibulata | Pancrustacea | Hexapoda | Insecta | Dicondylia | Pterygota | Neoptera | Endopterygota | Diptera | Brachycera | Muscomorpha | Eremoneura | Cyclorrhapha | Schizophora | Acalyptratae | Ephydroidea | Drosophilidae | Drosophilinae | Drosophilini | Drosophilina | Drosophiliti | Drosophila | Sophophora | melanogaster group | melanogaster subgroup
Reference/s:
International Species Sequencing Consortium: Drosophila Sequencing Consortium
National Center for Biotechnology Information: NCBI Insect Genomes Project
The Genome Sequencing Center at Washington University: Drosophila simulans Sequencing
Publication/s:
Clark AG *et. al.* , *Nature* , **450** , 203 (2007).

Drosophila sechellia Rob3c **(Dse)**

Taxonomy:
cellular organisms | Eukaryota | Opisthokonta | Metazoa | Eumetazoa | Bilateria | Coelomata | Protostomia | Panarthropoda | Arthropoda | Mandibulata | Pancrustacea | Hexapoda | Insecta | Dicondylia | Pterygota | Neoptera | Endopterygota | Diptera | Brachycera | Muscomorpha | Eremoneura | Cyclorrhapha | Schizophora | Acalyptratae | Ephydroidea | Drosophilidae | Drosophilinae | Drosophilini | Drosophilina | Drosophiliti | Drosophila | Sophophora | melanogaster group | melanogaster subgroup
Reference/s:
Broad Institute of Harvard and MIT: Other vertebrates and invertebrates
FlyBase: A Database of the Drosophila Genome
International Species Sequencing Consortium: Drosophila Sequencing Consortium
National Center for Biotechnology Information: NCBI Insect Genomes Project
UCSC Genome Bioinformatics: Drosophila sechellia Genome Browser Gateway
e! Ensembl: Drosophila sechellia
Publication/s:
Clark AG *et. al.* , *Nature* , **450** , 203 (2007).

Drosophila simulans str. Mosaic **(Dss)**
Taxonomy:
cellular organisms | Eukaryota | Opisthokonta | Metazoa | Eumetazoa | Bilateria | Coelomata | Protostomia | Panarthropoda | Arthropoda | Mandibulata | Pancrustacea | Hexapoda | Insecta | Dicondylia | Pterygota | Neoptera | Endopterygota | Diptera | Brachycera | Muscomorpha | Eremoneura | Cyclorrhapha | Schizophora | Acalyptratae | Ephydroidea | Drosophilidae | Drosophilinae | Drosophilini | Drosophilina | Drosophiliti | Drosophila | Sophophora | melanogaster group | melanogaster subgroup
Reference/s:
FlyBase: A Database of the Drosophila Genome
International Species Sequencing Consortium: Drosophila Sequencing Consortium
National Center for Biotechnology Information: NCBI Insect Genomes Project
The Genome Sequencing Center at Washington University: Drosophila simulans Sequencing
UCSC Genome Bioinformatics: Drosophila simulans Genome Browser Gateway
e! Ensembl: Drosophila simulans
Publication/s:
Clark AG *et. al.* , *Nature* , **450** , 203 (2007).

Drosophila simulans str. white501 **(Dss_a)**
Taxonomy:
cellular organisms | Eukaryota | Opisthokonta | Metazoa | Eumetazoa | Bilateria | Coelomata | Protostomia | Panarthropoda | Arthropoda | Mandibulata | Pancrustacea | Hexapoda | Insecta | Dicondylia | Pterygota | Neoptera | Endopterygota | Diptera | Brachycera | Muscomorpha | Eremoneura | Cyclorrhapha | Schizophora | Acalyptratae | Ephydroidea | Drosophilidae | Drosophilinae | Drosophilini | Drosophilina | Drosophiliti | Drosophila | Sophophora | melanogaster group | melanogaster subgroup
Reference/s:
International Species Sequencing Consortium: Drosophila Sequencing Consortium
National Center for Biotechnology Information: NCBI Insect Genomes Project
The Genome Sequencing Center at Washington University: Drosophila simulans Sequencing
Publication/s:
Clark AG *et. al.* , *Nature* , **450** , 203 (2007).

Drosophila erecta TSC#14021-0224.01 **(Der)**
Taxonomy:
cellular organisms | Eukaryota | Opisthokonta | Metazoa | Eumetazoa | Bilateria | Coelomata | Protostomia | Panarthropoda | Arthropoda | Mandibulata | Pancrustacea | Hexapoda | Insecta | Dicondylia | Pterygota | Neoptera | Endopterygota | Diptera | Brachycera | Muscomorpha | Eremoneura | Cyclorrhapha | Schizophora | Acalyptratae | Ephydroidea | Drosophilidae | Drosophilinae | Drosophilini | Drosophilina | Drosophiliti | Drosophila | Sophophora | melanogaster group | melanogaster subgroup
Reference/s:
FlyBase: A Database of the Drosophila Genome
International Species Sequencing Consortium: Drosophila Sequencing Consortium
National Center for Biotechnology Information: NCBI Insect Genomes Project
UCSC Genome Bioinformatics: Drosophila erecta Genome Browser Gateway
e! Ensembl: Drosophila erecta
Publication/s:
Clark AG *et. al.* , *Nature* , **450** , 203 (2007).

Drosophila pseudoobscura MV2-25 **(Dp)**
Taxonomy:
cellular organisms | Eukaryota | Opisthokonta | Metazoa | Eumetazoa | Bilateria | Coelomata | Protostomia | Panarthropoda | Arthropoda | Mandibulata | Pancrustacea | Hexapoda | Insecta | Dicondylia | Pterygota | Neoptera | Endopterygota | Diptera | Brachycera | Muscomorpha | Eremoneura | Cyclorrhapha | Schizophora | Acalyptratae | Ephydroidea | Drosophilidae | Drosophilinae | Drosophilini | Drosophilina | Drosophiliti | Drosophila | Sophophora | obscura group | pseudoobscura subgroup
Reference/s:
FlyBase: A Database of the Drosophila Genome
Human Genome Sequencing Center at Baylor College of Medicine: Drosophila pseudoobscura Genome Sequence
International Species Sequencing Consortium: Drosophila pseudoobscura Sequencing Consortium
National Center for Biotechnology Information: NCBI Insect Genomes Project
UCSC Genome Bioinformatics: Drosophila pseudoobscura Genome Browser Gateway
e! Ensembl: Drosophila pseudoobscura
Publication/s:
Richards S *et. al.* , *Genome Res* , **15** , 1 (2005).

Drosophila persimilis MSH-3 **(Drp)**
Taxonomy:
cellular organisms | Eukaryota | Opisthokonta | Metazoa | Eumetazoa | Bilateria | Coelomata | Protostomia | Panarthropoda | Arthropoda | Mandibulata | Pancrustacea | Hexapoda | Insecta | Dicondylia | Pterygota | Neoptera | Endopterygota | Diptera | Brachycera | Muscomorpha | Eremoneura | Cyclorrhapha | Schizophora | Acalyptratae | Ephydroidea | Drosophilidae | Drosophilinae | Drosophilini | Drosophilina | Drosophiliti | Drosophila | Sophophora | obscura group | pseudoobscura subgroup
Reference/s:
Broad Institute of Harvard and MIT: Other vertebrates and invertebrates
FlyBase: A Database of the Drosophila Genome
International Species Sequencing Consortium: Drosophila Sequencing Consortium

National Center for Biotechnology Information: NCBI Insect Genomes Project
UCSC Genome Bioinformatics: Drosophila persimilis Genome Browser Gateway
e! Ensembl: Drosophila persimilis
Publication/s:
Clark AG *et. al.* , *Nature* , **450** , 203 (2007).

Drosophila willistoni TSC#14030-0811.24 **(Dw)**
Taxonomy:
cellular organisms | Eukaryota | Opisthokonta | Metazoa | Eumetazoa | Bilateria | Coelomata | Protostomia | Panarthropoda | Arthropoda | Mandibulata | Pancrustacea | Hexapoda | Insecta | Dicondylia | Pterygota | Neoptera | Endopterygota | Diptera | Brachycera | Muscomorpha | Eremoneura | Cyclorrhapha | Schizophora | Acalyptratae | Ephydroidea | Drosophilidae | Drosophilinae | Drosophilini | Drosophilina | Drosophiliti | Drosophila | Sophophora | willistoni group | willistoni subgroup
Reference/s:
FlyBase: A Database of the Drosophila Genome
International Species Sequencing Consortium: Drosophila Sequencing Consortium
National Center for Biotechnology Information: NCBI Insect Genomes Project
e! Ensembl: Drosophila willistoni
Publication/s:
Clark AG *et. al.* , *Nature* , **450** , 203 (2007).

Bactrocera dorsalis **(Bcd)**
Taxonomy:
cellular organisms | Eukaryota | Opisthokonta | Metazoa | Eumetazoa | Bilateria | Coelomata | Protostomia | Panarthropoda | Arthropoda | Mandibulata | Pancrustacea | Hexapoda | Insecta | Dicondylia | Pterygota | Neoptera | Endopterygota | Diptera | Brachycera | Muscomorpha | Eremoneura | Cyclorrhapha | Schizophora | Acalyptratae | Tephritoidea | Tephritidae | Dacinae | Dacini | Bactrocera | Bactrocera | Bactrocera dorsalis species complex
Reference/s:
GenBank - NIH genetic sequence database: GenBank species TBLASTN

Glossina morsitans morsitans **(Gom)**
Taxonomy:
cellular organisms | Eukaryota | Opisthokonta | Metazoa | Eumetazoa | Bilateria | Coelomata | Protostomia | Panarthropoda | Arthropoda | Mandibulata | Pancrustacea | Hexapoda | Insecta | Dicondylia | Pterygota | Neoptera | Endopterygota | Diptera | Brachycera | Muscomorpha | Eremoneura | Cyclorrhapha | Schizophora | Calyptratae | Hippoboscoidea | Glossinidae | Glossina | Glossina | Glossina morsitans
Reference/s:
GenBank - NIH genetic sequence database: GenBank species TBLASTN
The Wellcome Trust Sanger Institute: Glossina Genome Project
VectorBase: Glossina morsitans morsitans@vectorbase.org
Publication/s:
Lehane MJ *et. al.* , *Genome Biol* , **4** , R63 (2003).

Mayetiola destructor **(Myd)**
Taxonomy:
cellular organisms | Eukaryota | Opisthokonta | Metazoa | Eumetazoa | Bilateria | Coelomata | Protostomia | Panarthropoda | Arthropoda | Mandibulata | Pancrustacea | Hexapoda | Insecta | Dicondylia | Pterygota | Neoptera | Endopterygota | Diptera | Nematocera | Bibionomorpha | Sciaroidea | Cecidomyiidae | Cecidomyiinae | Lasiopteridi | Oligotrophini | Mayetiola
Reference/s:
Agricultural Pest Genomics Resource Database: Hessian Fly Base
Human Genome Sequencing Center at Baylor College of Medicine: Hessian Fly Genome Project
National Center for Biotechnology Information: NCBI Insect Genomes Project

Rhynchosciara americana **(Rya)**
Taxonomy:
cellular organisms | Eukaryota | Opisthokonta | Metazoa | Eumetazoa | Bilateria | Coelomata | Protostomia | Panarthropoda | Arthropoda | Mandibulata | Pancrustacea | Hexapoda | Insecta | Dicondylia | Pterygota | Neoptera | Endopterygota | Diptera | Nematocera | Bibionomorpha | Sciaroidea | Sciaridae | Rhynchosciara

Culicoides sonorensis **(Ccs)**
Taxonomy:
cellular organisms | Eukaryota | Opisthokonta | Metazoa | Eumetazoa | Bilateria | Coelomata | Protostomia | Panarthropoda | Arthropoda | Mandibulata | Pancrustacea | Hexapoda | Insecta | Dicondylia | Pterygota | Neoptera | Endopterygota | Diptera | Nematocera | Culicimorpha | Chironomoidea | Ceratopogonidae | Ceratopogoninae | Culicoidini | Culicoides | Monoculicoides

Chironomus tentans **(Cht)**
Taxonomy:
cellular organisms | Eukaryota | Opisthokonta | Metazoa | Eumetazoa | Bilateria | Coelomata | Protostomia | Panarthropoda | Arthropoda | Mandibulata | Pancrustacea | Hexapoda | Insecta | Dicondylia | Pterygota | Neoptera | Endopterygota | Diptera | Nematocera | Culicimorpha | Chironomoidea | Chironomidae | Chironominae | Chironomini | Chironomus | Camptochironomus
Reference/s:
GenBank - NIH genetic sequence database: GenBank species TBLASTN
Publication/s:
Arvestad L *et. al.* , *Insect Mol Biol* , **14** , 689 (2005).

Anopheles gambiae str. PEST **(Ang)**
Taxonomy:
cellular organisms | Eukaryota | Opisthokonta | Metazoa | Eumetazoa | Bilateria | Coelomata | Protostomia | Panarthropoda | Arthropoda | Mandibulata | Pancrustacea | Hexapoda | Insecta | Dicondylia | Pterygota | Neoptera | Endopterygota | Diptera | Nematocera | Culicimorpha | Culicoidea | Culicidae | Anophelinae | Anopheles | Cellia | Pyretophorus | gambiae species complex | Anopheles gambiae
Reference/s:
AnoBase - The Anopheles Database: AnoBase - The Anopheles Database
Genoscope: Anopheles gambiae
International Species Sequencing Consortium: Anopheles gambiae Sequencing Consortium
National Center for Biotechnology Information: NCBI Insect Genomes Project

National Center for Biotechnology Information Reference Sequences: Anopheles gambiae (African malaria mosquito) genome view
The Gene Index Project: DFCI Mosquito Gene Index
UCSC Genome Bioinformatics: Anopheles gambiae Genome Browser Gateway
VectorBase: Anopheles gambiae@vectorbase.org
e! Ensembl: Mosquito
Publication/s:
Sharakhova MV *et. al.* , *Genome Biol* , **8** , R5 (2007).
Mongin E *et. al.* , *Trends Parasitol* , **20** , 49 (2004).
Holt RA *et. al.* , *Science* , **298** , 129 (2002).
Beard CB, Hamm DM, Collins FH , *Insect Mol Biol* , **2** , 103 (1993).

***Aedes albopictus* (Ada)**
Taxonomy:
cellular organisms | Eukaryota | Opisthokonta | Metazoa | Eumetazoa | Bilateria | Coelomata | Protostomia | Panarthropoda | Arthropoda | Mandibulata | Pancrustacea | Hexapoda | Insecta | Dicondylia | Pterygota | Neoptera | Endopterygota | Diptera | Nematocera | Culicimorpha | Culicoidea | Culicidae | Culicinae | Aedini | Aedes | Stegomyia

***Aedes aegypti str. Liverpool* (Aea)**
Taxonomy:
cellular organisms | Eukaryota | Opisthokonta | Metazoa | Eumetazoa | Bilateria | Coelomata | Protostomia | Panarthropoda | Arthropoda | Mandibulata | Pancrustacea | Hexapoda | Insecta | Dicondylia | Pterygota | Neoptera | Endopterygota | Diptera | Nematocera | Culicimorpha | Culicoidea | Culicidae | Culicinae | Aedini | Aedes | Stegomyia
Reference/s:
Broad Institute of Harvard and MIT: Aedes aegypti Database
GenBank - NIH genetic sequence database: GenBank species TBLASTN
International Species Sequencing Consortium: Aedes aegypti Sequencing Consortium
National Center for Biotechnology Information: NCBI Insect Genomes Project
The Gene Index Project: DFCI A. aegypti Gene Index
VectorBase: Aedes aegypti@vectorbase.org
e! Ensembl: A.aegypti
Publication/s:
Nene V *et. al.* , *Science* , **316** , 1718 (2007).
Bartholomay LC *et. al.* , *Infect Immun* , **72** , 4114 (2004).

***Armigeres subalbatus* (Ars)**
Taxonomy:
cellular organisms | Eukaryota | Opisthokonta | Metazoa | Eumetazoa | Bilateria | Coelomata | Protostomia | Panarthropoda | Arthropoda | Mandibulata | Pancrustacea | Hexapoda | Insecta | Dicondylia | Pterygota | Neoptera | Endopterygota | Diptera | Nematocera | Culicimorpha | Culicoidea | Culicidae | Culicinae | Aedini | Armigeres | Armigeres

***Culex pipiens quinquefasciatus str. JHB* (Cpq)**
Taxonomy:
cellular organisms | Eukaryota | Opisthokonta | Metazoa | Eumetazoa | Bilateria | Coelomata | Protostomia | Panarthropoda | Arthropoda | Mandibulata | Pancrustacea | Hexapoda | Insecta | Dicondylia | Pterygota | Neoptera | Endopterygota | Diptera | Nematocera | Culicimorpha | Culicoidea | Culicidae | Culicinae | Culicini | Culex | Culex | Culex pipiens complex
Reference/s:
Broad Institute of Harvard and MIT: Culex pipiens Database
International Species Sequencing Consortium: Culex pipiens Sequencing Consortium
National Center for Biotechnology Information: NCBI Insect Genomes Project
VectorBase: Culex pipiens quinquefasciatus@vectorbase.org
e! Ensembl: Culex quinquefasciatus (Culex quinquefasciatus)
Publication/s:
Arensburger P *et. al.* , *Science* , **330** , 86 (2010).

***Culex pipiens pipiens* (Cup)**
Taxonomy:
cellular organisms | Eukaryota | Opisthokonta | Metazoa | Eumetazoa | Bilateria | Coelomata | Protostomia | Panarthropoda | Arthropoda | Mandibulata | Pancrustacea | Hexapoda | Insecta | Dicondylia | Pterygota | Neoptera | Endopterygota | Diptera | Nematocera | Culicimorpha | Culicoidea | Culicidae | Culicinae | Culicini | Culex | Culex | Culex pipiens complex | Culex pipiens

***Lutzomyia longipalpis* (Ll)**
Taxonomy:
cellular organisms | Eukaryota | Opisthokonta | Metazoa | Eumetazoa | Bilateria | Coelomata | Protostomia | Panarthropoda | Arthropoda | Mandibulata | Pancrustacea | Hexapoda | Insecta | Dicondylia | Pterygota | Neoptera | Endopterygota | Diptera | Nematocera | Psychodomorpha | Psychodoidea | Psychodidae | Phlebotominae | Lutzomyia | Lutzomyia
Reference/s:
GenBank - NIH genetic sequence database: GenBank species TBLASTN
Human Genome Sequencing Center at Baylor College of Medicine: Lutzomyia longipalpis Genome Project
Publication/s:
Dillon RJ *et. al.* , *Genomics* , **88** , 831 (2006).

***Apis mellifera str. DH4* (Am)**
Taxonomy:
cellular organisms | Eukaryota | Opisthokonta | Metazoa | Eumetazoa | Bilateria | Coelomata | Protostomia | Panarthropoda | Arthropoda | Mandibulata | Pancrustacea | Hexapoda | Insecta | Dicondylia | Pterygota | Neoptera | Endopterygota | Hymenoptera | Apocrita | Aculeata | Apoidea | Apidae | Apinae | Apini | Apis
Reference/s:
BaNG Nematode and Neglected Genomics: HEXAPODA
GenBank - NIH genetic sequence database: GenBank species TBLASTN
Human Genome Sequencing Center at Baylor College of Medicine: Honey Bee Genome Project
Hymenoptera Genome Database: BeeBase
International Species Sequencing Consortium: Honeybee Genome Sequencing Consortium
National Center for Biotechnology Information: Honey bee Sequencing Project

National Center for Biotechnology Information Reference Sequences: Apis mellifera (honey bee) genome view
The Gene Index Project: DFCI Honeybee (Apis mellifera) Gene Index
UCSC Genome Bioinformatics: Apis mellifera Genome Browser Gateway
Publication/s:
Honeybee Genome Sequencing Consortium *et. al.* , *Nature* , **443** , 931 (2006).
Nunes FM *et. al.* , *BMC Genomics* , **5** , 84 (2004).
Whitfield CW *et. al.* , *Genome Res* , **12** , 555 (2002).

***Apis florea* (Apf)**
Taxonomy:
cellular organisms | Eukaryota | Opisthokonta | Metazoa | Eumetazoa | Bilateria | Coelomata | Protostomia | Panarthropoda | Arthropoda | Mandibulata | Pancrustacea | Hexapoda | Insecta | Dicondylia | Pterygota | Neoptera | Endopterygota | Hymenoptera | Apocrita | Aculeata | Apoidea | Apidae | Apinae | Apini | Apis
Reference/s:
Human Genome Sequencing Center at Baylor College of Medicine: Dwarf Honey Bee Genome Project
National Center for Biotechnology Information: NCBI Insect Genomes Project

***Bombus terrestris* (Bot)**
Taxonomy:
cellular organisms | Eukaryota | Opisthokonta | Metazoa | Eumetazoa | Bilateria | Coelomata | Protostomia | Panarthropoda | Arthropoda | Mandibulata | Pancrustacea | Hexapoda | Insecta | Dicondylia | Pterygota | Neoptera | Endopterygota | Hymenoptera | Apocrita | Aculeata | Apoidea | Apidae | Bombinae | Bombini | Bombus | Bombus
Reference/s:
Human Genome Sequencing Center at Baylor College of Medicine: Bumble Bee Genome Project
National Center for Biotechnology Information: NCBI Insect Genomes Project

***Bombus impatiens* (Boi)**
Taxonomy:
cellular organisms | Eukaryota | Opisthokonta | Metazoa | Eumetazoa | Bilateria | Coelomata | Protostomia | Panarthropoda | Arthropoda | Mandibulata | Pancrustacea | Hexapoda | Insecta | Dicondylia | Pterygota | Neoptera | Endopterygota | Hymenoptera | Apocrita | Aculeata | Apoidea | Apidae | Bombinae | Bombini | Bombus | Pyrobombus
Reference/s:
National Center for Biotechnology Information: NCBI Insect Genomes Project

***Megachile rotundata* (Mer)**
Taxonomy:
cellular organisms | Eukaryota | Opisthokonta | Metazoa | Eumetazoa | Bilateria | Coelomata | Protostomia | Panarthropoda | Arthropoda | Mandibulata | Pancrustacea | Hexapoda | Insecta | Dicondylia | Pterygota | Neoptera | Endopterygota | Hymenoptera | Apocrita | Aculeata | Apoidea | Megachilidae | Megachilinae | Megachilini | Megachile
Reference/s:
National Center for Biotechnology Information: NCBI Insect Genomes Project

***Linepithema humile* (Lh)**
Taxonomy:
cellular organisms | Eukaryota | Opisthokonta | Metazoa | Eumetazoa | Bilateria | Coelomata | Protostomia | Panarthropoda | Arthropoda | Mandibulata | Pancrustacea | Hexapoda | Insecta | Dicondylia | Pterygota | Neoptera | Endopterygota | Hymenoptera | Apocrita | Aculeata | Vespoidea | Formicidae | Dolichoderinae | Linepithema
Reference/s:
Hymenoptera Genome Database: ArgieBase
International Species Sequencing Consortium: Argentine ant Sequencing Consortium
National Center for Biotechnology Information: NCBI Insect Genomes Project
Publication/s:
Smith CD *et. al.* , *Proc Natl Acad Sci U S A* , **108** , 5673 (2011).

***Camponotus floridanus* (Cmf)**
Taxonomy:
cellular organisms | Eukaryota | Opisthokonta | Metazoa | Eumetazoa | Bilateria | Coelomata | Protostomia | Panarthropoda | Arthropoda | Mandibulata | Pancrustacea | Hexapoda | Insecta | Dicondylia | Pterygota | Neoptera | Endopterygota | Hymenoptera | Apocrita | Aculeata | Vespoidea | Formicidae | Formicinae | Camponotini | Camponotus
Reference/s:
Hymenoptera Genome Database: Florida Carpenter Ant Genome Project
National Center for Biotechnology Information: NCBI Insect Genomes Project
Publication/s:
Bonasio R *et. al.* , *Science* , **329** , 1068 (2010).

***Acromyrmex echinatior* (Ae)**
Taxonomy:
cellular organisms | Eukaryota | Opisthokonta | Metazoa | Eumetazoa | Bilateria | Coelomata | Protostomia | Panarthropoda | Arthropoda | Mandibulata | Pancrustacea | Hexapoda | Insecta | Dicondylia | Pterygota | Neoptera | Endopterygota | Hymenoptera | Apocrita | Aculeata | Vespoidea | Formicidae | Myrmicinae | Attini | Acromyrmex
Reference/s:
International Species Sequencing Consortium: Leafcutter ant Sequencing Consortium
National Center for Biotechnology Information: NCBI Insect Genomes Project
Publication/s:
Nygaard S *et. al.* , *Genome Res* , **21** , 1339 (2011).

***Atta cephalotes* (Aac)**
Taxonomy:
cellular organisms | Eukaryota | Opisthokonta | Metazoa | Eumetazoa | Bilateria | Coelomata | Protostomia | Panarthropoda | Arthropoda | Mandibulata | Pancrustacea | Hexapoda | Insecta | Dicondylia | Pterygota | Neoptera | Endopterygota | Hymenoptera | Apocrita | Aculeata | Vespoidea | Formicidae | Myrmicinae | Attini | Atta
Reference/s:
Hymenoptera Genome Database: AttaBase
International Species Sequencing Consortium: Atta cephalotes Sequencing Consortium
National Center for Biotechnology Information: NCBI Insect Genomes Project
Publication/s:
Suen G *et. al.* , *PLoS Genet* , **7** , e1002007 (2011).

***Pogonomyrmex barbatus* (Pob)**
Taxonomy:
cellular organisms | Eukaryota | Opisthokonta | Metazoa | Eumetazoa | Bilateria | Coelomata | Protostomia | Panarthropoda | Arthropoda | Mandibulata | Pancrustacea | Hexapoda | Insecta | Dicondylia | Pterygota | Neoptera | Endopterygota | Hymenoptera | Apocrita | Aculeata | Vespoidea | Formicidae | Myrmicinae | Myrmicini | Pogonomyrmex
Reference/s:
Hymenoptera Genome Database: PogoBase
International Species Sequencing Consortium: Red harvester ant Sequencing Consortium
National Center for Biotechnology Information: NCBI Insect Genomes Project
Publication/s:
Smith CR *et. al.* , *Proc Natl Acad Sci U S A* , **108** , 5667 (2011).

***Solenopsis invicta* (Soi)**
Taxonomy:
cellular organisms | Eukaryota | Opisthokonta | Metazoa | Eumetazoa | Bilateria | Coelomata | Protostomia | Panarthropoda | Arthropoda | Mandibulata | Pancrustacea | Hexapoda | Insecta | Dicondylia | Pterygota | Neoptera | Endopterygota | Hymenoptera | Apocrita | Aculeata | Vespoidea | Formicidae | Myrmicinae | Solenopsidini | Solenopsis
Reference/s:
Hymenoptera Genome Database: Fire Ant Genome Project
International Species Sequencing Consortium: Fire ant Sequencing Consortium
National Center for Biotechnology Information: NCBI Insect Genomes Project
Publication/s:
Wurm Y *et. al.* , *Proc Natl Acad Sci U S A* , **108** , 5679 (2011).

***Harpegnathos saltator* (Hrs)**
Taxonomy:
cellular organisms | Eukaryota | Opisthokonta | Metazoa | Eumetazoa | Bilateria | Coelomata | Protostomia | Panarthropoda | Arthropoda | Mandibulata | Pancrustacea | Hexapoda | Insecta | Dicondylia | Pterygota | Neoptera | Endopterygota | Hymenoptera | Apocrita | Aculeata | Vespoidea | Formicidae | Ponerinae | Ponerini | Harpegnathos
Reference/s:
Hymenoptera Genome Database: Jumping Ant Genome Project
National Center for Biotechnology Information: NCBI Insect Genomes Project
Publication/s:
Bonasio R *et. al.* , *Science* , **329** , 1068 (2010).

***Nasonia vitripennis str. SymAX* (Nav)**
Taxonomy:
cellular organisms | Eukaryota | Opisthokonta | Metazoa | Eumetazoa | Bilateria | Coelomata | Protostomia | Panarthropoda | Arthropoda | Mandibulata | Pancrustacea | Hexapoda | Insecta | Dicondylia | Pterygota | Neoptera | Endopterygota | Hymenoptera | Apocrita | Chaldicoidea group | Chalcidoidea | Pteromalidae | Pteromalinae | Nasonia
Reference/s:
Human Genome Sequencing Center at Baylor College of Medicine: Nasonia Genome Project
Hymenoptera Genome Database: NasoniaBase
International Species Sequencing Consortium: Nasonia Sequencing Consortium
National Center for Biotechnology Information: NCBI Insect Genomes Project
National Center for Biotechnology Information Reference Sequences: Wasp Genome Resources
University of Rochester: Nasonia Homepage
Publication/s:
Werren JH *et. al.* , *Science* , **327** , 343 (2010).

***Reticulitermes flavipes* (Ref)**
Taxonomy:
cellular organisms | Eukaryota | Opisthokonta | Metazoa | Eumetazoa | Bilateria | Coelomata | Protostomia | Panarthropoda | Arthropoda | Mandibulata | Pancrustacea | Hexapoda | Insecta | Dicondylia | Pterygota | Neoptera | Orthopteroidea | Dictyoptera | Isoptera | Rhinotermitidae | Heterotermitinae | Reticulitermes | Reticulitermes
Reference/s:
GenBank - NIH genetic sequence database: GenBank species TBLASTN
Publication/s:
Zhou X, Oi FM, Scharf ME , *Proc Natl Acad Sci U S A* , **103** , 4499 (2006).

***Sphodromantis centralis* (Spc)**
Taxonomy:
cellular organisms | Eukaryota | Opisthokonta | Metazoa | Eumetazoa | Bilateria | Coelomata | Protostomia | Panarthropoda | Arthropoda | Mandibulata | Pancrustacea | Hexapoda | Insecta | Dicondylia | Pterygota | Neoptera | Orthopteroidea | Dictyoptera | Mantodea | Mantidae | Mantinae | Paramantini | Sphodromantis
Reference/s:
BaNG Nematode and Neglected Genomics: HEXAPODA

***Locusta migratoria* (Lom)**
Taxonomy:
cellular organisms | Eukaryota | Opisthokonta | Metazoa | Eumetazoa | Bilateria | Coelomata | Protostomia | Panarthropoda | Arthropoda | Mandibulata | Pancrustacea | Hexapoda | Insecta | Dicondylia | Pterygota | Neoptera | Orthopteroidea | Orthoptera | Caelifera | Acridomorpha | Acridoidea | Acrididae | Oedipodinae | Locusta
Reference/s:
GenBank - NIH genetic sequence database: GenBank species TBLASTN
Publication/s:
Kang L *et. al.* , *Proc Natl Acad Sci U S A* , **101** , 17611 (2004).

***Gryllus pennsylvanicus* (Grp)**
Taxonomy:
cellular organisms | Eukaryota | Opisthokonta | Metazoa | Eumetazoa | Bilateria | Coelomata | Protostomia | Panarthropoda | Arthropoda | Mandibulata | Pancrustacea | Hexapoda | Insecta | Dicondylia | Pterygota | Neoptera | Orthopteroidea | Orthoptera | Ensifera | Grylloidea | Gryllidae | Gryllinae | Gryllus

***Gryllus bimaculatus* (Gb)**
Taxonomy:

cellular organisms | Eukaryota | Opisthokonta | Metazoa | Eumetazoa | Bilateria | Coelomata | Protostomia | Panarthropoda | Arthropoda | Mandibulata | Pancrustacea | Hexapoda | Insecta | Dicondylia | Pterygota | Neoptera | Orthopteroidea | Orthoptera | Ensifera | Grylloidea | Gryllidae | Gryllinae | Gryllus
Reference/s:
BaNG Nematode and Neglected Genomics: HEXAPODA

Laupala kohalensis **(Lk)**
Taxonomy:
cellular organisms | Eukaryota | Opisthokonta | Metazoa | Eumetazoa | Bilateria | Coelomata | Protostomia | Panarthropoda | Arthropoda | Mandibulata | Pancrustacea | Hexapoda | Insecta | Dicondylia | Pterygota | Neoptera | Orthopteroidea | Orthoptera | Ensifera | Grylloidea | Gryllidae | Trigonidiinae | Laupala

Homalodisca coagulata **(Hoc)**
Taxonomy:
cellular organisms | Eukaryota | Opisthokonta | Metazoa | Eumetazoa | Bilateria | Coelomata | Protostomia | Panarthropoda | Arthropoda | Mandibulata | Pancrustacea | Hexapoda | Insecta | Dicondylia | Pterygota | Neoptera | Paraneoptera | Hemiptera | Euhemiptera | Clypeorrhyncha | Membracoidea | Cicadellidae | Cicadellinae | unclassified Cicadellinae | Homalodisca

Oncometopia nigricans **(Onn)**
Taxonomy:
cellular organisms | Eukaryota | Opisthokonta | Metazoa | Eumetazoa | Bilateria | Coelomata | Protostomia | Panarthropoda | Arthropoda | Mandibulata | Pancrustacea | Hexapoda | Insecta | Dicondylia | Pterygota | Neoptera | Paraneoptera | Hemiptera | Euhemiptera | Clypeorrhyncha | Membracoidea | Cicadellidae | Tettigellinae | Oncometopia

Lygus lineolaris **(Lyl)**
Taxonomy:
cellular organisms | Eukaryota | Opisthokonta | Metazoa | Eumetazoa | Bilateria | Coelomata | Protostomia | Panarthropoda | Arthropoda | Mandibulata | Pancrustacea | Hexapoda | Insecta | Dicondylia | Pterygota | Neoptera | Paraneoptera | Hemiptera | Euhemiptera | Neohemiptera | Prosorrhyncha | Heteroptera | Euheteroptera | Neoheteroptera | Panheteroptera | Cimicomorpha | Cimicoidea | Miridae | Mirinae | Mirini | Lygus

Rhodnius prolixus **(Rhp)**
Taxonomy:
cellular organisms | Eukaryota | Opisthokonta | Metazoa | Eumetazoa | Bilateria | Coelomata | Protostomia | Panarthropoda | Arthropoda | Mandibulata | Pancrustacea | Hexapoda | Insecta | Dicondylia | Pterygota | Neoptera | Paraneoptera | Hemiptera | Euhemiptera | Neohemiptera | Prosorrhyncha | Heteroptera | Euheteroptera | Neoheteroptera | Panheteroptera | Cimicomorpha | Reduvioidea | Reduviidae | Triatominae | Rhodnius
Reference/s:
National Center for Biotechnology Information: NCBI Insect Genomes Project
The Genome Sequencing Center at Washington University: Rhodnius prolixus

Toxoptera citricida **(Txc)**
Taxonomy:
cellular organisms | Eukaryota | Opisthokonta | Metazoa | Eumetazoa | Bilateria | Coelomata | Protostomia | Panarthropoda | Arthropoda | Mandibulata | Pancrustacea | Hexapoda | Insecta | Dicondylia | Pterygota | Neoptera | Paraneoptera | Hemiptera | Sternorrhyncha | Aphidiformes | Aphidomorpha | Aphidoidea | Aphididae | Aphidinae | Aphidini | Toxoptera
Reference/s:
GenBank - NIH genetic sequence database: GenBank species TBLASTN
Publication/s:
Hunter WB *et. al.* , *J Insect Sci* , **3** , 1 (2003).

Acyrthosiphon pisum LSR1 **(Ayp)**
Taxonomy:
cellular organisms | Eukaryota | Opisthokonta | Metazoa | Eumetazoa | Bilateria | Coelomata | Protostomia | Panarthropoda | Arthropoda | Mandibulata | Pancrustacea | Hexapoda | Insecta | Dicondylia | Pterygota | Neoptera | Paraneoptera | Hemiptera | Sternorrhyncha | Aphidiformes | Aphidomorpha | Aphidoidea | Aphididae | Aphidinae | Macrosiphini | Acyrthosiphon
Reference/s:
BaNG Nematode and Neglected Genomics: HEXAPODA
GenBank - NIH genetic sequence database: GenBank species TBLASTN
Human Genome Sequencing Center at Baylor College of Medicine: Pea Aphid Genome Project
International Species Sequencing Consortium: Pea aphid Sequencing Consortium
National Center for Biotechnology Information: NCBI Insect Genomes Project
National Center for Biotechnology Information Reference Sequences: Acyrthosiphon pisum (pea aphid) genome view
e! Ensembl: Acyrthosiphon pisum (Acyrthosiphon pisum)
Publication/s:
International Aphid Genomics Consortium *et. al.* , *PLoS Biol* , **8** , e1000313 (2010).
Nakabachi A *et. al.* , *Proc Natl Acad Sci U S A* , **102** , 5477 (2005).

Myzus persicae **(Myp)**
Taxonomy:
cellular organisms | Eukaryota | Opisthokonta | Metazoa | Eumetazoa | Bilateria | Coelomata | Protostomia | Panarthropoda | Arthropoda | Mandibulata | Pancrustacea | Hexapoda | Insecta | Dicondylia | Pterygota | Neoptera | Paraneoptera | Hemiptera | Sternorrhyncha | Aphidiformes | Aphidomorpha | Aphidoidea | Aphididae | Aphidinae | Macrosiphini | Myzus

Maconellicoccus hirsutus **(Mah)**
Taxonomy:
cellular organisms | Eukaryota | Opisthokonta | Metazoa | Eumetazoa | Bilateria | Coelomata | Protostomia | Panarthropoda | Arthropoda | Mandibulata | Pancrustacea | Hexapoda | Insecta | Dicondylia | Pterygota | Neoptera | Paraneoptera | Hemiptera | Sternorrhyncha | Aphidiformes | Coccoidea | Pseudococcidae | Maconellicoccus

Pediculus humanus corporis str. USDA **(Pdc)**
Taxonomy:
cellular organisms | Eukaryota | Opisthokonta | Metazoa | Eumetazoa | Bilateria | Coelomata | Protostomia | Panarthropoda | Arthropoda | Mandibulata | Pancrustacea | Hexapoda | Insecta | Dicondylia | Pterygota | Neoptera | Paraneoptera | Phthiraptera | Anoplura | Pediculidae | Pediculus | Pediculus humanus
Reference/s:
International Species Sequencing Consortium: Human body louse Sequencing Consortium

National Center for Biotechnology Information: NCBI Insect Genomes Project
VectorBase: P. humanus@vectorbase.org
e! Ensembl: Pediculus humanus
Publication/s:
Kirkness EF *et. al.* , *Proc Natl Acad Sci U S A* , **107** , 12168 (2010).

Plectus acuminatus **(Pla)**
Taxonomy:
cellular organisms | Eukaryota | Opisthokonta | Metazoa | Eumetazoa | Bilateria | Pseudocoelomata | Nematoda | Chromadorea | Araeolaimida | Plectoidea | Plectidae | Plectus
Reference/s:
GenBank - NIH genetic sequence database: GenBank species TBLASTN
Publication/s:
Sturzenbaum SR, Arts MS, Kammenga JE , *Cell Stress Chaperones* , **10** , 79 (2005).

Ascaris suum **(Ass)**
Taxonomy:
cellular organisms | Eukaryota | Opisthokonta | Metazoa | Eumetazoa | Bilateria | Pseudocoelomata | Nematoda | Chromadorea | Ascaridida | Ascaridoidea | Ascarididae | Ascaris
Reference/s:
International Species Sequencing Consortium: Ascaris suum Sequencing Consortium
National Center for Biotechnology Information: NCBI Nematoda genomes
Nematode.net Genome Sequencing Center: Ascaris suum
The Wellcome Trust Sanger Institute: Ascaris suum
Publication/s:
Wang J *et. al.* , *Genome Res* , **21** , 1462 (2011).
Jex AR *et. al.* , *Nature* , **479** , 529 (2011).
Parkinson J *et. al.* , *Nat Genet* , **36** , 1259 (2004).

Pristionchus pacificus **(Psp)**
Taxonomy:
cellular organisms | Eukaryota | Opisthokonta | Metazoa | Eumetazoa | Bilateria | Pseudocoelomata | Nematoda | Chromadorea | Diplogasterida | Neodiplogasteridae | Pristionchus
Reference/s:
International Species Sequencing Consortium: Pristionchus pacificus Sequencing Consortium
Max-Planck-Institut fuer Entwicklungsbiologie: Pristionchus pacificus
National Center for Biotechnology Information: NCBI Nematoda genomes
Nematode.net Genome Sequencing Center: Pristionchus pacificus
The Genome Sequencing Center at Washington University: Pristionchus pacificus var. california
UCSC Genome Bioinformatics: P. pacificus (Pristionchus pacificus) Genome Browser Gateway
WormBase: WormBase
e! Ensembl: Pristionchus pacificus
Publication/s:
Dieterich C *et. al.* , *Nat Genet* , **40** , 1193 (2008).
Parkinson J *et. al.* , *Nat Genet* , **36** , 1259 (2004).

Panagrellus redivivus **(Par)**
Taxonomy:
cellular organisms | Eukaryota | Opisthokonta | Metazoa | Eumetazoa | Bilateria | Pseudocoelomata | Nematoda | Chromadorea | Rhabditida | Panagrolaimoidea | Panagrolaimidae | Panagrellus

Strongyloides stercoralis **(Sts)**
Taxonomy:
cellular organisms | Eukaryota | Opisthokonta | Metazoa | Eumetazoa | Bilateria | Pseudocoelomata | Nematoda | Chromadorea | Rhabditida | Panagrolaimoidea | Strongyloididae | Strongyloides
Reference/s:
Nematode.net Genome Sequencing Center: Strongyloides stercoralis
Publication/s:
Parkinson J *et. al.* , *Nat Genet* , **36** , 1259 (2004).

Strongyloides ratti **(Str)**
Taxonomy:
cellular organisms | Eukaryota | Opisthokonta | Metazoa | Eumetazoa | Bilateria | Pseudocoelomata | Nematoda | Chromadorea | Rhabditida | Panagrolaimoidea | Strongyloididae | Strongyloides
Reference/s:
GenBank - NIH genetic sequence database: GenBank species TBLASTN
National Center for Biotechnology Information: NCBI Nematoda genomes
Nematode.net Genome Sequencing Center: Strongyloides ratti
The Wellcome Trust Sanger Institute: Strongyloides Sequencing
Publication/s:
Thompson FJ *et. al.* , *Mol Biochem Parasitol* , **142** , 32 (2005).
Parkinson J *et. al.* , *Nat Genet* , **36** , 1259 (2004).

Heterorhabditis bacteriophora **(Hb)**
Taxonomy:
cellular organisms | Eukaryota | Opisthokonta | Metazoa | Eumetazoa | Bilateria | Pseudocoelomata | Nematoda | Chromadorea | Rhabditida | Rhabditoidea | Heterorhabditidae | Heterorhabditis
Reference/s:
National Center for Biotechnology Information: NCBI Nematoda genomes
Nematode.net Genome Sequencing Center: Heterorhabditis bacteriophora
The Genome Sequencing Center at Washington University: Heterorhabditis bacteriophora

***Caenorhabditis remanei EM464* (Car_b)**
Taxonomy:
cellular organisms | Eukaryota | Opisthokonta | Metazoa | Eumetazoa | Bilateria | Pseudocoelomata | Nematoda | Chromadorea | Rhabditida | Rhabditoidea | Rhabditidae | Peloderinae | Caenorhabditis

***Caenorhabditis brenneri* (Cab)**
Taxonomy:
cellular organisms | Eukaryota | Opisthokonta | Metazoa | Eumetazoa | Bilateria | Pseudocoelomata | Nematoda | Chromadorea | Rhabditida | Rhabditoidea | Rhabditidae | Peloderinae | Caenorhabditis
Reference/s:
National Center for Biotechnology Information: NCBI Nematoda genomes
Nematode.net Genome Sequencing Center: Caenorhabditis brenneri
The Genome Sequencing Center at Washington University: Caenorhabditis brenneri
UCSC Genome Bioinformatics: C. brenneri (Caenorhabditis brenneri) Genome Browser Gateway
WormBase: WormBase
e! Ensembl: Caenorhabditis brenneri (Caenorhabditis brenneri)

***Caenorhabditis elegans* (Ce)**
Taxonomy:
cellular organisms | Eukaryota | Opisthokonta | Metazoa | Eumetazoa | Bilateria | Pseudocoelomata | Nematoda | Chromadorea | Rhabditida | Rhabditoidea | Rhabditidae | Peloderinae | Caenorhabditis
Reference/s:
GenBank - NIH genetic sequence database: GenBank species TBLASTN
International Species Sequencing Consortium: Caenorhabditis elegans Sequencing Consortium
National Center for Biotechnology Information: NCBI Nematoda genomes
National Center for Biotechnology Information Reference Sequences: Caenorhabditis elegans (nematodes) genome view
Nematode.net Genome Sequencing Center: Caenorhabditis elegans
The Gene Index Project: DFCI Caenorhabditis elegans Gene Index
The Genome Sequencing Center at Washington University: Caenorhabditis elegans
The Wellcome Trust Sanger Institute: Caenorhabditis Genome Sequencing Projects
UCSC Genome Bioinformatics: Caenorhabditis elegans Genome Browser Gateway
WorfDB: The C. elegans ORFeome cloning project
WormBase: WormBase
e! Ensembl: C.elegans
Publication/s:
Li S *et. al.* , *Science* , **303** , 540 (2004).
Lamesch P *et. al.* , *Genome Res* , **14** , 2064 (2004).
Reboul J *et. al.* , *Nat Genet* , **34** , 35 (2003).
C. elegans Sequencing Consortium , *Science* , **282** , 2012 (1998).

***Caenorhabditis briggsae* (Cb)**
Taxonomy:
cellular organisms | Eukaryota | Opisthokonta | Metazoa | Eumetazoa | Bilateria | Pseudocoelomata | Nematoda | Chromadorea | Rhabditida | Rhabditoidea | Rhabditidae | Peloderinae | Caenorhabditis
Reference/s:
International Species Sequencing Consortium: Caenorhabditis briggsae Sequencing Consortium
National Center for Biotechnology Information: NCBI Nematoda genomes
Nematode.net Genome Sequencing Center: Caenorhabditis briggsae
The Genome Sequencing Center at Washington University: Caenorhabditis briggsae
The Wellcome Trust Sanger Institute: The Caenorhabditis briggsae Genome Project
UCSC Genome Bioinformatics: Caenorhabditis briggsae Genome Browser Gateway
WormBase: WormBase
e! Ensembl: Caenorhabditis briggsae (Caenorhabditis briggsae)
Publication/s:
Stein LD *et. al.* , *PLoS Biol* , **1** , E45 (2003).

***Caenorhabditis japonica DF5081* (Cej)**
Taxonomy:
cellular organisms | Eukaryota | Opisthokonta | Metazoa | Eumetazoa | Bilateria | Pseudocoelomata | Nematoda | Chromadorea | Rhabditida | Rhabditoidea | Rhabditidae | Peloderinae | Caenorhabditis
Reference/s:
National Center for Biotechnology Information: NCBI Nematoda genomes
Nematode.net Genome Sequencing Center: Caenorhabditis japonica
The Genome Sequencing Center at Washington University: Caenorhabditis japonica
UCSC Genome Bioinformatics: C. japonica (Caenorhabditis japonica) Genome Browser Gateway
WormBase: WormBase
e! Ensembl: Caenorhabditis japonica (Caenorhabditis japonica)

***Caenorhabditis remanei PB4641* (Car)**
Taxonomy:
cellular organisms | Eukaryota | Opisthokonta | Metazoa | Eumetazoa | Bilateria | Pseudocoelomata | Nematoda | Chromadorea | Rhabditida | Rhabditoidea | Rhabditidae | Peloderinae | Caenorhabditis
Reference/s:
National Center for Biotechnology Information: NCBI Nematoda genomes
Nematode.net Genome Sequencing Center: Caenorhabditis remanei
The Genome Sequencing Center at Washington University: Caenorhabditis remanei
UCSC Genome Bioinformatics: C. remanei (Caenorhabditis remanei) Genome Browser Gateway
WormBase: WormBase
e! Ensembl: Caenorhabditis remanei (Caenorhabditis remanei)

***Ancylostoma caninum* (Acc)**
Taxonomy:
cellular organisms | Eukaryota | Opisthokonta | Metazoa | Eumetazoa | Bilateria | Pseudocoelomata | Nematoda | Chromadorea | Rhabditida | Strongylida | Ancylostomatoidea | Ancylostomatidae | Ancylostomatinae | Ancylostoma
Reference/s:
Nematode.net Genome Sequencing Center: Ancylostoma caninum
Publication/s:
Parkinson J *et. al.* , *Nat Genet* , **36** , 1259 (2004).

***Cooperia oncophora* (Coo)**
Taxonomy:
cellular organisms | Eukaryota | Opisthokonta | Metazoa | Eumetazoa | Bilateria | Pseudocoelomata | Nematoda | Chromadorea | Rhabditida | Strongylida | Trichostrongyloidea | Cooperiidae | Cooperia

***Haemonchus contortus* (Hc)**
Taxonomy:
cellular organisms | Eukaryota | Opisthokonta | Metazoa | Eumetazoa | Bilateria | Pseudocoelomata | Nematoda | Chromadorea | Rhabditida | Strongylida | Trichostrongyloidea | Haemonchidae | Haemonchinae | Haemonchus
Reference/s:
Nematode.net Genome Sequencing Center: Haemonchus contortus
The Wellcome Trust Sanger Institute: Haemonchus contortus Sequencing
WormBase: WormBase
Publication/s:
Parkinson J *et. al.* , *Nat Genet* , **36** , 1259 (2004).

***Nippostrongylus brasiliensis* (Nib)**
Taxonomy:
cellular organisms | Eukaryota | Opisthokonta | Metazoa | Eumetazoa | Bilateria | Pseudocoelomata | Nematoda | Chromadorea | Rhabditida | Strongylida | Trichostrongyloidea | Heligmonellidae | Nippostrongylinae | Nippostrongylus
Reference/s:
Nematode.net Genome Sequencing Center: Nippostrongylus brasiliensis
The Wellcome Trust Sanger Institute: Nippostrongylus brasiliensis

***Brugia malayi* (Brm)**
Taxonomy:
cellular organisms | Eukaryota | Opisthokonta | Metazoa | Eumetazoa | Bilateria | Pseudocoelomata | Nematoda | Chromadorea | Spirurida | Filarioidea | Onchocercidae | Brugia
Reference/s:
International Species Sequencing Consortium: Brugia malayi Sequencing Consortium
National Center for Biotechnology Information: NCBI Nematoda genomes
Nematode.net Genome Sequencing Center: Brugia malayi
The Gene Index Project: DFCI Brugia malayi Gene Index
The Institute for Genomic Research: Brugia malayi Genome Project
WormBase: WormBase
Publication/s:
Ghedin E *et. al.* , *Science* , **317** , 1756 (2007).
Parkinson J *et. al.* , *Nat Genet* , **36** , 1259 (2004).

***Litomosoides sigmodontis* (Lts)**
Taxonomy:
cellular organisms | Eukaryota | Opisthokonta | Metazoa | Eumetazoa | Bilateria | Pseudocoelomata | Nematoda | Chromadorea | Spirurida | Filarioidea | Onchocercidae | Litomosoides
Reference/s:
Nematode Genomes: Litomosoides sigmodontis lab strain established from Cameroon by Odile Bain
Nematode.net Genome Sequencing Center: Litomosoides sigmodontis

***Loa loa* (Lol)**
Taxonomy:
cellular organisms | Eukaryota | Opisthokonta | Metazoa | Eumetazoa | Bilateria | Pseudocoelomata | Nematoda | Chromadorea | Spirurida | Filarioidea | Onchocercidae | Loa
Reference/s:
Broad Institute of Harvard and MIT: Filarial worms Database
National Center for Biotechnology Information: NCBI Nematoda genomes

***Onchocerca volvulus* (Ov)**
Taxonomy:
cellular organisms | Eukaryota | Opisthokonta | Metazoa | Eumetazoa | Bilateria | Pseudocoelomata | Nematoda | Chromadorea | Spirurida | Filarioidea | Onchocercidae | Onchocerca
Reference/s:
Broad Institute of Harvard and MIT: Filarial worms Database
GenBank - NIH genetic sequence database: GenBank species TBLASTN
National Center for Biotechnology Information: NCBI Nematoda genomes
Nematode.net Genome Sequencing Center: Onchocerca volvulus & Onchocerca ochengi
The Gene Index Project: DFCI Onchocerca volvulus Gene Index
The Wellcome Trust Sanger Institute: Onchocerca volvulus
Publication/s:
Parkinson J *et. al.* , *Nat Genet* , **36** , 1259 (2004).
Lizotte-Waniewski M *et. al.* , *Infect Immun* , **68** , 3491 (2000).

***Wuchereria bancrofti* (Wb)**
Taxonomy:

cellular organisms | Eukaryota | Opisthokonta | Metazoa | Eumetazoa | Bilateria | Pseudocoelomata | Nematoda | Chromadorea | Spirurida | Filarioidea | Onchocercidae | Wuchereria
Reference/s:
Broad Institute of Harvard and MIT: Filarial worms Database
National Center for Biotechnology Information: NCBI Nematoda genomes
Nematode.net Genome Sequencing Center: Wuchereria bancrofti

***Globodera rostochiensis* (Gr)**
Taxonomy:
cellular organisms | Eukaryota | Opisthokonta | Metazoa | Eumetazoa | Bilateria | Pseudocoelomata | Nematoda | Chromadorea | Tylenchida | Tylenchina | Tylenchoidea | Heteroderidae | Heteroderinae | Globodera
Reference/s:
Nematode.net Genome Sequencing Center: Globodera rostochiensis
Publication/s:
Parkinson J *et. al.* , *Nat Genet* , **36** , 1259 (2004).

***Heterodera glycines* (Heg)**
Taxonomy:
cellular organisms | Eukaryota | Opisthokonta | Metazoa | Eumetazoa | Bilateria | Pseudocoelomata | Nematoda | Chromadorea | Tylenchida | Tylenchina | Tylenchoidea | Heteroderidae | Heteroderinae | Heterodera
Reference/s:
International Species Sequencing Consortium: Heterodera Sequencing Consortium
National Center for Biotechnology Information: NCBI Nematoda genomes
Nematode.net Genome Sequencing Center: Heterodera glycines
Publication/s:
Bekal S *et. al.* , *Mol Genet Genomics* , **279** , 535 (2008).
Parkinson J *et. al.* , *Nat Genet* , **36** , 1259 (2004).

***Meloidogyne hapla* (Mh)**
Taxonomy:
cellular organisms | Eukaryota | Opisthokonta | Metazoa | Eumetazoa | Bilateria | Pseudocoelomata | Nematoda | Chromadorea | Tylenchida | Tylenchina | Tylenchoidea | Meloidogynidae | Meloidogyninae | Meloidogyne
Reference/s:
International Species Sequencing Consortium: Meloidogyne Sequencing Consortium
National Center for Biotechnology Information: NCBI Nematoda genomes
Nematode.net Genome Sequencing Center: Meloidogyne hapla
WormBase: WormBase
Publication/s:
Opperman CH *et. al.* , *Proc Natl Acad Sci U S A* , **105** , 14802 (2008).
Parkinson J *et. al.* , *Nat Genet* , **36** , 1259 (2004).

***Meloidogyne incognita* (Mi)**
Taxonomy:
cellular organisms | Eukaryota | Opisthokonta | Metazoa | Eumetazoa | Bilateria | Pseudocoelomata | Nematoda | Chromadorea | Tylenchida | Tylenchina | Tylenchoidea | Meloidogynidae | Meloidogyninae | Meloidogyne | Meloidogyne incognita group
Reference/s:
International Species Sequencing Consortium: Meloidogyne incognita Sequencing Consortium
National Center for Biotechnology Information: NCBI Nematoda genomes
Nematode.net Genome Sequencing Center: Meloidogyne incognita
Publication/s:
Abad P *et. al.* , *Nat Biotechnol* , **26** , 909 (2008).
Parkinson J *et. al.* , *Nat Genet* , **36** , 1259 (2004).

***Pratylenchus vulnus* (Prv)**
Taxonomy:
cellular organisms | Eukaryota | Opisthokonta | Metazoa | Eumetazoa | Bilateria | Pseudocoelomata | Nematoda | Chromadorea | Tylenchida | Tylenchina | Tylenchoidea | Pratylenchidae | Pratylenchinae | Pratylenchus
Reference/s:
Nematode.net Genome Sequencing Center: Pratylenchus vulnus

***Xiphinema index* (Xi)**
Taxonomy:
cellular organisms | Eukaryota | Opisthokonta | Metazoa | Eumetazoa | Bilateria | Pseudocoelomata | Nematoda | Enoplea | Enoplia | Dorylaimida | Dorylaimina | Longidoroidea | Longidoridae | Xiphinema
Reference/s:
Nematode.net Genome Sequencing Center: Xiphinema index

***Trichinella spiralis* (Trs)**
Taxonomy:
cellular organisms | Eukaryota | Opisthokonta | Metazoa | Eumetazoa | Bilateria | Pseudocoelomata | Nematoda | Enoplea | Enoplia | Trichocephalida | Trichinellidae | Trichinella
Reference/s:
International Species Sequencing Consortium: Trichinella Sequencing Consortium
National Center for Biotechnology Information: NCBI Nematoda genomes
Nematode.net Genome Sequencing Center: Trichinella spiralis
The Genome Sequencing Center at Washington University: Trichinella spiralis
Publication/s:
Mitreva M *et. al.* , *Nat Genet* , **43** , 228 (2011).
Parkinson J *et. al.* , *Nat Genet* , **36** , 1259 (2004).

***Trichuris muris* (Trm)**
Taxonomy:
cellular organisms | Eukaryota | Opisthokonta | Metazoa | Eumetazoa | Bilateria | Pseudocoelomata | Nematoda | Enoplea | Enoplia | Trichocephalida | Trichuridae | Trichuris
Reference/s:
Nematode.net Genome Sequencing Center: Trichuris muris
The Wellcome Trust Sanger Institute: T. muris Project
Publication/s:
Parkinson J *et. al.* , *Nat Genet* , **36** , 1259 (2004).

***Philodina roseola* (Pir)**
Taxonomy:
cellular organisms | Eukaryota | Opisthokonta | Metazoa | Eumetazoa | Bilateria | Pseudocoelomata | Rotifera | Bdelloidea | Philodinida | Philodinidae | Philodina

***Brachionus plicatilis* (Brp)**
Taxonomy:
cellular organisms | Eukaryota | Opisthokonta | Metazoa | Eumetazoa | Bilateria | Pseudocoelomata | Rotifera | Monogononta | Ploimida | Brachionidae | Brachionus

***Nematostella vectensis CH2 x CH6* (Nv)**
Taxonomy:
cellular organisms | Eukaryota | Opisthokonta | Metazoa | Eumetazoa | Cnidaria | Anthozoa | Hexacorallia | Actiniaria | Edwardsiidae | Nematostella
Reference/s:
DOE Joint Genome Institute: Nematostella vectensis
GenBank - NIH genetic sequence database: GenBank species TBLASTN
GenBank - NIH genetic sequence database: GenBank species TBLASTN WGS
International Species Sequencing Consortium: Nematostella vectensis Sequencing Consortium
StellaBase: Nematostella vectensis genomic database
Publication/s:
Putnam NH *et. al.* , *Science* , **317** , 86 (2007).
Technau U *et. al.* , *Trends Genet* , **21** , 633 (2005).

***Acropora palmata* (Arp)**
Taxonomy:
cellular organisms | Eukaryota | Opisthokonta | Metazoa | Eumetazoa | Cnidaria | Anthozoa | Hexacorallia | Scleractinia | Astrocoeniina | Acroporidae | Acropora

***Acropora millepora* (Acm)**
Taxonomy:
cellular organisms | Eukaryota | Opisthokonta | Metazoa | Eumetazoa | Cnidaria | Anthozoa | Hexacorallia | Scleractinia | Astrocoeniina | Acroporidae | Acropora
Reference/s:
GenBank - NIH genetic sequence database: GenBank species TBLASTN
Publication/s:
Technau U *et. al.* , *Trends Genet* , **21** , 633 (2005).

***Hydra magnipapillata* (Hm)**
Taxonomy:
cellular organisms | Eukaryota | Opisthokonta | Metazoa | Eumetazoa | Cnidaria | Hydrozoa | Hydroida | Anthomedusae | Hydridae | Hydra
Reference/s:
DOE Joint Genome Institute:
GenBank - NIH genetic sequence database: GenBank species TBLASTN WGS
International Species Sequencing Consortium: Hydra Sequencing Consortium
Publication/s:
Chapman JA *et. al.* , *Nature* , **464** , 592 (2010).

***Mnemiopsis leidyi* (Mnl)**
Taxonomy:
cellular organisms | Eukaryota | Opisthokonta | Metazoa | Eumetazoa | Ctenophora | Cyclocoela | Lobata | Bolinopsidae | Mnemiopsis
Reference/s:
GenBank - NIH genetic sequence database: GenBank species TBLASTN WGS

***Trichoplax adhaerens Grell-BS-1999* (Tia)**
Taxonomy:
cellular organisms | Eukaryota | Opisthokonta | Metazoa | Placozoa | Trichoplax
Reference/s:
DOE Joint Genome Institute: Trichoplax adhaerens
GenBank - NIH genetic sequence database: GenBank species TBLASTN WGS
International Species Sequencing Consortium: Trichoplax adhaerens Sequencing Consortium
Publication/s:
Srivastava M *et. al.* , *Nature* , **454** , 955 (2008).

***Amphimedon queenslandica* (Amq)**
Taxonomy:
cellular organisms | Eukaryota | Opisthokonta | Metazoa | Porifera | Demospongiae | Ceractinomorpha | Haplosclerida | Niphatidae | Amphimedon
Reference/s:
DOE Joint Genome Institute:
GenBank - NIH genetic sequence database: Amphimedon queenslandica
International Species Sequencing Consortium: Amphimedon queenslandica Sequencing Consortium
Publication/s:
Srivastava M *et. al.* , *Nature* , **466** , 720 (2010).

***Oscarella carmela* (Osc)**
Taxonomy:

cellular organisms | Eukaryota | Opisthokonta | Metazoa | Porifera | Demospongiae | Homoscleromorpha | Homosclerophorida | Plakinidae | Oscarella
Reference/s:
GenBank - NIH genetic sequence database: GenBank species TBLASTN
Publication/s:
Nichols SA *et. al.* , *Proc Natl Acad Sci U S A* , **103** , 12451 (2006).

Capsaspora owczarzaki ATCC 30864 **(Co)**
Taxonomy:
cellular organisms | Eukaryota | Opisthokonta | Opisthokonta incertae sedis | Ichthyosporea | Capsaspora | Capsaspora owczarzaki
Reference/s:
Broad Institute of Harvard and MIT: Origins of Multicellularity Database
National Center for Biotechnology Information: NCBI Protozoa genomes
TBestDB - Taxonomically Broad EST Database: Capsaspora owczarzaki

Sphaeroforma arctica **(Spa)**
Taxonomy:
cellular organisms | Eukaryota | Opisthokonta | Opisthokonta incertae sedis | Ichthyosporea | Ichthyophonida | Sphaeroforma
Reference/s:
Broad Institute of Harvard and MIT: Origins of Multicellularity Database
GenBank - NIH genetic sequence database: GenBank species TBLASTN WGS
TBestDB - Taxonomically Broad EST Database: Sphaeroforma arctica

Trichomonas vaginalis G3 **(Tv_a)**
Taxonomy:
cellular organisms | Eukaryota | Parabasalia | Trichomonadida | Trichomonadidae | Trichomonas | Trichomonas vaginalis
Reference/s:
National Center for Biotechnology Information: NCBI Protozoa genomes
The Institute for Genomic Research: Trichomonas vaginalis Genome Project
The Institute for Genomic Research: The Trichomonas vaginalis Genome Sequencing Project
TrichDB: TrichDB
Publication/s:
Carlton JM *et. al.* , *Science* , **315** , 207 (2007).

Bigelowiella natans **(Bin)**
Taxonomy:
cellular organisms | Eukaryota | Rhizaria | Cercozoa | Chlorarachniophyceae | Bigelowiella
Reference/s:
DOE Joint Genome Institute: Bigelowiella natans CCMP2755 v1.0
TBestDB - Taxonomically Broad EST Database: Bigelowiella natans

Cyanidioschyzon merolae 10D **(Cm)**
Taxonomy:
cellular organisms | Eukaryota | Rhodophyta | Bangiophyceae | Cyanidiales | Cyanidiaceae | Cyanidioschyzon
Reference/s:
Cyanidioschyzon merolae Genome Project: Cyanidioschyzon merolae Genome Project
GenBank - NIH genetic sequence database: GenBank species TBLASTN WGS
International Species Sequencing Consortium: Cyanidioschyzon merolae Sequencing Consortium
Publication/s:
Nozaki H *et. al.* , *BMC Biol* , **5** , 28 (2007).
Matsuzaki M *et. al.* , *Nature* , **428** , 653 (2004).
Ohta N *et. al.* , *DNA Res* , **10** , 67 (2003).
Ohta N, Sato N, Kuroiwa T , *Nucleic Acids Res* , **26** , 5190 (1998).

Volvox carteri f. nagariensis **(Vc)**
Taxonomy:
cellular organisms | Eukaryota | Viridiplantae | Chlorophyta | Chlorophyceae | Chlamydomonadales | Volvocaceae | Volvox | Volvox carteri
Reference/s:
DOE Joint Genome Institute: Volvox carteri Genome Sequencing
GenBank - NIH genetic sequence database: GenBank species TBLASTN WGS
International Species Sequencing Consortium: Volvox carteri Sequencing Consortium
Phytozome: Volvox carteri
Publication/s:
Prochnik SE *et. al.* , *Science* , **329** , 223 (2010).

Micromonas pusilla CCMP1545 **(Mip_a)**
Taxonomy:
cellular organisms | Eukaryota | Viridiplantae | Chlorophyta | Mamiellophyceae | Mamiellales | Micromonas | Micromonas pusilla
Reference/s:
DOE Joint Genome Institute: Micromonas pusilla CCMP1545
GenBank - NIH genetic sequence database: GenBank species TBLASTN WGS
International Species Sequencing Consortium: Micromonas Sequencing Consortium
Publication/s:
Worden AZ *et. al.* , *Science* , **324** , 268 (2009).

Ostreococcus lucimarinus **(Osl)**
Taxonomy:
cellular organisms | Eukaryota | Viridiplantae | Chlorophyta | Mamiellophyceae | Mamiellales | Ostreococcus
Reference/s:
DOE Joint Genome Institute: Ostreococcus lucimarinus
International Species Sequencing Consortium: Ostreococcus lucimarinus Sequencing Consortium

Publication/s:
Palenik B *et. al.* , *Proc Natl Acad Sci U S A* , **104** , 7705 (2007).

***Chlorella NC64A* (Chl)**
Taxonomy:
cellular organisms | Eukaryota | Viridiplantae | Chlorophyta | Trebouxiophyceae | Prasiolales | Prasiolales incertae sedis
Reference/s:
DOE Joint Genome Institute: Chlorella NC64A
International Species Sequencing Consortium: Chlorella Sequencing Consortium
Publication/s:
Blanc G *et. al.* , *Plant Cell* , **22** , 2943 (2010).

***Physcomitrella patens* (Php)**
Taxonomy:
cellular organisms | Eukaryota | Viridiplantae | Streptophyta | Streptophytina | Embryophyta | Bryophyta | Bryophytina | Bryopsida | Funariidae | Funariales | Funariaceae | Physcomitrella
Reference/s:
DOE Joint Genome Institute: Physcomitrella patens ssp patens ecotype Gransden 2004
GenBank - NIH genetic sequence database: GenBank species TBLASTN WGS
International Species Sequencing Consortium: Physcomitrella patens Sequencing Consortium
PHYSCObase: Physcomitrella patens
Physcomitrella patens resource: Physcomitrella patens
Phytozome: Physcomitrella patens (Moss)
e! Ensembl: Physcomitrella patens
Publication/s:
Rensing SA *et. al.* , *Science* , **319** , 64 (2008).

***Oryza sativa (indica cultivar-group)* (Os_b)**
Taxonomy:
cellular organisms | Eukaryota | Viridiplantae | Streptophyta | Streptophytina | Embryophyta | Tracheophyta | Euphyllophyta | Spermatophyta | Magnoliophyta | Liliopsida | commelinids | Poales | Poaceae | BEP clade | Ehrhartoideae | Oryzeae | Oryza
Reference/s:
Beijing Genomics Institute: Rice Information System
GenBank - NIH genetic sequence database: GenBank species TBLASTN
GenBank - NIH genetic sequence database: GenBank species TBLASTN WGS
International Species Sequencing Consortium: Oryza sativa (indica cultivar-group) Sequencing Consortium
The Gene Index Project: DFCI Rice (Oryza sativa) Gene Index
e! Ensembl: Oryza sativa indica group (Oryza indica)
Publication/s:
Xie K *et. al.* , *Sci China C Life Sci* , **48** , 445 (2005).
Zhang J *et. al.* , *Plant J* , **42** , 772 (2005).
Yu J *et. al.* , *PLoS Biol* , **3** , e38 (2005).
Matsumoto T *et. al.* , *Nature* , **436** , 793 (2005).
Feng Q *et. al.* , *Nature* , **420** , 316 (2002).
Yu J *et. al.* , *Science* , **296** , 79 (2002).

***Oryza sativa (japonica cultivar-group)* (Os_a)**
Taxonomy:
cellular organisms | Eukaryota | Viridiplantae | Streptophyta | Streptophytina | Embryophyta | Tracheophyta | Euphyllophyta | Spermatophyta | Magnoliophyta | Liliopsida | commelinids | Poales | Poaceae | BEP clade | Ehrhartoideae | Oryzeae | Oryza
Reference/s:
Beijing Genomics Institute: Rice Information System
GenBank - NIH genetic sequence database: GenBank species TBLASTN
GenBank - NIH genetic sequence database: GenBank species TBLASTN WGS
International Species Sequencing Consortium: Oryza sativa (japonica cultivar-group) Sequencing Consortium
Phytozome: Oryza sativa (Rice)
The Gene Index Project: DFCI Rice (Oryza sativa) Gene Index
e! Ensembl: Oryza sativa
Publication/s:
Yamamoto T *et. al.* , *BMC Genomics* , **11** , 267 (2010).
Yu J *et. al.* , *PLoS Biol* , **3** , e38 (2005).
Jantasuriyarat C *et. al.* , *Plant Physiol* , **138** , 105 (2005).
Matsumoto T *et. al.* , *Nature* , **436** , 793 (2005).
Kikuchi S *et. al.* , *Science* , **301** , 376 (2003).
Sasaki T *et. al.* , *Nature* , **420** , 312 (2002).
Goff SA *et. al.* , *Science* , **296** , 92 (2002).

***Oryza barthii* (Ob)**
Taxonomy:
cellular organisms | Eukaryota | Viridiplantae | Streptophyta | Streptophytina | Embryophyta | Tracheophyta | Euphyllophyta | Spermatophyta | Magnoliophyta | Liliopsida | commelinids | Poales | Poaceae | BEP clade | Ehrhartoideae | Oryzeae | Oryza
Reference/s:
GenBank - NIH genetic sequence database: GenBank species TBLASTN WGS

***Triticum aestivum* (Ta)**
Taxonomy:
cellular organisms | Eukaryota | Viridiplantae | Streptophyta | Streptophytina | Embryophyta | Tracheophyta | Euphyllophyta | Spermatophyta | Magnoliophyta | Liliopsida | commelinids | Poales | Poaceae | BEP clade | Pooideae | Triticeae | Triticum
Reference/s:

GenBank - NIH genetic sequence database: GenBank species TBLASTN WGS
The Gene Index Project: DFCI Wheat (Triticum aestivum) Gene Index

***Sorghum bicolor* (Sob)**
Taxonomy:
cellular organisms | Eukaryota | Viridiplantae | Streptophyta | Streptophytina | Embryophyta | Tracheophyta | Euphyllophyta | Spermatophyta | Magnoliophyta | Liliopsida | commelinids | Poales | Poaceae | PACMAD clade | Panicoideae | Andropogoneae | Sorghum
Reference/s:
DOE Joint Genome Institute: Sorghum genome project
GenBank - NIH genetic sequence database: GenBank species TBLASTN WGS
International Species Sequencing Consortium: Sorghum bicolor Sequencing Consortium
Phytozome: Sorghum bicolor (Cereal grass)
The Gene Index Project: DFCI Sorghum bicolor Gene Index
e! Ensembl: Sorghum bicolor
Publication/s:
Paterson AH *et. al.* , *Nature* , **457** , 551 (2009).

***Zea mays B73* (Zm)**
Taxonomy:
cellular organisms | Eukaryota | Viridiplantae | Streptophyta | Streptophytina | Embryophyta | Tracheophyta | Euphyllophyta | Spermatophyta | Magnoliophyta | Liliopsida | commelinids | Poales | Poaceae | PACMAD clade | Panicoideae | Andropogoneae | Zea
Reference/s:
GenBank - NIH genetic sequence database: GenBank species TBLASTN
International Species Sequencing Consortium: Zea mays Sequencing Consortium
MaizeSequence: MaizeSequence
National Center for Biotechnology Information Reference Sequences: Zea mays (maize) genome view
Phytozome: Zea mays (Corn)
The Gene Index Project: DFCI Maize (Zea mays) Gene Index
e! Ensembl: Zea mays
Publication/s:
Schnable PS *et. al.* , *Science* , **326** , 1112 (2009).
Verza NC *et. al.* , *Plant Mol Biol* , **59** , 363 (2005).
Lai J *et. al.* , *Genome Res* , **14** , 1932 (2004).

***Lycopersicon esculentum* (Le)**
Taxonomy:
cellular organisms | Eukaryota | Viridiplantae | Streptophyta | Streptophytina | Embryophyta | Tracheophyta | Euphyllophyta | Spermatophyta | Magnoliophyta | eudicotyledons | core eudicotyledons | asterids | lamiids | Solanales | Solanaceae | Solanoideae | Solaneae | Solanum | Lycopersicon
Reference/s:
GenBank - NIH genetic sequence database: GenBank species TBLASTN
GenBank - NIH genetic sequence database: GenBank species TBLASTN WGS
Kazusa DNA Research Institute: Tomato SBM Database
The Gene Index Project: DFCI Tomato (Lycopersicon esculentum) Gene Index
Publication/s:
Yamamoto N *et. al.* , *Gene* , **356** , 127 (2005).

***Glycine max* (Glm)**
Taxonomy:
cellular organisms | Eukaryota | Viridiplantae | Streptophyta | Streptophytina | Embryophyta | Tracheophyta | Euphyllophyta | Spermatophyta | Magnoliophyta | eudicotyledons | core eudicotyledons | rosids | fabids | Fabales | Fabaceae | Papilionoideae | Phaseoleae | Glycine
Reference/s:
DOE Joint Genome Institute: Glycine max
GenBank - NIH genetic sequence database: GenBank species TBLASTN WGS
International Species Sequencing Consortium: Glycine Sequencing Consortium
Legume base: Glycine max
Phytozome: Glycine max (Soybean)
The Gene Index Project: DFCI Soybean (Glycine max) Gene Index
Publication/s:
Kim MY *et. al.* , *Proc Natl Acad Sci U S A* , **107** , 22032 (2010).

***Medicago truncatula* (Mt)**
Taxonomy:
cellular organisms | Eukaryota | Viridiplantae | Streptophyta | Streptophytina | Embryophyta | Tracheophyta | Euphyllophyta | Spermatophyta | Magnoliophyta | eudicotyledons | core eudicotyledons | rosids | fabids | Fabales | Fabaceae | Papilionoideae | Trifolieae | Medicago
Reference/s:
GenBank - NIH genetic sequence database: GenBank species TBLASTN WGS
International Species Sequencing Consortium: Medicago truncatula Sequencing Consortium
Medicago Sequencing Resources: Medicago truncatula Sequencing Resources
Phytozome: Medicago truncatula (Barrel medic)
The Gene Index Project: DFCI Medicago truncatula Gene Index
Publication/s:
Young ND *et. al.* , *Nature* , **480** , 520 (2011).

***Ricinus communis* (Ric)**
Taxonomy:
cellular organisms | Eukaryota | Viridiplantae | Streptophyta | Streptophytina | Embryophyta | Tracheophyta | Euphyllophyta | Spermatophyta | Magnoliophyta | eudicotyledons | core eudicotyledons | rosids | fabids | Malpighiales | Euphorbiaceae | Acalyphoideae | Acalypheae | Ricinus
Reference/s:
GenBank - NIH genetic sequence database: GenBank species TBLASTN WGS

International Species Sequencing Consortium: Ricinus Sequencing Consortium
Phytozome: Ricinus communis (Castor bean plant)
Publication/s:
Chan AP *et. al.* , *Nat Biotechnol* , **28** , 951 (2010).

Populus trichocarpa **(Pot)**
Taxonomy:
cellular organisms | Eukaryota | Viridiplantae | Streptophyta | Streptophytina | Embryophyta | Tracheophyta | Euphyllophyta | Spermatophyta | Magnoliophyta | eudicotyledons | core eudicotyledons | rosids | fabids | Malpighiales | Salicaceae | Saliceae | Populus
Reference/s:
DOE Joint Genome Institute: Populus trichocarpa
DOE Joint Genome Institute: Populus trichocarpa v1.1
GenBank - NIH genetic sequence database: GenBank species TBLASTN WGS
International Species Sequencing Consortium: Populus trichocarpa Sequencing Consortium
Phytozome: Populus trichocarpa (Western poplar)
PopulusDB: PopulusDB
The Gene Index Project: DFCI Populus (Poplar) Gene Index
e! Ensembl: Populus trichocarpa
Publication/s:
Tuskan GA *et. al.* , *Science* , **313** , 1596 (2006).
Sterky F *et. al.* , *Proc Natl Acad Sci U S A* , **101** , 13951 (2004).

Brassica rapa **(Brr)**
Taxonomy:
cellular organisms | Eukaryota | Viridiplantae | Streptophyta | Streptophytina | Embryophyta | Tracheophyta | Euphyllophyta | Spermatophyta | Magnoliophyta | eudicotyledons | core eudicotyledons | rosids | malvids | Brassicales | Brassicaceae | Brassiceae | Brassica
Reference/s:
Brassica database (BRAD): Brassica database
GenBank - NIH genetic sequence database: GenBank species TBLASTN WGS
L'Institut National de la Recherche Agronomique: 5X French Brassica rapa
Phytozome: Brassica rapa (Turnip mustard)
brassica.info: The Multinational Brassica Genome Project

Arabidopsis thaliana **(At)**
Taxonomy:
cellular organisms | Eukaryota | Viridiplantae | Streptophyta | Streptophytina | Embryophyta | Tracheophyta | Euphyllophyta | Spermatophyta | Magnoliophyta | eudicotyledons | core eudicotyledons | rosids | malvids | Brassicales | Brassicaceae | Camelineae | Arabidopsis
Reference/s:
GenBank - NIH genetic sequence database: GenBank species TBLASTN
International Species Sequencing Consortium: Arabidopsis thaliana Sequencing Consortium
Kazusa DNA Research Institute: Arabidopsis thaliana EST Index
National Center for Biotechnology Information Reference Sequences: BLAST Arabidopsis thaliana Sequences
Phytozome: Arabidopsis thaliana (Thale cress)
RIKEN Arabidopsis Genome Encyclopedia: Arabidopsis resource data
TAIR The Arabidopsis Information Resource: The Arabidopsis Information Resource
The Gene Index Project: DFCI Arabidopsis thaliana Gene Index
The Institute for Genomic Research: The TIGR Arabidopsis thaliana Database
e! Ensembl: Arabidopsis thaliana (Arabidopsis thaliana)
Publication/s:
Schmid KJ *et. al.* , *Genome Res* , **13** , 1250 (2003).
Seki M *et. al.* , *Science* , **296** , 141 (2002).
Tabata S *et. al.* , *Nature* , **408** , 823 (2000).
Asamizu E *et. al.* , *DNA Res* , **7** , 175 (2000).
Theologis A *et. al.* , *Nature* , **408** , 816 (2000).
Arabidopsis Genome Initiative , *Nature* , **408** , 796 (2000).
Salanoubat M *et. al.* , *Nature* , **408** , 820 (2000).
Lin X *et. al.* , *Nature* , **402** , 761 (1999).
Mayer K *et. al.* , *Nature* , **402** , 769 (1999).

Arabidopsis lyrata **(Arl)**
Taxonomy:
cellular organisms | Eukaryota | Viridiplantae | Streptophyta | Streptophytina | Embryophyta | Tracheophyta | Euphyllophyta | Spermatophyta | Magnoliophyta | eudicotyledons | core eudicotyledons | rosids | malvids | Brassicales | Brassicaceae | Camelineae | Arabidopsis
Reference/s:
DOE Joint Genome Institute: Arabidopsis lyrata
GenBank - NIH genetic sequence database: GenBank species TBLASTN WGS
International Species Sequencing Consortium: Arabidopsis lyrata Sequencing Consortium
Phytozome: Arabidopsis lyrata (Lyrate rockcress)
e! Ensembl: Arabidopsis lyrata (Arabidopsis lyrata)
Publication/s:
Hu TT *et. al.* , *Nat Genet* , **43** , 476 (2011).

Capsella rubella **(Cpr)**
Taxonomy:
cellular organisms | Eukaryota | Viridiplantae | Streptophyta | Streptophytina | Embryophyta | Tracheophyta | Euphyllophyta | Spermatophyta | Magnoliophyta | eudicotyledons | core eudicotyledons | rosids | malvids | Brassicales | Brassicaceae | Camelineae | Capsella
Reference/s:
Phytozome: Capsella rubella (Red shepherd's purse)

***Eutrema parvulum* (Eup)**
Taxonomy:
cellular organisms | Eukaryota | Viridiplantae | Streptophyta | Streptophytina | Embryophyta | Tracheophyta | Euphyllophyta | Spermatophyta | Magnoliophyta | eudicotyledons | core eudicotyledons | rosids | malvids | Brassicales | Brassicaceae | Eutremeae | Eutrema
Reference/s:
GenBank - NIH genetic sequence database: GenBank species TBLASTN WGS
International Species Sequencing Consortium: Thellungiella parvula Sequencing Consortium
Publication/s:
Dassanayake M *et. al.* , *Nat Genet* , **43** , 913 (2011).

***Eutrema halophilum* (Euh)**
Taxonomy:
cellular organisms | Eukaryota | Viridiplantae | Streptophyta | Streptophytina | Embryophyta | Tracheophyta | Euphyllophyta | Spermatophyta | Magnoliophyta | eudicotyledons | core eudicotyledons | rosids | malvids | Brassicales | Brassicaceae | Eutremeae | Eutrema
Reference/s:
Phytozome: Thellungiella halophila (Salt cress)

***Carica papaya* (Cip)**
Taxonomy:
cellular organisms | Eukaryota | Viridiplantae | Streptophyta | Streptophytina | Embryophyta | Tracheophyta | Euphyllophyta | Spermatophyta | Magnoliophyta | eudicotyledons | core eudicotyledons | rosids | malvids | Brassicales | Caricaceae | Carica
Reference/s:
ASGPB University of Hawaii at Manoa: The Hawaii Papaya Genome Project
GenBank - NIH genetic sequence database: GenBank species TBLASTN WGS
International Species Sequencing Consortium: Carica papaya Sequencing Consortium
Phytozome: Carica papaya (Papaya)
Publication/s:
Ming R *et. al.* , *Nature* , **452** , 991 (2008).

***Theobroma cacao* (Thc)**
Taxonomy:
cellular organisms | Eukaryota | Viridiplantae | Streptophyta | Streptophytina | Embryophyta | Tracheophyta | Euphyllophyta | Spermatophyta | Magnoliophyta | eudicotyledons | core eudicotyledons | rosids | malvids | Malvales | Malvaceae | Byttnerioideae | Theobroma
Reference/s:
Cacao Genome Database: Cacao Genome Sequencing
GenBank - NIH genetic sequence database: GenBank species TBLASTN
International Species Sequencing Consortium: Cacao Sequencing Consortium
The Gene Index Project: DFCI Theobroma cacao (Cocoa) Gene Index
Publication/s:
Argout X *et. al.* , *Nat Genet* , **43** , 101 (2011).
Jones PG *et. al.* , *Planta* , **216** , 255 (2002).

***Gossypium raimondii* (Gor)**
Taxonomy:
cellular organisms | Eukaryota | Viridiplantae | Streptophyta | Streptophytina | Embryophyta | Tracheophyta | Euphyllophyta | Spermatophyta | Magnoliophyta | eudicotyledons | core eudicotyledons | rosids | malvids | Malvales | Malvaceae | Malvoideae | Gossypium
Reference/s:
Phytozome: Gossypium raimondii

***Eucalyptus camaldulensis* (Euc)**
Taxonomy:
cellular organisms | Eukaryota | Viridiplantae | Streptophyta | Streptophytina | Embryophyta | Tracheophyta | Euphyllophyta | Spermatophyta | Magnoliophyta | eudicotyledons | core eudicotyledons | rosids | malvids | Myrtales | Myrtaceae | Eucalyptus
Reference/s:
GenBank - NIH genetic sequence database: GenBank species TBLASTN WGS
Kazusa DNA Research Institute: Eucalyptus camaldulensis Genome Database

***Eucalyptus grandis* (Eug)**
Taxonomy:
cellular organisms | Eukaryota | Viridiplantae | Streptophyta | Streptophytina | Embryophyta | Tracheophyta | Euphyllophyta | Spermatophyta | Magnoliophyta | eudicotyledons | core eudicotyledons | rosids | malvids | Myrtales | Myrtaceae | Eucalyptus
Reference/s:
Phytozome: Eucalyptus grandis (Eucalyptus)

***Citrus sinensis* (Cts)**
Taxonomy:
cellular organisms | Eukaryota | Viridiplantae | Streptophyta | Streptophytina | Embryophyta | Tracheophyta | Euphyllophyta | Spermatophyta | Magnoliophyta | eudicotyledons | core eudicotyledons | rosids | malvids | Sapindales | Rutaceae | Citrus
Reference/s:
Phytozome: Citrus sinensis (Sweet orange)

***Citrus clementina* (Cic)**
Taxonomy:
cellular organisms | Eukaryota | Viridiplantae | Streptophyta | Streptophytina | Embryophyta | Tracheophyta | Euphyllophyta | Spermatophyta | Magnoliophyta | eudicotyledons | core eudicotyledons | rosids | malvids | Sapindales | Rutaceae | Citrus
Reference/s:
GenBank - NIH genetic sequence database: GenBank species TBLASTN
Phytozome: Citrus clementina (Clementine)
Publication/s:
Forment J *et. al.* , *Plant Mol Biol* , **57** , 375 (2005).

***Vitis vinifera* (Vv)**
Taxonomy:
cellular organisms | Eukaryota | Viridiplantae | Streptophyta | Streptophytina | Embryophyta | Tracheophyta | Euphyllophyta | Spermatophyta | Magnoliophyta | eudicotyledons | core eudicotyledons | rosids | rosids incertae sedis | Vitales | Vitaceae | Vitis
Reference/s:
GenBank - NIH genetic sequence database: GenBank species TBLASTN
GenBank - NIH genetic sequence database: GenBank species TBLASTN WGS
International Species Sequencing Consortium: Vitis vinifera Sequencing Consortium
National Center for Biotechnology Information Reference Sequences: Vitis vinifera (wine grape) genome view
Phytozome: Vitis vinifera (Wine grape)
The Gene Index Project: DFCI Vitis vinifera (Grape) Gene Index
e! Ensembl: Vitis vinifera
Publication/s:
Velasco R *et. al.* , *PLoS ONE* , **2** , e1326 (2007).
Jaillon O *et. al.* , *Nature* , **449** , 463 (2007).
Moser C *et. al.* , *Funct Integr Genomics* , **5** , 208 (2005).

***Fragilariopsis cylindrus* (Frc)**
Taxonomy:
cellular organisms | Eukaryota | stramenopiles | Bacillariophyta | Bacillariophyceae | Bacillariophycidae | Bacillariales | Bacillariaceae | Fragilariopsis
Reference/s:
DOE Joint Genome Institute: Fragilariopsis cylindrus

***Phaeodactylum tricornutum CCAP1055/1* (Pht)**
Taxonomy:
cellular organisms | Eukaryota | stramenopiles | Bacillariophyta | Bacillariophyceae | Bacillariophycidae | Naviculales | Phaeodactylaceae | Phaeodactylum
Reference/s:
DOE Joint Genome Institute: Phaeodactylum tricornutum
DOE Joint Genome Institute: Phaeodactylum tricornutum v2.0
International Species Sequencing Consortium: Phaeodactylum tricornutum Sequencing Consortium
National Center for Biotechnology Information: NCBI Eukaryotic Genomes Project
e! Ensembl: Phaeodactylum tricornutum
Publication/s:
Bowler C *et. al.* , *Nature* , **456** , 239 (2008).

***Thalassiosira pseudonana CCMP1335* (Thp)**
Taxonomy:
cellular organisms | Eukaryota | stramenopiles | Bacillariophyta | Coscinodiscophyceae | Thalassiosirophycidae | Thalassiosirales | Thalassiosiraceae | Thalassiosira | Thalassiosira pseudonana
Reference/s:
DOE Joint Genome Institute: Thalassiosira pseudonana
International Species Sequencing Consortium: Thalassiosira pseudonana Sequencing Consortium
National Center for Biotechnology Information: NCBI Protozoa genomes
e! Ensembl: Thalassiosira pseudonana
Publication/s:
Armbrust EV *et. al.* , *Science* , **2004** , 79 (2004).

***Blastocystis hominis* (Bh)**
Taxonomy:
cellular organisms | Eukaryota | stramenopiles | Blastocystis
Reference/s:
International Species Sequencing Consortium: Blastocystis Sequencing Consortium
National Center for Biotechnology Information: NCBI Protozoa genomes
TBestDB - Taxonomically Broad EST Database: Blastocystis hominis
Publication/s:
Denoeud F *et. al.* , *Genome Biol* , **12** , R29 (2011).

***Albugo laibachii Nc14* (All)**
Taxonomy:
cellular organisms | Eukaryota | stramenopiles | Oomycetes | Albuginales | Albuginaceae | Albugo | Albugo laibachii
Reference/s:
GenBank - NIH genetic sequence database: GenBank species TBLASTN
International Species Sequencing Consortium: Albugo Sequencing Consortium
Publication/s:
Kemen E *et. al.* , *PLoS Biol* , **9** , e1001094 (2011).

***Hyaloperonospora arabidopsidis Emoy2* (Hya)**
Taxonomy:
cellular organisms | Eukaryota | stramenopiles | Oomycetes | Peronosporales | Peronosporaceae | Hyaloperonospora | Hyaloperonospora parasitica sensu lato
Reference/s:
International Species Sequencing Consortium: Hyaloperonospora Sequencing Consortium
National Center for Biotechnology Information: NCBI Protozoa genomes
The Genome Sequencing Center at Washington University: Hyaloperonospora parasitica Sequencing
Publication/s:
Baxter L *et. al.* , *Science* , **330** , 1549 (2010).

***Phytophthora ramorum Pr102* (Phr)**
Taxonomy:
cellular organisms | Eukaryota | stramenopiles | Oomycetes | Peronosporales | Phytophthora
Reference/s:

DOE Joint Genome Institute: Phytophthora ramorum v1.1
DOE Joint Genome Institute: Phytophthora ramorum
National Center for Biotechnology Information: NCBI Protozoa genomes
Publication/s:
Tyler BM *et. al.* , *Science* , **313** , 1261 (2006).

Phytophthora brassicae **(Pyb)**
Taxonomy:
cellular organisms | Eukaryota | stramenopiles | Oomycetes | Peronosporales | Phytophthora

Phytophthora sojae P6497 **(Phs)**
Taxonomy:
cellular organisms | Eukaryota | stramenopiles | Oomycetes | Peronosporales | Phytophthora
Reference/s:
DOE Joint Genome Institute: Phytophthora sojae v3.0
DOE Joint Genome Institute: Phytophthora sojae v1.1
DOE Joint Genome Institute: Phytophthora sojae
National Center for Biotechnology Information: NCBI Protozoa genomes
Publication/s:
Tyler BM *et. al.* , *Science* , **313** , 1261 (2006).

Phytophthora infestans T30-4 **(Phi)**
Taxonomy:
cellular organisms | Eukaryota | stramenopiles | Oomycetes | Peronosporales | Phytophthora | Phytophthora infestans
Reference/s:
Broad Institute of Harvard and MIT: Phytophthora infestans Database
GenBank - NIH genetic sequence database: GenBank species TBLASTN
International Species Sequencing Consortium: Phytophthora infestans Sequencing Consortium
National Center for Biotechnology Information: NCBI Protozoa genomes
The Wellcome Trust Sanger Institute: Phytophthora infestans BAC Sequencing
Publication/s:
Raffaele S *et. al.* , *Science* , **330** , 1540 (2010).
Haas BJ *et. al.* , *Nature* , **461** , 393 (2009).
Randall TA *et. al.* , *Mol Plant Microbe Interact* , **18** , 229 (2005).

Pythium ultimum DAOM BR144 **(Pu)**
Taxonomy:
cellular organisms | Eukaryota | stramenopiles | Oomycetes | Pythiales | Pythiaceae | Pythium | Pythium ultimum
Reference/s:
International Species Sequencing Consortium: Pythium Sequencing Consortium
National Center for Biotechnology Information: NCBI Protozoa genomes
Publication/s:
Levesque CA *et. al.* , *Genome Biol* , **11** , R73 (2010).

Aphanomyces euteiches **(Ape)**
Taxonomy:
cellular organisms | Eukaryota | stramenopiles | Oomycetes | Saprolegniales | Saprolegniaceae | Aphanomyces

Saprolegnia parasitica CBS 223.65 **(Srp)**
Taxonomy:
cellular organisms | Eukaryota | stramenopiles | Oomycetes | Saprolegniales | Saprolegniaceae | Saprolegnia | Saprolegnia parasitica
Reference/s:
National Center for Biotechnology Information: NCBI Protozoa genomes

Ectocarpus siliculosus **(Ecs)**
Taxonomy:
cellular organisms | Eukaryota | stramenopiles | PX clade | Phaeophyceae | Ectocarpales | Ectocarpaceae | Ectocarpus
Reference/s:
International Species Sequencing Consortium: Ectocarpus Sequencing Consortium
National Center for Biotechnology Information: NCBI Protozoa genomes
Publication/s:
Cock JM *et. al.* , *Nature* , **465** , 617 (2010).

Aureococcus anophagefferens **(Aua)**
Taxonomy:
cellular organisms | Eukaryota | stramenopiles | Pelagophyceae | Aureococcus
Reference/s:
DOE Joint Genome Institute: Aureococcus anophagefferens
International Species Sequencing Consortium: Aureococcus anophagefferens Sequencing Consortium
National Center for Biotechnology Information: NCBI Protozoa genomes
Publication/s:
Gobler CJ *et. al.* , *Proc Natl Acad Sci U S A* , **108** , 4352 (2011).

Trimastix pyriformis **(Tip)**
Taxonomy:
cellular organisms | Eukaryota | unclassified eukaryotes | Trimastix
Reference/s:
TBestDB - Taxonomically Broad EST Database: Trimastix pyriformis
