# Supplementary material for: Evolution of the eukaryotic dynactin complex, the activator of cytoplasmic dynein
Source: BMC Evol Biol. 2012 Jun 22;12:95. doi: 10.1186/1471-2148-12-95 (PMC3583065; doi:10.1186/1471-2148-12-95)
Supplement: Additional file 3 — Phylogenetic tree of dynactin1. The file contains the phylogenetic tree of dynactin1 highlighting the species- and branch-specific gene duplication events. [file 1471-2148-12-95-S3.pdf]

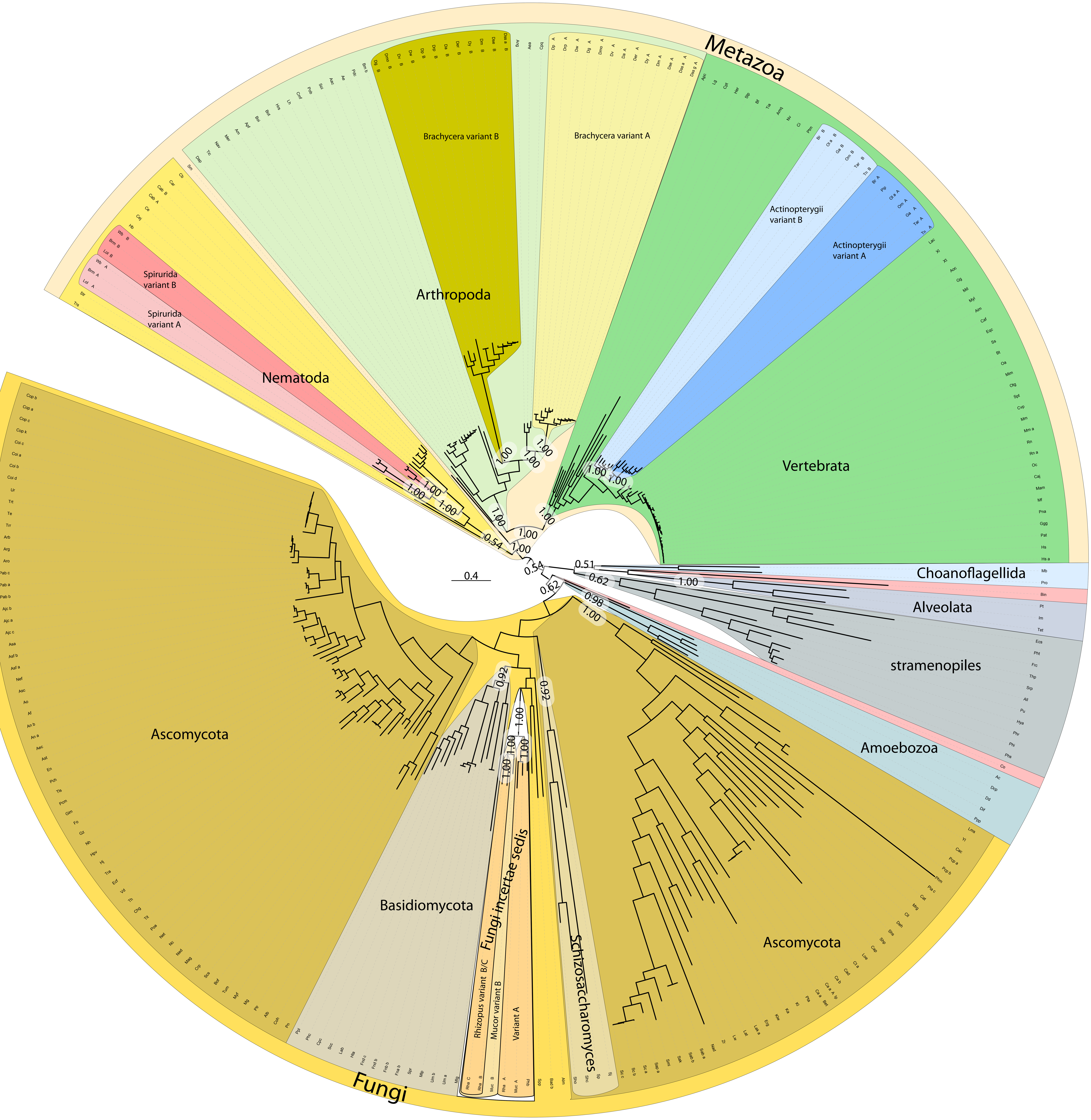

Phylogenetic tree of the dynactin1 subunit

The phylogenetic tree of the dynactin1 from Bayesian inference (MrBayes) using the JTT model. The unrooted tree was drawn with iTOL and branches were coloured according to taxonomic distributions and taxon-specific dynactin1 duplications. Posterior probability support values are only given for some major branches due to space limitations. For the extended tree including the posterior probability values for all branches see Additional File 1. The scale bar represents amino acid substitutions per site.
